# Comparative immunoinformatic analysis of *Rhipicephalus microplus* cocktail vaccine targets

**DOI:** 10.1186/s13071-025-07109-y

**Published:** 2025-12-09

**Authors:** Seham H. M. Hendawy, Heba F. Alzan, Massaro W. Ueti

**Affiliations:** 1 Department of Parasitology and Animal Diseases, Veterinary Research Institute, National Research Centre, 33 El Buhouth St., Dokki, 12622 Cairo Egypt; 2 Tick and Tick-Borne Diseases Research Unit, Veterinary Research Institute, National Research Centre, 33 El Buhouth St., Dokki, 12622 Cairo Egypt; 3 Department of Veterinary Microbiology and Pathology, College of Veterinary Medicine, Washington State University, Pullman, WA 99164-7040 USA; 4 Animal Disease Research Unit, U.S Department of Agriculture—Agricultural Research Service (USDA–ARS), Pullman, WA USA

**Keywords:** *Rhipicephalus microplus*, Vaccine, Immunoinformatic, Bioinformatics, Reverse vaccinology, Peptides cocktail

## Abstract

**Background:**

The cattle fever tick, *Rhipicephalus microplus*, is found in tropical and subtropical regions worldwide. Infestations of this tick lead to significant economic losses for cattle producers and dairy farmers, and the ticks can transmit a variety of pathogens that cause diseases such as babesiosis, anaplasmosis and theileriosis. The proteins Bm86, AQP1, AQP2 and VgR are expressed in various tick tissues, including the gut, salivary glands and ovaries. These proteins regulate essential physiological processes, including water balance (AQP1, AQP2), reproduction (VgR) and cell membrane integrity (Bm86).

**Methods:**

Comprehensive bioinformatic and immunoinformatic analyses were conducted to evaluate Bm86, AQP1, AQP2 and VgR as potential vaccine targets against *R. microplus*. Specifically, we conducted studies on these proteins that included analysis of their physicochemical properties; topographical protein analyses; prediction of N-glycosylation sites, O-glycosylation sites, phosphorylation sites and B-cell and T-cell epitopes; and immune response simulation. The overall aim was to identify key epitopes and highlight their behavior within the host, representing a promising multicomponent vaccine formulation.

**Results:**

The predictions for *R. microplus* Bm86, VgR, AQP1 and AQP2 proteins indicate strong antigenicity, low allergenicity and minimal toxicity, suggesting the potential for safe and effective immune response elicitation. The immune profile simulations for a cocktail of these proteins as vaccine candidates predicted consistently high levels of interferon-gamma and antibody isotypes, which could improve vaccine efficacy and control tick fitness and survivability in subsequent generations.

**Conclusions:**

The application of immunoinformatic tools for anti-tick vaccination was validated for the investigation of combining *R. microplus* Bm86, VgR, AQP1 and AQP2 proteins as a potential cocktail vaccine candidate.

**Graphical abstract:**

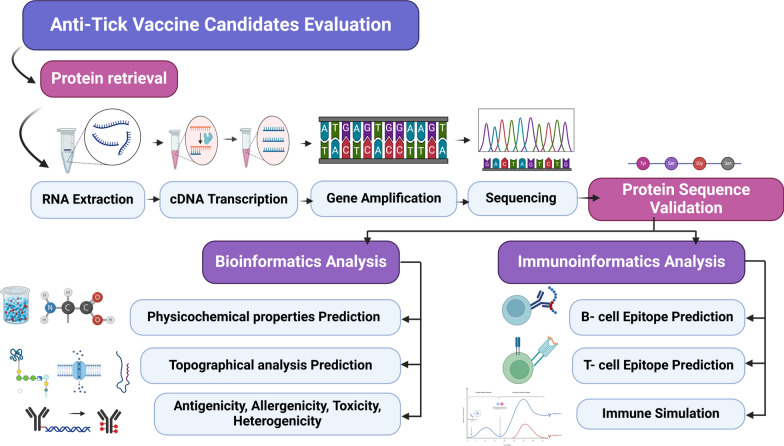

**Supplementary Information:**

The online version contains supplementary material available at 10.1186/s13071-025-07109-y.

## Background

Ticks are blood-feeding ectoparasites that can transmit viruses, bacteria and protozoa, significantly impacting human and animal health [1, 2]. The cattle fever tick, *Rhipicephalus microplus*, is found in tropical and subtropical regions worldwide, and its distribution can expand depending on weather conditions. Infestations of *R. microplus* lead to economic losses for cattle producers and dairy farmers. These losses stem from both direct effects of the infestation, such as anemia, reduced weight gain, decreased milk production and damage to hides [1–3], as well as from the indirect effects of tick-borne diseases, mainly babesiosis, anaplasmosis and theileriosis, that are transmitted by these ticks [4, 5].

The primary strategy for tick control that is currently employed involves the use of acaricides. However, the intensive use of these chemicals is linked to a number of drawbacks, including contamination of meat, milk and the environment, as well as increasingly limited effectiveness in reducing tick populations, often accompanied by the emergence of acaricide-resistant ticks [1, 2, 5]. These issues underscore the need for alternative tick control strategies and encourage scientific research into potential candidates for anti-tick vaccine development [6]. In the early 1990s, a subunit vaccine approach based on Bm86, a membrane-bound glycoprotein found on the gut surface of adult female *R. microplus*, was commercially developed for tick control. The protective mechanism involves the lysis of midgut cells in adult ticks due to the specific humoral anti-Bm86 immunoglobulin G (IgG), which leads to the disruption of blood meal processing and subsequently impairs tick fitness [1, 7, 8]. In addition to Bm86, numerous other tick protective antigens have been identified and explored as potential vaccine candidates [9]. Some of these antigens affect water balance and osmoregulation, both of which are critical for tick physiology and the survival of subsequent generations. Aquaporins (AQPs) play a vital role in efficient tick feeding by regulating the transport of water and small solutes, such as glycerol and urea, across cell membranes. Specific AQPs, such as AQP1 and AQP2, have been identified in various tick species, especially *R. microplus*, and verified as vaccine candidates [10, 11]. The efficacy of AQP1 as a vaccine candidate was tested in cattle, where it demonstrated an impressive efficacy of 68% [10]. In contrast, the AQP2 peptide vaccine for *R. microplus* achieved a limited efficacy of only 25% [11]. Another protein, the vitellogenin receptor (VgR) protein, is crucial for yolk formation in developing tick oocytes and can be targeted to control *R. microplus* infestations. The VgR proteins are involved in vitellogenesis, embryogenesis and the fertility of adult females, thereby impacting embryo development and the survival of future generations [12].

Advanced technologies, such as computer algorithms, machine learning, deep learning systems, immunoinformatics and reverse vaccinology, are paving the way for improved anti-tick vaccine research and development. These approaches can save both animal lives and research time and costs by predicting and computationally validating promising anti-tick vaccine candidates [1, 13]. The integration of machine learning algorithms, such as random forests and artificial neural networks, enhances the accuracy of results and minimizes misleading predictions. Utilizing these computational approaches in vaccine design significantly contributes to the identification of promising candidates and current understanding of host immune responses to various viral and bacterial infectious diseases [14–19]. However, detailed interventions in the field of anti-tick vaccines are still limited [13]. To address these gaps, in this study we have applied bioinformatic and immunoinformatic tools to analyze the physicochemical properties, epitope distribution and immune simulation responses of four key *R. microplus* proteins: Bm86, AQP1, AQP2, and VgR. We investigated these key proteins either as individual or multicomponent formulations to compare their effectiveness as anti-tick vaccine candidates. Based on the results, we hypothesize that utilizing multiple proteins with diverse physicochemical and topographical properties, along with a robust immune response due to their high antigenicity and strong generation of B-cell and T-cell epitopes, will enhance the vaccine's protective efficacy.

## Methods

### Study design

**Fig. 1:**
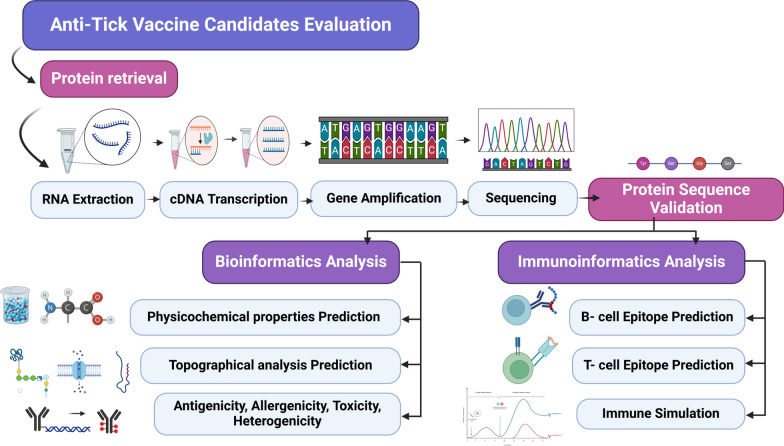
Schematic representation of the work pipeline that was applied in the current work. Created by BioRender

To assess antigenicity, structural features, and immune response simulation of *R. microplus* Bm86, AQP1, AQP2, and VgR proteins as a cocktail vaccine, the following bioinformatic and immunoinformatic analyses were performed according to Ullah et al. [13], with some modifications, as shown in Fig. 1.

### RNA extraction, and cloning and sequencing of *Bm86*, *AQP1*, *AQP2* and *VgR*

Thirty partially fed and fully engorged *R. microplus* La Minita strain tick females were collected from calves ethically utilized for maintaining this strain [11]. The methodology outlined by Hendawy et al. [20] was used to dissect the salivary glands (SG), guts (GT) and ovaries (OV), which were preserved in RNAlater® (catalog [cat.] no. R0901; Sigma-Aldrich, St. Louis, MO. USA) at − 20 °C. The PureLink RNA Mini Kit (cat. no. 12183018A; Thermo Fisher Scientific, Waltham, MA, USA) was used to extract *Bm86* RNA from the dissected, partially fed GT samples; *AQP1* RNA, from replete GT samples; *AQP2* RNA, from partially fed SG; and *VgR* RNA from replete OV. Complimentary DNA (cDNA) was synthesized to serve as a template for the amplification of the full-length coding sequence of targeted genes (*Bm86*, *AQP1*, *AQP2* and *VgR*), using the qScript Ultra SuperMix Kit (cat. no. 95,217–025; Quantabio, Beverly, MA) along with the designed primers listed in Table 1. The PCR cycling program for the *AQP1* and *AQP2* genes were: an initial denaturing step at 95 °C for 3 min; 34 cycles of denaturation at 95 °C for 30 s, annealing at 60 °C for 30 s for *AQP1*, and at 56 °C for 30 s for *AQP2* and extension at 72 °C for 30 s; and a final extension step at 72 °C for 5 min, using Sigma 2× JumpStart™ REDTaq® ReadyMix™ (Sigma-Aldrich). For the *Bm86* gene, the PCR cycling conditions were: an initial denaturing step at 95 °C for 3 min; 24 cycles of denaturation at 95 °C for 45 s, annealing at 55 °C for 30 s and extension at 72 °C for 45 s; and a final extension step at 72 °C for 7 min, using the same PCR mix. For the*VgR* gene, the PCR cycling conditions were: an initial denaturing step at 98 °C for 30 s; 34 cycles of denaturation at 98 °C for 10 s, annealing at 60 °C for 10 s and extension at 72 °C for 3 min; and a final extension step at 72 °C for 10 min, the using 2× Phusion High-Fidelity PCR Master Mix® (cat. no. F531L; Thermo Fisher Scientific). To obtain the complete sequences of the amplified transcripts for *Bm86*, *AQP1* and *AQP2*, the TOPO TA Cloning Kit (pCR® 4-TOPO® vector; Invitrogen, Thermo Fisher Scientific) and One Shot® TOP10 chemically competent cells were utilized; for *VgR*, we used the TOPO XL® Invitrogen Cloning Kit) and One Shot™ OmniMAX™ 2 T1^R^ chemically competent *Escherichia coli* cells (both Thermo Fisher Scientific). The manufacturers’ instructions on the incubation conditions for transformation and colony selection were followed. The resultant sequences were translated using the ExPASy Translate tool. The translated Bm86, AQP1, AQP2 and VgR proteins from *R. microplus* were submitted to GenBank under accession numbers PQ030824 for Bm86, PQ030825 for AQP1, PQ030826 for AQP2 and PQ030827 for VgR. These sequences were then subjected to a BLAST search against *R. microplus* (taxid: 6941) on the NCBI-BLASTp website (https://blast.ncbi.nlm.nih.gov/Blast), using the non-redundant protein sequences standard database. Default parameters and thresholds were applied, with the expectation value (E-value) maintained at 0.005 [21].

**Table 1:** Primers used to amplify the full-length codon gene sequences

Primer name	Sequence	Length (*n* nucleotide)	Annealing temperature (°C)	Amplicon size (bp)
AQP1X1X3-F	ATGAAGATCGAGAACCTGCTC	21	60	897
AQP1X1X3-R2	CAGCCGCTGGTGCTGGGTAGGTTC	24		
AQP2-F	ATGAAGCCCAACACCGTGACC	21	56	879
AQP2-R1	GGCGCAGATCTTCGCATTTGA	21		
BM86-F1	ATGCGTGGCATCGCTTTGTTC	21	55	1950
BM86-R1	CAACGATGCTGCGGTGACTGA	21		
VgR-F	ATGAAGTTTACCGCCTGTGC	20	60	5397
VgR1-R1	GTGCTTCCGGAAGAAGAA	18		

*AQP* Aquaporin,* VgR* vitellogenin receptor

### Physicochemical properties prediction

The Expasy ProtParam tool (https://web.expasy.org/protparam/; accessed 16 Aug 2023) was utilized to evaluate the physicochemical properties of the four *R. microplus* proteins (Bm86, AQP1, AQP2, VgR). The properties analyzed included the number of amino acids, molecular weight (MW), aliphatic index, instability index, total counts of positively and negatively charged residues, theoretical isoelectric point (pI), estimated half-life in mammalian cells, yeast cells, *E. coli* and reticulocytes, extinction coefficients and the grand average of hydropathicity (GRAVY) [22].

### Subcellular localization and structural feature prediction

The analysis of protein topographical features provides insights into subcellular localization and functional characteristics. This method was utilized to identify the presence or absence of transmembrane domains, signal peptides and glycosylphosphatidylinositol (GPI) anchors.

#### Transmembrane helices and subcellular localization prediction

DeepTMHMM (https://services.healthtech.dtu.dk/services/DeepTMHMM-1.0/) [23] and DeepLoc 2.0 (https://services.healthtech.dtu.dk/services/DeepLoc-2.1/) [24] were utilized as tools from the DTU Health Tech Bioinformatic Services web server to predict the transmembrane helices and the subcellular localization of the translated Bm86, AQP1, AQP2, and VgR proteins of *R. microplus* with predicted probability ≥ 0.5.

#### Signal peptide and GPI anchor prediction

The SignalP 6.0 server (https://services.healthtech.dtu.dk/services/SignalP-6.0/) [25] was utilized to predict the types of signal peptides that regulate the secretion and translocation of proteins such as Bm86, AQP1, AQP2 and VgR in the cells of *R. microplus* ticks. Additionally, the NetGPI 1.1 predictor (https://services.healthtech.dtu.dk/services/NetGPI-1.1/) [26] was used to identify the presence of GPI anchors, which help maintain the attachment of these proteins to the cell membrane. The GPI anchor can hinder the accessibility of antibodies to conserved protein epitopes, allowing the tick to feed without disturbance. In addition, the GPI structure enhances vaccine stability and delivery.

### Secondary structure and solvent accessibility analysis

The NetSurfP 3.0 server (https://services.healthtech.dtu.dk/services/NetSurfP-3.0/), provided by DTU Health Tech's Bioinformatics Services, was used to analyze the structural characteristics of the proteins under investigation, specifically Bm86, AQP1, AQP2 and VgR. This analysis provided information on their secondary structures, surface accessibility and residue disorder [27].

### Post-translational modification sites prediction

Post-translational modification is an important factor to be considered in anti-tick vaccine development. It regulates protein function and behavior within the cell. Consequently, it plays a crucial role in antigen recognition that improves the immunogenicity and enhances vaccine efficacy when choosing an anti-tick vaccine candidate [9].

#### Glycosylation sites prediction

##### N-Glycosylation sites

NetNGlyc–1.0 predicts N-glycosylation sites of the translated Bm86 AQP1, AQP2 and VgR of *R. microplus* proteins with a potential threshold of 0.5 using the following link (https://services.healthtech.dtu.dk/services/NetNGlyc-1.0/) [28].

##### O-Glycosylation sites

The NetOGlyc 4.0 server (https://services.healthtech.dtu.dk/services/NetOGlyc-4.0/) was used to predict mucin-type GalNAc O-glycosylation sites in the translated proteins Bm86, AQP1, AQP2 and VgR from *R. microplus* with a potential score threshold > than 0.5 [29].

#### Phosphorylation sites prediction

The NetPhos 3.1 server (https://services.healthtech.dtu.dk/services/NetPhos-3.1/) [30] was utilized to predict phosphorylation sites for serine, threonine or tyrosine, as well as to identify 17 specific kinases. These kinases include ATM, CKI, CKII, CaMII, DNAPK, EGFR, GSK3, INSR, PKA, PKB, PKC, PKG, RSK, SRC, cdc2, cdk5 and p38MAPK. The predictions were made for the translated proteins AQP1, AQP2, Bm86 and VgR from *R. microplus*.

### Antigenicity prediction

To validate the *R. microplus* proteins Bm86, AQP1, AQP2, and VgR as potential vaccine candidates capable of eliciting a strong immune response following immunization, we conducted an antigenicity prediction. This was performed using the Vaxijen 2.0 server (http://www.ddg-pharmfac.net/vaxijen/VaxiJen/VaxiJen.html), which predicts antigenicity based on the identification of the parasite and uses a threshold for immunogenicity set at 0.7. This threshold corresponds to that of the commercially available anti-tick vaccine Bm86 [31].

### Toxicity prediction

To validate the *R. microplus* proteins Bm86, AQP1, AQP2 and VgR as promising candidates for a safe anti-tick vaccine, it is essential to ensure that they do not contain toxic motifs or peptides that could lead to host toxicity. To assess the potential toxicity of these proteins, we utilized the ToxinPred2 server https://webs.iiitd.edu.in/raghava/toxinpred2/. This web server relies on a comprehensive dataset comprising 8233 toxic and 8233 non-toxic protein sequences, employing machine learning techniques along with BLAST and MERCI tools for prediction [32]. More importantly, the ToxinPred2 server provides a motif scan tool for the protein candidates, which was employed in this study for detailed protein analysis.

### Host and tick species similarity

To prevent any autoimmune reactions during host immunization, we compared the sequences of the *R. microplus* proteins AQP1, AQP2, Bm86 and VgR against the protein sequences of *Bos taurus* (taxid: 9913) available in the National Center for Biotechnology Information (NCBI) database. Additionally, we conducted a comparison of the *R. microplus* AQP1, AQP2, Bm86 and VgR proteins with those from other tick species, including *Ixodes scapularis*, *Ixodes ricinus*, *Haemaphysalis longicornis*, *Dermacentor andersoni*, *Hyalomma dromedarii*, *Rhipicephalus annulatus* and *Rhipicephalus sanguineus*. The aim of this analysis was to gather more information on the potential of these vaccine candidates to provide cross-protection against other tick species. The analysis was conducted using NCBI-BLASTp (https://blast.ncbi.nlm.nih.gov/Blast). Default parameters and thresholds were applied, with the expectation value (E-value) set at 0.005 [21]. To perform multiple sequence alignment, we utilized Clustal2.1 via the Clustal Omega site (Clustal Omega < EMBL-EBI) [33].

### B-Cell and T-cell epitope prediction

Linear B-cell and T-cell epitopes were predicted from the translated protein sequences Bm86, AQP1, AQP2 and VgR to obtain detailed data on the immunogenicity of these proteins as potential vaccine candidates capable of eliciting a strong immune response upon host immunization. For B-cell epitopes, the BepiPred2 tool available on the IEDB server (http://tools.iedb.org/bcell/) was utilized to identify antigenic determinants recognized by B-cells. The default threshold value was set at 0.5, based on the random forest algorithm, which was trained on both epitopes and non-epitope amino acids derived from crystal structures [34]. Bovine CD4 T-cell epitopes were predicted and evaluated for all protein sequences in this study through two steps. In the first step, bovine CD4 T-cell epitopes were predicted using a list of bovine MHC Class II BoLA-DRB3 molecules via the NetMHCIIpan-4.3 server (https://services.healthtech.dtu.dk/services/NetMHCIIpan-4.3/). These molecules are designated as BoLA-DRB3_00101 through BoLA-DRB3_02601 and are well-represented in the NetMHCIIpan-4.3 training data. Strong binding affinity peptides were identified by comparing their scores to those of 100,000 random natural peptides, with a threshold for strong binders set at a rank of 2% for the specified molecules [35]. In the second step, the predicted CD4 T-cell epitopes were evaluated using the interactive web tool named the Epitope Evaluator (https://fuxmanlab.shinyapps.io/Epitope-Evaluator/) [36]. This tool can identify a set of epitopes that may be recognized by various MHC alleles within the population. It presents these epitopes according to their distribution within different binding score ranges among diverse alleles. Furthermore, it can determine proteins containing a high number of predicted epitopes and calculate the epitope density within these proteins, which could serve as vaccine candidates. The tool also enables the identification of shared conserved epitopes across multiple proteins.

### Immune simulation

An in silico immune simulation analysis was conducted using the C-IMMSIM online server (available at http://kraken.iac.rm.cnr.it/C-IMMSIM) to evaluate the immunogenicity and immune response profile of the *R. microplus* AQP1, AQP2, Bm86 and VgR proteins. This server is helping in the prediction of promising vaccine candidates, with a costless approach that provides preliminary anticipated evidence of the potential effectiveness and safety of such candidates. These proteins were assessed both individually and as a cocktail vaccine. The server utilizes a position-specific scoring matrix (PSSM) and machine learning algorithms to predict immune epitopes and their interactions. All parameters were set to default, except for the antigen injection schedule. Three injections were administered at intervals of 1, 21 and 42 days, with each time step of the simulation corresponding to 8 h [37].

## Results

### Physicochemical properties prediction

The analysis of the translated amino acid sequences revealed the lengths and MW for *R. microplus* proteins Bm86 (650 amino acids, MW 71.8 kDa), AQP1 (299 amino acids, MW 32.5 kDa), AQP2 (293 amino acids, MW 30.8 kDa) and VgR (1799 amino acids, MW 19.8 kDa). The theoretical pI calculated for each of these proteins was: 5.72 for Bm86, 6.05 for AQP1, 5.87 for AQP2 and 5.38 for VgR (Table 2). The Bm86 and VgR proteins were classified as slightly unstable proteins, with instability indices > 40 (44.5 for Bm86 and 43.5 for VgR). The aliphatic index of Bm86 was 56.4 and that of VgR was 67.8. The GRAVY values of these two proteins were − 0.579 for Bm86 and − 0.336 for VgR. Conversely, AQP1 and AQP2 were classified as stable proteins, with instability indices of 29.4 and 22.2, respectively. The aliphatic indices for AQP1 and AQP2 were 107.66 and 111.16, respectively, while their GRAVY values were 0.401 and 0.641, respectively (Table 2). In terms of the total number of negatively charged residues (Asp + Glu), there were 93 negatively charged residues in Bm86, 25 in AQP1, 17 in AQP2 and 235 in VgR. In contrast, the total number of positively charged residues (Arg + Lys) was 85 for Bm86, 21 for AQP1, 15 for AQP2 and 156 for VgR. The amino acid composition varied among the proteins, with Cys (C) accounting for 10.3% of the amino acid residues in Bm86, Ile (I) accounting for 10.4% of those in AQP1, both Ala (A) and Leu (L) accounting for 13% each in AQP2 and Ser (S) accounting for 9% of amino acids in VgR. The extinction coefficients, measured in units of M⁻^1^ cm⁻^1^ at 280 nm in water, were 65,435, 46,785, 58,035, and 280,750 for the Bm86, AQP1, AQP2, and VgR proteins, respectively, under the assumption that all pairs of Cys residues form cystines; alternatively, under the assumption that all Cys residues were reduced, the respective extinction coefficients were 61,310, 46,410, 57,410, and 272,500 (Table 2).

**Table 2:** Physicochemical properties of vaccine target translated proteins Bm86, AQP1, AQP2 and VgR

Protein	No. of amino acids	Molecular weight (Da)	pI	Instability Index	Aliphatic Index	Extension coefficient	GRAVY	Half life		
								*Escherichia coli* in vivo (h)	Yeast in vivo (h)	Mammal in vitro (h)
Bm86	650	71,858.29	5.72	44.50	56.45	65,435	− 0.579	> 10	> 20	> 30
AQP1	299	32,595.28	6.05	29.40	107.66	46,785	0.401	> 10	> 20	> 30
AQP2	293	30,891.02	5.87	22.23	111.16	58,035	0.641	> 10	> 20	> 30
VgR	1799	198,893.42	5.38	43.54	67.80	280,750	− 0.336	> 10	> 20	> 30

*AQP* Aquaporin, *GRAVY* grand average of hydropathicity,* VgR* vitellogenin receptor

### Topographical characteristics analysis

#### Transmembrane helices and subcellular localization prediction

The *R. microplus* Bm86 protein does not contain any transmembrane helices throughout its entire sequence (Table 3; Additional file 1: Figure S1). Furthermore, it is classified as an extracellular protein, with a probability of 0.7085, and a cell membrane localization probability of 0.6944 (Table 3). In contrast, the AQP1 and AQP2 proteins from *R. microplus* each possess six transmembrane helices (Table 3; Additional file 1: Figure S1;) and are predicted to be predominantly localized in lysosomes or vacuoles, with probabilities of 0.407 for AQP1 and 0.816 for AQP2. The VgR protein only has one transmembrane helix in its entire sequence. Its localization is predicted to be in the cell membrane, with a probability of 0.7926, and in lysosomes/vacuoles, with a probability of 0.7131 (Table 3; Additional file 1: Figure S1).

#### Signal peptide and GPI anchor prediction

The data analysis revealed that both *R. microplus* Bm86 and VgR contain signal peptides at the N-terminal region of the protein. Specifically, Bm86 has its signal peptide starting at position 1 and ending at position 19, while VgR has its signal peptide starting at position 1 and ending at position 22. The cleavage site for Bm86 occurs between positions 19 and 20, with a probability of 0.977334, whereas for VgR, the cleavage site is between positions 22 and 23, with a probability of 0.981977 (Table 3; Additional file 2: Figure S2). Additionally, Bm86 shows a predicted omega-site S residue identified as a GPI anchor at position 627 (Additional file 3: Figure S3). In contrast, AQP1 and AQP2 lack both a signal peptide and a GPI anchor, similar to VgR (Table 3; Additional file 3: Figure S3).

### Secondary structure and solvent accessibility analysis

The relative surface accessibility (RSA) of the *R. microplus* proteins Bm86, AQP1, AQP2 and VgR was predicted and is shown in Additional file 4: Figure S4. Residues labeled in red are exposed, while those labeled in blue are buried, with a threshold of 25%. Additionally, the secondary structure of the four protein sequences (including helix, strand and coil) is presented in the same figure. The thickness of the gray line indicates the probability of disordered residues within each protein sequence. Notably, both the starting and ending segments of all proteins exhibit a long stretch of disordered residues that is comparable to their overall length in amino acids (Additional file 4: Figure S4) with the following percentages: 16.4% for AQP1, 13% for AQP2, 17.7% for Bm68 and 19.4% for VgR which has starting, ending and intrinsic disordered segments. All of the protein sequences have exposed coil loops that foster strong electrostatic and hydrogen-bond interactions with antibodies.

### Post-translational modification sites prediction

#### Glycosylation sites prediction

##### O-Glycosylation sites prediction

NetOGlyc predicted that only the *R. microplus* VgR and AQP2 proteins are glycosylated and that neither *R. microplus* AQP1 nor Bm86 contains any glycosylation sites. The *R. microplus* VgR protein has 94 GalNAc O-glycosylation sites located at the following positions: 59, 61, 63, 102, 103, 143, 144, 145, 146, 147, 149, 247, 279, 280, 281, 291, 311, 319, 322, 323, 327, 330, 817, 823, 826, 827, 831, 833, 858, 860, 866, 867, 870, 885, 890, 891, 892, 918, 925, 928, 930, 937, 966, 968, 969, 972, 976, 1005, 1006, 1011, 1014, 1035, 1044, 1094, 1095, 1143, 1165, 1172, 1173, 1176, 1177, 1178, 1179, 1185, 1199, 1220, 1224, 1225, 1236, 1243, 1265, 1276, 1283, 1318, 1417, 1450, 1455, 1472, 1494, 1509, 1510, 1538, 1540, 1549, 1553, 1565, 1595, 1614, 1636, 1651, 1659, 1662, 1665 and 1666. *Rhipicephalus microplus* AQP2 has only two GalNAc O-glycosylation sites, at positions 141 and 168.

##### N-Glycosylation sites prediction

NetNGlyc predicted one asparagine N-glycosylation site with high specificity (9/9 jury agreement) at position 141 in the *R. microplus* Bm86 protein, with a potential score of 0.75, and two asparagine N-glycosylation sites, both with high specificity and a potential score of 0.6 (9/9 jury agreement) at positions 104 and 1003 in the *R. microplus* VgR protein. In contrast, the *R. microplus* AQP1 and AQP2 proteins do not possess any Asparagine N-glycosylation sites. Other sites in these proteins may well be glycosylated, but they exhibit lower potential scores of 0.5 and a lower number of jury agreements (Additional file 5: Figure S5).

#### Phosphorylation sites prediction

The NetPhos 3.1 server identified the highest number of phosphorylation sites across various kinases in the VgR protein of *R. microplus*. Specifically, it found 111 serine, 57 threonine and 24 tyrosine phosphorylation sites with 10 kinases: CKI, CKII, DNAPK, EGFR, INSR, PKA, PKG, PKC, cdc2 and cdk5 at different sites along the amino acid sequence. In contrast, the Bm86 protein of *R. microplus* had 28 serine, 25 threonine and 10 tyrosine phosphorylation sites with nine kinases: CKI, CKII, DNAPK, p38 MARK, INSR, PKC, SRC, cdc2 and PKA. In comparison, the AQP1 and AQP2 proteins had a limited number of phosphorylation sites, with AQP1 showing six serine, eight threonine and three tyrosine sites with two kinases (PKC and DNAPK), and AQP2 displaying nine serine, seven threonine and one tyrosine site with three kinases (PKC, PKA and cdc2). In addition, there were multiple phosphorylation sites with unspecified kinases along the amino acid sequences of all proteins. The phosphorylation sites and types are presented in Additional file 6: Figure S6.

### Antigenicity prediction

The predicted data from the Vaxijen-2.0 server indicated that *R. microplus* Bm86 had the highest antigenicity, followed in descending order of antigenicity by AQP1, VgR and AQP2, with respective recorded values of 0.7695, 0.596, 0.470 and 0.589 (Table 3).

**Table 3:** Topographical characteristics analysis of *Rhipicephalus microplus* vaccine target proteins Bm86, AQP1, AQP2 and VgR

Proteins	Subcellular localization	TM helices (*n*)	Signal peptide	GPI-anchor	Antigenicity (VaxiJen 2)^a^	Toxicity
BM86	Extracellular	0	1 (1:19)	1 (627)	0.7695	Non-toxic
AQP1	Lysosome	6	No	No	0.596	Non-toxic
AQP2	Lysosome	6	No	No	0.47	Non-toxic
VgR	Cell membrane and lysosome	1	1 (1:22)	No	0.589	Toxic and non-toxic

*AQP* Aquaporin,* GPI* glycosylphosphatidylinositol,* TM* transmembrane, * VgR* vitellogenin receptor

^a^Antigenicity according to the VaxiJen 2.0 server

### Toxicity prediction

The toxicity of the *R. microplus* proteins AQP1, AQP2, Bm86 and VgR was predicted using the ToxinPred2 server. Toxicity scores were calculated using a default threshold of 0.6, based on a comprehensive database containing 8233 toxic and 8233 non-toxic protein sequences. This analysis employed a combination of machine learning techniques, along with BLAST and MERCI tools. The results indicated that AQP2, AQP1 and Bm86 proteins had the lowest hybrid toxicity scores of − 0.07, − 0.28 and 0.19, respectively. In contrast, the VgR protein showed a higher hybrid toxicity score of 1.36 at the 0.6 threshold; however, it was classified as non-toxic at the threshold of 1 according to the machine learning random forest model (Table 3). Regarding the motif scan, which is based on the MERC tool, *R. microplus* proteins AQP1, AQP2, Bm86 and VgR have no toxic motifs and could be considered safe vaccine candidates.

### Heterogenicity of tick vaccine protein candidates with the host and other tick species

The sequence similarity of the *R. microplus* AQP1, AQP2, Bm86 and VgR proteins was predicted against query sequences from multiple tick species (*I. scapularis*, *I. ricinus*; *H. longicornis*; *D. andersoni*, *H. drommedarii*, *R. annulatus* and *R. sanguineous*) other than *R. microplus*, and against *Bos taurus* as a host; these protein sequences are available on the NCBI database. Multiple sequence alignment identity matrices revealed the highest similarity (> 90%) among *R. microplus*, *R. annulatus* and *R. sanguineous* proteins according to the NCBI-BLASTp analysis (Additional file 7: Figure S7). While the NCBI-BLASTp analysis of the *Bos taurus* protein sequence showed no significant homology with *R. microplus* Bm86, AQP1, AQP2 and VgR proteins (Additional file 7: Figure S7). The similarity of protein sequence regions that might raise autoimmune concerns with the *Bos taurus* protein sequence or offer cross-protective potential with multiple tick species are presented in Additional file 8: Dataset S1 as follows:

- Bm86 sequence identity matrix for other tick species and *Bos taurus*. **1:** hemicentin-2_isoform_X1_[*Bos tartus*] (XP_010808812.1), **2:** Bm86 *I. scapularis* (XP_040071062.1), **3:** Bm86 *I. ricinus* (ADR01316.1), **4:** Bm86 *H. longicornis* (BAF56919.1), **5:** Bm86 *D. andersoni* (XP_050035013.1), **6:** Bm86 *H. drommedarii* (ADY76577.1), **7:** Bm86 *R. sanguineous* (ABN11119.1), **8:** Bm86 *R. microplus* (PQ030824) and **9:** Bm86 *R. annulatus* (ACL27210.1) showed the highest similarity of 93.52% with the *R. annulatus* (ACL27210.1), followed by 72.02% with *R. sanguineous* (ABN11119.1), and the lowest similarity of 32.11% with *I. ricinus* (ADR01316.1) (Additional file 7: Figure S7). The hemicentin-2_isoform_X1_[*Bos tartus*] (XP_010808812.1) protein sequence presented a similarity of 21.08% (Additional file 7: Figure S7).
- AQP1 sequence identity matrix for other tick species and *Bos taurus*. **1:** aquaporin [*Bos taurus*] (NP_001192762.2), **2:** AQP1 *I. ricinus* (CAX48964.1), **3:** AQP1 *I. scapularis* (XP_029845132.1), **4:** Hypothetical *H. longicornis* (KAH9370733.1), **5:** AQP1 *D. andersoni* (XP_050044617.1), **6:** AQP1 *R. microplus* (PQ030825) and **7:** AQP1 *R. sanguineous* (XP_037510823.1) represented the highest similarity of 92.28% with AQP1 *R. sanguineous* (XP_037510823.1.), and the lowest similarity of 43.75% showed with *I. ricinus* (CAX48964.1). The aquaporin [*Bos taurus*] (NP_001192762.2) protein sequence presented a similarity of 38.04% (Additional file 7: Figure S7).
- AQP2 sequence identity matrix for other tick species and *Bos taurus*. **1:** aquaporin 9 [*Bos taurus*] (NP_001192762.2), **2:** AQP2 *I. ricinus* (CAX48964.1), **3:** AQP2 *I. scapularis* (XP_029851087.2), **4:** hypothetical *H. longicornis* (KAH9380169.1), **5:** AQP2 *D. andersoni* (XP_050050937.1), **6:** AQP2 *R. microplus* (PQ030826), **7:** AQP2 *R. sanguineous* (XP_037518224.1) represented the maximum similarity of 92.44% with AQP2 *R. sanguineous* (XP_037518224.1), and the lowest similarity of 54.03% with *I. ricinus* (CAX48964.1). Aquaporin 9 [*Bos taurus*] (NP_001192762.2) presented a similarity of 35.34% (Additional file 7: Figure S7).
- VgR sequence identity matrix for other tick species and *Bos taurus*. **1:** VgR *R. annulatus*_Bm86-like (ACR19242.1), **2:** VgR *H. drommedarii* _Bm86-like (ADY76577.1), **3:** Low Denisty Lipo-Protein (LDLP) [*Bos taurus*] (XP_059734109.1), **4:** VgR *I. scapularis* (XP_029830196.2), **5:** VgR *H. longicornis* (BAG14342.1), **6:** VgR *D. andersoni* (XP_050052543.1), **7:** VgR *R. microplus* (PQ030827), **8:** VgR *R. sanguineous* (XP_037521270.1) showed the highest similarity of 92.38% with the VgR *R. sanguineous* (XP_037521270.1), and the lowest similarity of 19.53% with VgR *R. annulatus*_Bm86-like (ACR19242.1). The LDLP_[*Bos taurus*] (XP_059734109.1) protein sequence presented a similarity of 33.35% (Additional file 7: Figure S7).

### B-cell Epitope prediction

Linear B-cell epitopes were predicted from the amino acid sequences of the *R. microplus* Bm86, AQP1, AQP2 and VgR proteins. The average of the residue scores was almost similar for the Bm86 and VgR proteins (0.529 and 0.520, respectively), and was 0.429 and 0.438 for AQP1 and AQP2, respectively (Table 4; Additional file 9: Figure S8). The predicted B-cell peptides numbers and positions were predicted as 15, five, five and 24 peptides for the Bm86, AQP1, AQP2 and VgR translated sequence proteins, respectively (Table 4; Additional file 10: Tables S1, S2, S3 and S4).

**Table 4:** B-cell epitope residue scores

Protein	Residue scores			Predicted peptide number
	Average	Minimum	Maximum	
Bm86	0.529	0.221	0.654	15
AQP1	0.429	0.241	0.688	5
AQP2	0.438	0.245	0.654	6
VgR	0.520	0.191	0.705	24

*AQP* Aquaporin, * VgR* vitellogenin receptor

### T-cell epitope prediction

**Fig. 2:**
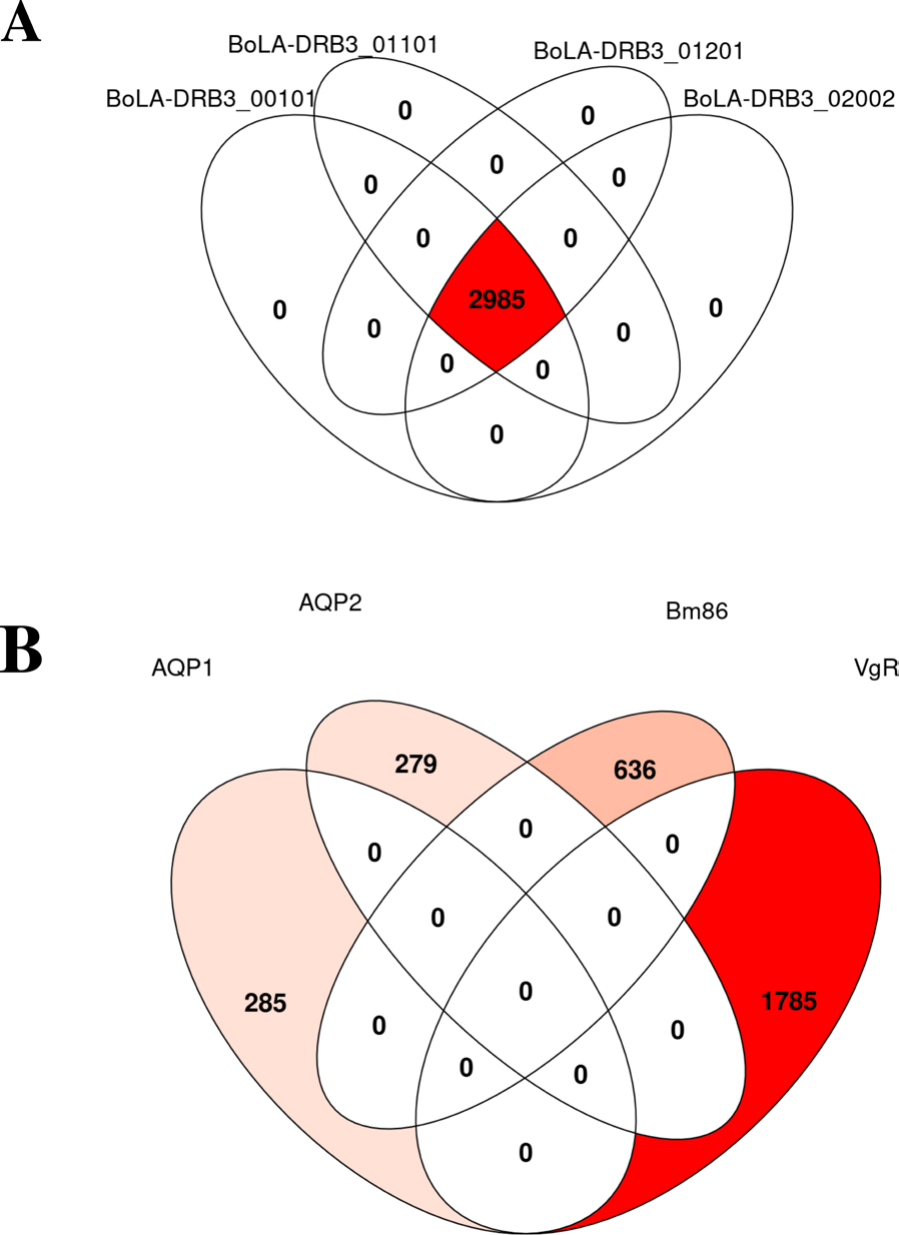
**A** Venn diagram representing the epitope intersection tool, with the number of epitopes predicted to bind to different MHC allele combinations. **B** Venn diagram representing no shared conserved epitopes among the protein sequences Bm86, AQP1, AQP2 and VgR. *AQP* Aquaporin, * VgR* vitellogenin receptor

For this prediction, we first predicted the bovine CD4 T-cell epitopes for *R. microplus* Bm86, AQP1, AQP2, and VgR amino acid sequences. The strong binding peptides were identified as having a threshold rank ≤ 2%. The starting position offset and the optimal binding core had been presented for all protein sequences with the percentile rank for each BoLA-DRB3 MS-COVERED T-cell allele. Secondly, the output data of predicted bovine CD4 T-cell epitopes (Additional file 11: Dataset S2) in all proteins were distributed using the epitope evaluator. The number of shared epitopes in the targeted protein sequences under study that bind to BoLA-DRB3_00101, BoLA-DRB3_01101, BoLA-DRB3_01201, BoLA-DRB3_01601, BoLA-DRB3_01801 and BoLA-DRB3_02002 MHC II alleles are presented in Additional file 12: Figure S9. In order to select the strongest binding epitopes, the parameters were set so that strong binder epitopes were only considered using 0% as the minimum percentile rank and 2% as the maximum percentile rank, with 0.1 as a value for the width of the bins in the plot and the heatmap. A total of 2985 epitopes were predicted as potential vaccine candidates by the following MHC allele combinations: BoLA-DRB3_00101, BoLA-DRB3_01101, BoLA-DRB3_01201 and BoLA-DRB3_02002 (Fig. 2a). All protein sequences presented a good epitope density, ranging from 0.95 to 0.99 when the number of epitopes were predicted in correlation to their protein sequence length to bind combination of MHC II alleles (BoLA-DRB3_00101, BoLA-DRB3_01101, BoLA-DRB3_01201 and BoLA-DRB3_02002), while the densities ranged from 0.01 to 0.05 when the epitopes were predicted to bind to MCH II BoLA-DRB3_01601, and BoLA-DRB3_01801. The maximum cutoff percentile rank to consider a peptide as an epitope was 2% (Additional file 13: Figure S10). However, there are no conserved shared epitopes among the predicted epitopes from the four protein sequences Bm86, AQP1, AQP2 and VgR (Fig. 2b). It is worth noting that the most promiscuous strong binder bovine CD4 T-cell epitopes revealed an overlap with glycosylation and/or phosphorylation sites, as the following: two out of 12 for Bm68; one out of 37 for AQP1; and 26 out of 115 for VgR (Additional file 10: Table S5). A 16 CD4 T-cell epitope of AQP2 was predicted to be a strong binder; however, it does not have any overlap with post-translational modification sites.

### Immune system simulation

The simulated immune profile for two case scenarios over 104 days, either as a subunit vaccine based on an individual *R. microplus* protein (AQP1, AQP2, Bm86 and VgR) or as a cocktail vaccine that includes the four proteins, revealed polarized cell activities of the immune responses. Both primary and adaptive immune responses were elicited. An expanded immunological memory with a good antigen clearance was predicted (Additional file 14: Figure S11; Additional file 15: Figure S12).

With the prime dose injection, for all individual protein vaccines the total macrophage and dendritic cells populations showed a fluctuating level ranging from 140 cells/mm^3^ to 195 and 195 cells/mm^3^, respectively. For the cocktail injection, the prediction was a consistent level of 195 cells/mm^3^ along the immunization schedule (Additional file 16: Figure S13; Additional file 17: Figure S14).

With the second booster dose of the immunization program, the active CD8 T-cytotoxic lymphocyte count reached its maximum level at 600 cells/mm^3^ with the *R. microplus* Bm86 vaccine candidate (Additional file 18: Figure S15A) and 1100 cells/mm^3^ with the other *R. microplus* individual vaccine candidates (AQP1, AQP2 and VgR), on day 40 (Additional file 18: Figure S15B, C, D). For all individual vaccine candidates, the count declined on day 80. The level of the active CD8 T-cytotoxic lymphocyte count in the latter three candidates reached its maximum level of 1100 cells/mm^3^ on day 40 and the level remained stable with increasing time (Additional file 18: Figure S15E). Conversely, the active CD4 T-helper lymphocyte count exhibited a higher level of 8500, 9500 and 7500 cells/mm^3^ with the Bm86, both AQP1 & AQP2 and VgR individual vaccine candidates, respectively (Additional file 19: Figure S16A–D); with the cocktail vaccine candidate, the active CD4 T-helper lymphocyte count declined slightly to 5900 cells/mm^3^ (Additional file 19: Figure S16E). Simultaneously, the highest memory CD4 T-helper lymphocyte count was observed on day 50 of the immunization for both the individual and cocktail vaccines (Additional file 20: Figure S17A–E); a corresponding increase concurrently appeared for the peaks of the active B lymphocytes population, reaching 700 cells/mm^3^ and 650 cells/mm^3^, which decreased gradually with the immunization schedule in the case of the immunization with *R. microplus* Bm86, AQP1, AQP2 and VgR individual vaccine candidates, respectively. Although the level of the active B-lymphocyte population declined to 550 cells/mm^3^ for cocktail vaccine candidates, it kept a consistent and continuing level beginning from day 80 (Additional file 21: Figure S18A–E). The same profile was noted with the level of memory B lymphocytes for both individual and cocktail vaccines (Additional file 22: Figure S19A–E).

Consequently, boosted kinetics of the humoral immune responses, following the second booster dose of immunization were remarkable. A noticeable antibody titer of the IgG1 and IgM reached its highest level on day 50 in all individual and cocktail proteins immune simulations. The highest titer reached 140,000 arbitrary units when using the *R. microplus* Bm86, AQP1, AQP2 and VgR individual proteins as a vaccine candidate, which is comparable to the 550,000 arbitrary units reached upon using the cocktail vaccine (Additional file 14: Figure S11).

A prominent elevated level of 450,000 ng/ml of interferon (IFN)-gamma and of 450,000–800,000 ng/ml) of interleukin ( IL)-2 with a pronounced decline in the level of IFN-gamma to 100,000 ng/ml was observed before each booster dose combined with a very low Simpson index (D) from the beginning of the vaccination schedule with the *R. microplus* Bm86, AQP1, AQP2 and VgR individual proteins as a vaccine candidate. A comparable marked consistent and continuing level of 2.4 × 10^6^ ng/ml of IFN-gamma and 1.9 × 10^6^ ng/ml of IL-2 was predicted upon simulation of the immune profile against the cocktail proteins as a vaccine candidate, from the beginning of the prime dose injection up to day 70 (Additional file 15: Figure S12). However, a low Simpson index (D), indicating high immune diversity, was observed after the second booster dose of the vaccination schedule upon using the cocktail proteins as a vaccine candidate (Additional file 15: Figure S12).

## Discussion

The current analysis of the targeted *R. microplus* proteins Bm86, AQP1, AQP2, and VgR provides an evidence-based evaluation for their potential as anti-tick vaccine candidates. The aim of using the cocktail vaccine is to target multiple locations such as the salivary glands, gut, and ovaries of the tick as well as different developmental stages, like larvae, nymph, and adult, during the tick life cycle. Hitting multiple physiological processes simultaneously in different tick stages could enhance the vaccine efficacy. The current protein candidates were chosen as they are differentially expressed in terms of time and location inside the tick body. For example, Bm86 is usually expressed in the midgut of all tick stages (larva, nymph, and adult) because it is essential in the blood feeding process, and it is highly expressed in partially engorged female ticks [7, 8]. VgR protein is only expressed in the ovary of mated females, while it is not expressed in other female tissues, as well as male ticks or the other developmental stages. It indicates the expression specificity for sex or tick tissue, consequently, with a restricted vaccine effectiveness if used as a single subunit vaccine [4]. Regarding the AQP1 and AQP2 expression profile, it was reported that AQP1 is highly expressed in the synganglia and gut of adult ticks, while AQP2 is expressed in the salivary glands of the partially engorged females [10, 11]. The previous literatures support in choosing the current candidates to fulfill the aim of the present work. The high aliphatic index and favorable (which is less than 40) instability indices of AQP1 and AQP2 (29.4 and 22.23, respectively), along with the acceptable instability indices for Bm86 and VgR (44.50 and 43.55, respectively) [22], suggest the suitability of these proteins in a vaccine cocktail. These characteristics indicate that these proteins have greater structural stability at high temperatures, making them valuable for animal vaccination trials and future vaccine commercialization. Additionally, the negative GRAVY values of the *R. microplus* Bm86 and VgR proteins (-0.579 and -0.336, respectively) indicate their hydrophilicity, allowing for better binding and solubility with surrounding water molecules. Considering the hydrophilic proteins in vaccine formula could enhance the antigen release and avoid depot retention, and achieve appropriate immune cell interaction and uptake upon vaccination. The predicted chemical composition of these proteins’ sheds light on their biological functions and their importance for the survival of tick generations. For the Bm86 protein, the highest amino acid composition is cysteine ©) at 10.3%, which highlights its specific adaptation to its anatomical location within tick tissue, particularly as a gut membrane protein that encounters substantial blood meals during feeding [18, 36]. Thus, cysteine plays a crucial role in redox activity and has a high affinity for metals, aiding in the detoxification of elevated iron levels ingested from large blood meals [20, 38–40]. In contrast, AQP1 has the highest amino acid composition of isoleucine (I) at 10.4%, while AQP2 shows alanine (A) and leucine (L) at 13%. This predicted data suggests that both AQP1 and AQP2 are transmembrane proteins. Their non-polar, hydrophobic aliphatic residues are essential for maintaining the stability of these membrane proteins, which are crucial for their biological functions related to water balance and solute transport [10, 11, 39]. The most prevalent amino acid composition in VgR is serine (S) at 9%, which may contribute to the protein's stability and its role in regulating the signaling pathway that facilitates vitellogenin transfer for oocyte maturation [12, 42]. Overall, these physicochemical properties suggest effective antigen presentation and potential to elicit promising protective immune responses [14, 43–45]. Topographical protein analysis plays a crucial role in identifying better epitopes that are essential for vaccine development, ultimately leading to more effective immune responses. The full-length extracellular localization of the Bm86 protein, along with the long amino acid sequence of the VgR, which contains only one transmembrane (TM) helix, suggests that these proteins have excellent immunogenicity. Although AQP1 and AQP2 have six TM helices, their extracellular regions may contribute significantly to enhancing the protective effectiveness of the vaccine.

The understanding of protein behavior within the cell is greatly informed by information about signal peptides and post-translational modifications [44, 46]. Both Bm86 and VgR possess numerous glycosylation and phosphorylation sites. The existence of these putative glycosylated and phosphorylated residues supports their biological functions and indicates that they are extracellular proteins. Bm86 may play a role in cell growth and blood coagulation, while VgR is essential for oocyte maturation through the initiation of receptor-mediated endocytosis [12, 47]. Collectively, these proteins are crucial for cellular regulation, signaling, and the immune response [48, 49]. In addition, the phosphorylated and glycosylated epitopes enhance antigen accessibility, the MHC binding and stability, providing appropriate immune recognition, T-cell phenotype switching, and cytokine production resulting in specific immune responses [48–50]. Thus, the combination of the four proteins, *R. microplus* Bm86, VgR, AQP1, and AQP2, each with diverse protein structure and post-translational modification behaviors, could enhance vaccine efficacy and protection [6, 17, 44, 48]. Blast-P predictions for all proteins studied show acceptable similarity percentages compared to the *Bos taurus* protein sequences (their orthologs): 21.08% for Bm86, 38.04% for AQP1, 35.34% for AQP2, and 33.35% for VgR. These percentages indicate a reduced likelihood of developing autoimmune reactions following vaccination with these proteins [2, 52]. The highest identity matrix percentages with *R. sanguineous* suggest the potential of these vaccine candidates to provide cross-species protection, which broadens the vaccine effectiveness and improves host immunity against multiple tick species, and lowers the financial demands [9, 53]. Furthermore, all tested proteins, *R. microplus* Bm86, VgR, AQP1, and AQP2, have demonstrated good scores for antigenicity, which compares on the site to the Bm68 recombinant protein (the only commercialized anti-tick vaccine), supporting its predicted scores as future promising candidates. Additionally, non-allergenicity and non-toxicity, suggesting they could elicit a safe and effective protective immune response [44, 52]. Regarding toxicity predictions for proteins and/or peptides, the Toxipred2 tool, which is trained on extensive data using BLAST-based similarity, motif emergence, and class-identification-based motif searches, offers reliable predictive models. Therefore, similarity and motif-based techniques are expected to yield high probabilities of accurate predictions. It is recommended to select peptide candidates from the *R. microplus* VgR protein to minimize the risk of toxicity that could result from toxic motifs or peptides present within the entire protein sequence [52]. The secondary structure of proteins plays a crucial role in determining their stability and accessibility. In the case of *R. microplus*, the Bm86 and VgR proteins exhibit a significant amount of beta-strand structure. In contrast, the AQP1 and AQP2 proteins of *R. microplus* lack beta strands, with the predominant secondary structure being alpha helices and coils. Both types of structures possess a substantial number of hydrogen bonds that help maintain protein stability and conformation. This stability facilitates better interactions between their epitopes and antibodies, contributing to the immunogenic response [20, 44, 52].

The predicted number of B-cell epitopes for the *R. microplus* proteins, Bm86, AQP1, AQP2, and VgR, was 15, 5, 6, and 24 peptides, respectively, as determined using the IEDB B-cell epitope web server. B-cell receptors can recognize the solvent-exposed regions of the protein sequences, which trigger the production of immunoglobulins. The residue scores for Bm86 and VgR were 0.529 and 0.520, respectively, while the scores for AQP1 and AQP2 were lower, at 0.429 and 0.438, respectively. This difference may be due to AQP1 and AQP2 being transmembrane proteins, each containing six transmembrane (TM) domains; however, some portions of their protein sequences are accessible to B-cell receptors [54]. Moreover, all protein sequences (Bm86, AQP1, AQP2, and VgR) exhibited a good epitope density, ranging from 0.95 to 0.99. This density correlates with the number of predicted epitopes based on the protein sequence lengths and their ability to bind various combinations of MHC II alleles (BoLA-DRB3_00101, BoLA-DRB3_01101, BoLA-DRB3_01201 and BoLA-DRB3_02002). An appropriate epitope density could enhance CD4 T-helper immune response, resulting in proper affinity maturation and eliciting a good level of antibody isotypes. However, in case of exceeding the epitope density beyond an appropriate level, immune tolerance might be developed [55, 59]. A total of 2985 shared epitopes could potentially be recognized by the same MHC allele combinations. However, no conserved shared epitopes were found among the predicted epitopes from the four protein sequences. This suggests a variable immunogenic response with little or no antigenic competition among the epitopes in the vaccine cocktail, while still providing a wide range of epitopes that could improve immune responses upon vaccination [9, 55, 56]. Additionally, immune system simulations of the individual proteins and the cocktail of *R. microplus* proteins as vaccine candidates demonstrated robust humoral and cell-mediated immune responses. In the case of the tick vaccine, T helper cell type 2 (Th2) is usually the molecule in cross-talk with the injected proteins, inducing cytokines such as IL4, -5 and -13, which will be responsible for regulating the humoral immune response and consequently the antibodies elicited against tick infestation. The immune profiles indicated excellent antigen presentation, with high levels of cytotoxic T-cells, helper T-cells, memory B-cells, plasma B-cells and different antibody isotypes, including IgM, total IgG and IgG1, both individually and in cocktail form. These findings suggest exceptional antigen presentation and robust antibody-secreting cells, leading to a high level of immunoglobulins [56–58]. The higher antibody titer predicted with the cocktail vaccine, compared to each individual vaccine candidate, may be due to the synergistic effects of the *R. microplus* proteins Bm86, AQP1, AQP2 and VgR working together. This observation was further supported by the predicted T-cell epitopes, which also revealed no conserved shared epitopes among the four protein sequences, indicating a synergistic response that enhances antibody isotype levels [51]. Therefore, the synergistic effect of exposed and concealed poly-antigenic-based anti-tick vaccine, which is targeting multiple physiological processes, with no antigenic competition, could broaden the vaccine efficacy [55, 58]. Furthermore, the consistently high level of IFN-gamma (2.4 × 10^6^ ng/ml) observed during the simulation of the immune profile against the cocktail proteins as a vaccine candidate is noteworthy. This result may be attributed to the same synergistic effect of the protein combination used in the cocktail. The elevated levels of IFN-gamma could promote macrophage activation and help balance Th1 and Th2 responses, thereby boosting the overall immune response [56]. A fluctuating high level of IFN-gamma and IL2 after each booster dose enhances the Th1 immune response (cell-mediated immunity), which could provoke the Th2 response (humoral immunity), thereby supporting memory B-cell formation for useful long-term humoral immunity [56–58]. Additionally, the limited Simpson index (D) indicated a restricted danger signal value regarding the discrimination between self and non-self-antigen after the third dose of vaccination, which means a high level of biodiversity among the vaccine candidates and the host, suggesting a diverse and effective immune response from the cocktail vaccine [18]. Thus, both the consistently high levels of IFN-gamma and the elevated antibody isotypes could contribute to improving vaccine efficacy, aiding in the control of tick fitness and the survivability of future generations.

## Conclusions

The bioinformatic and immunoinformatic tools provide a robust pipeline for predicting and evaluating potential peptide cocktail vaccine candidates against *R. microplus* infestations. Based on the physicochemical properties, topographical analysis, antigenicity, and toxicity predictions, *R. microplus* proteins Bm86, AQP1, AQP2, and VgR appear to be promising candidates for a cocktail protein vaccine. The need for computational approaches for vaccine candidates’ validation could save financial costs and animal lives. However, in vitro and in vivo experiments are crucial steps for protein efficacy validation. Simulating the immune response to the cocktail proteins as vaccine candidates demonstrates the synergistic effect of combining these proteins, which can enhance the overall immune response. Given the protein cocktail has a greater synergistic effect than individual proteins, we recommend designing strong MHC binding affinity epitopes which is conserved and cross-reactive with multiple species from each protein to enhance and broaden the vaccine efficacy. This approach aims to maximize the benefits and improve the effectiveness of the future anti-*R. microplus* peptide cocktail vaccine.

## Supplementary Information

Additional file 1: Figure S1. Transmembrane helices prediction of vaccine target *R. microplus* proteins (Bm86, AQP1, AQP2, and VgR).

Additional file 2: Figure S2. Signal Peptide prediction of vaccine target *R. microplus* proteins (Bm86, AQP1, AQP2, and VgR).

Additional file 3: Figure S3. GPI Anchor prediction of vaccine target* R. microplus* proteins (Bm86, AQP1, AQP2, and VgR).

Additional file 4: Figure S4. Secondary structure and solvent accessibility prediction of vaccine target *R. microplus* proteins (Bm86, AQP1, AQP2, and VgR). Surface Accessibility: Red is exposed, and blue is buried, threshold at 25%. Secondary Structure: Helix, Strand, Coil. Disorder: Thickness of gray line equals probability of disordered residue.

Additional file 5: Figure S5. N-glycosylation sites prediction of vaccine target *R. microplus* proteins (Bm86, AQP1, AQP2, and VgR).

Additional file 6: Figure S6. Phosphorylation sites prediction of vaccine target *R. microplus *proteins (Bm86, AQP1, AQP2, and VgR).

Additional file 7: Figure S7. Sequence identity matrix heatmaps for tick species and Bos taurus of vaccine target *R. microplus* proteins (Bm86, AQP1, AQP2, and VgR).

Additional file  8:  Dataset S1. Protein sequence alignment of *R. microplus *proteins (Bm86, AQP1, AQP2, and VgR) with *Bos taurus*  and tick species. 

Additional file 9: Figure S8. B- cell epitopes prediction of vaccine target *R. microplus *proteins (Bm86, AQP1, AQP2, and VgR).

Additional file  10: Tables S1-5. B-cell and CD4 T-cell epitopes predicted peptides of *R. microplus* proteins (Bm86, AQP1, AQP2, and VgR).

Additional file  11: Dataset S2. T-cell epitopes peptides of *R. microplus* proteins (Bm86, AQP1, AQP2, and VgR) predicted by NetMHCIIpan Version 4.3d.

Additional file  12: Figure S9. Heatmap (A) and a cumulative histogram (B) showed the number of distributed shared epitopes of vaccine target *R. microplus* proteins (Bm86, AQP1, AQP2, and VgR) that bind to different MHC II BoLA-DRB3s alleles.

Additional file  13: Figure S10. Heatmap and a bar plot represented the epitope densities and the number of epitopes (n) predicted in correlation to their protein combination of bovine sequences length to bind MHC II alleles.

Additional file  14: Figure S11. C-ImmSim prediction represented the immune profile of isotypes (IgM, IgG1 and IgG2) levels for the individual *R. microplus* Bm86 (A), AQP1 (B), AQP2 (C), and VgR (D) and cocktail proteins (E) as vaccine candidates.

Additional file  15: Figure S12. C-ImmSim prediction represented the immune profile of IFN-gamma, and IL-2 levels for the individual *R. microplus* Bm86 (A), AQP1 (B), AQP2 (C), and VgR (D) and cocktail proteins(E) as vaccine candidates.

Additional file  16: Figure S13. C-ImmSim prediction represented the immune profile of macrophages (MA) population state levels for the individual *R. microplus* Bm86 (A), AQP1 (B), AQP2 (C), and VgR (D) and cocktail proteins(E) as vaccine candidates.

Additional file  17: Figure S14. C-ImmSim prediction represented the immune profile of dendritic cell (DC) population state levels for the individual *R. microplus *Bm86 (A), AQP1 (B), AQP2 (C), and VgR (D) and cocktail proteins(E) as vaccine candidates.

Additional file  18: Figure S15. C-ImmSim prediction represented the immune profile of T-lymphocytes (TC) population state levels for the individual *R. microplus* Bm86 (A), AQP1 (B), AQP2 (C), and VgR (D) and cocktail proteins(E) as vaccine candidates.

Additional file  19: Figure S16. C-ImmSim prediction represented the immune profile of T- helper (TH) cell population state levels for the individual *R. microplus* Bm86 (A), AQP1 (B), AQP2 (C), and VgR (D) and cocktail proteins(E) as vaccine candidates.

Additional file  20: Figure S17. C-ImmSim prediction represented the immune profile of T- helper (TH) cell population levels for the individual *R. microplus* Bm86 (A), AQP1 (B), AQP2 (C), and VgR (D) and cocktail proteins(E) as vaccine candidates.

Additional file  21: Figure S18. C-ImmSim prediction represented the immune profile of B-cell population state levels for the individual *R. microplus* Bm86 (A), AQP1 (B), AQP2 (C), and VgR (D) and cocktail proteins(E) as vaccine candidates.

Additional file  22: Figure S19. C-ImmSim prediction represented the immune profile of B-cell population levels for the individual *R. microplus* Bm86 (A), AQP1 (B), AQP2 (C), and VgR (D) and cocktail proteins(E) as vaccine candidates.

## Data Availability

The data presented in this study are included in the manuscript and supplementary materials. The datasets generated and/or analyzed during the current study are available in the GenBank under the following accession numbers: PQ030824 for Bm86, PQ030825 for AQP1, PQ030826 for AQP2 and PQ030827 for VgR.
