# Supplementary figures and images for: Comparative immunoinformatic analysis of Rhipicephalus microplus cocktail vaccine targets
Source: Parasit Vectors. 2025 Dec 9;18:502. doi: 10.1186/s13071-025-07109-y (PMC12690872; doi:10.1186/s13071-025-07109-y)

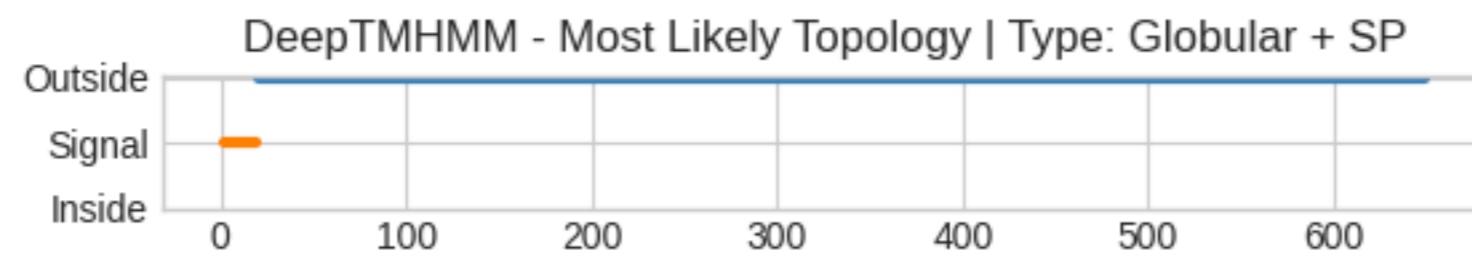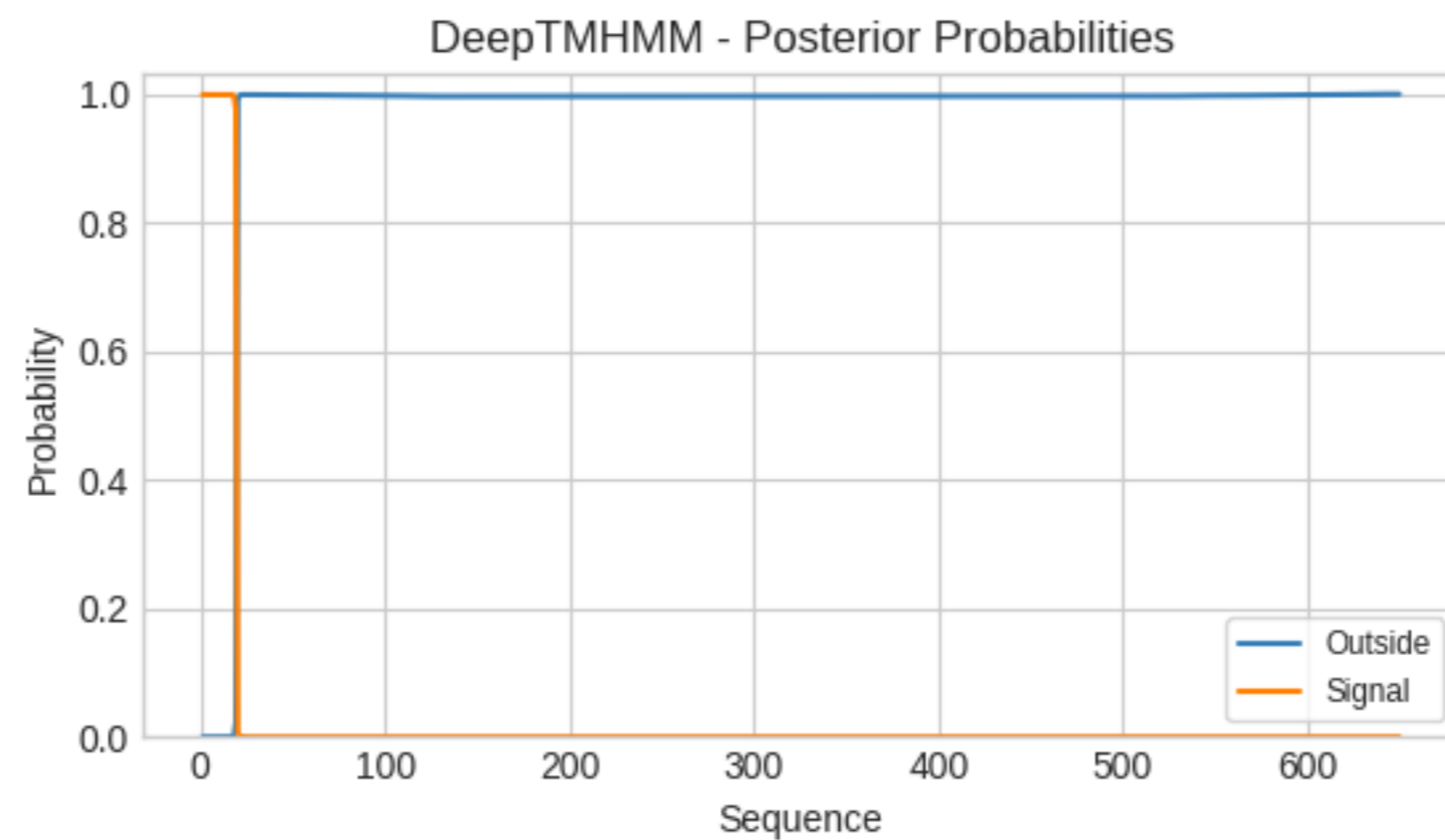

**Bm86**

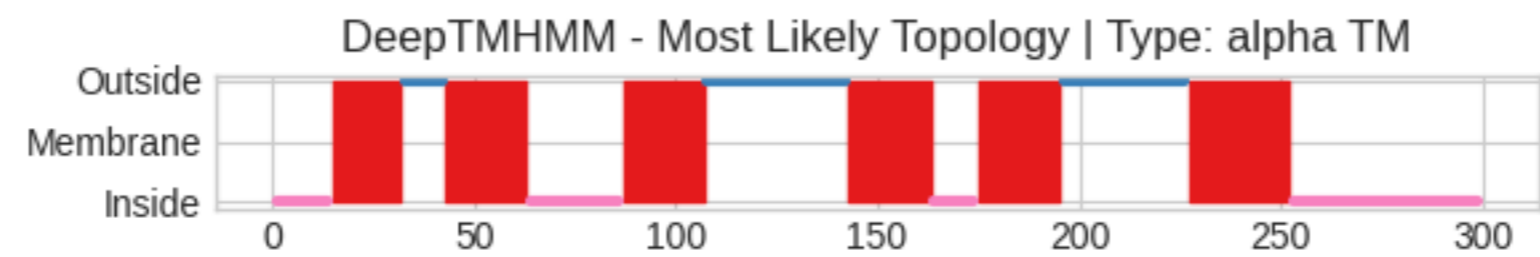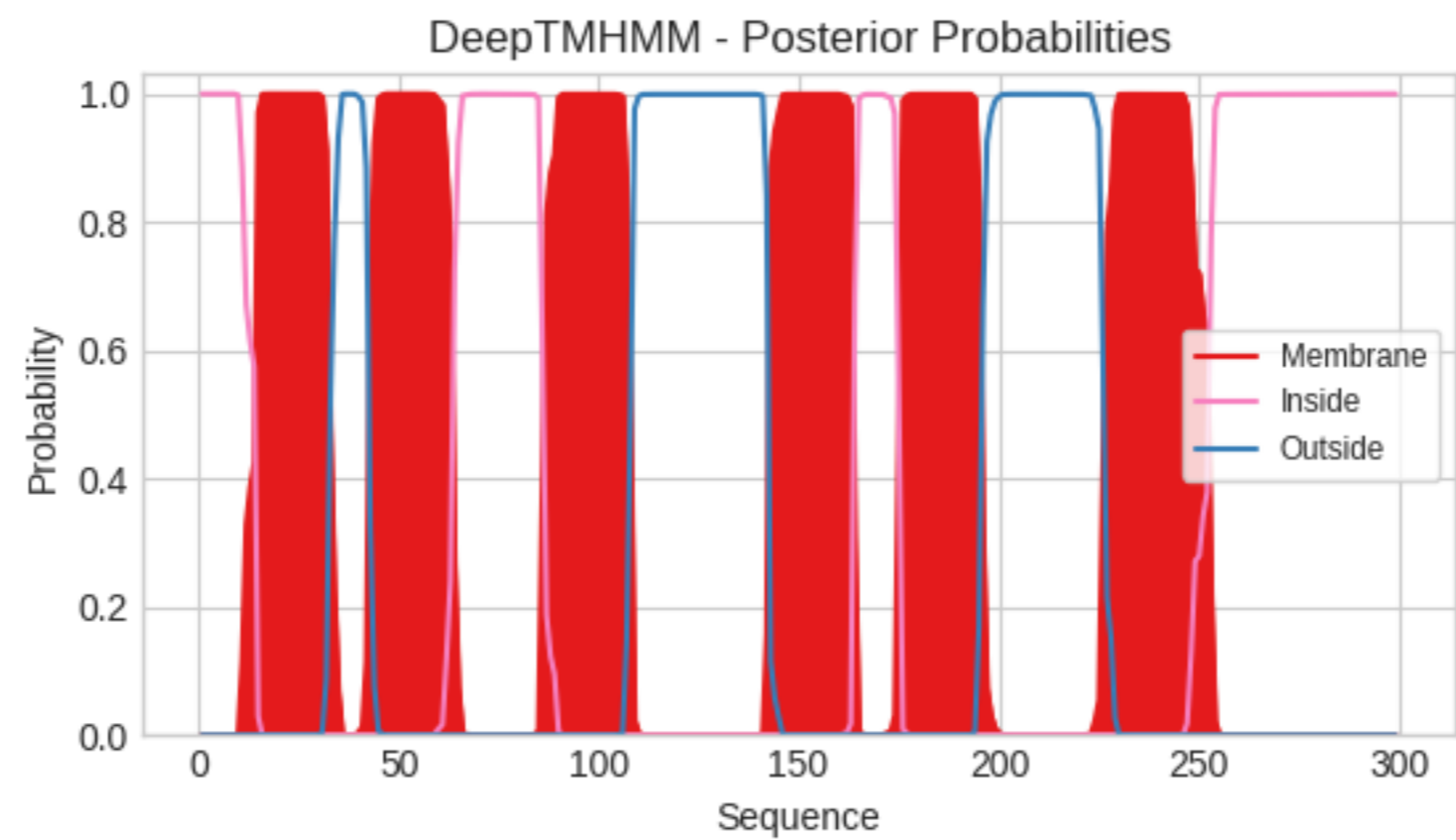

AQP1

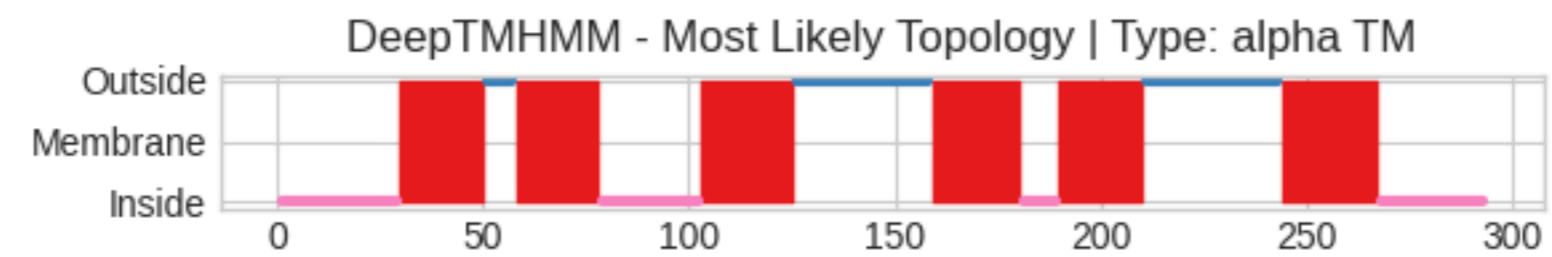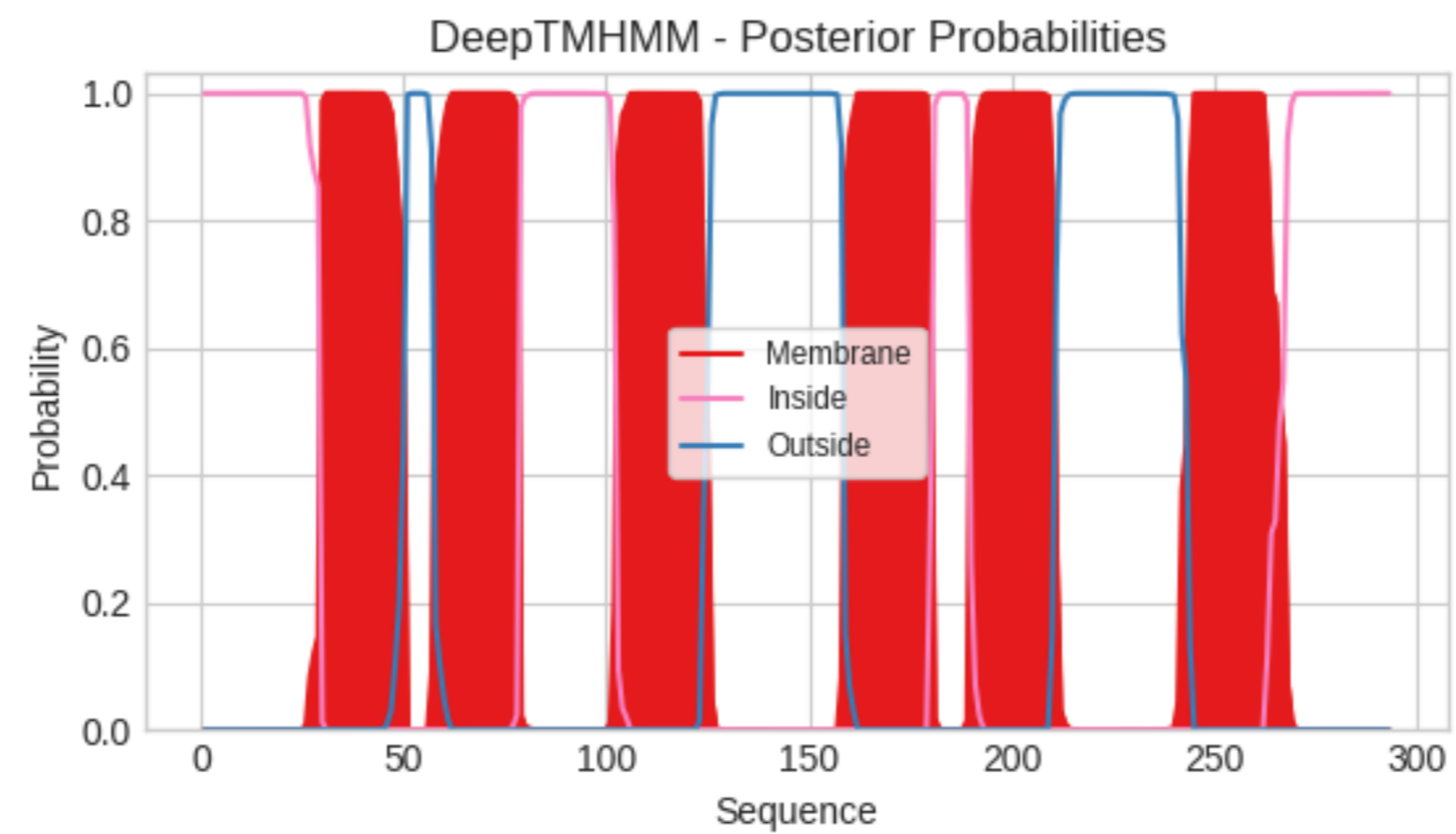

AQP2

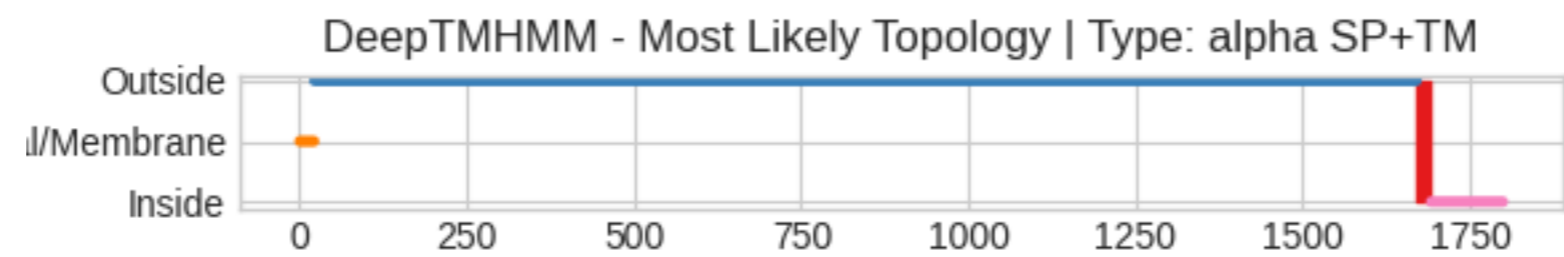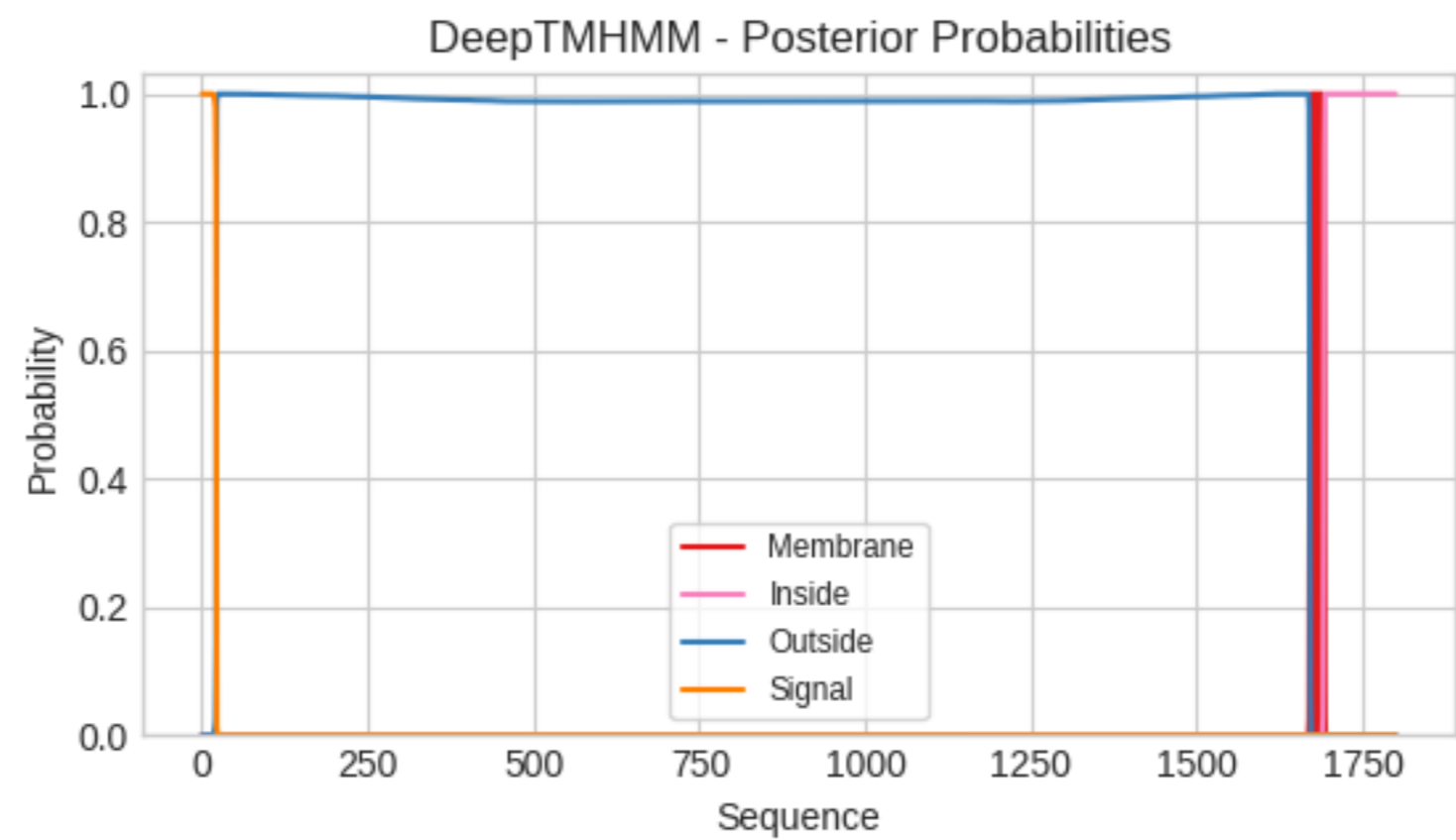

**VgR**

Supplement: Supplementary file 1 — Additional file 1: Figure S1. Transmembrane helices prediction of vaccine target R. microplus proteins (Bm86, AQP1, AQP2, and VgR). [file 13071_2025_7109_MOESM1_ESM.pdf]

**AQP1\_299\_bp**

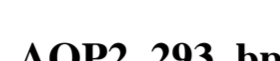

Supplement: Supplementary file 4 — Additional file 4: Figure S4. Secondary structure and solvent accessibility prediction of vaccine target R. microplus proteins (Bm86, AQP1, AQP2, and VgR). Surface Accessibility: Red is exposed, and blue is buried, threshold at 25%. Secondary Structure: Helix, Strand, Coil. Disorder: Thickness of gray line equals probability of disordered residue. [file 13071_2025_7109_MOESM4_ESM.pdf]

# Bm86

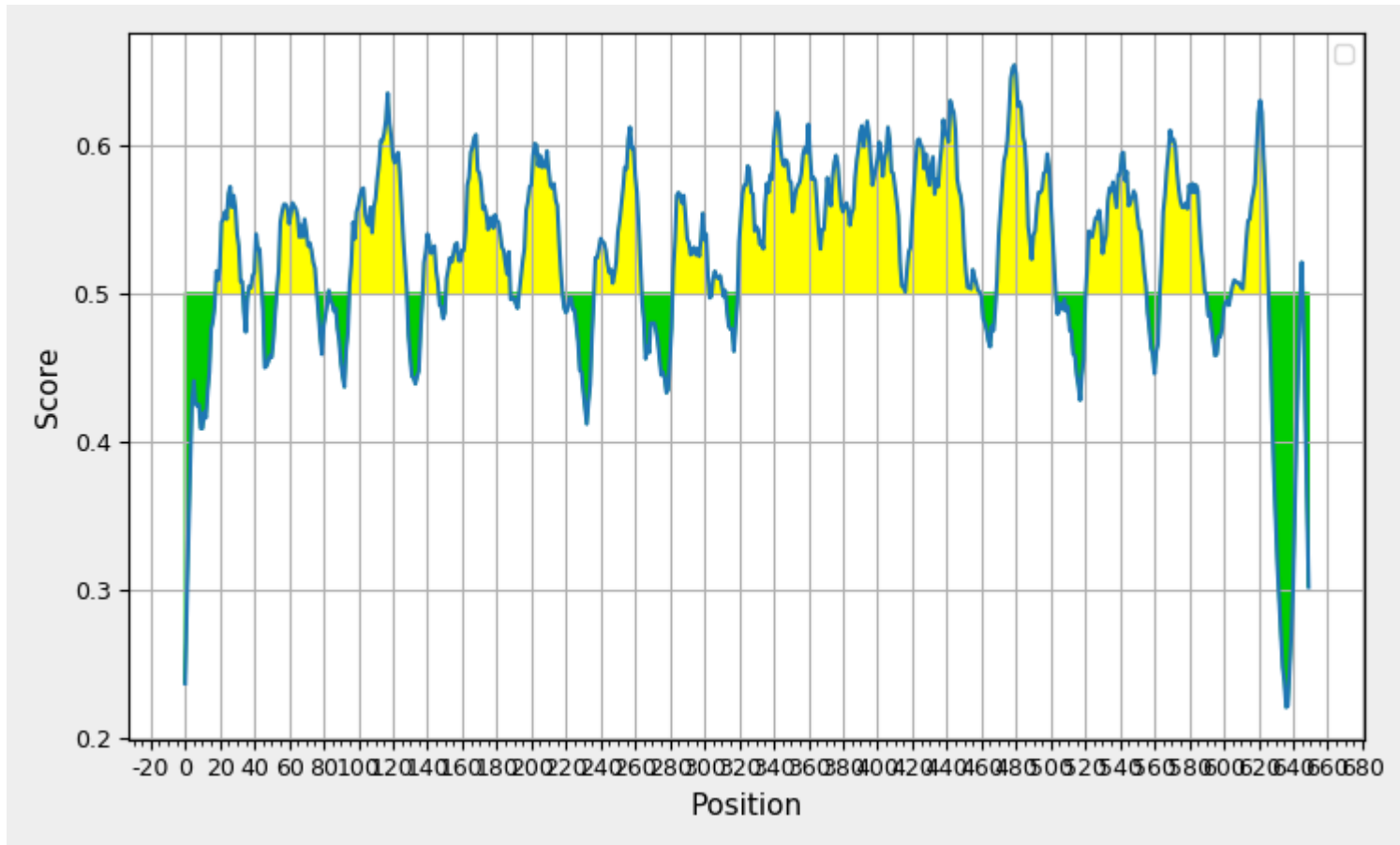

# AQP1

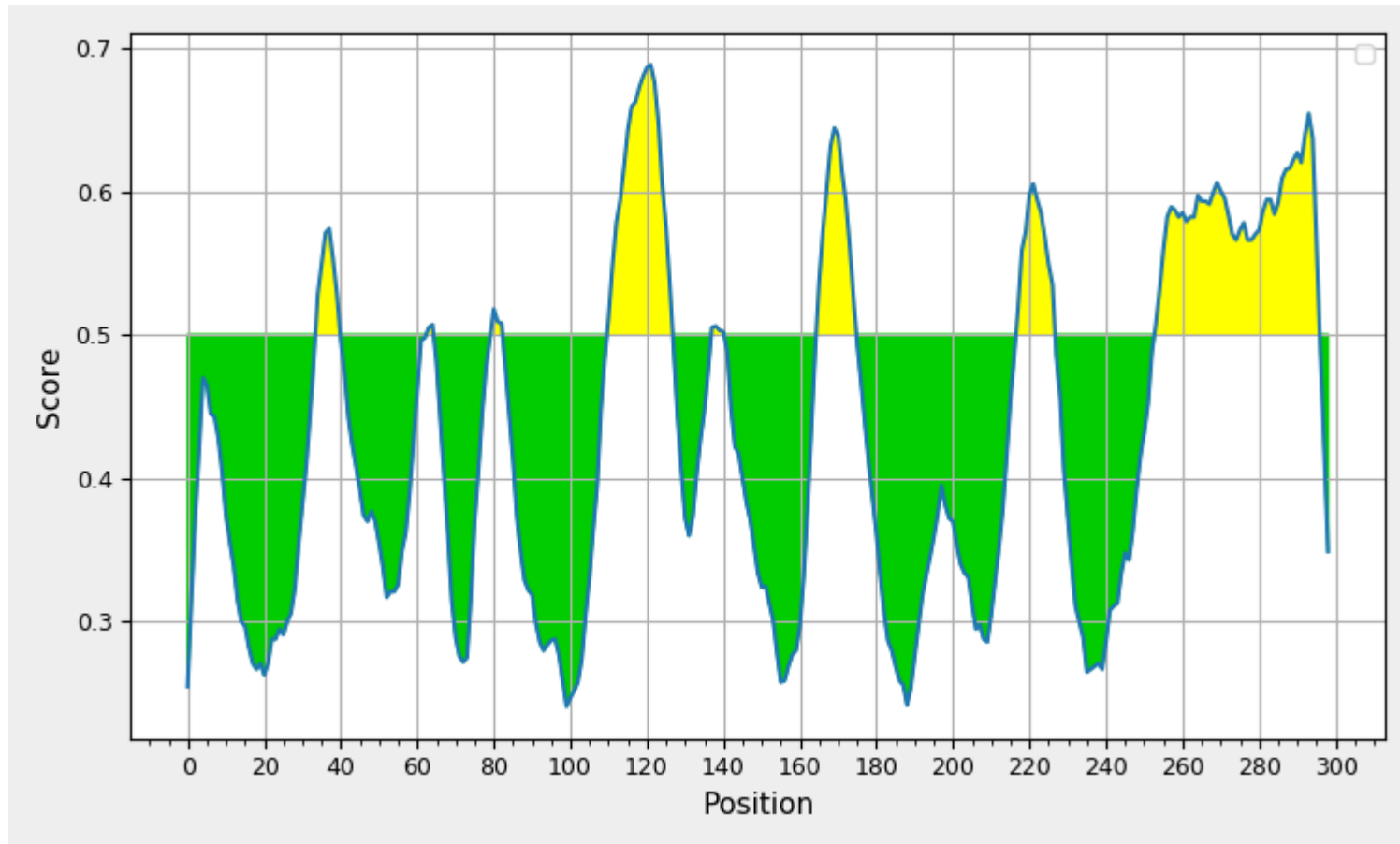

# AQP2

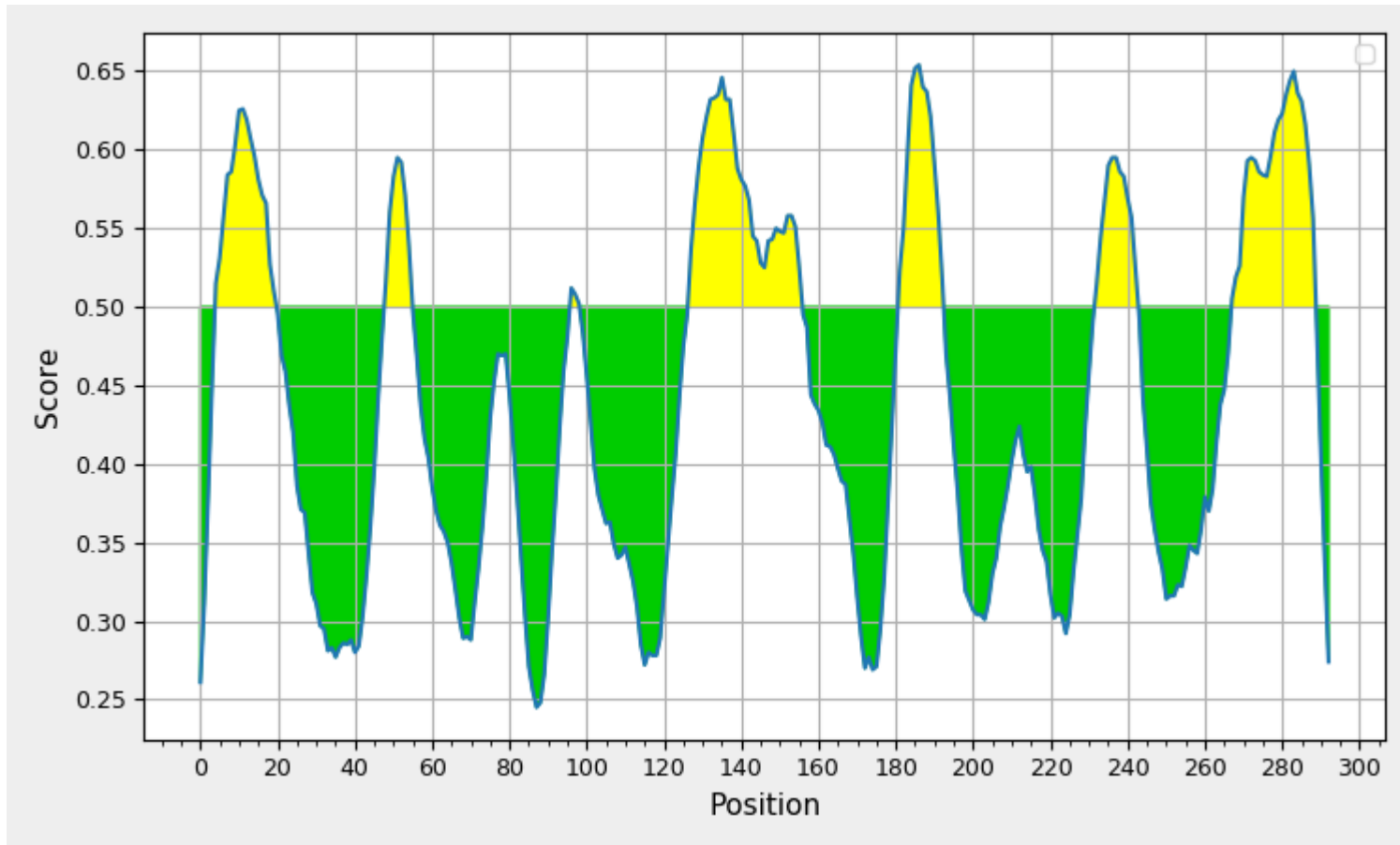

# VgR

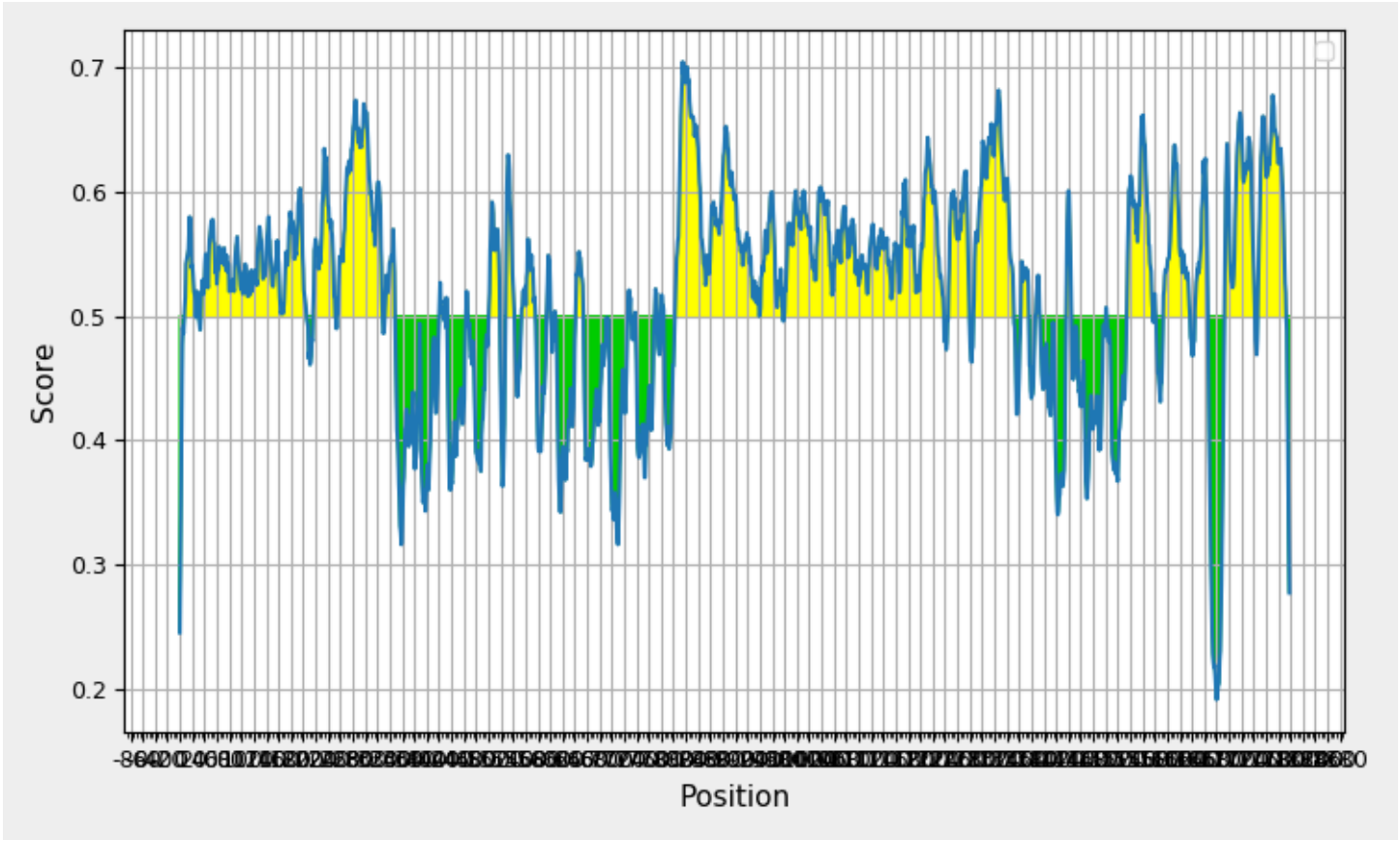

Supplement: Supplementary file 9 — Additional file 9: Figure S8. B- cell epitopes prediction of vaccine target R. microplus proteins (Bm86, AQP1, AQP2, and VgR). [file 13071_2025_7109_MOESM9_ESM.pdf]

BoLA-DRB3\_00101

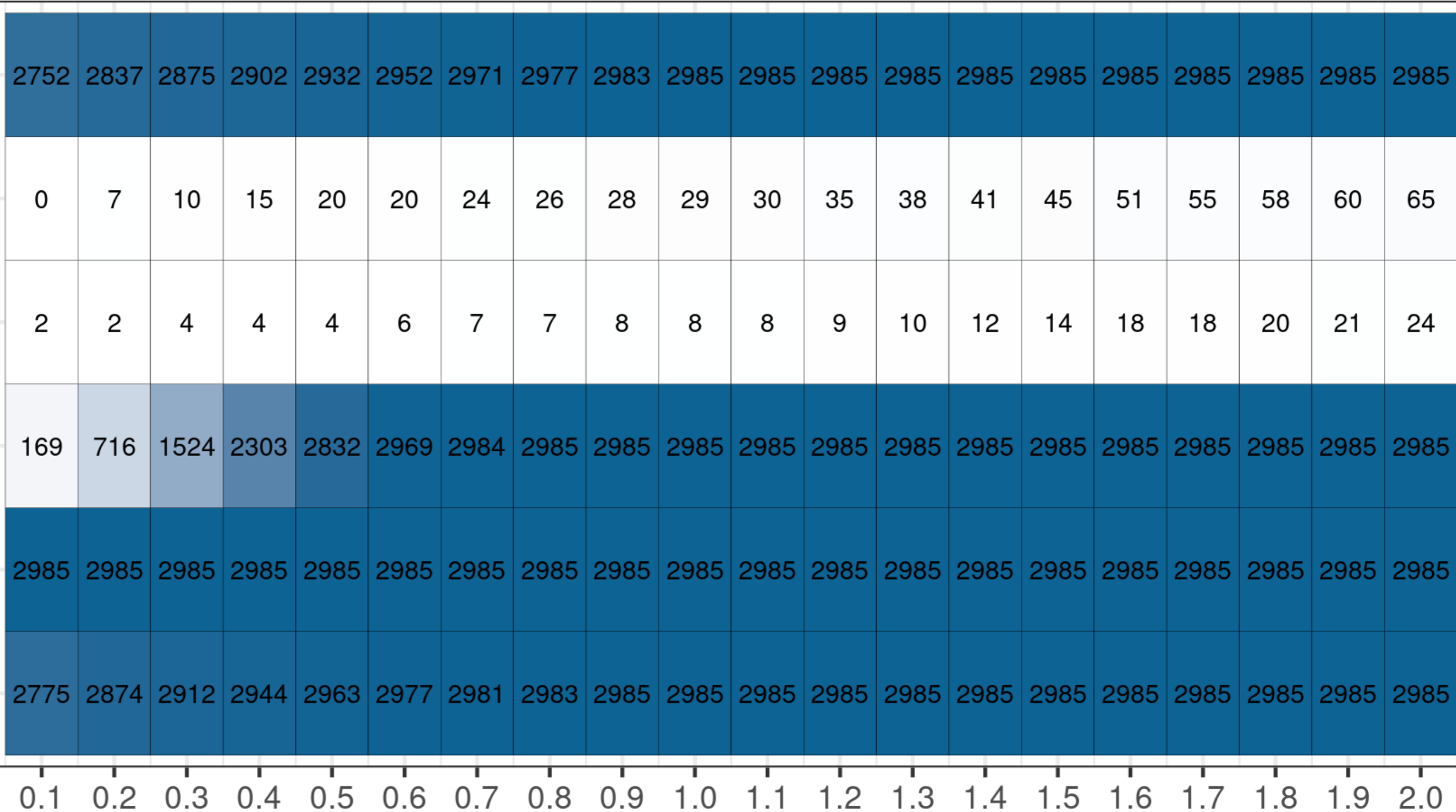

**B**

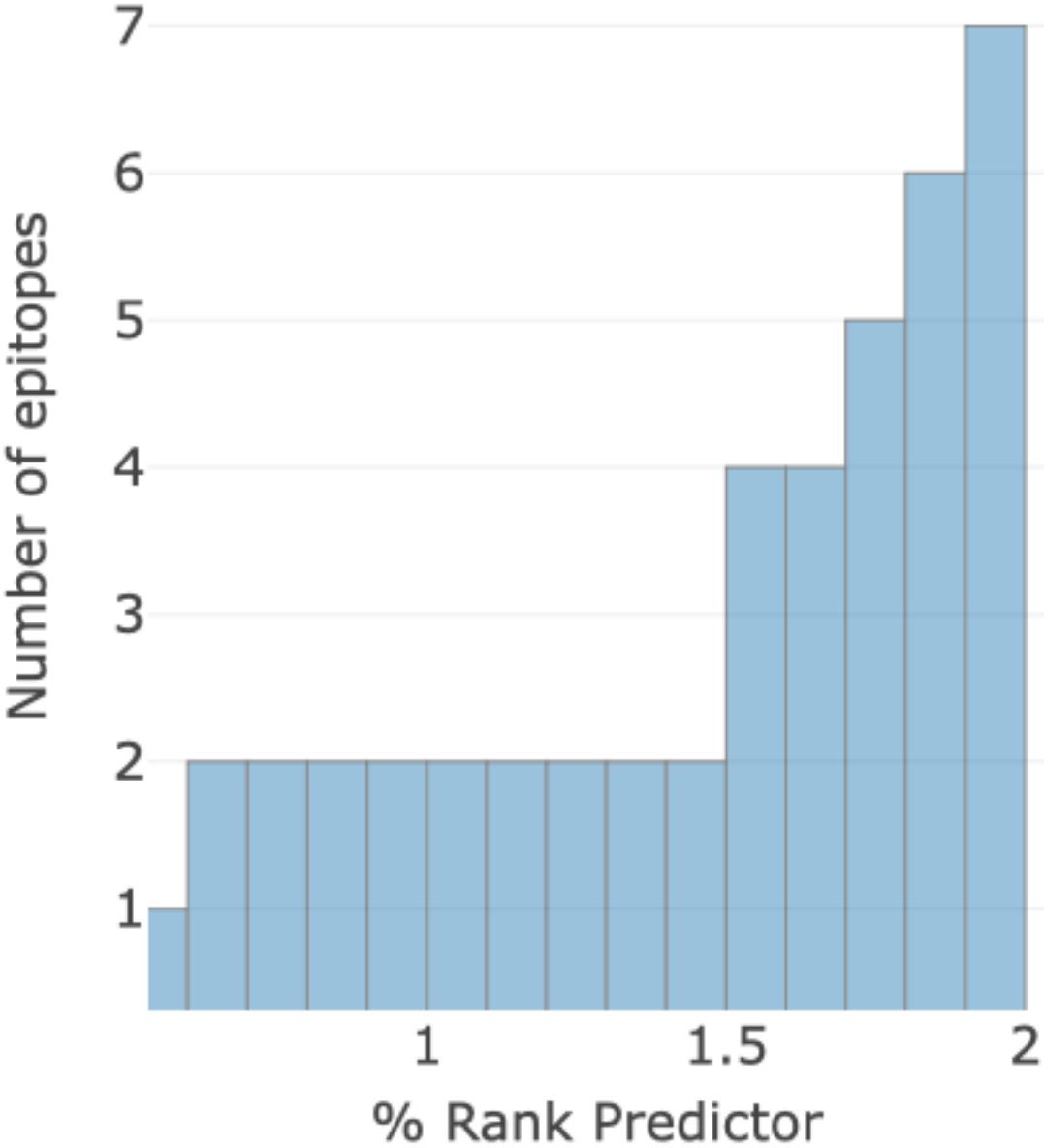

Supplement: Supplementary file 12 — Additional file 12: Figure S9. Heatmap (A) and a cumulative histogram (B) showed the number of distributed shared epitopes of vaccine target R. microplus proteins (Bm86, AQP1, AQP2, and VgR) that bind to different MHC II BoLA-DRB3s alleles. [file 13071_2025_7109_MOESM12_ESM.pdf]

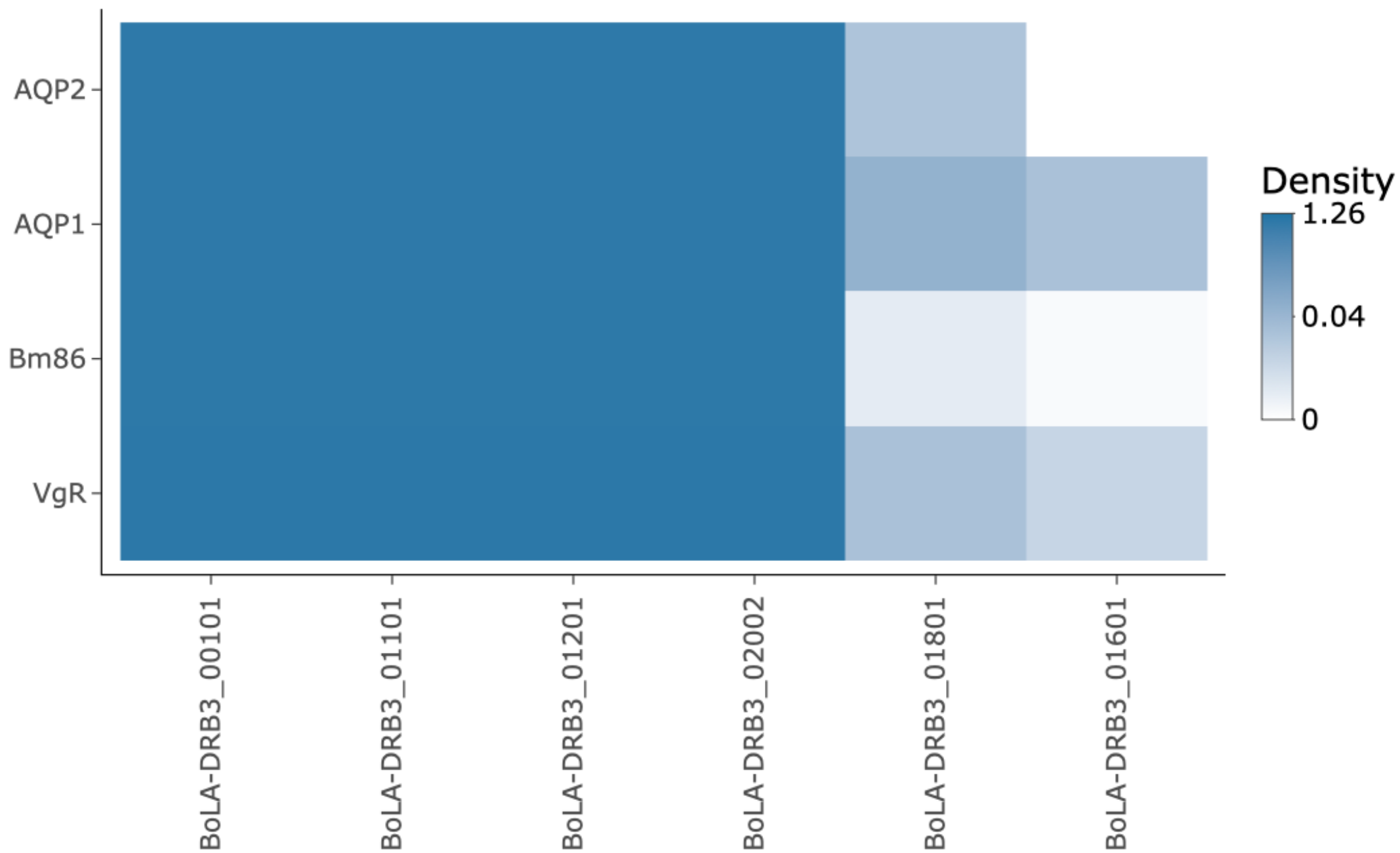

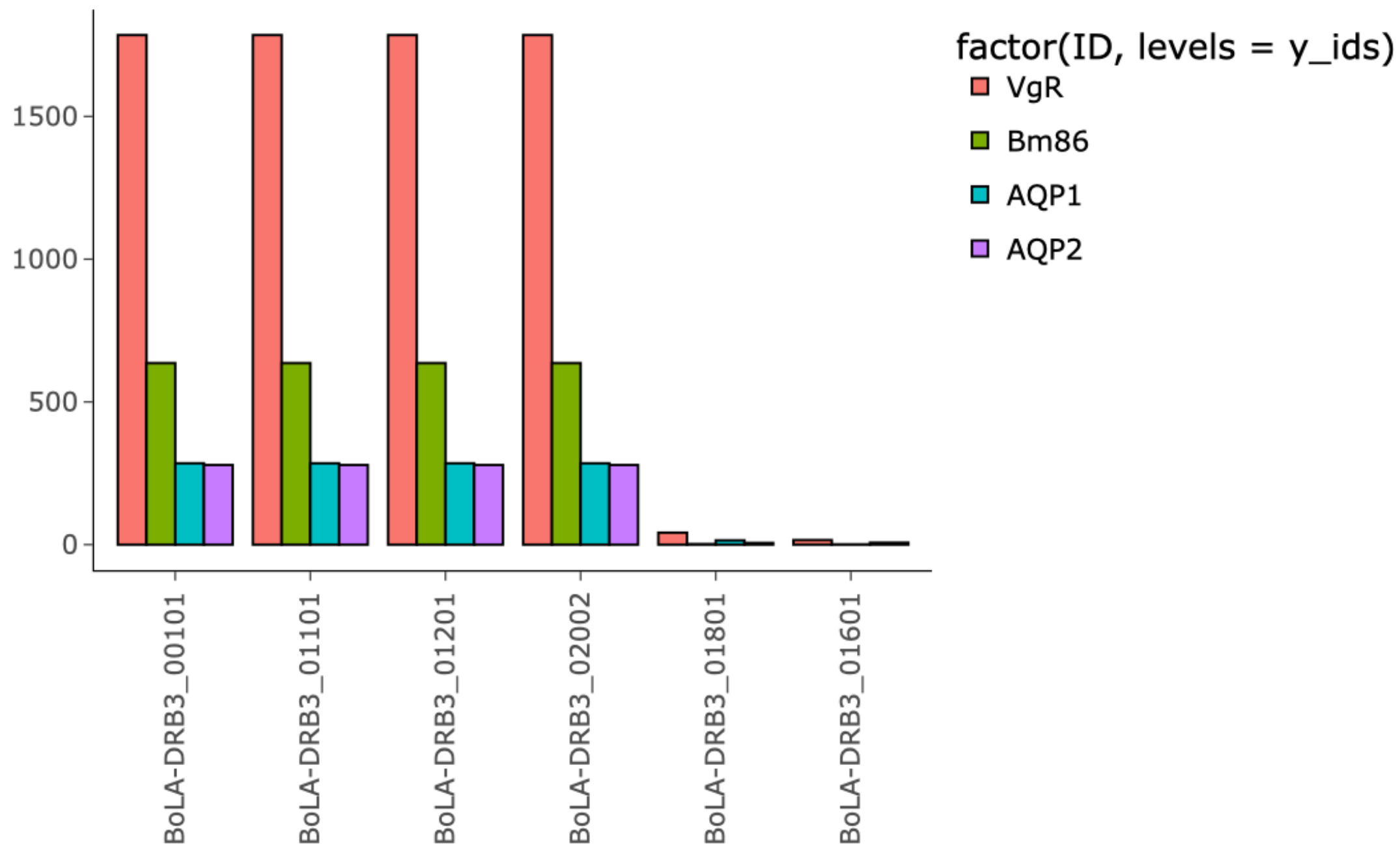

Supplement: Supplementary file 13 — Additional file 13: Figure S10. Heatmap and a bar plot represented the epitope densities and the number of epitopes (n) predicted in correlation to their protein combination of bovine sequences length to bind MHC II alleles. [file 13071_2025_7109_MOESM13_ESM.pdf]

A

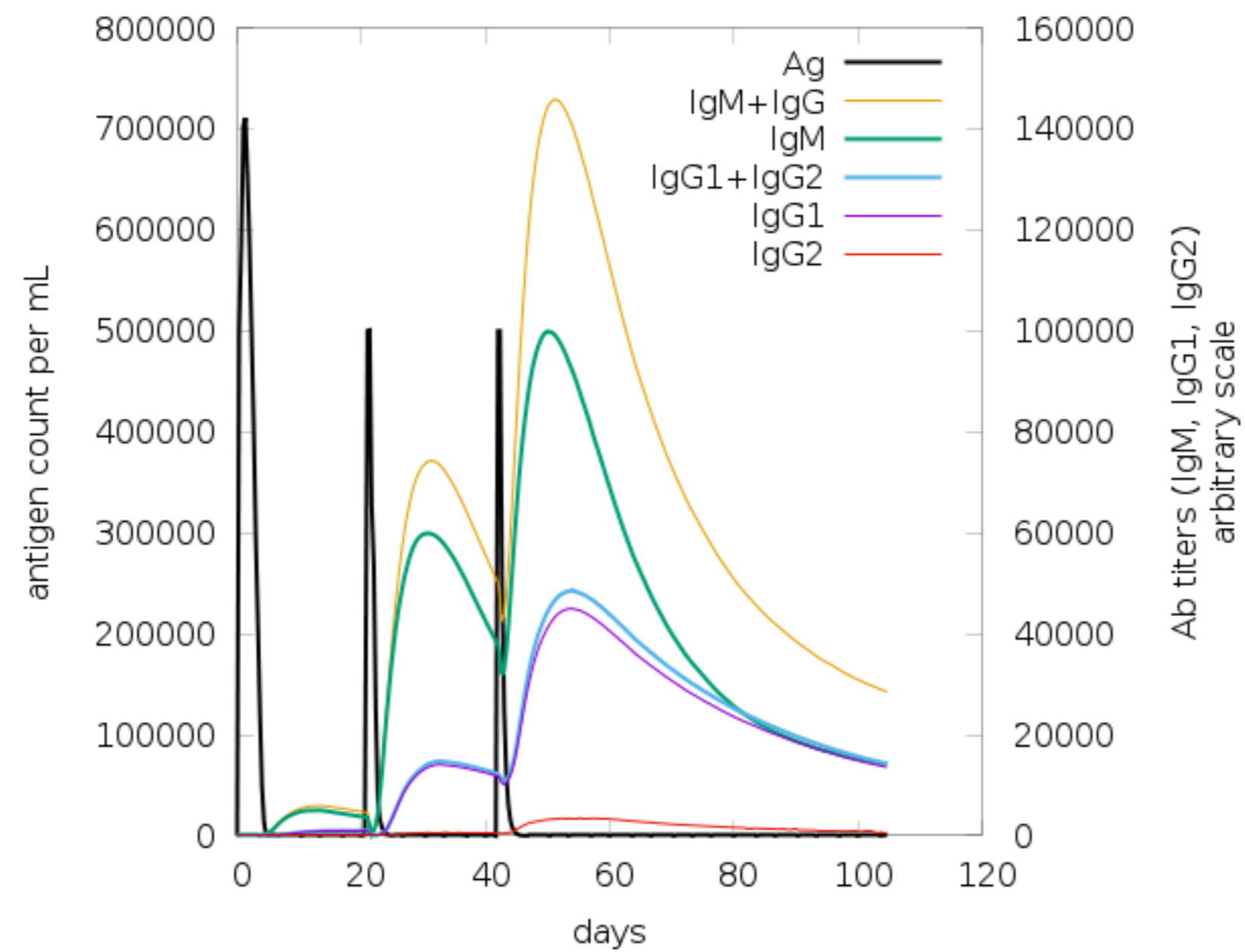

**B**

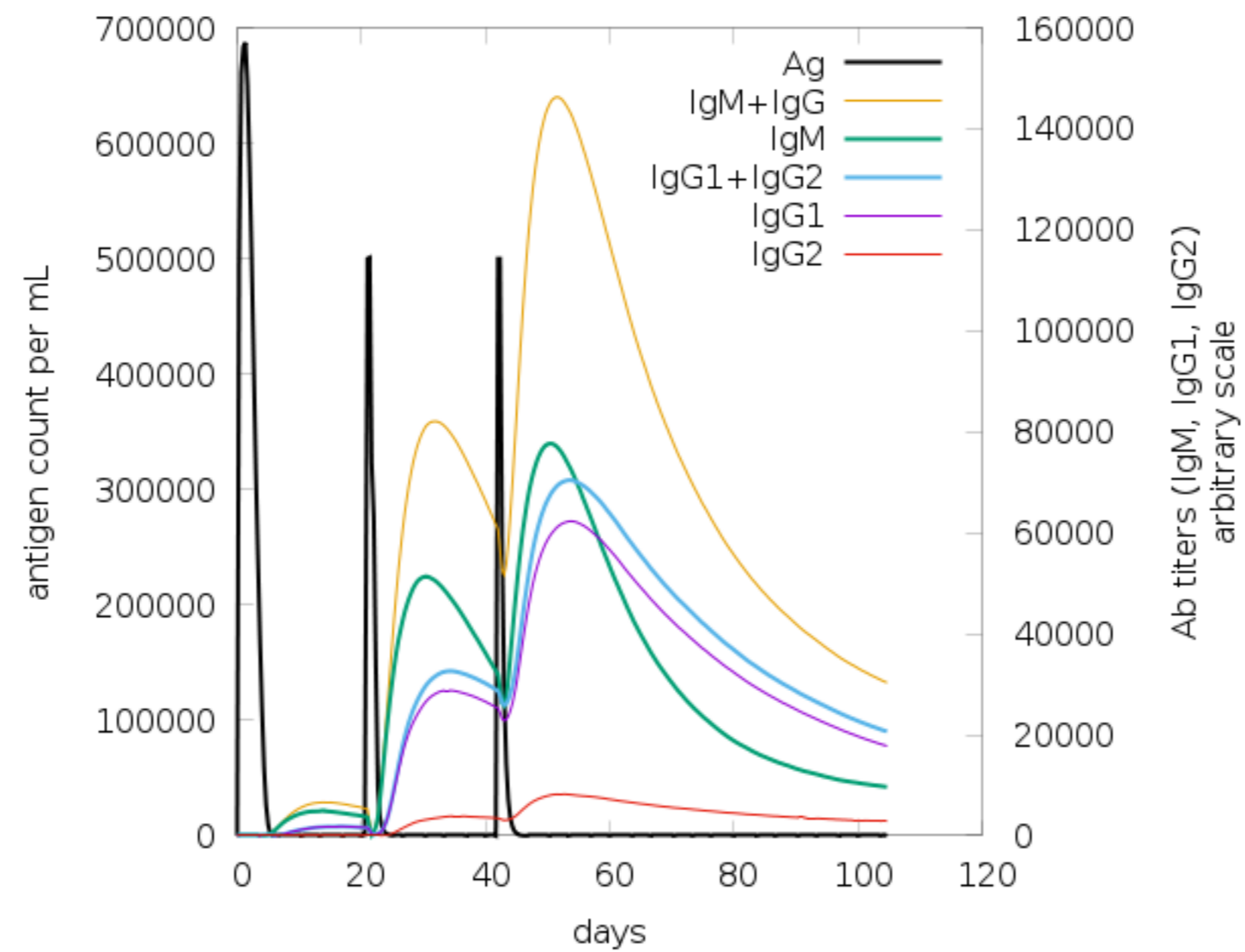

C

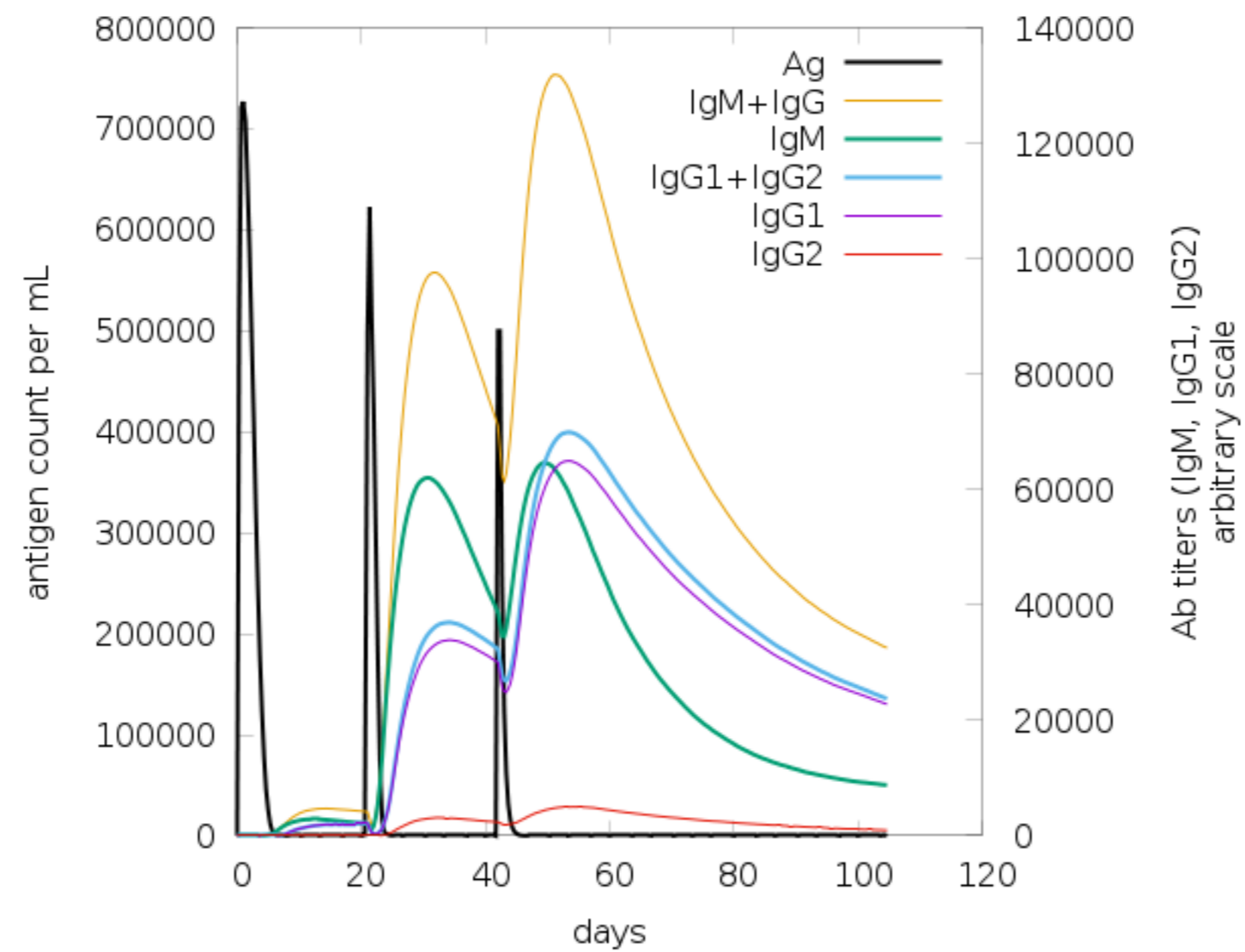

**D**

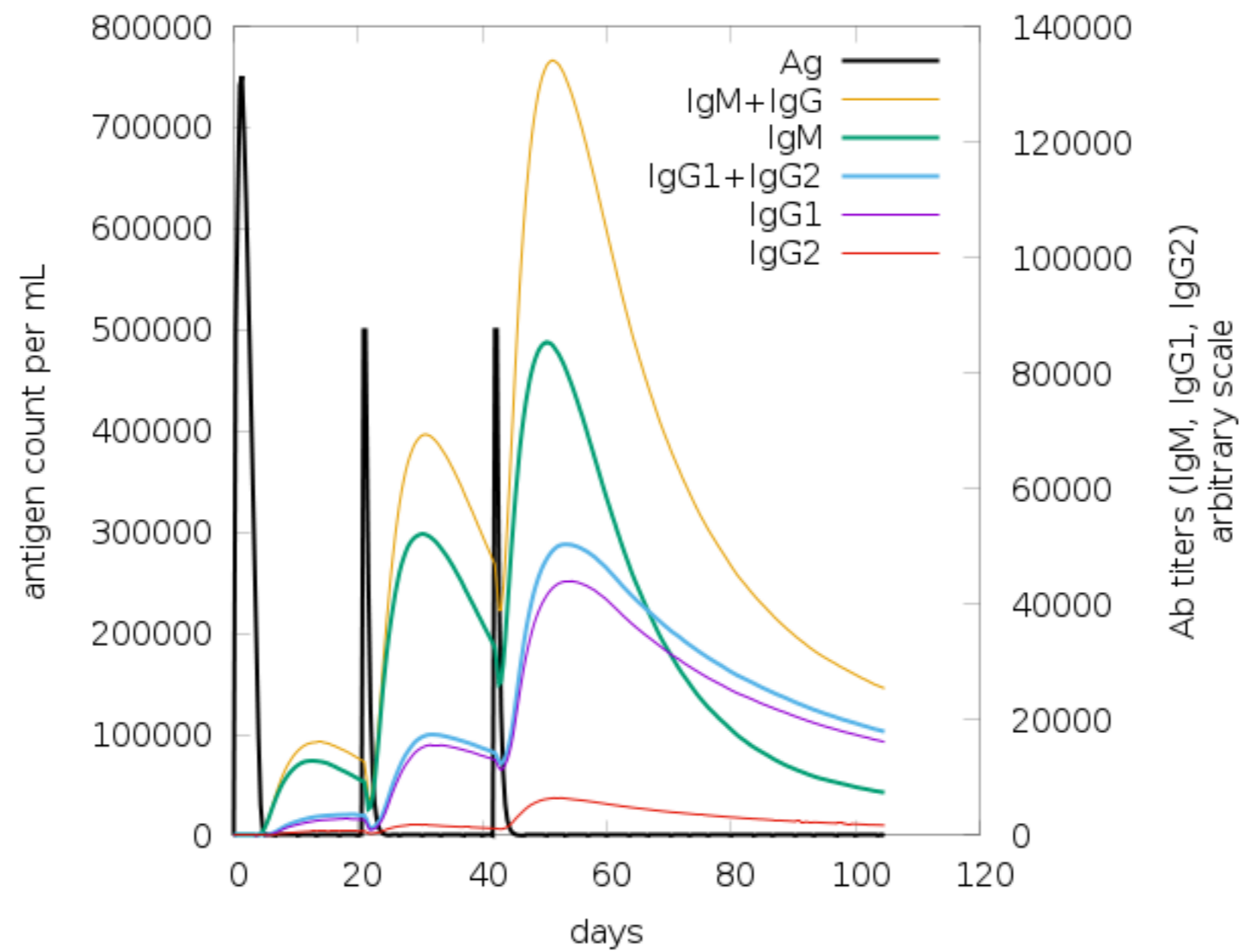

**E**

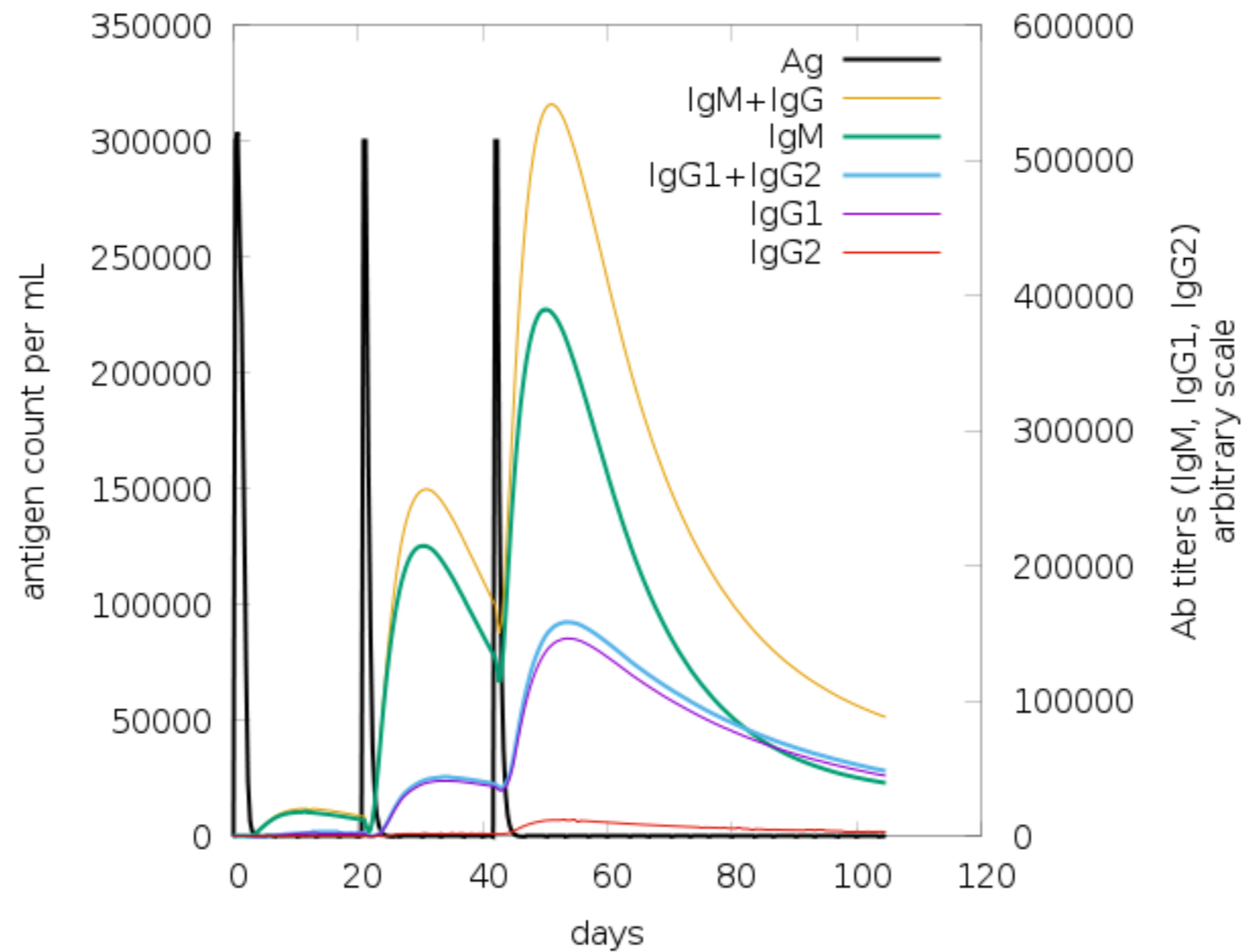

Supplement: Supplementary file 14 — Additional file 14: Figure S11. C-ImmSim prediction represented the immune profile of isotypes (IgM, IgG1 and IgG2) levels for the individual R. microplus Bm86 (A), AQP1 (B), AQP2 (C), and VgR (D) and cocktail proteins (E) as vaccine candidates. [file 13071_2025_7109_MOESM14_ESM.pdf]

A

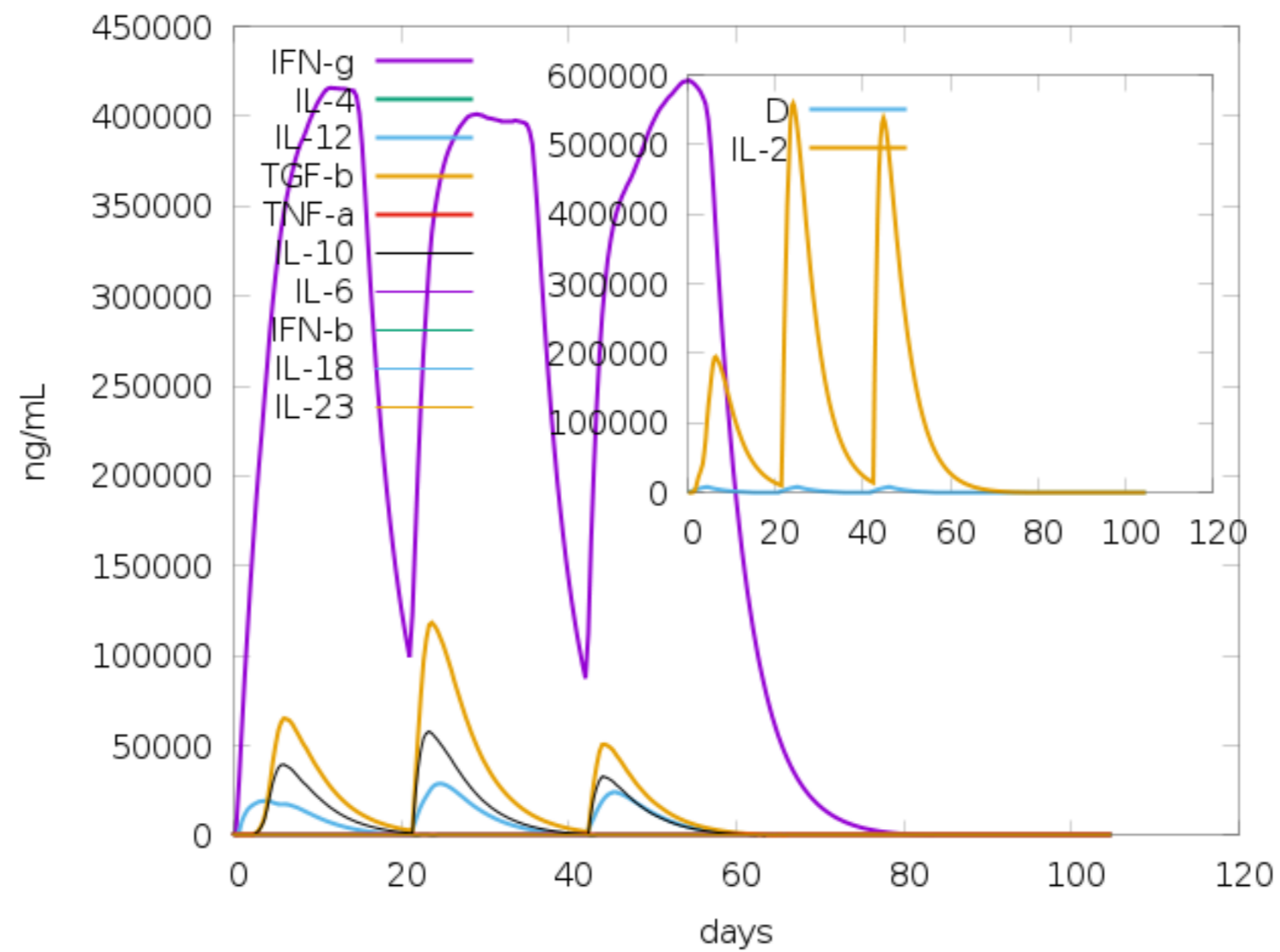

# B

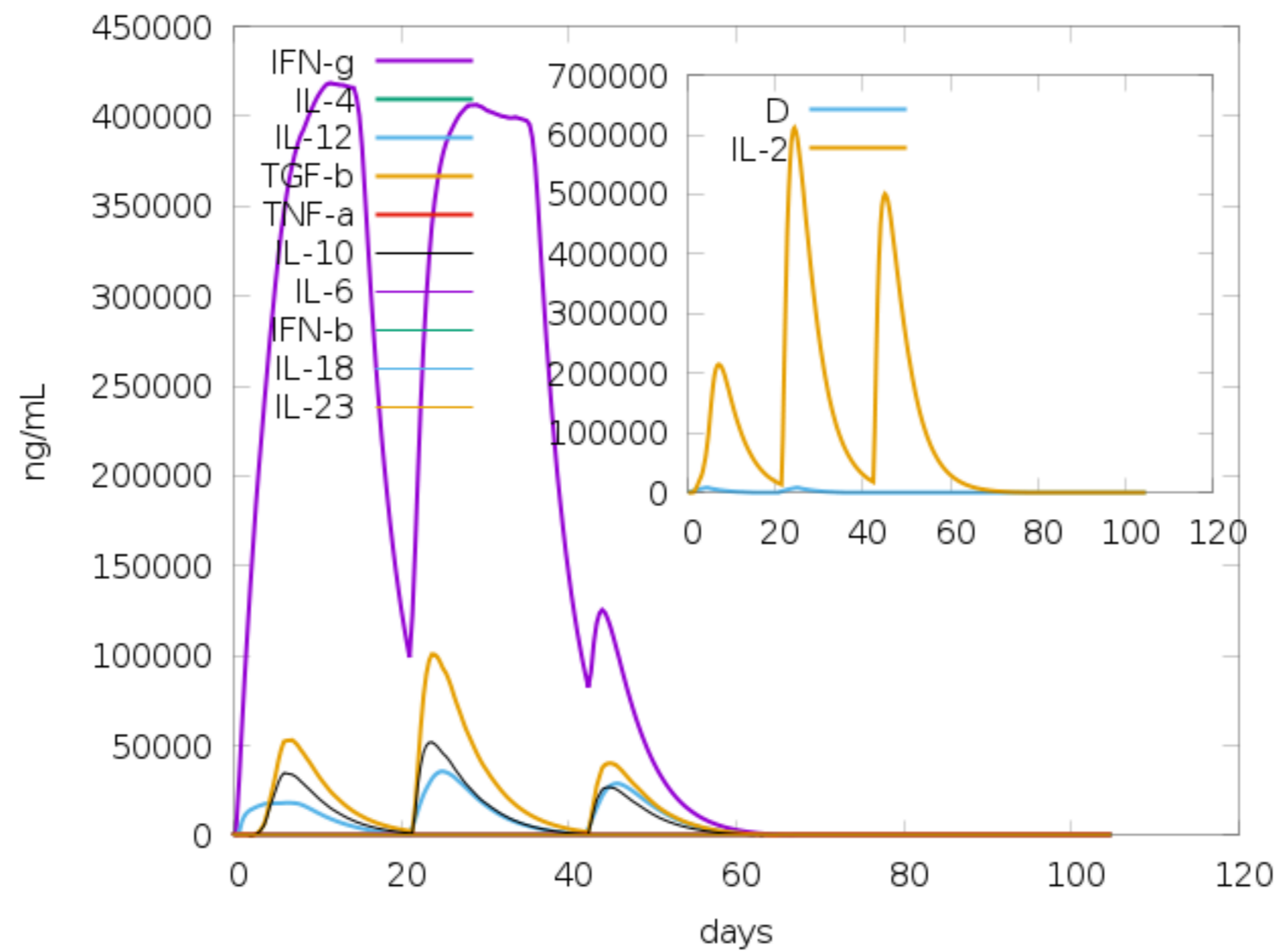

C

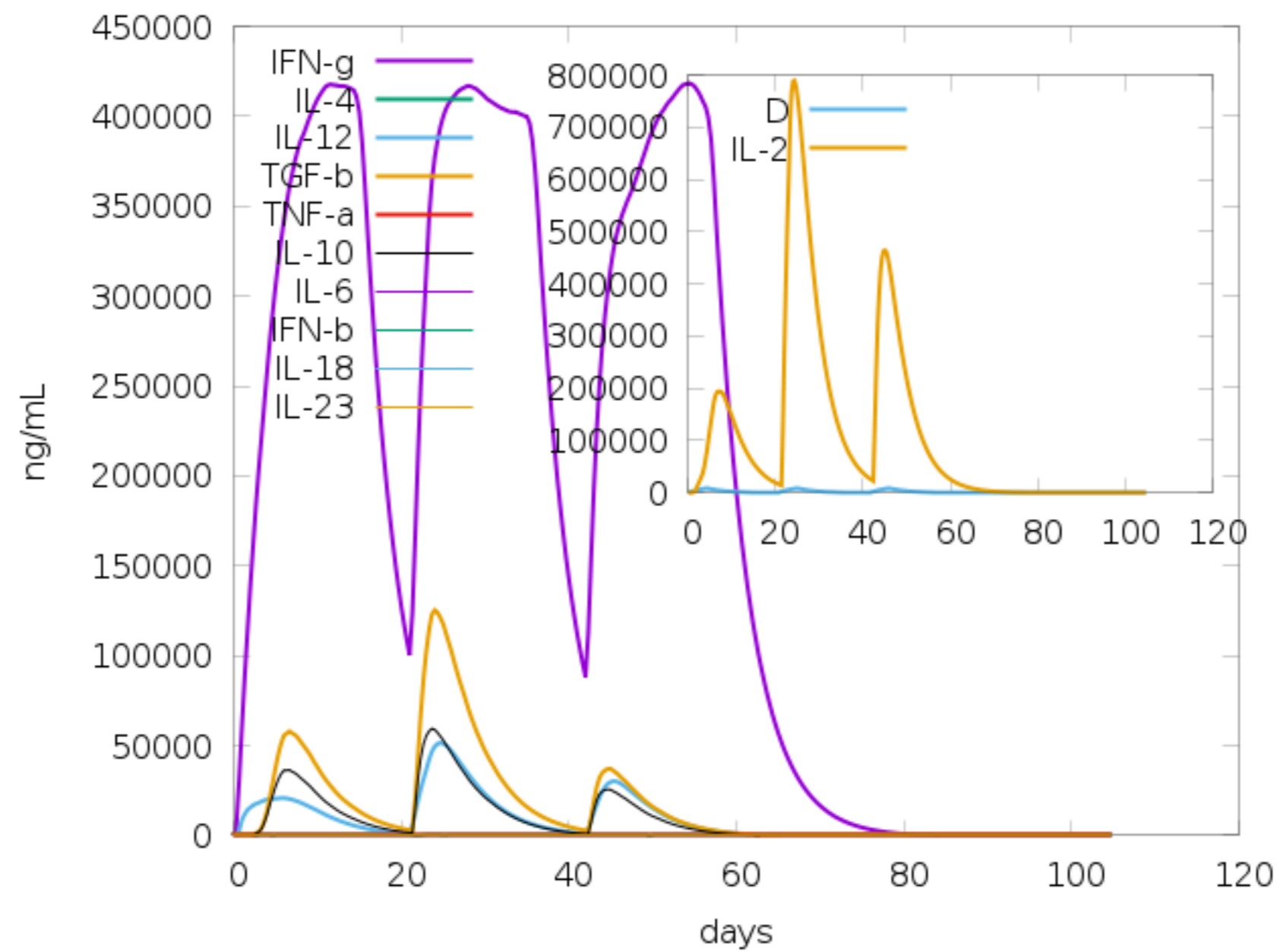

D

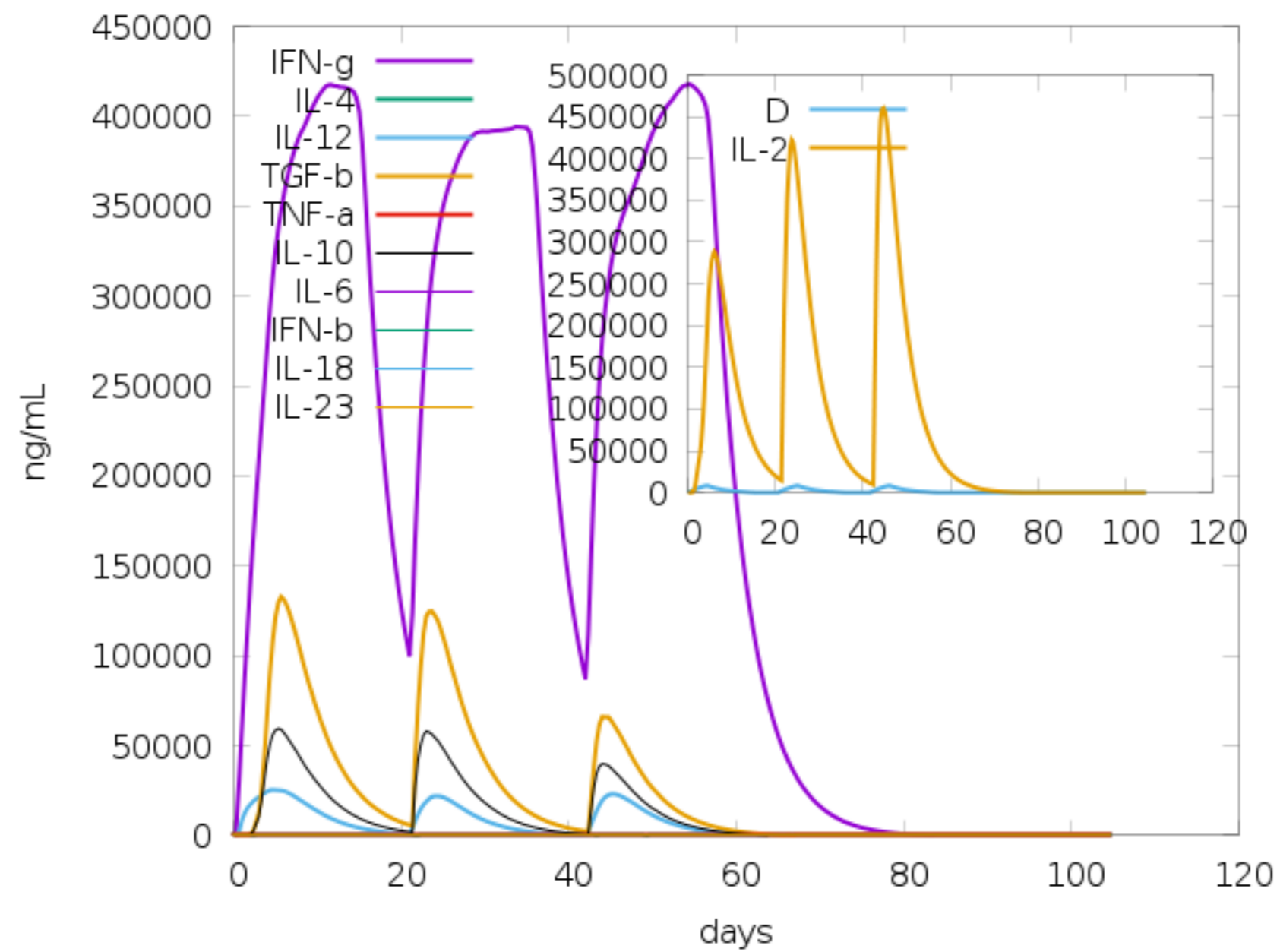

# E

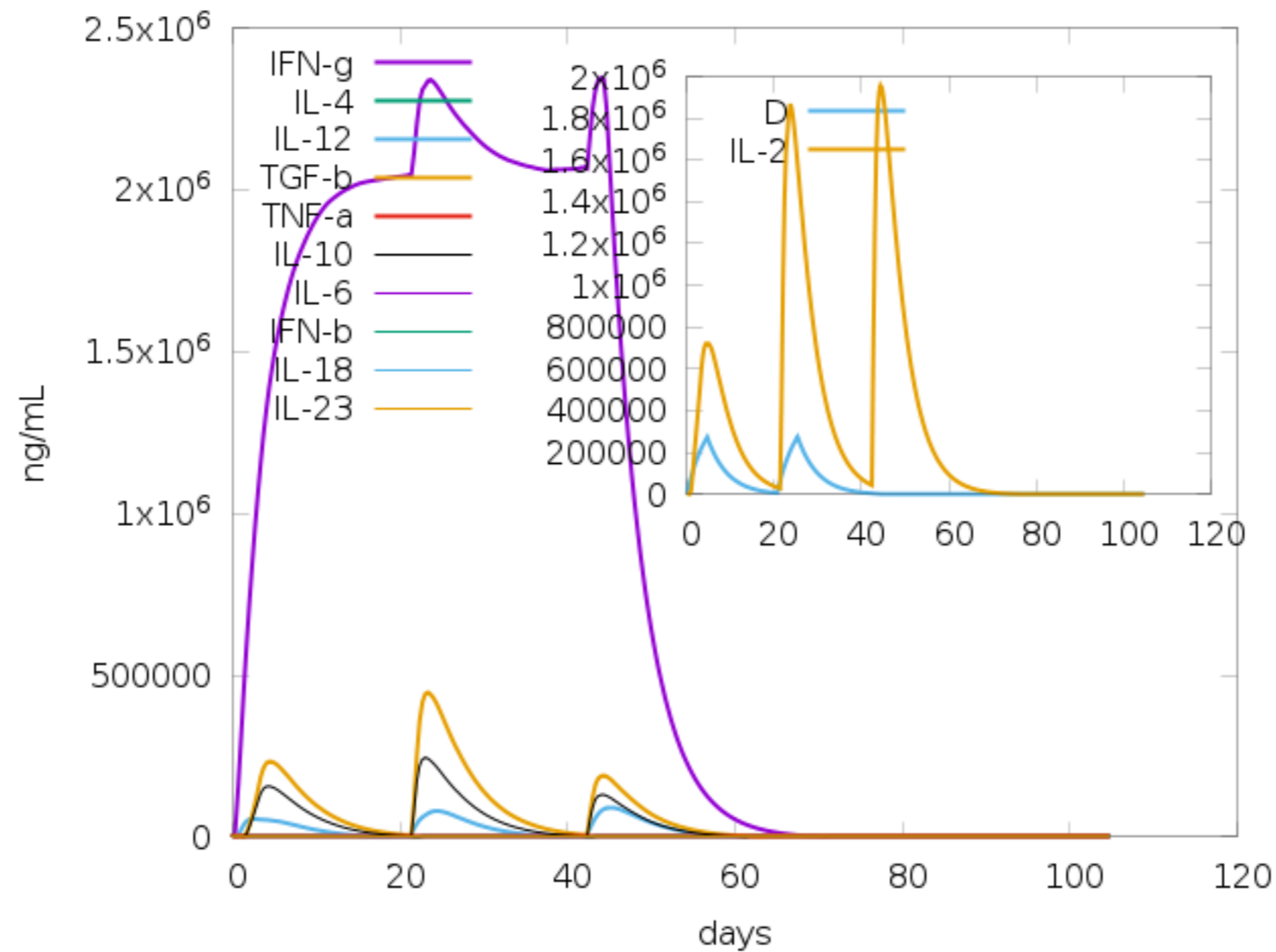

Supplement: Supplementary file 15 — Additional file 15: Figure S12. C-ImmSim prediction represented the immune profile of IFN-gamma, and IL-2 levels for the individual R. microplus Bm86 (A), AQP1 (B), AQP2 (C), and VgR (D) and cocktail proteins(E) as vaccine candidates. [file 13071_2025_7109_MOESM15_ESM.pdf]

A

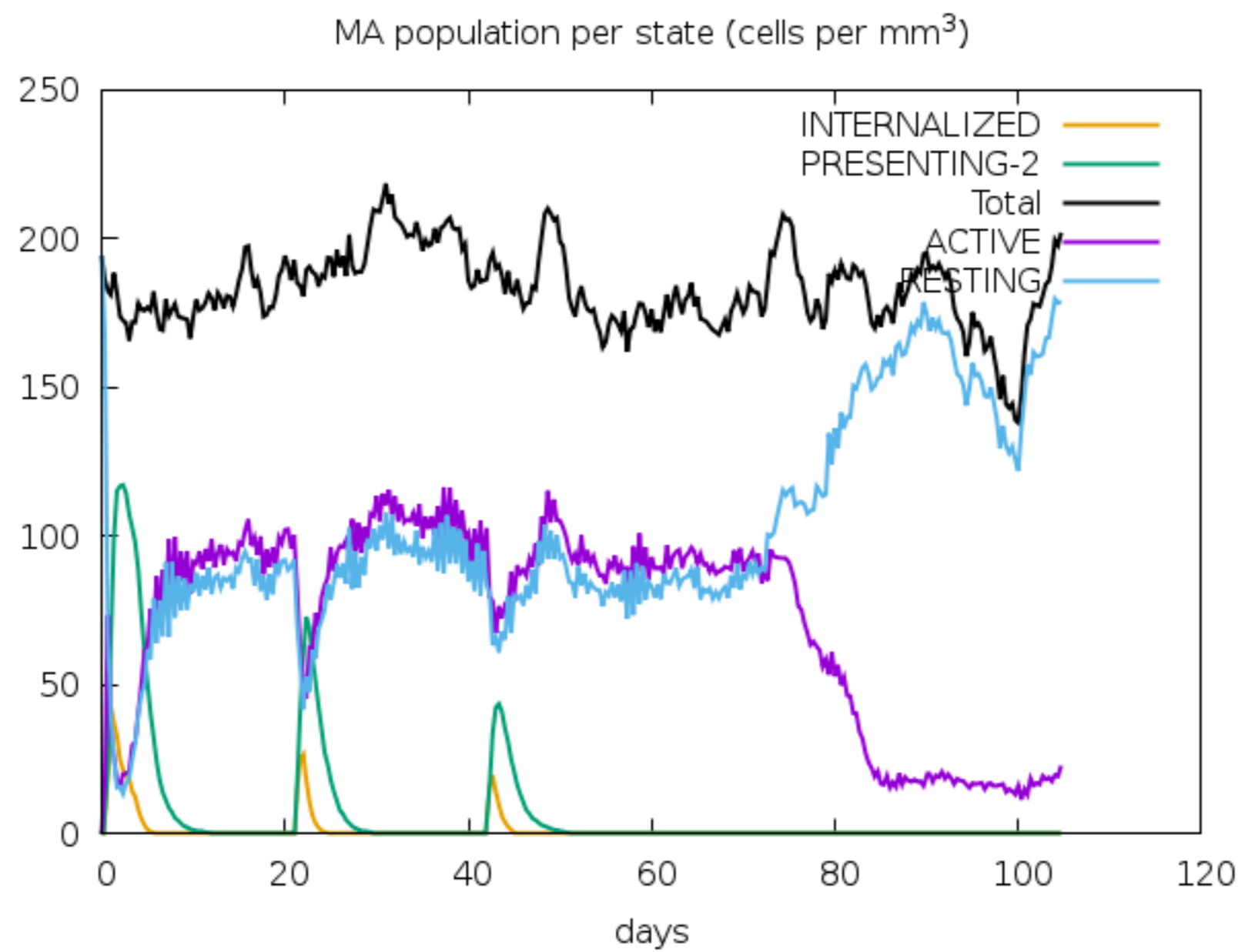

**B**

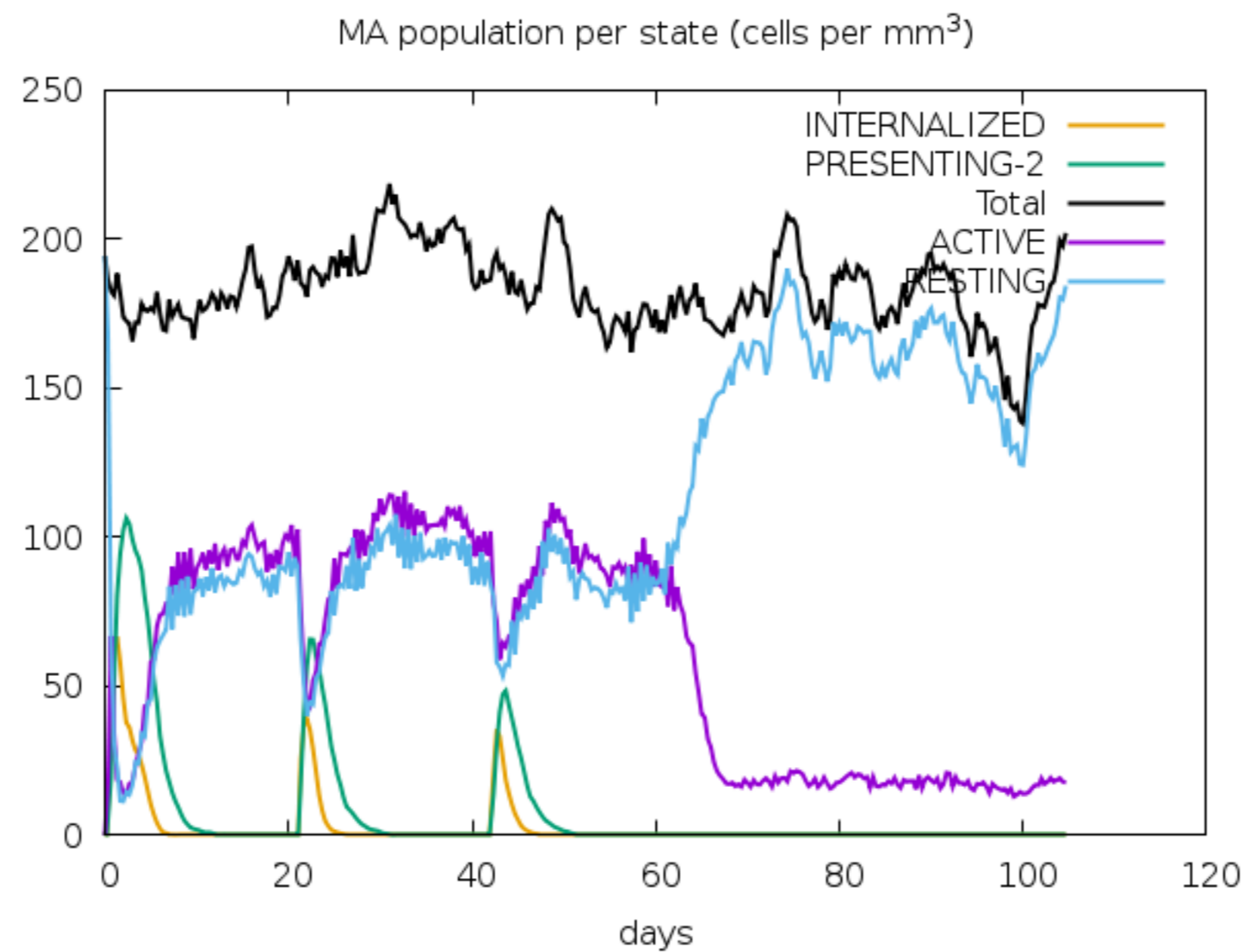

C

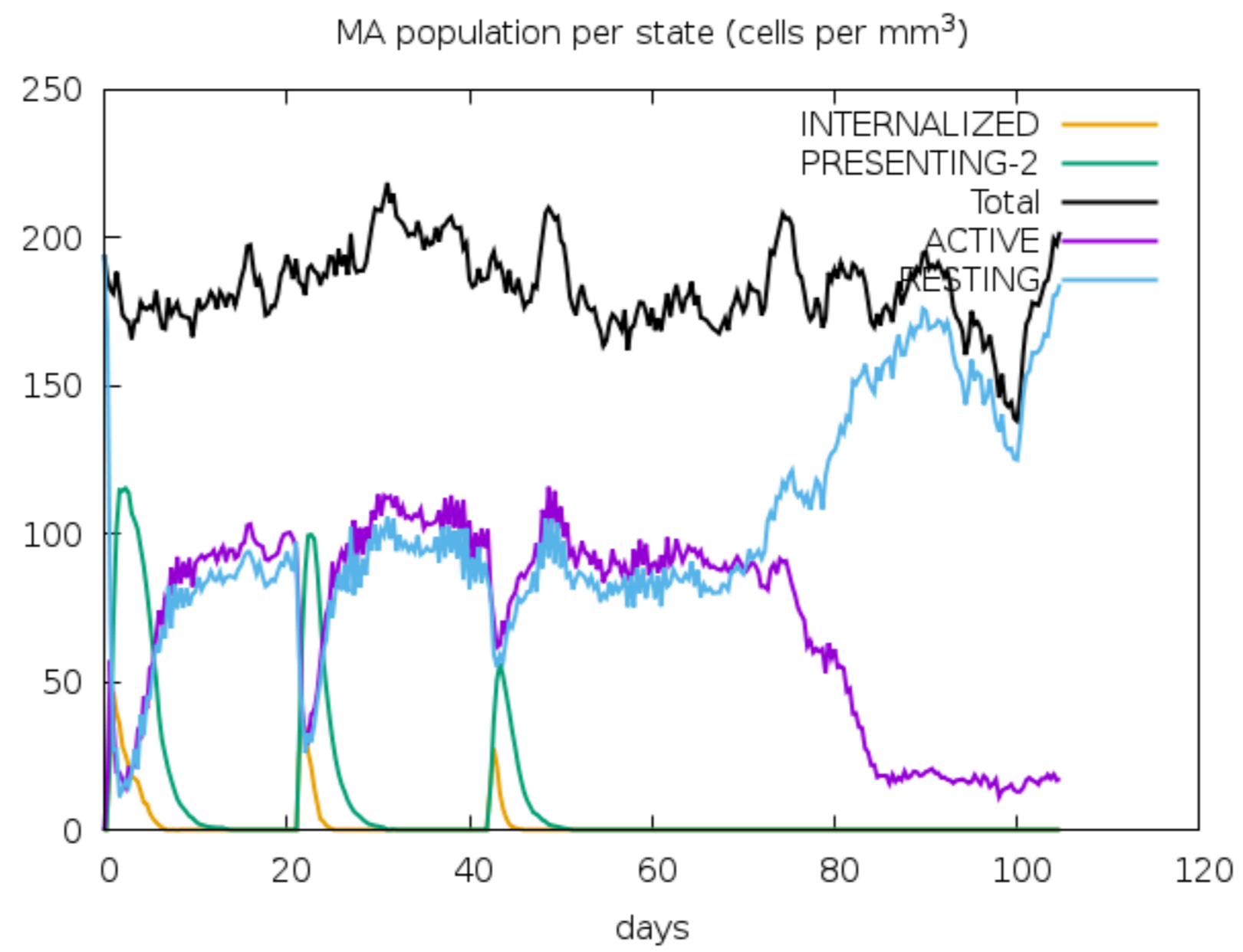

D

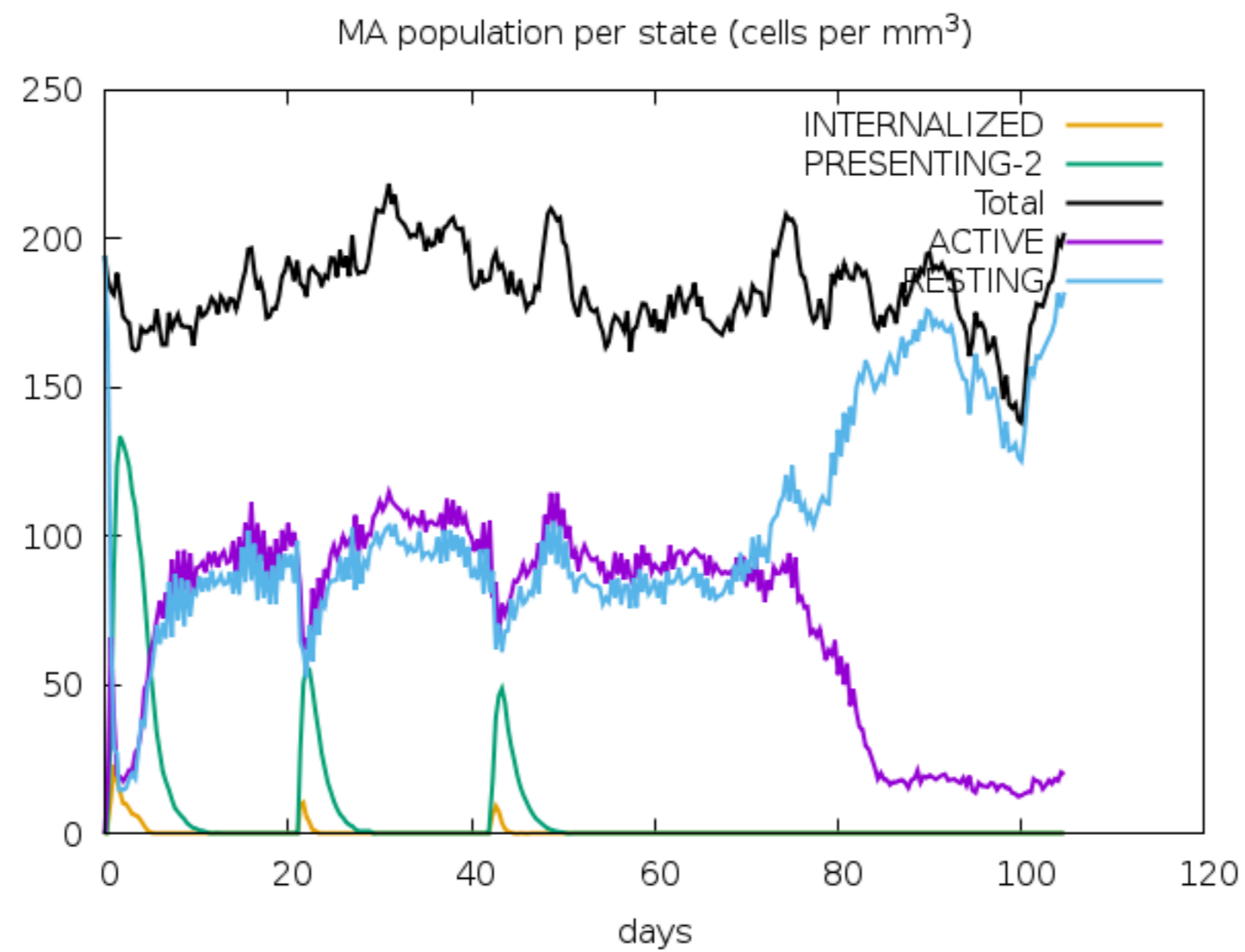

**E**

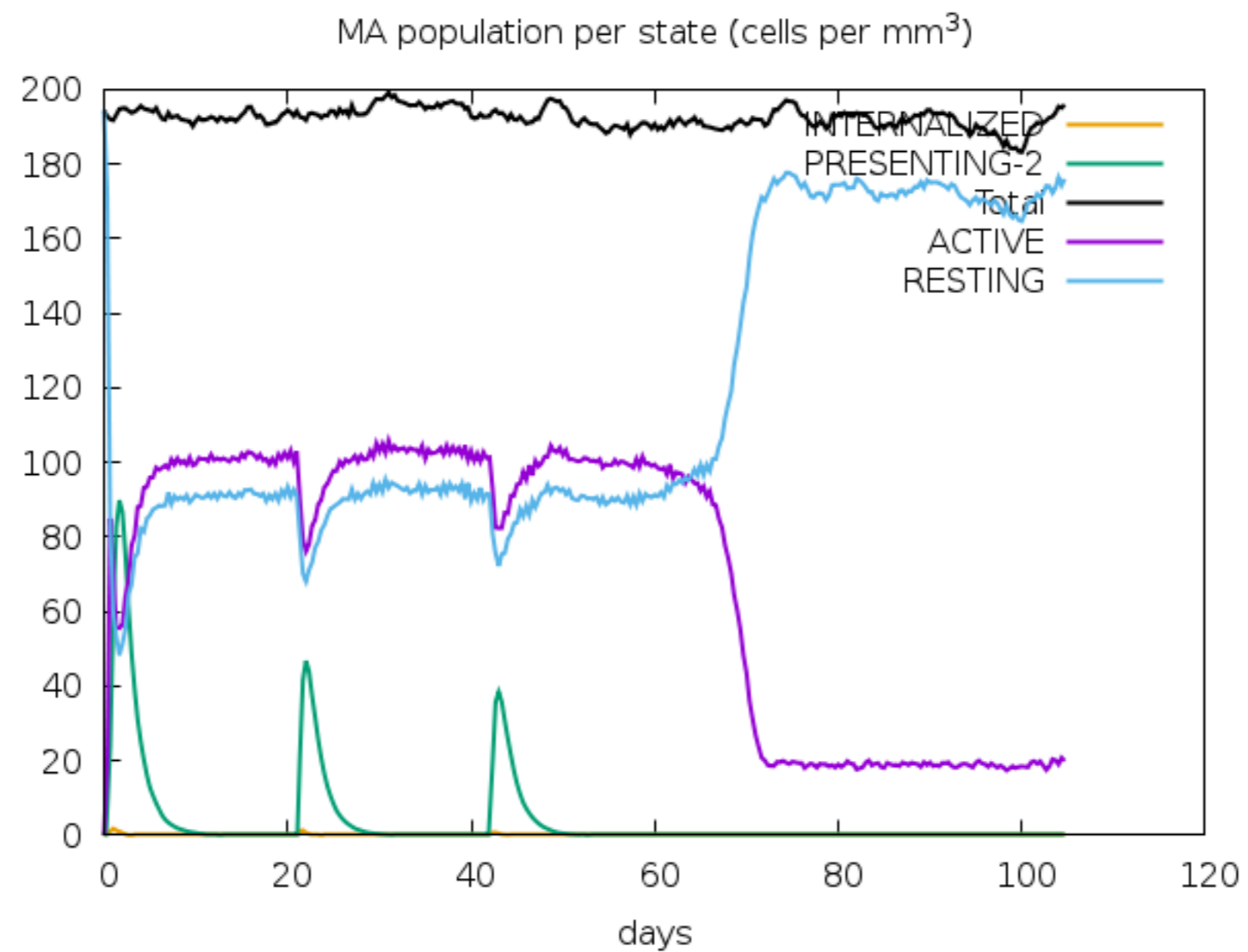

Supplement: Supplementary file 16 — Additional file 16: Figure S13. C-ImmSim prediction represented the immune profile of macrophages (MA) population state levels for the individual R. microplus Bm86 (A), AQP1 (B), AQP2 (C), and VgR (D) and cocktail proteins(E) as vaccine candidates. [file 13071_2025_7109_MOESM16_ESM.pdf]

A

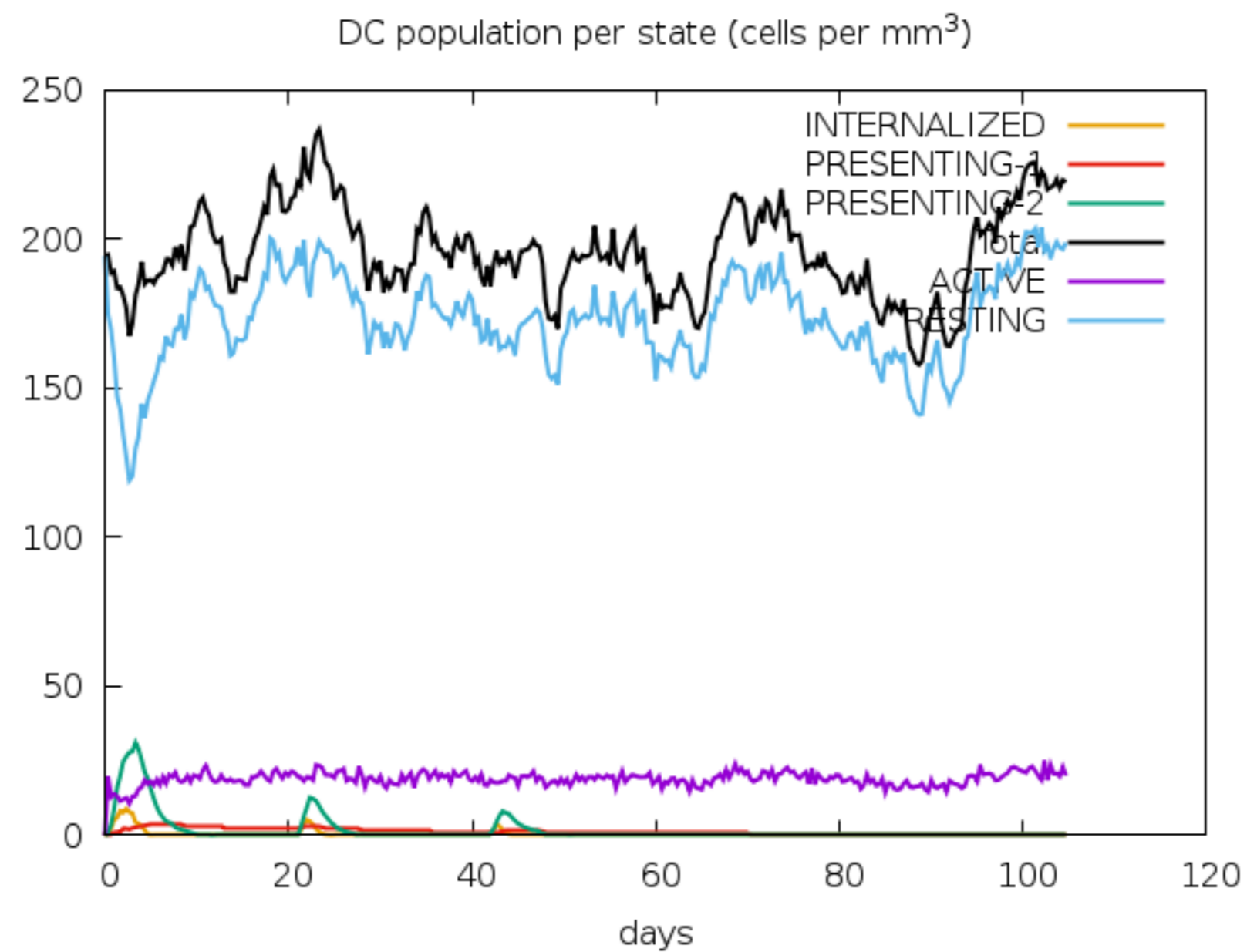

**B**

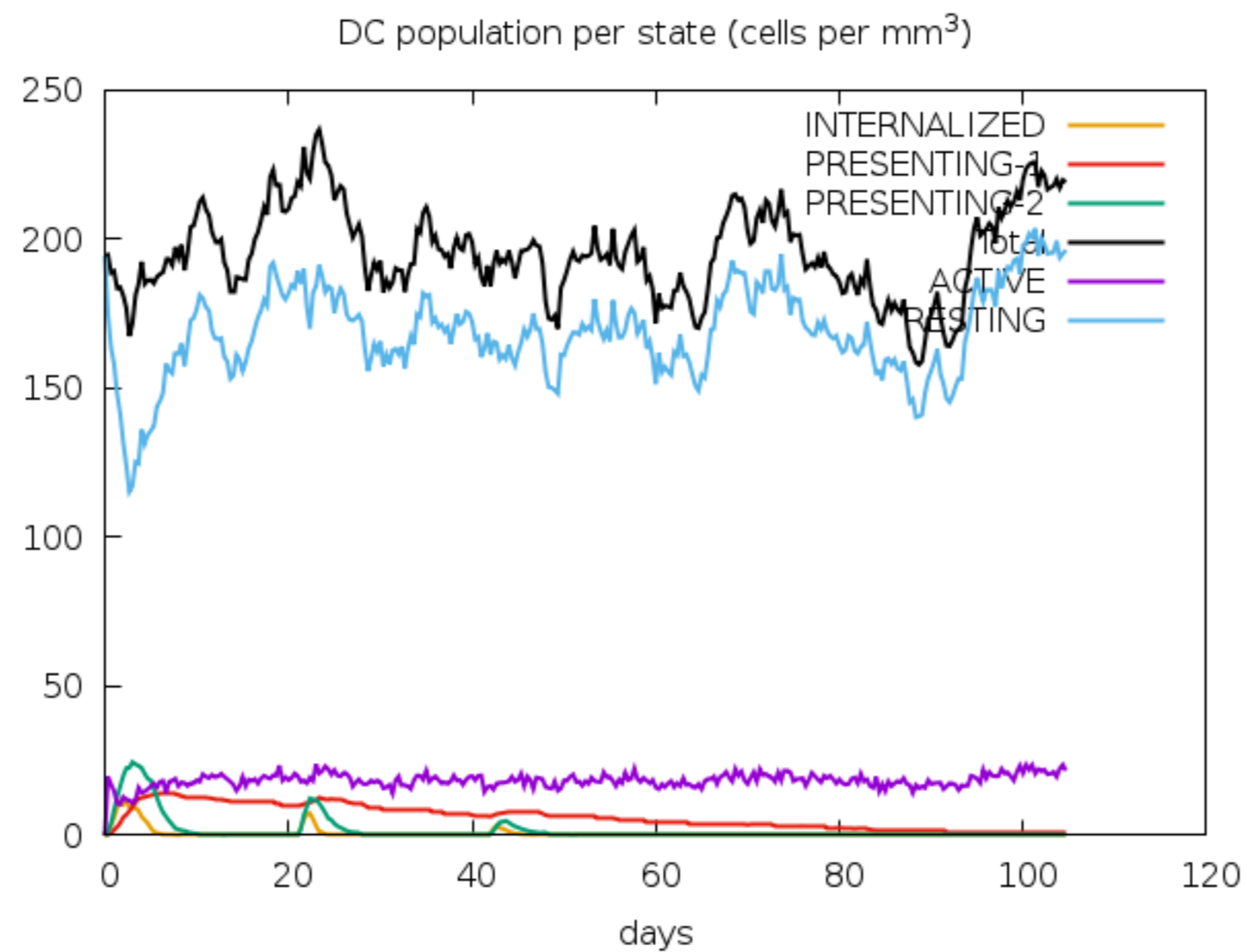

C

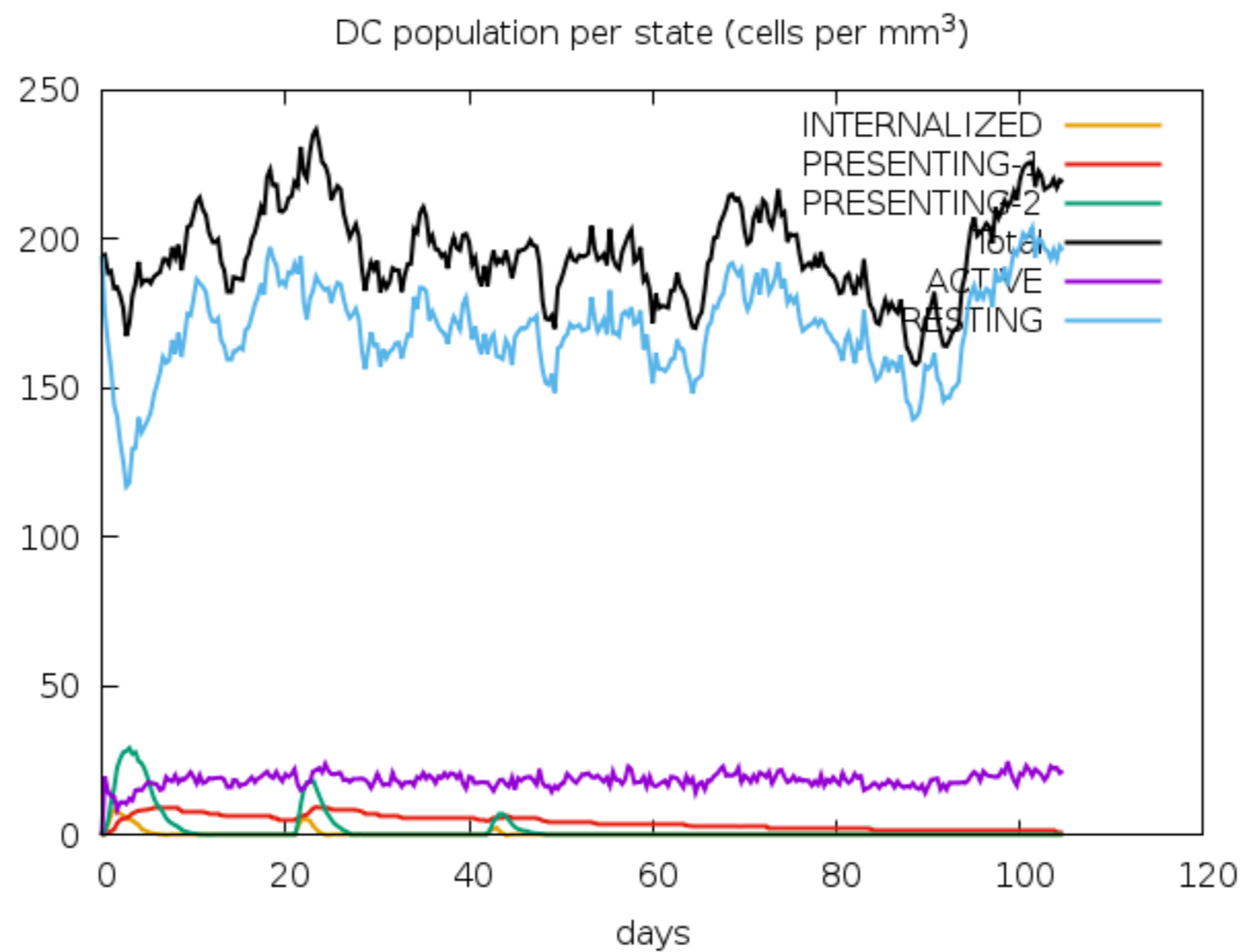

**D**

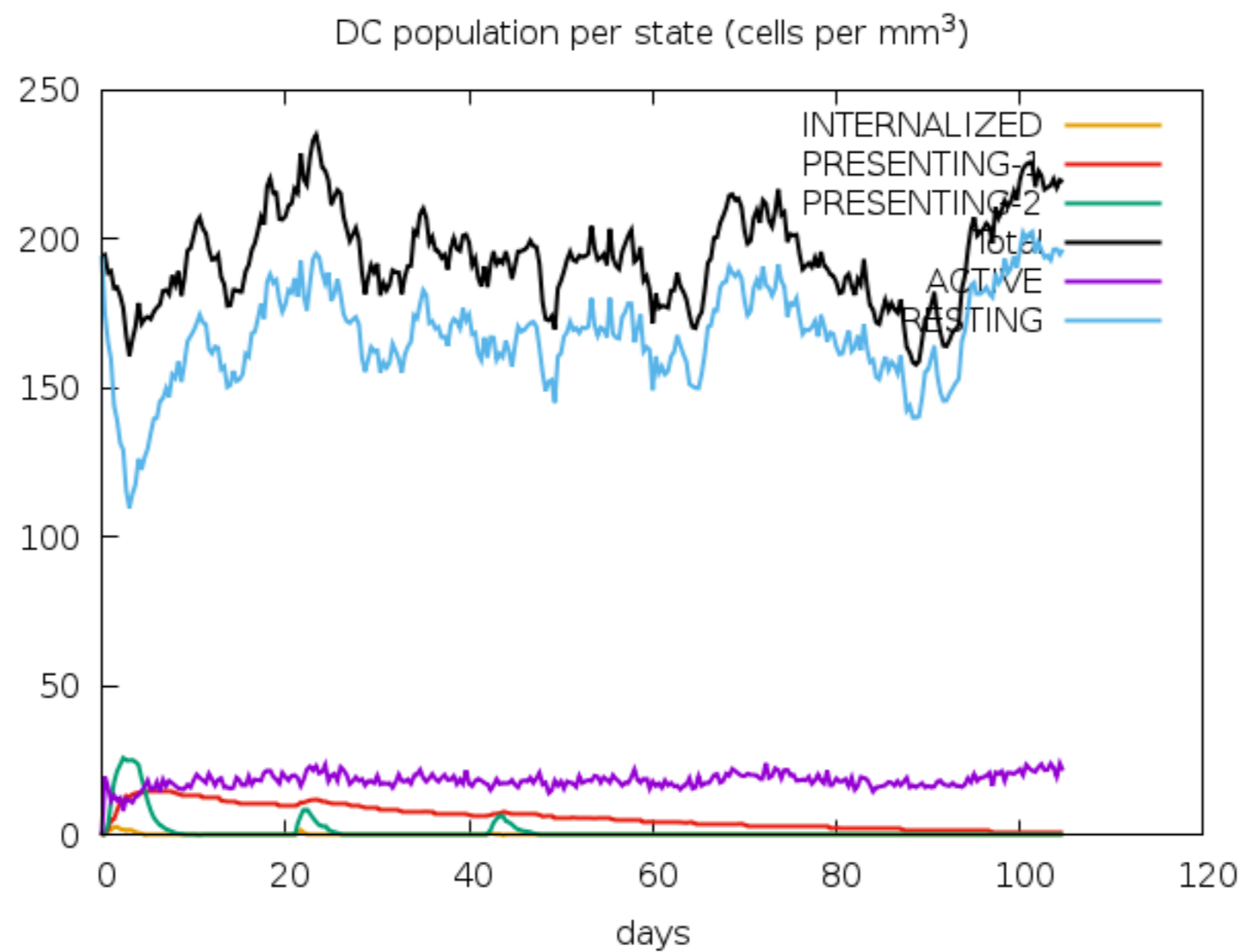

**E**

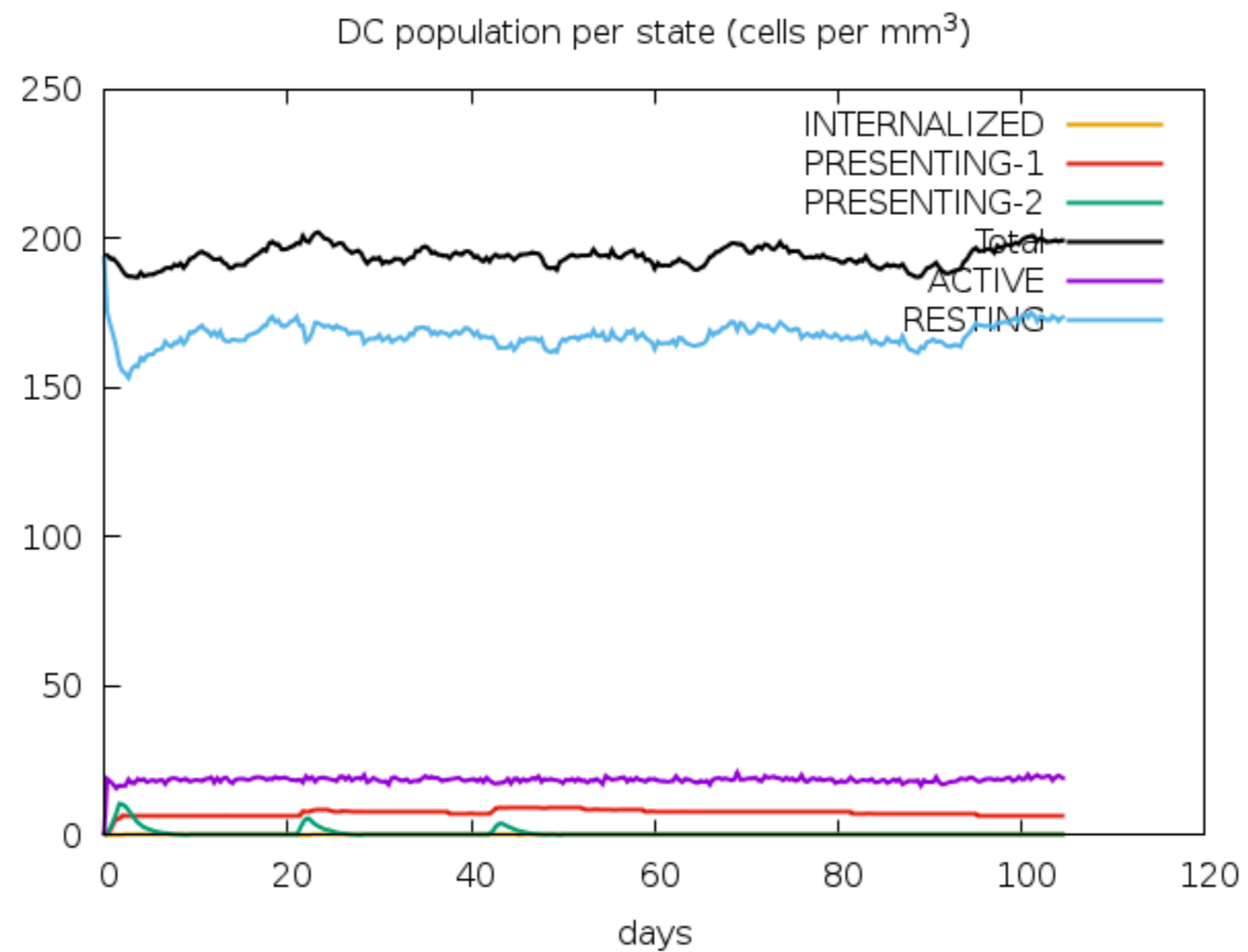

Supplement: Supplementary file 17 — Additional file 17: Figure S14. C-ImmSim prediction represented the immune profile of dendritic cell (DC) population state levels for the individual R. microplus Bm86 (A), AQP1 (B), AQP2 (C), and VgR (D) and cocktail proteins(E) as vaccine candidates. [file 13071_2025_7109_MOESM17_ESM.pdf]

A

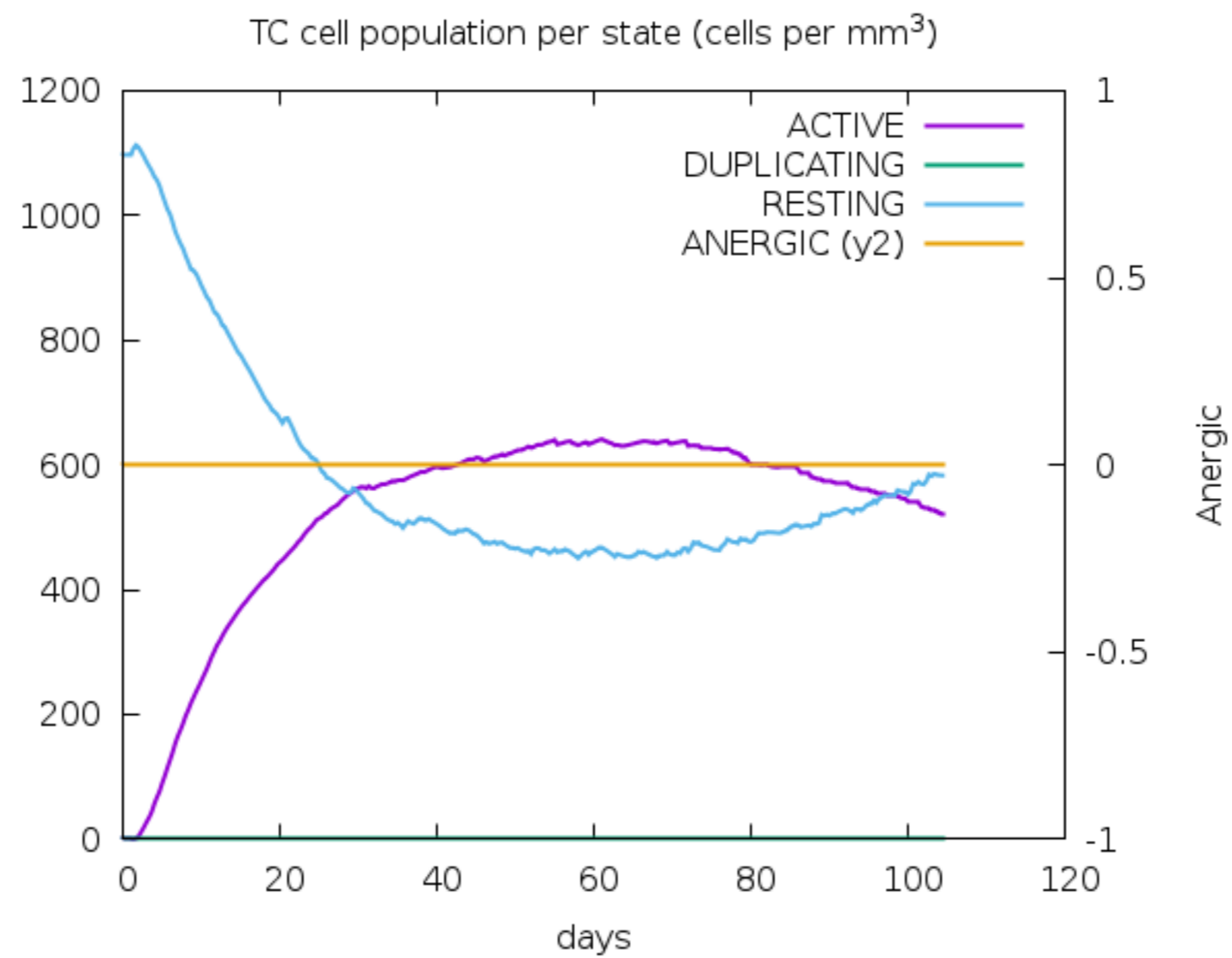

**B**

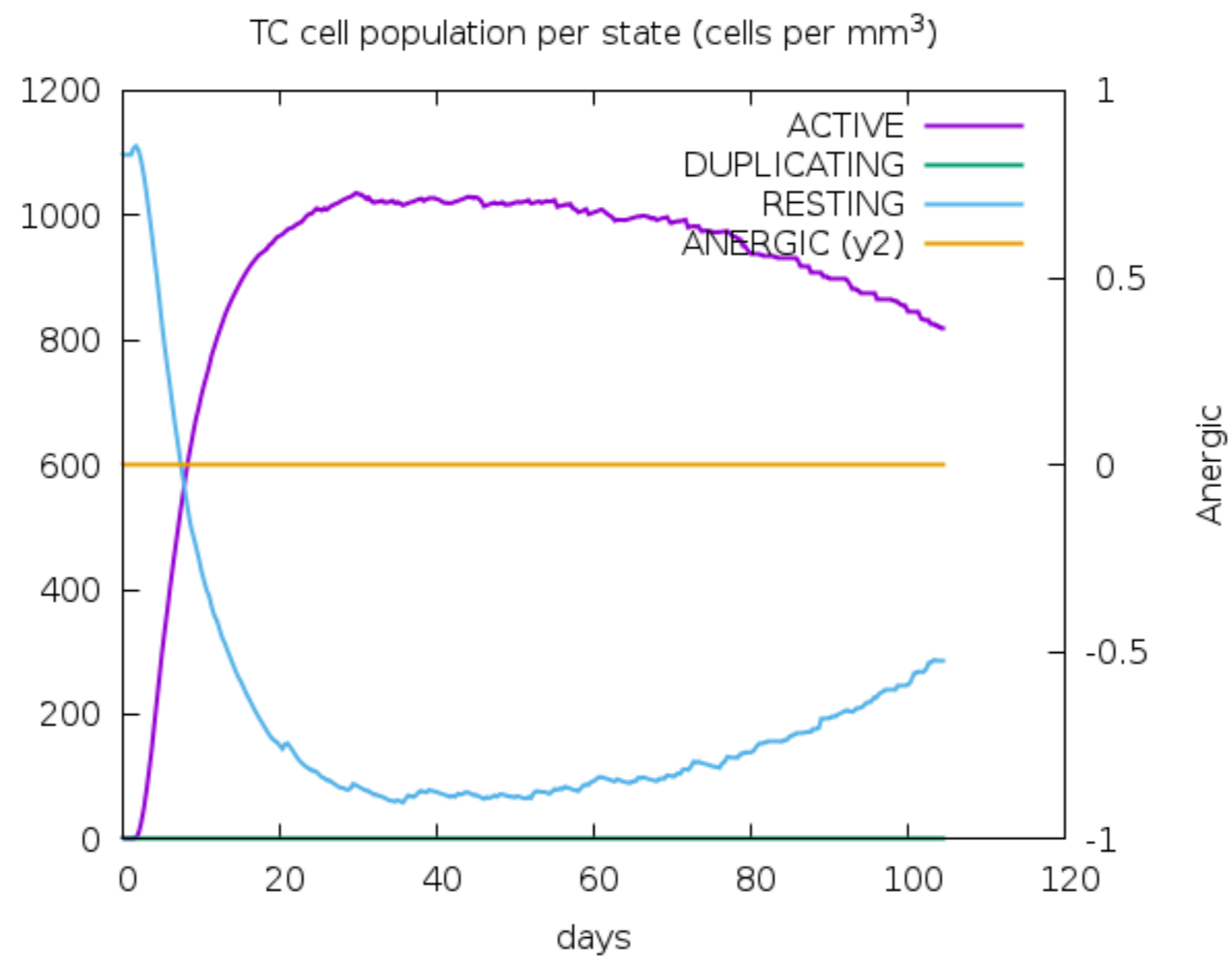

C

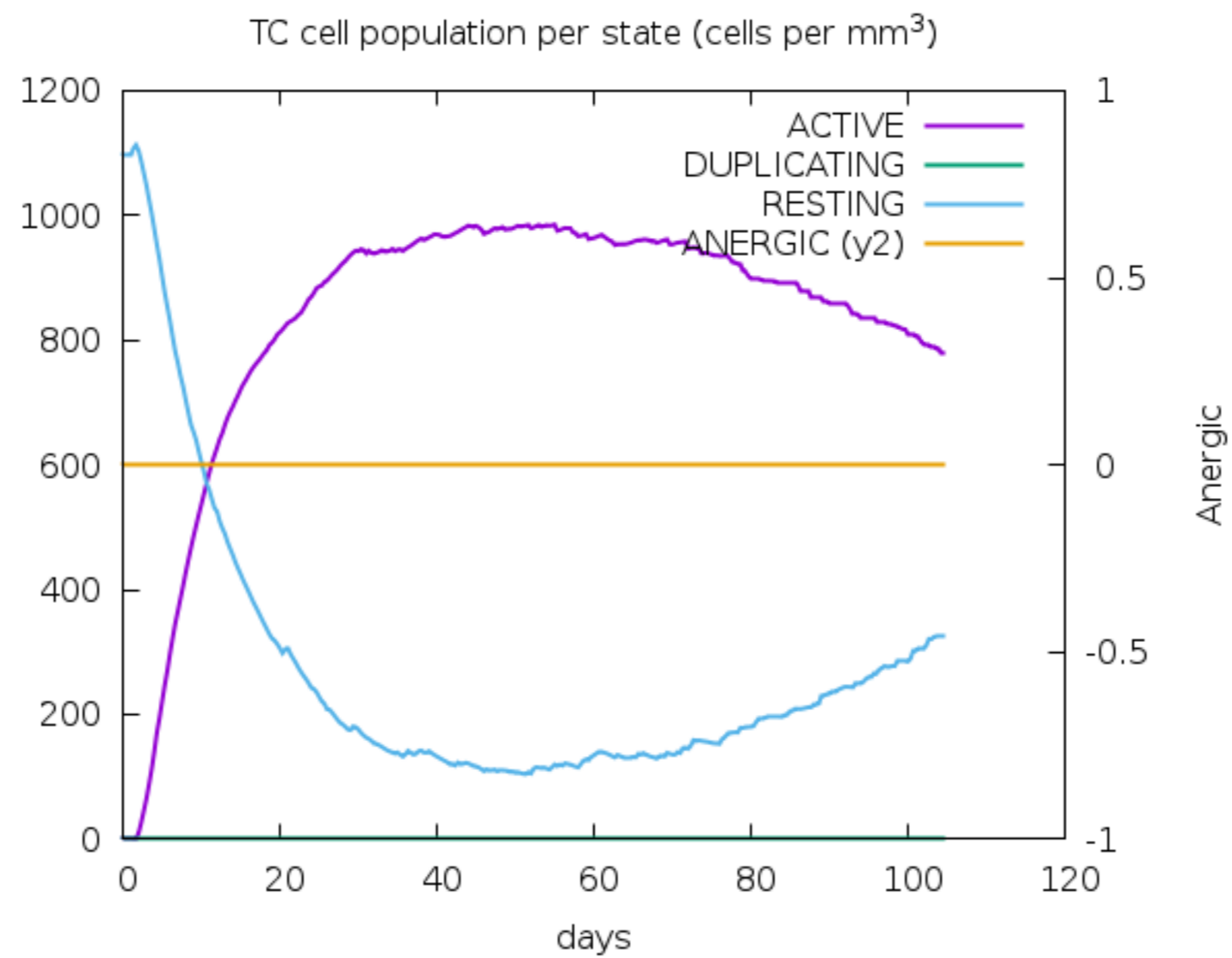

**D**

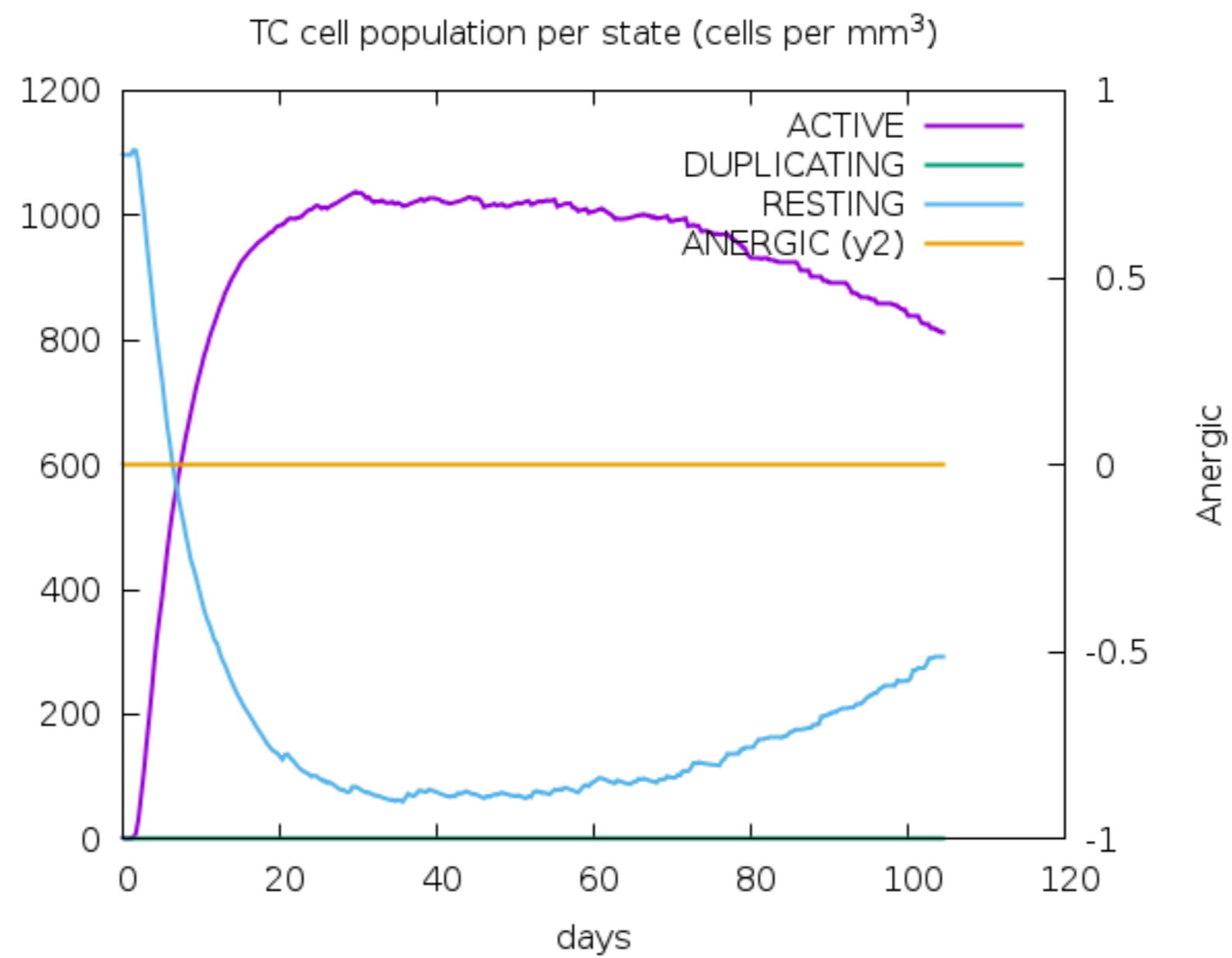

**E**

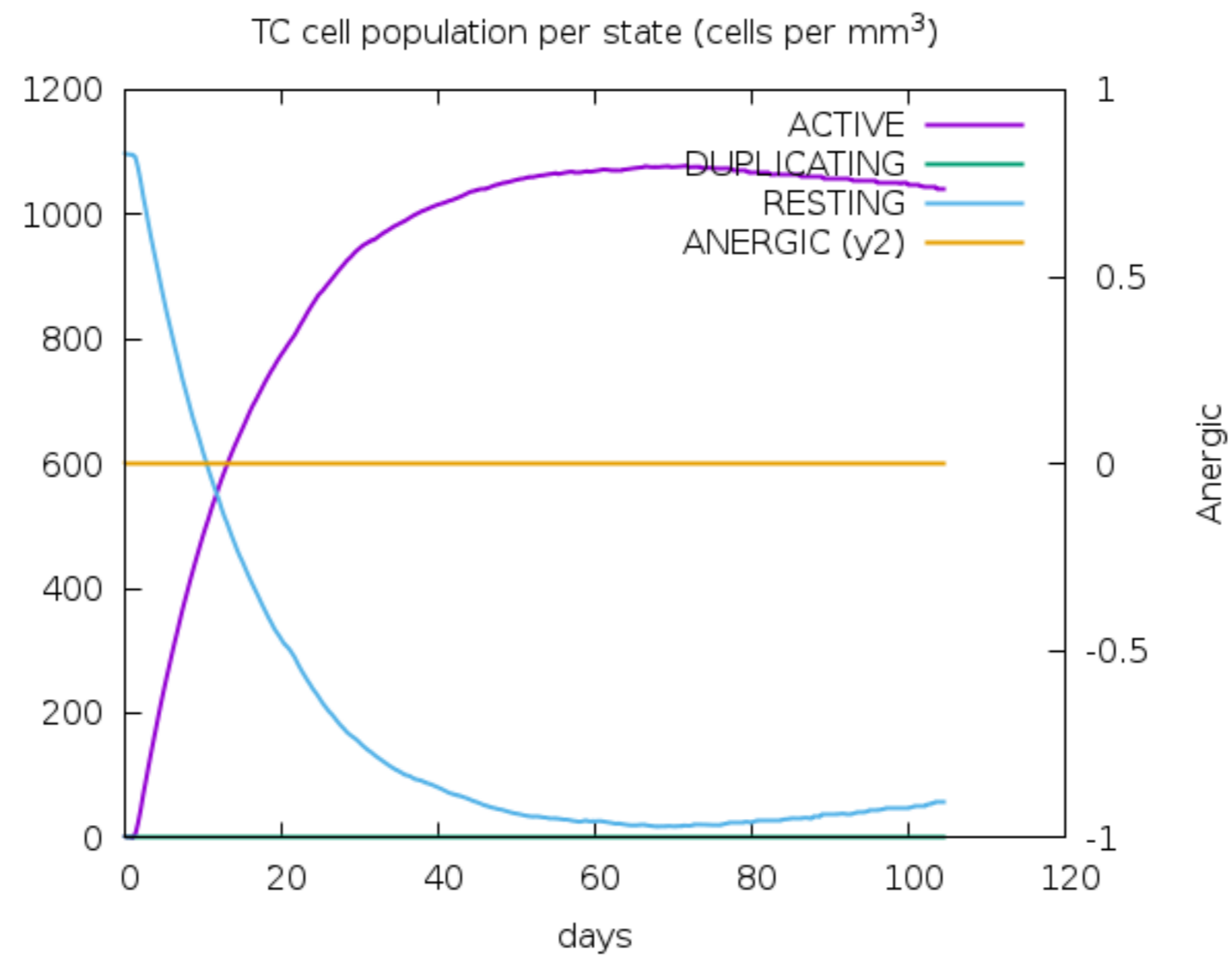

Supplement: Supplementary file 18 — Additional file 18: Figure S15. C-ImmSim prediction represented the immune profile of T-lymphocytes (TC) population state levels for the individual R. microplus Bm86 (A), AQP1 (B), AQP2 (C), and VgR (D) and cocktail proteins(E) as vaccine candidates. [file 13071_2025_7109_MOESM18_ESM.pdf]

A

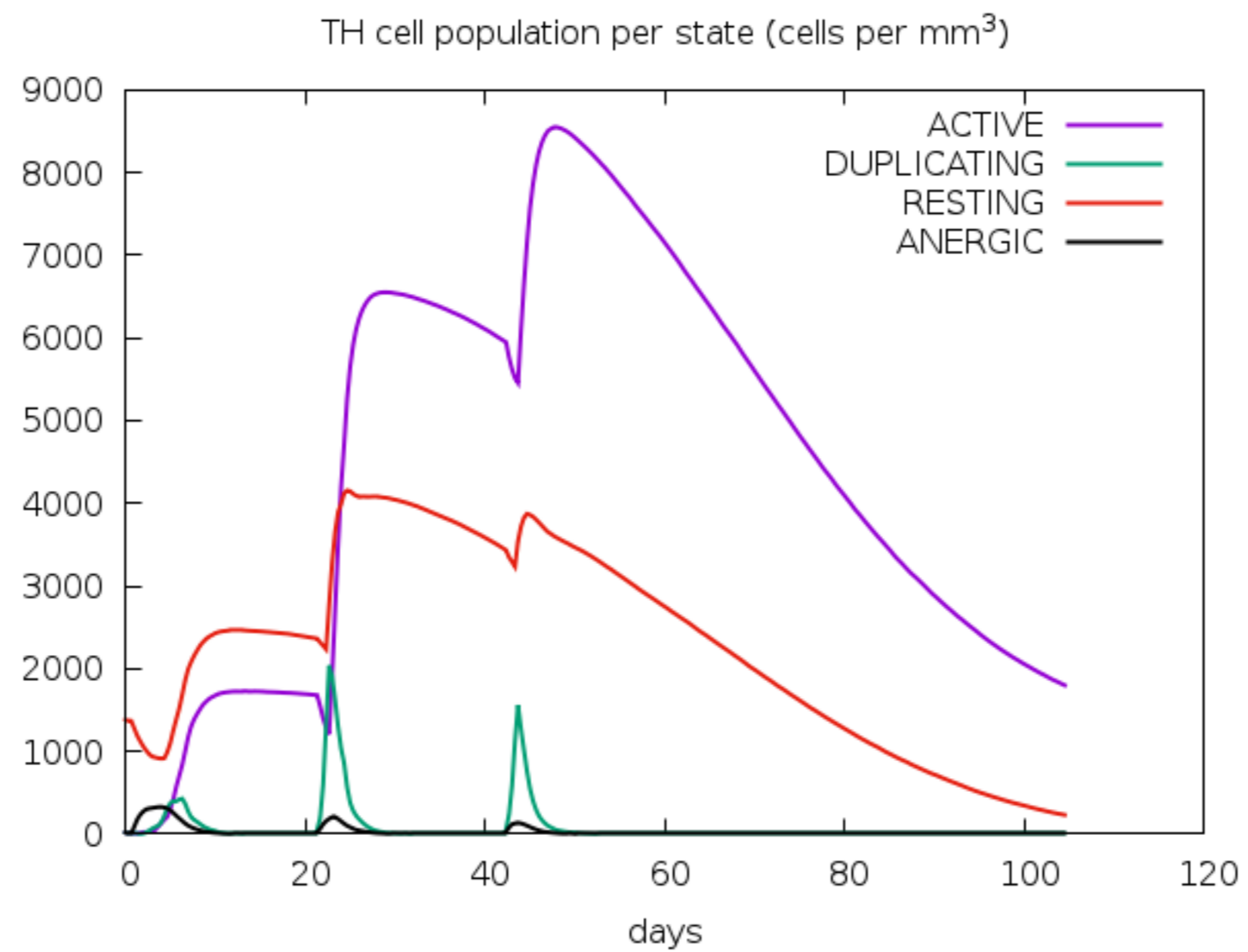

**B**

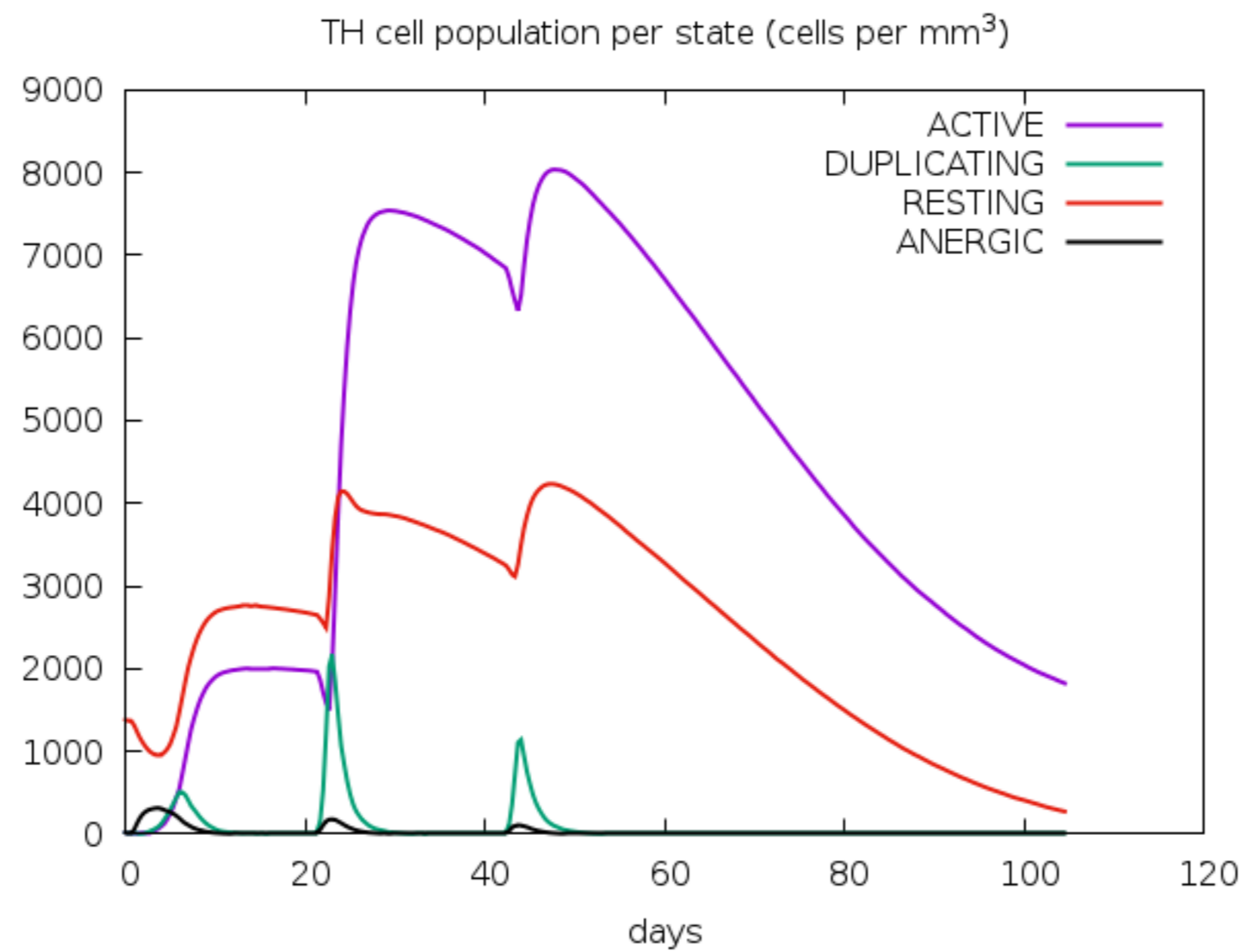

C

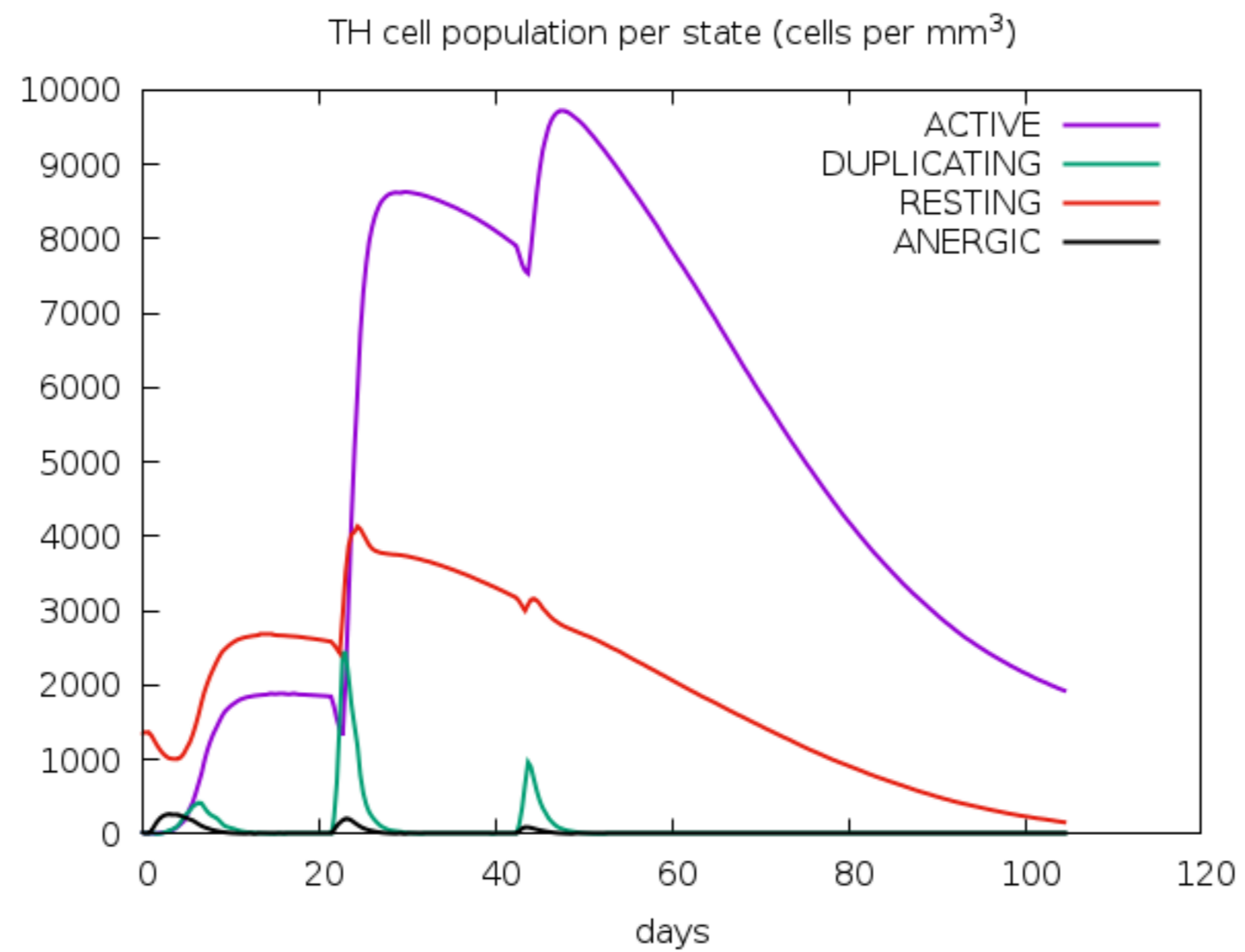

**D**

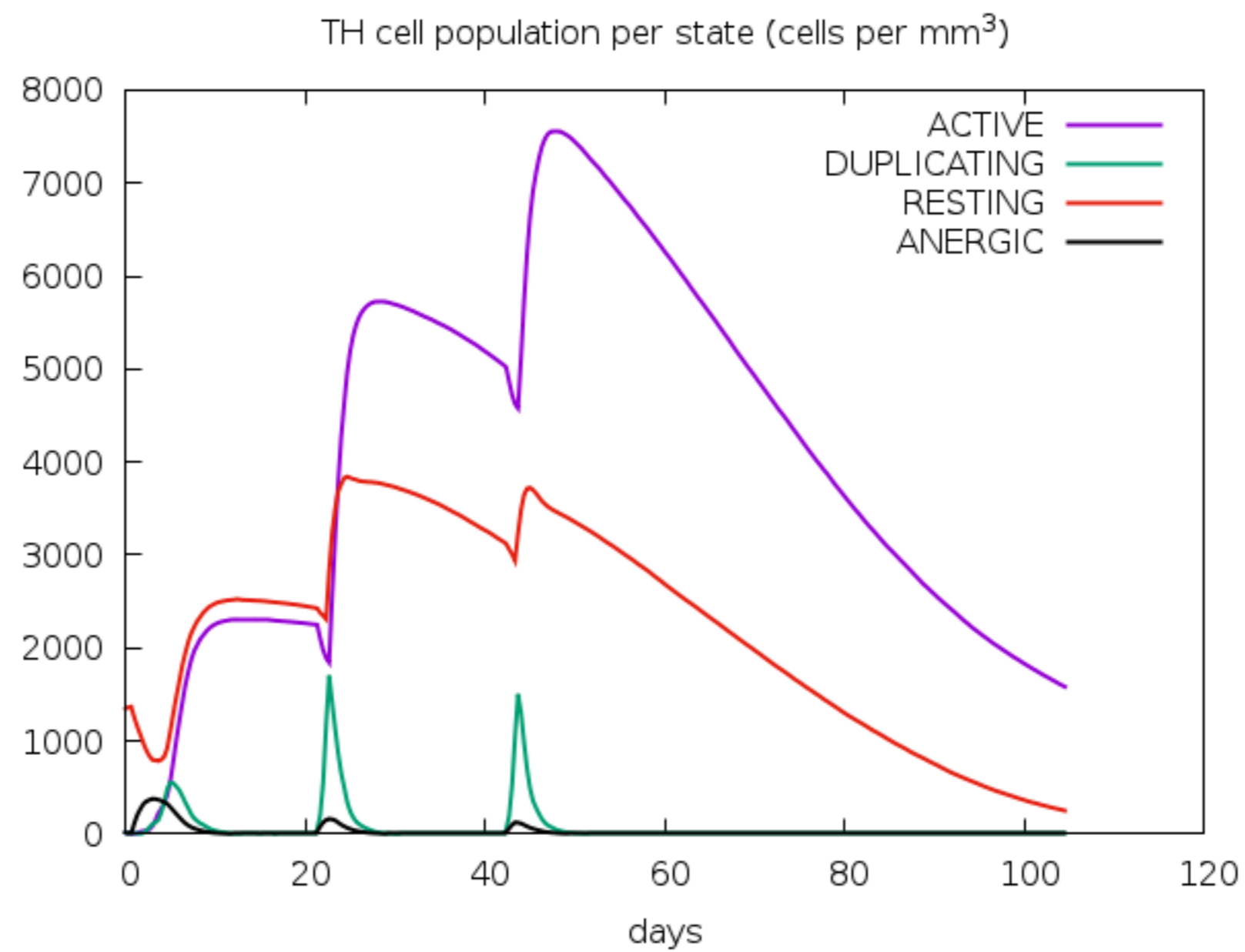

**E**

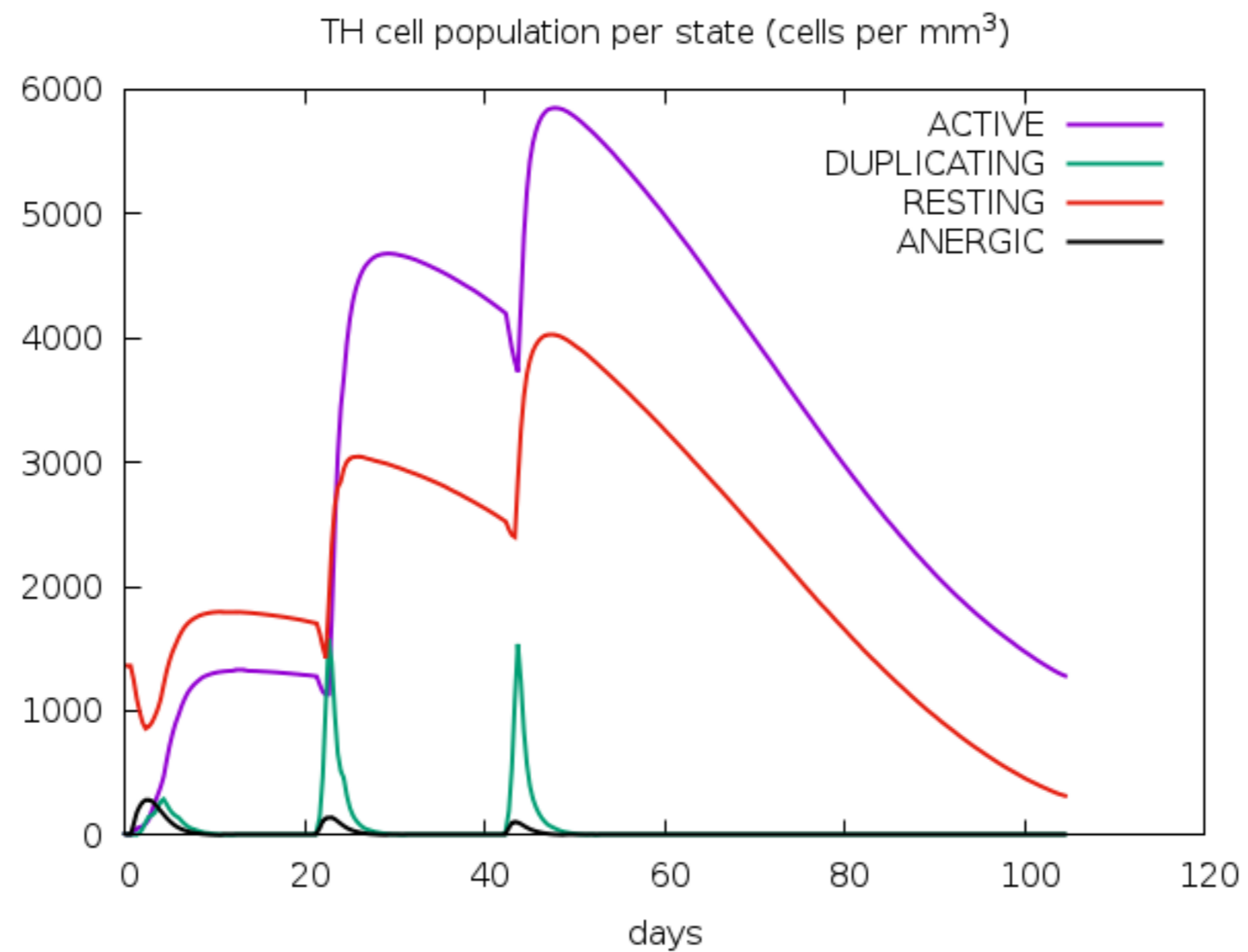

Supplement: Supplementary file 19 — Additional file 19: Figure S16. C-ImmSim prediction represented the immune profile of T- helper (TH) cell population state levels for the individual R. microplus Bm86 (A), AQP1 (B), AQP2 (C), and VgR (D) and cocktail proteins(E) as vaccine candidates. [file 13071_2025_7109_MOESM19_ESM.pdf]

A

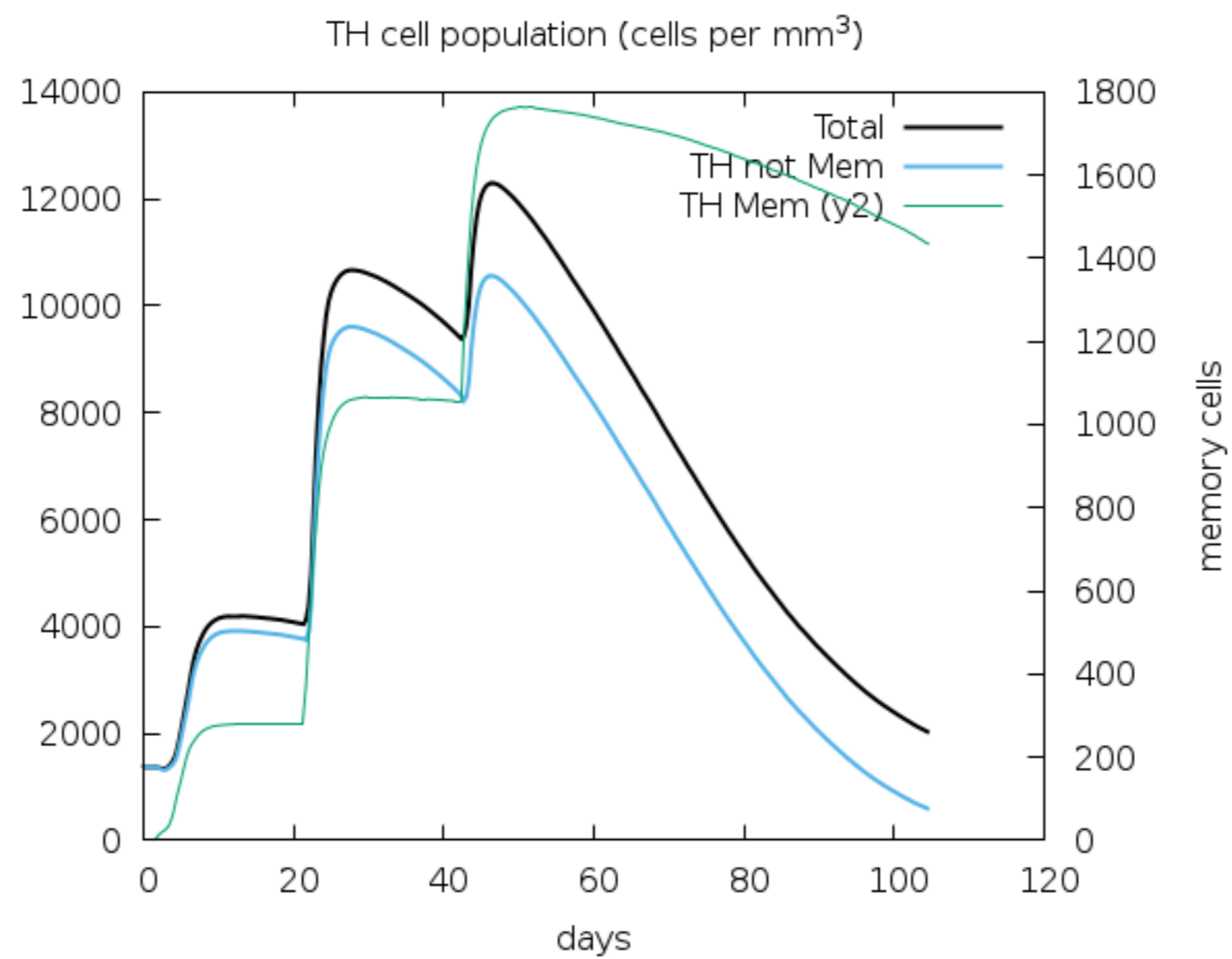

**B**

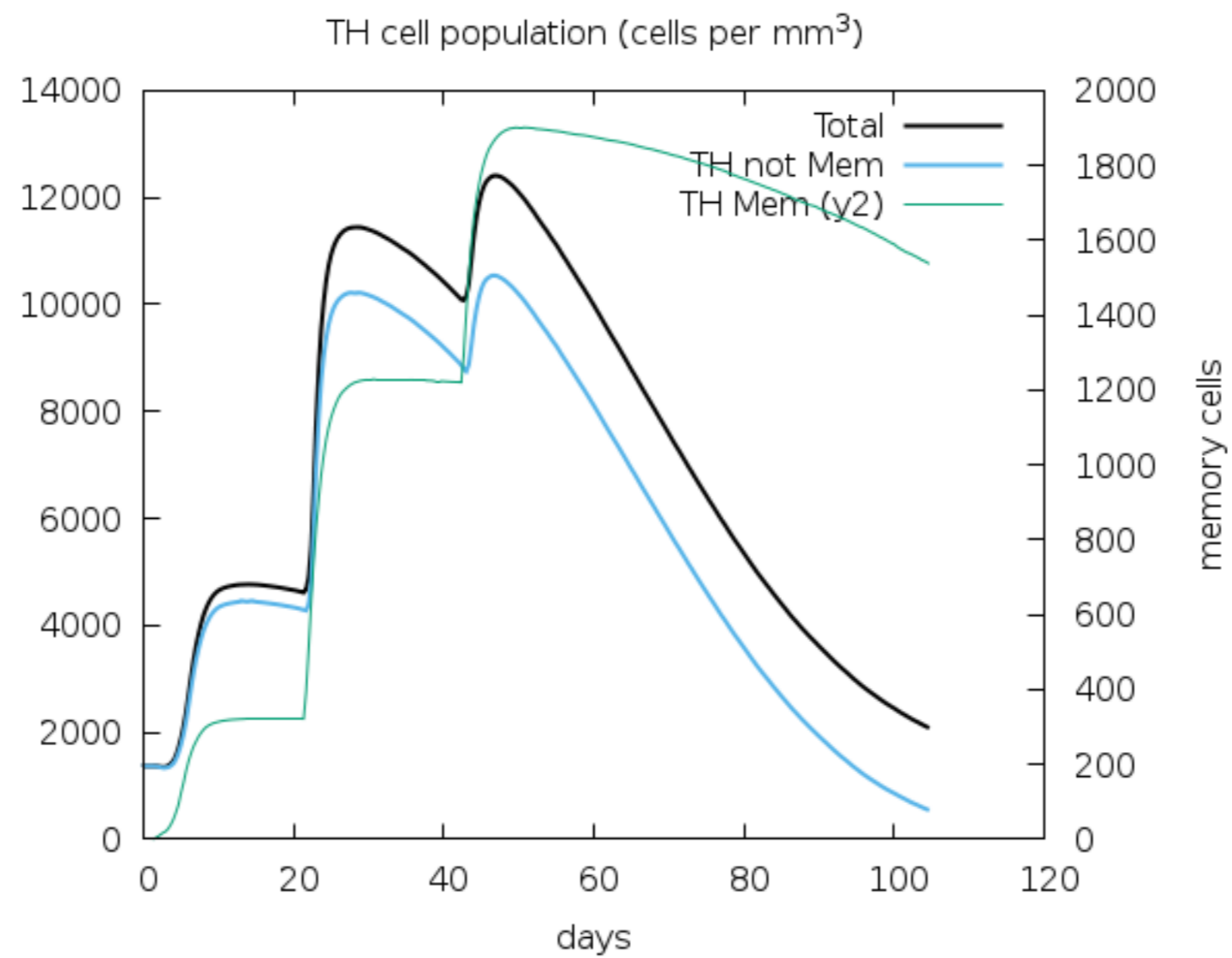

C

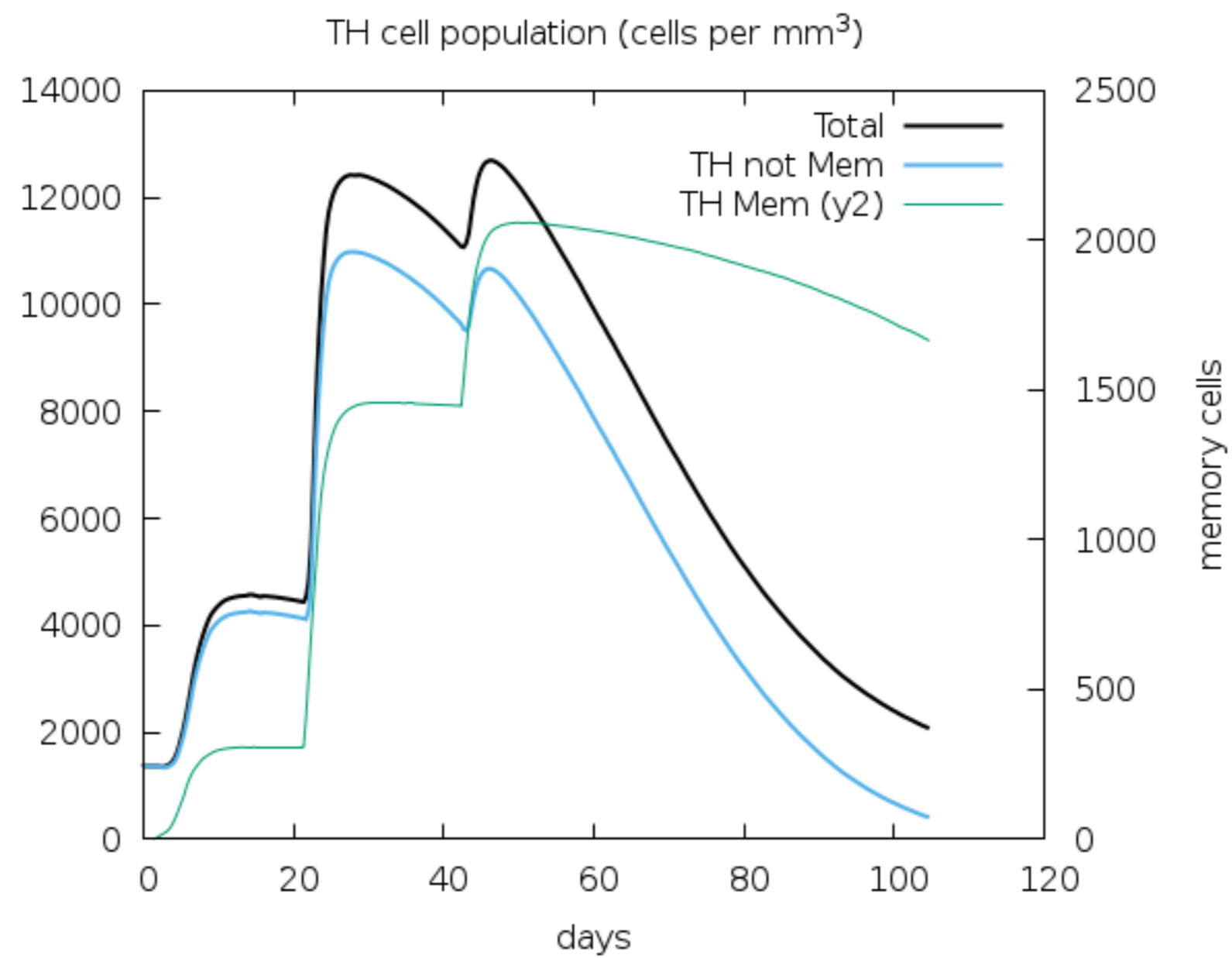

D

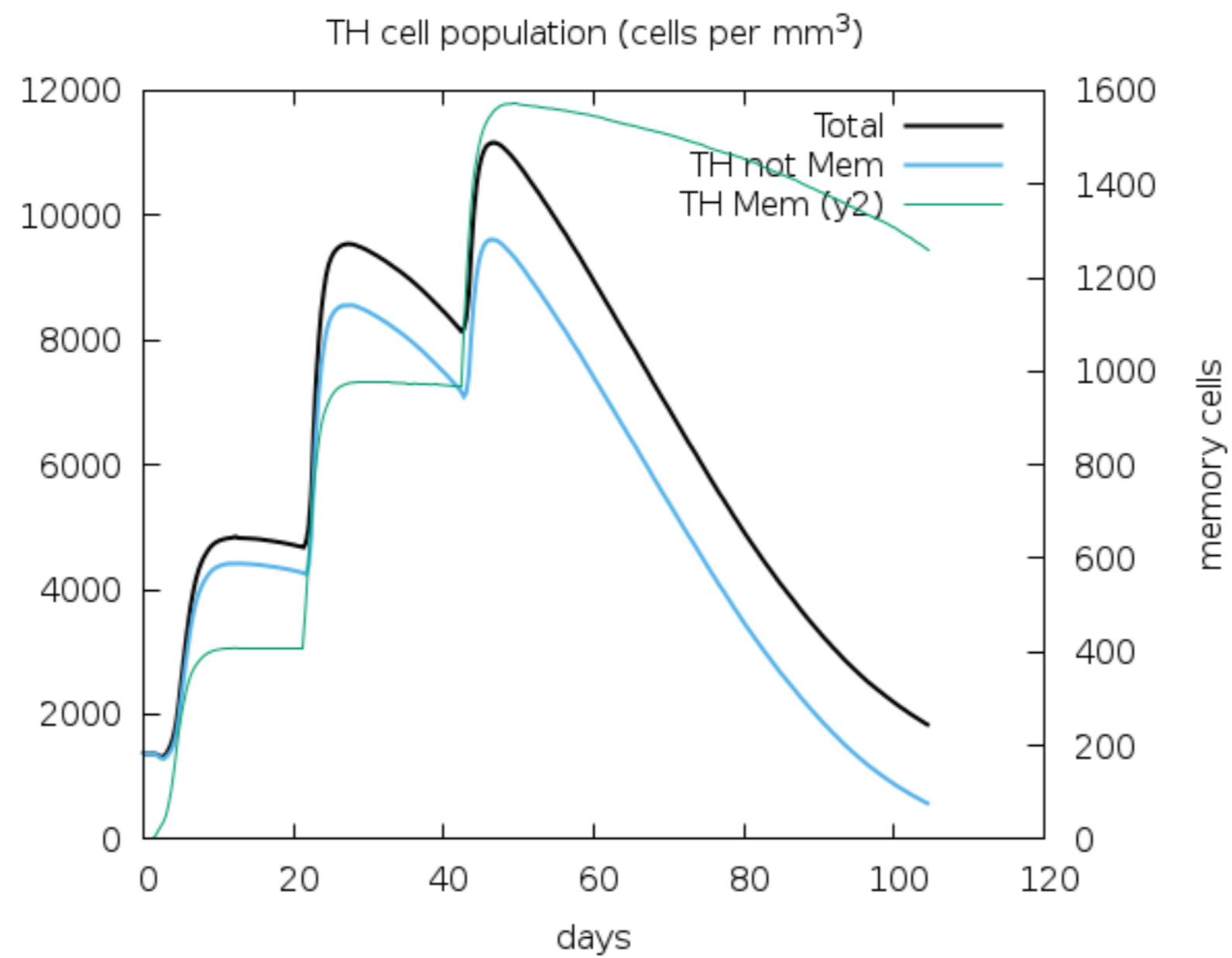

**E**

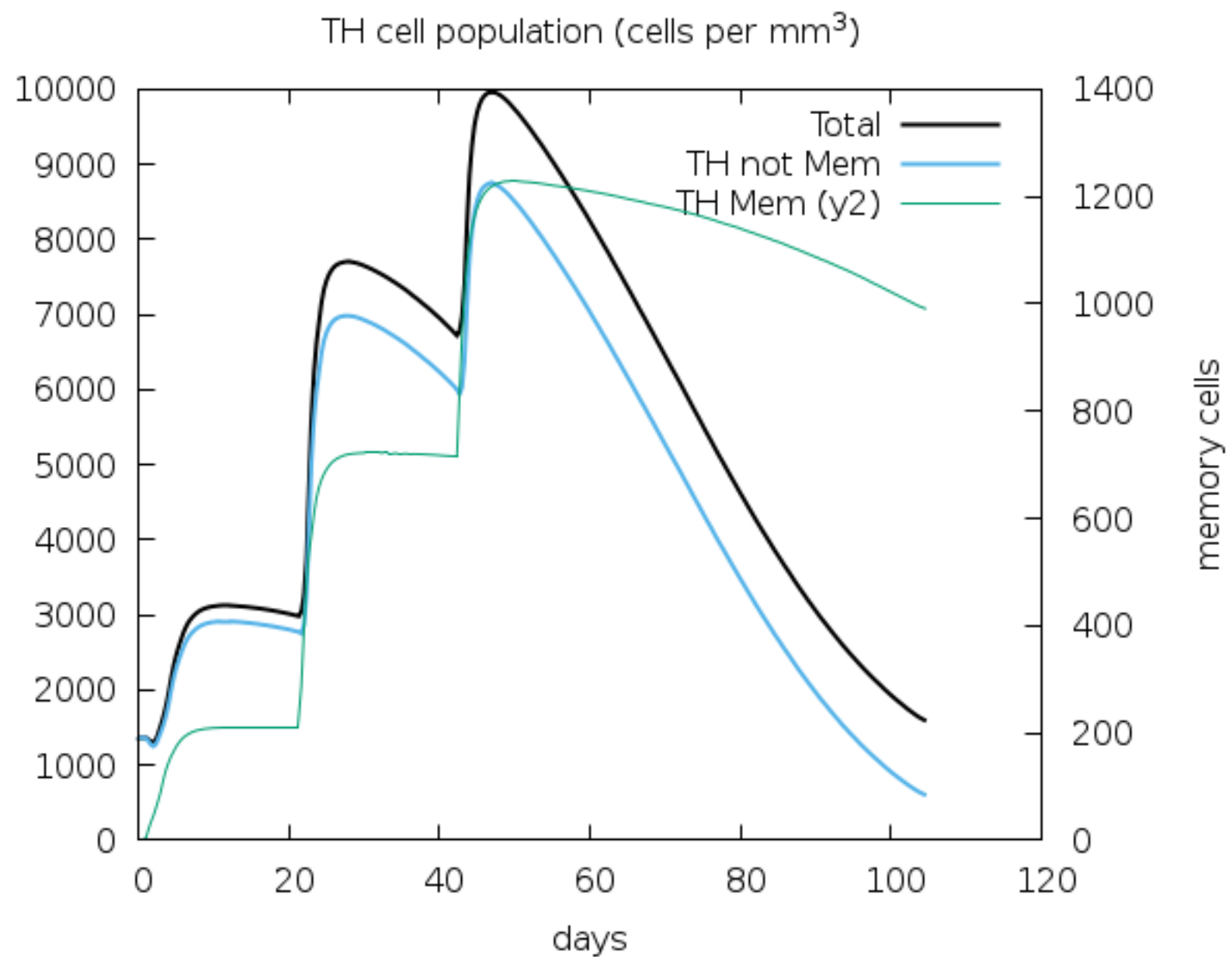

Supplement: Supplementary file 20 — Additional file 20: Figure S17. C-ImmSim prediction represented the immune profile of T- helper (TH) cell population levels for the individual R. microplus Bm86 (A), AQP1 (B), AQP2 (C), and VgR (D) and cocktail proteins(E) as vaccine candidates. [file 13071_2025_7109_MOESM20_ESM.pdf]

A

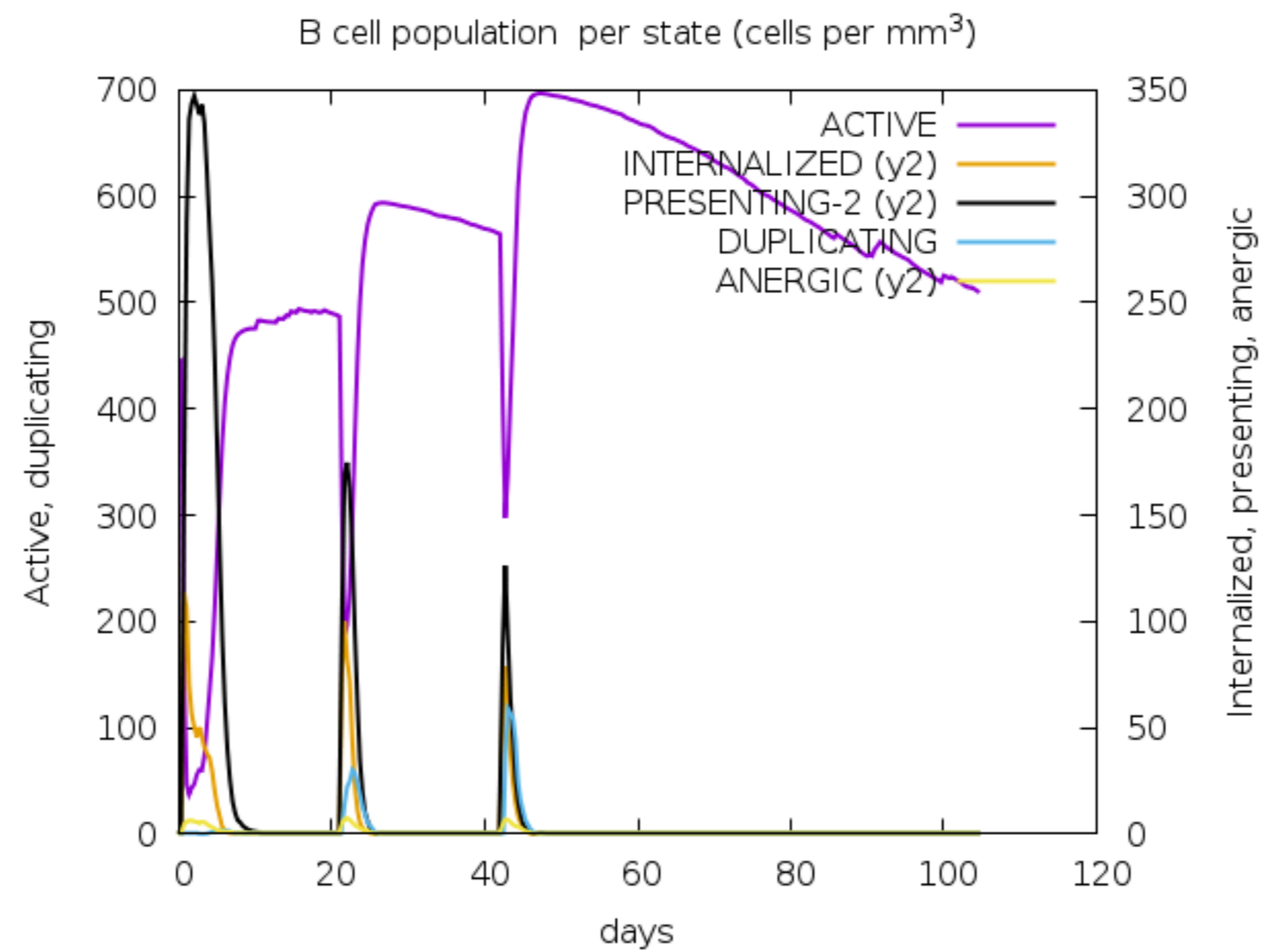

**B**

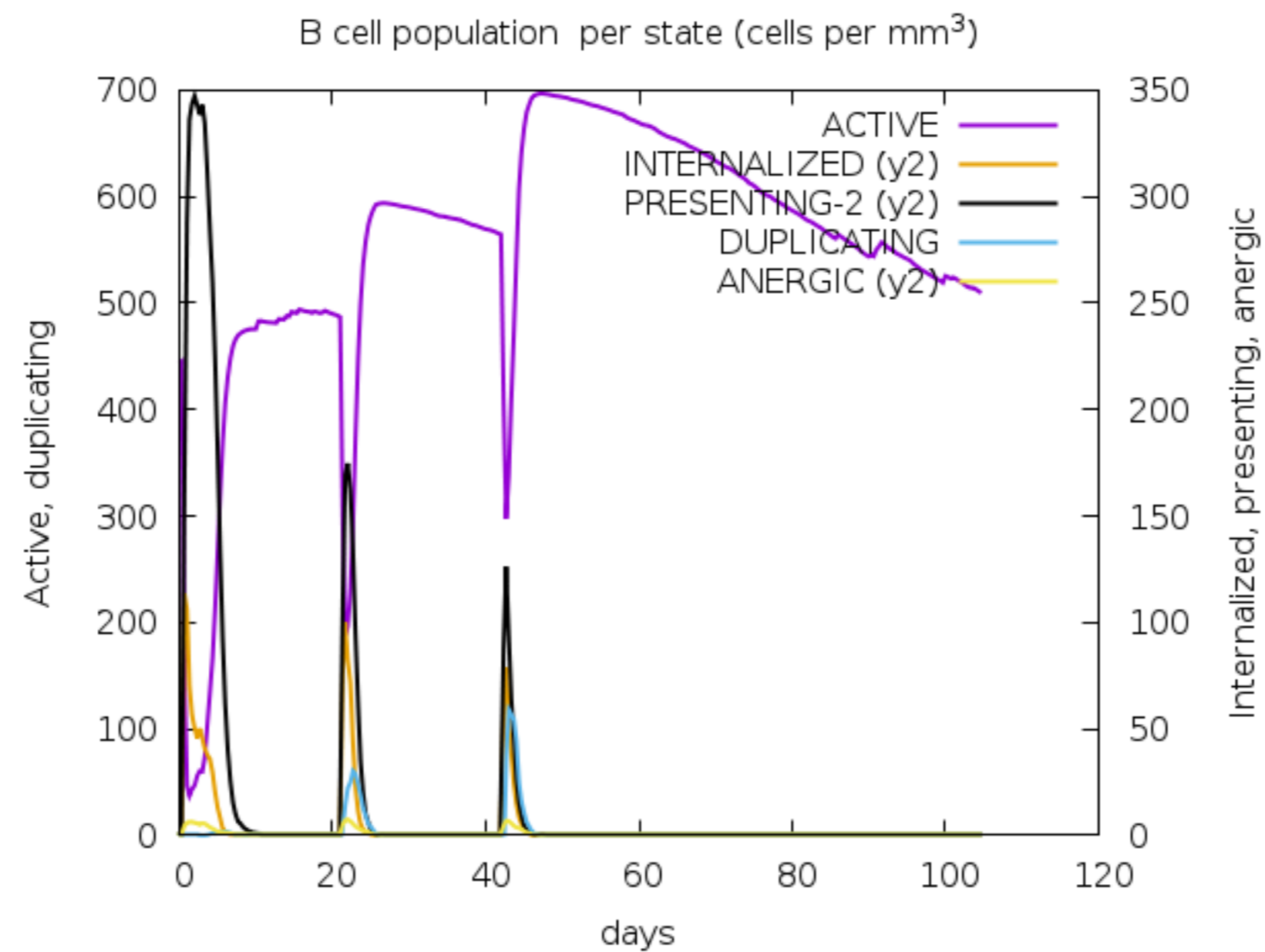

C

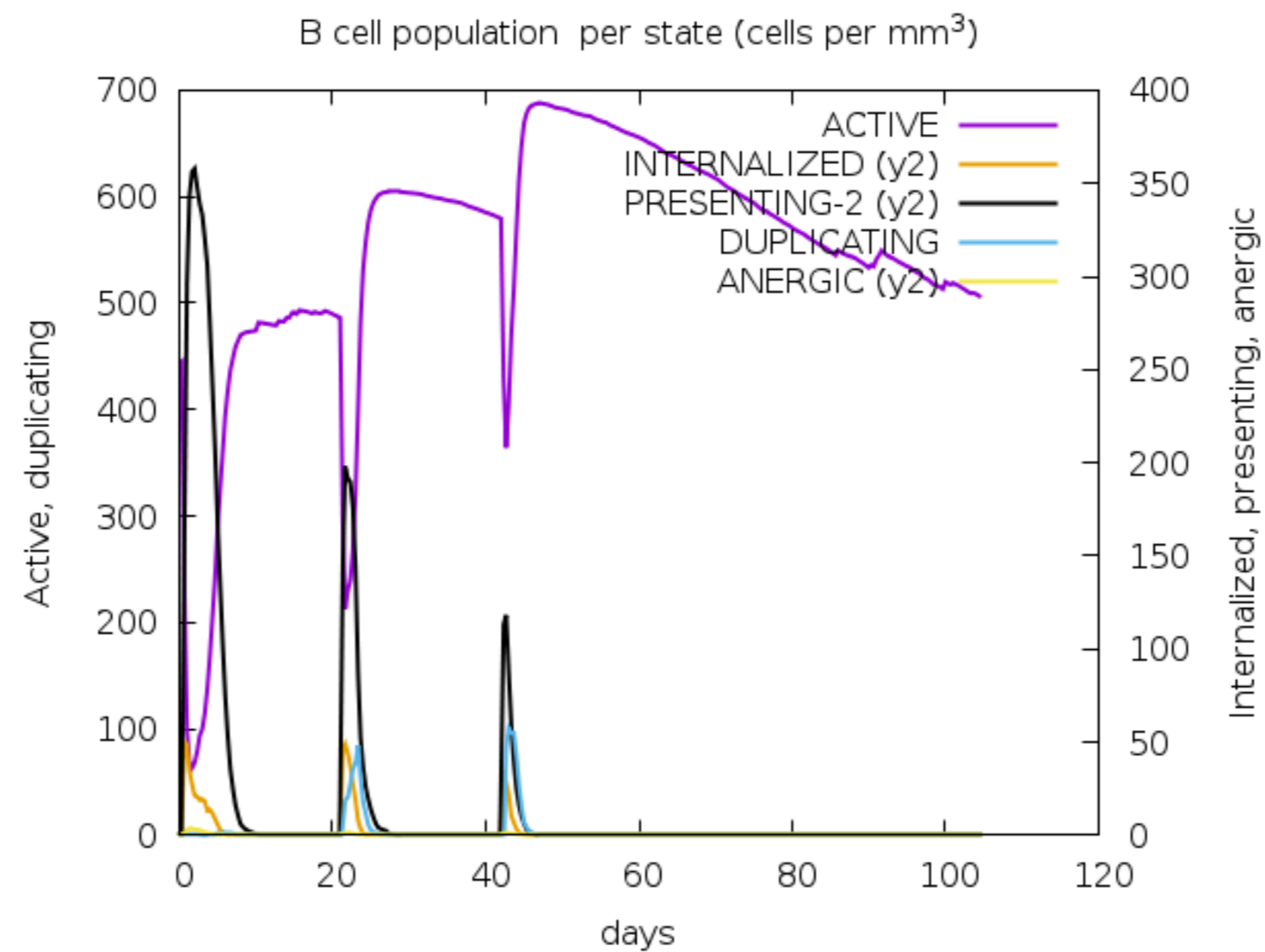

D

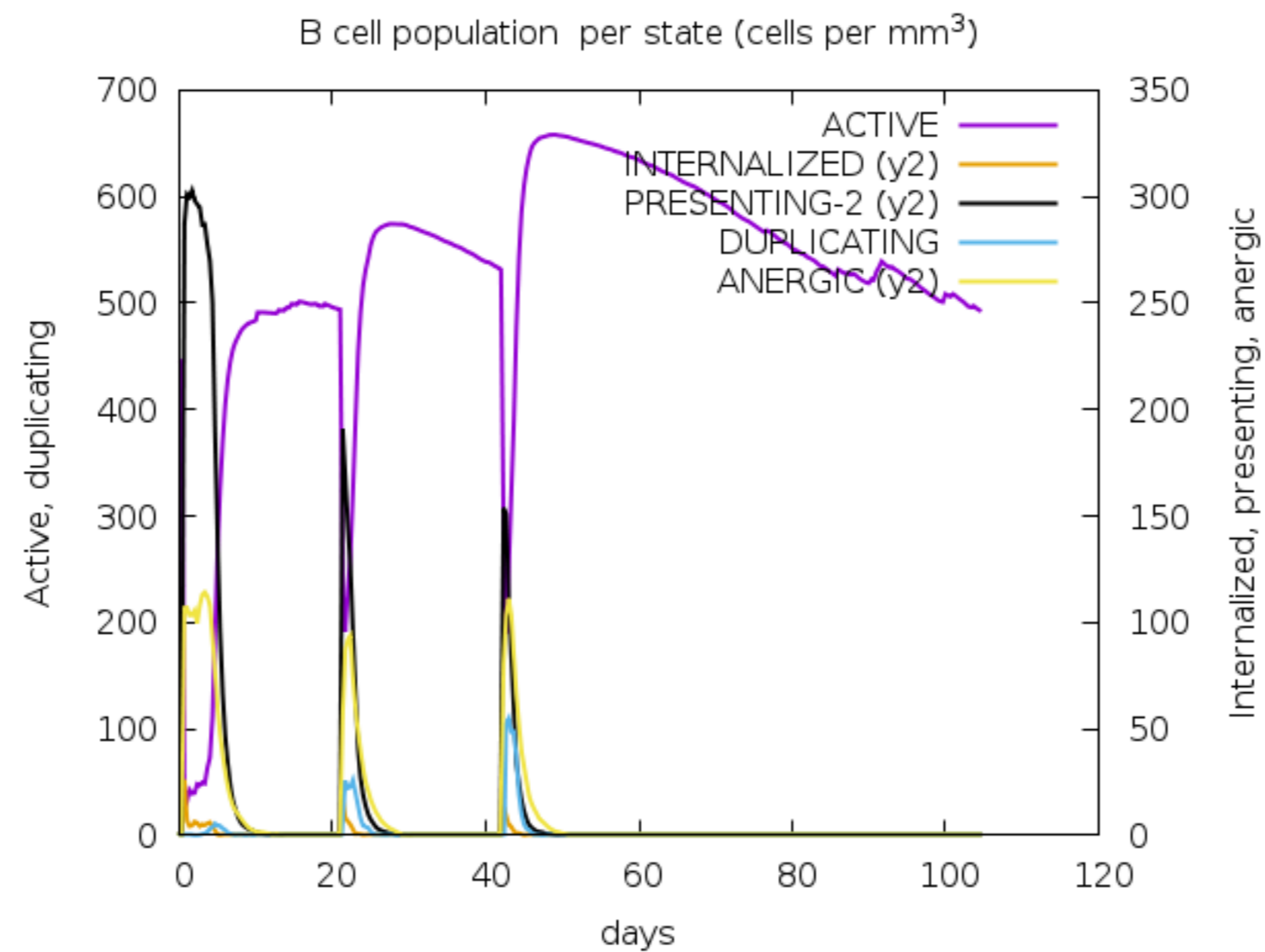

**E**

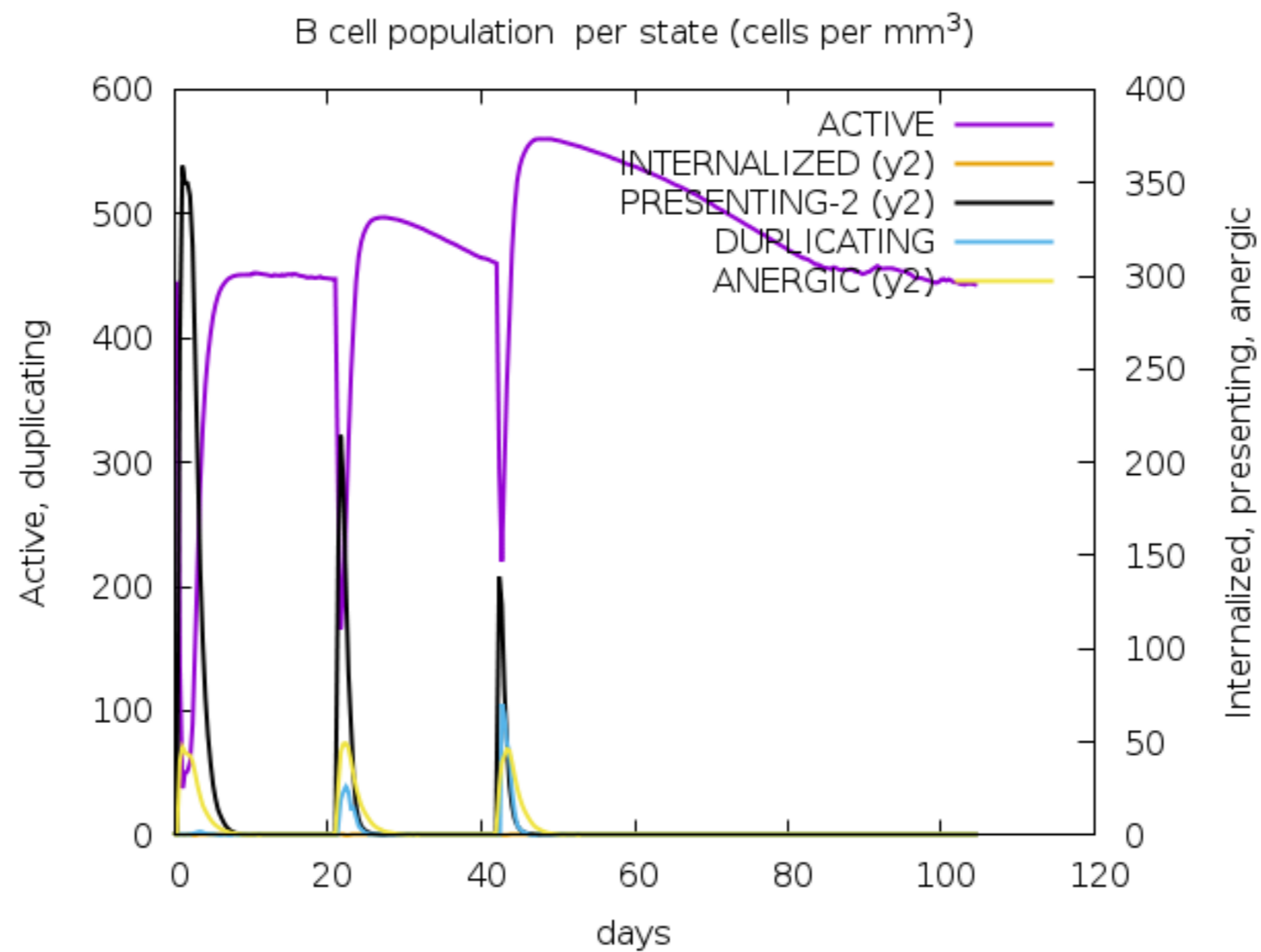

Supplement: Supplementary file 21 — Additional file 21: Figure S18. C-ImmSim prediction represented the immune profile of B-cell population state levels for the individual R. microplus Bm86 (A), AQP1 (B), AQP2 (C), and VgR (D) and cocktail proteins(E) as vaccine candidates. [file 13071_2025_7109_MOESM21_ESM.pdf]

A

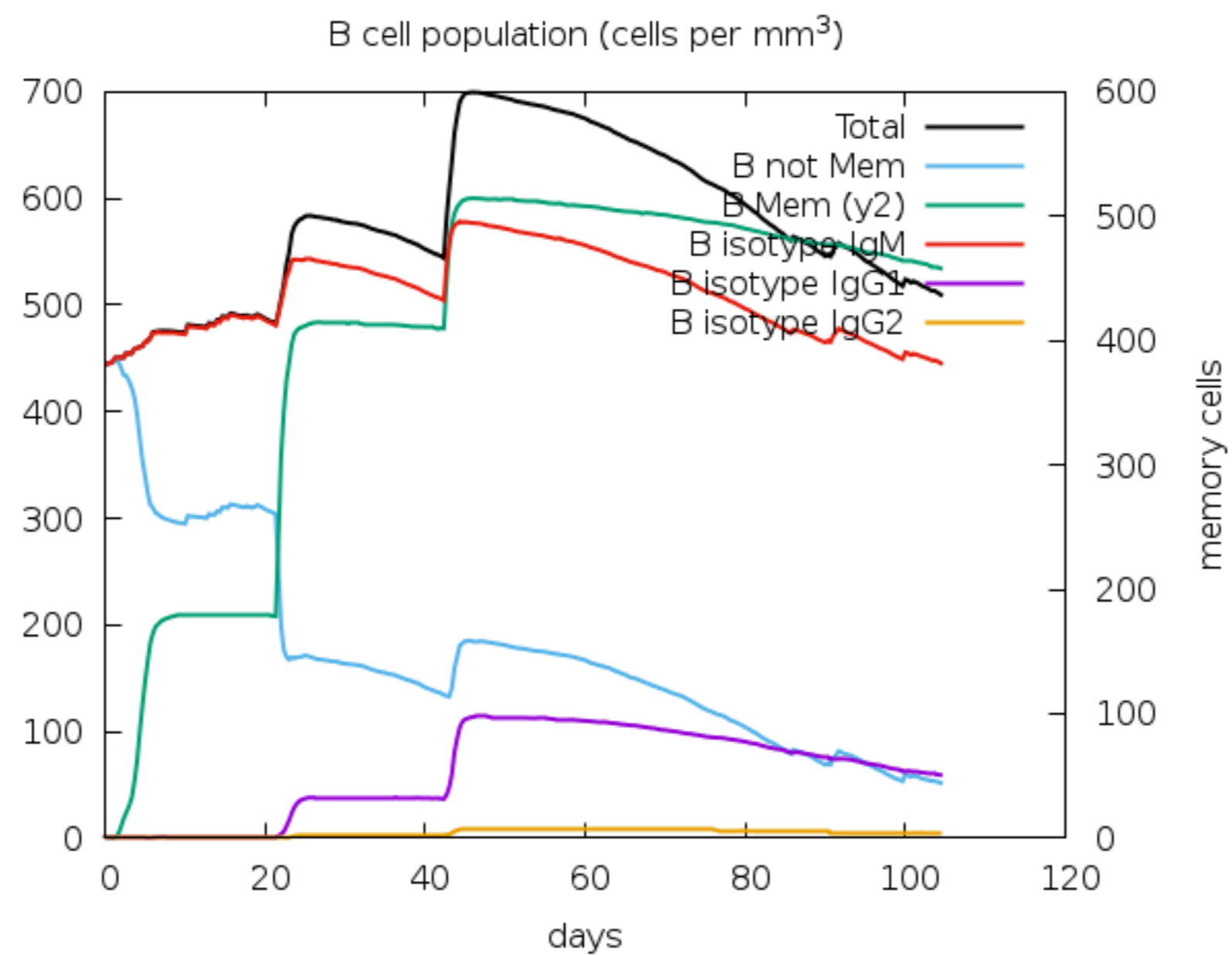

**B**

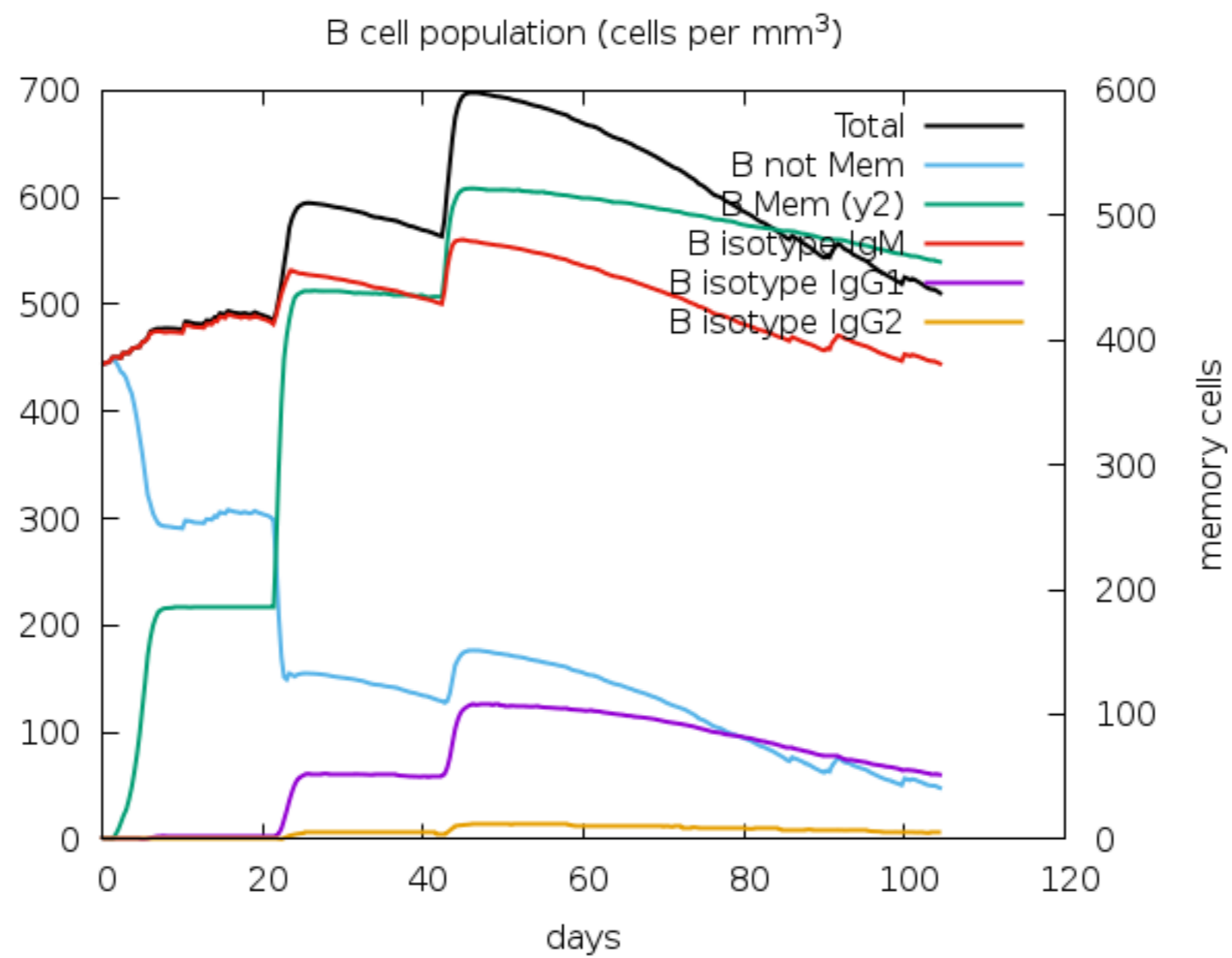

C

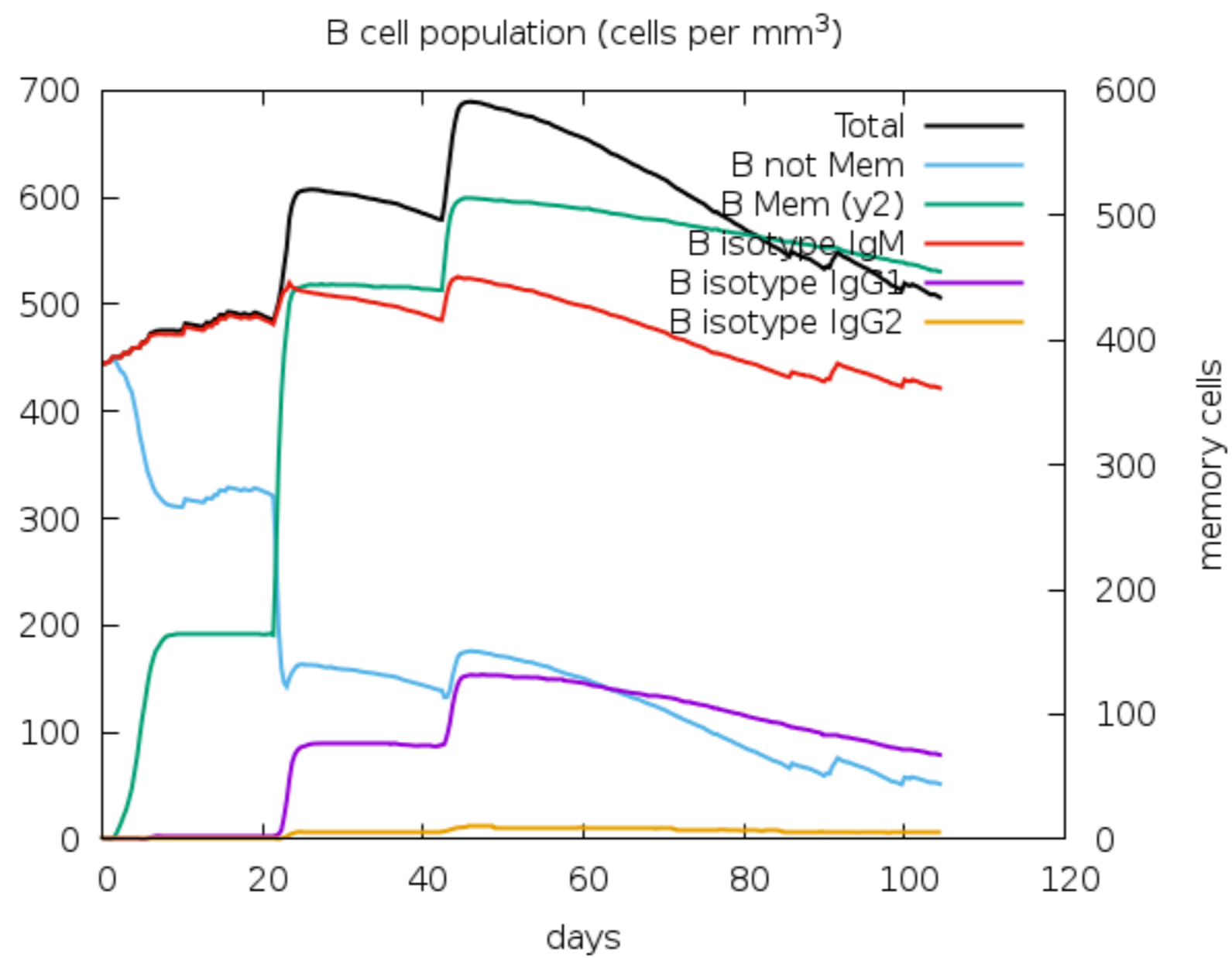

**D**

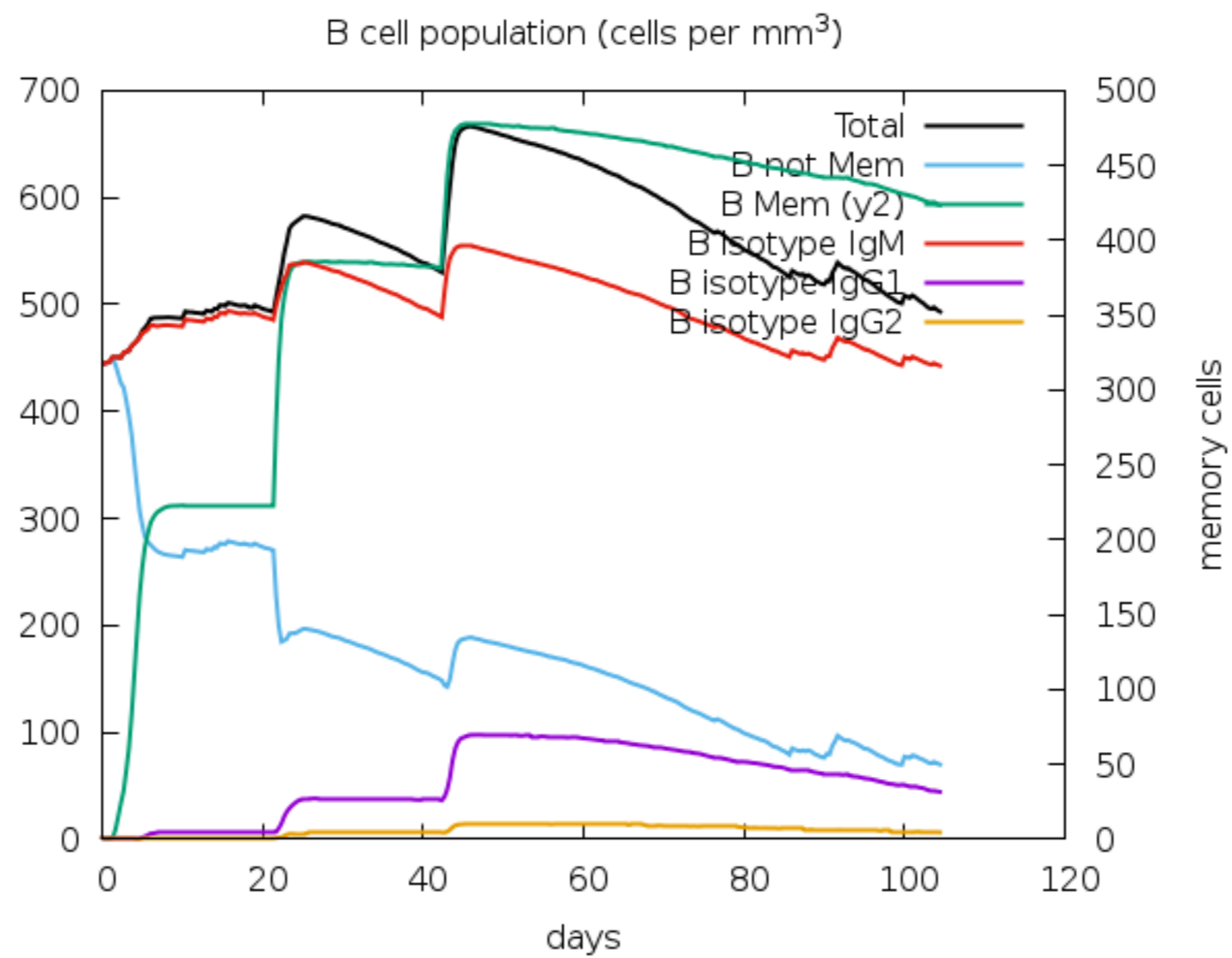

E

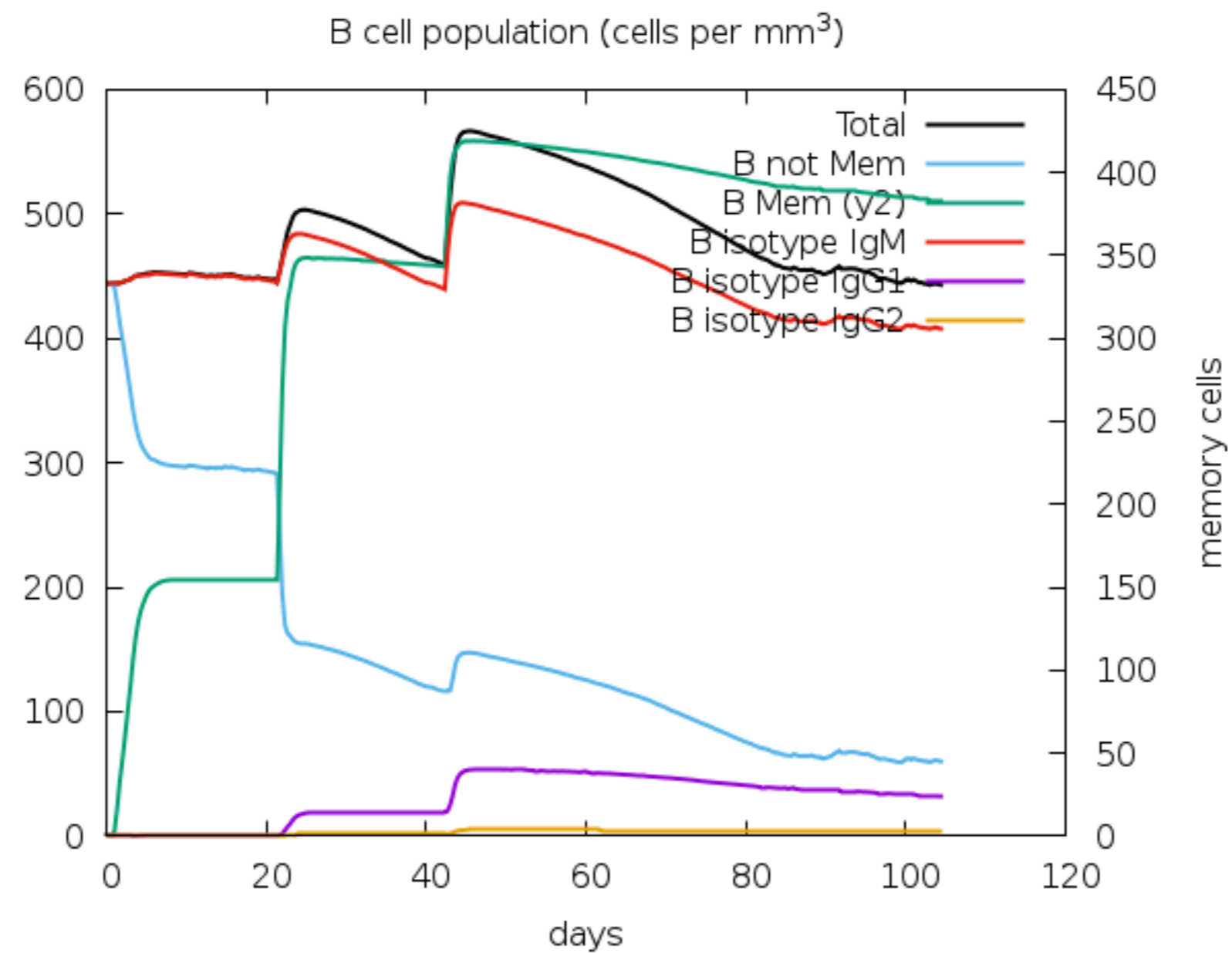

Supplement: Supplementary file 22 — Additional file 22: Figure S19. C-ImmSim prediction represented the immune profile of B-cell population levels for the individual R. microplus Bm86 (A), AQP1 (B), AQP2 (C), and VgR (D) and cocktail proteins(E) as vaccine candidates. [file 13071_2025_7109_MOESM22_ESM.pdf]
