# Supplementary material for: Comparative immunoinformatic analysis of Rhipicephalus microplus cocktail vaccine targets
Source: Parasit Vectors. 2025 Dec 9;18:502. doi: 10.1186/s13071-025-07109-y (PMC12690872; doi:10.1186/s13071-025-07109-y)
Supplement: Supplementary file 2 — Additional file 2: Figure S2. Signal Peptide prediction of vaccine target R. microplus proteins (Bm86, AQP1, AQP2, and VgR). [file 13071_2025_7109_MOESM2_ESM.pdf]

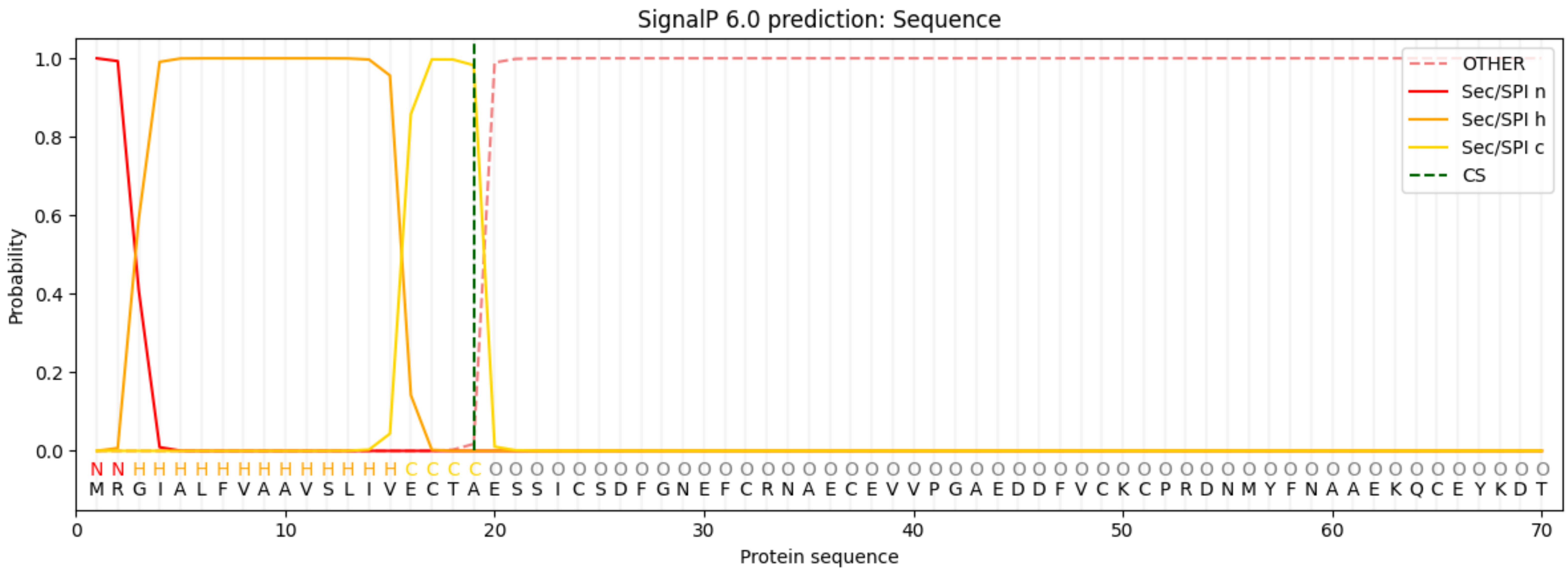
SignalP 6.0 prediction: Sequence
OTHER
Sec/SPI n
Sec/SPI h
Sec/SPI c
CS
Probability
Protein sequence
N N H H H H H H H H H H H H H C C C C O O O O O O O O O O O O O O O O O O O O O O O O O O O O O O O O O O O O O O O O O O O O O O O O O O
M R G I A L F V A A V S L I V E C T A E S S I C S D F G N E F C R N A E C E V V P G A E D D F V C K C P R D N M Y F N A A E K Q C E Y K D T

BM86

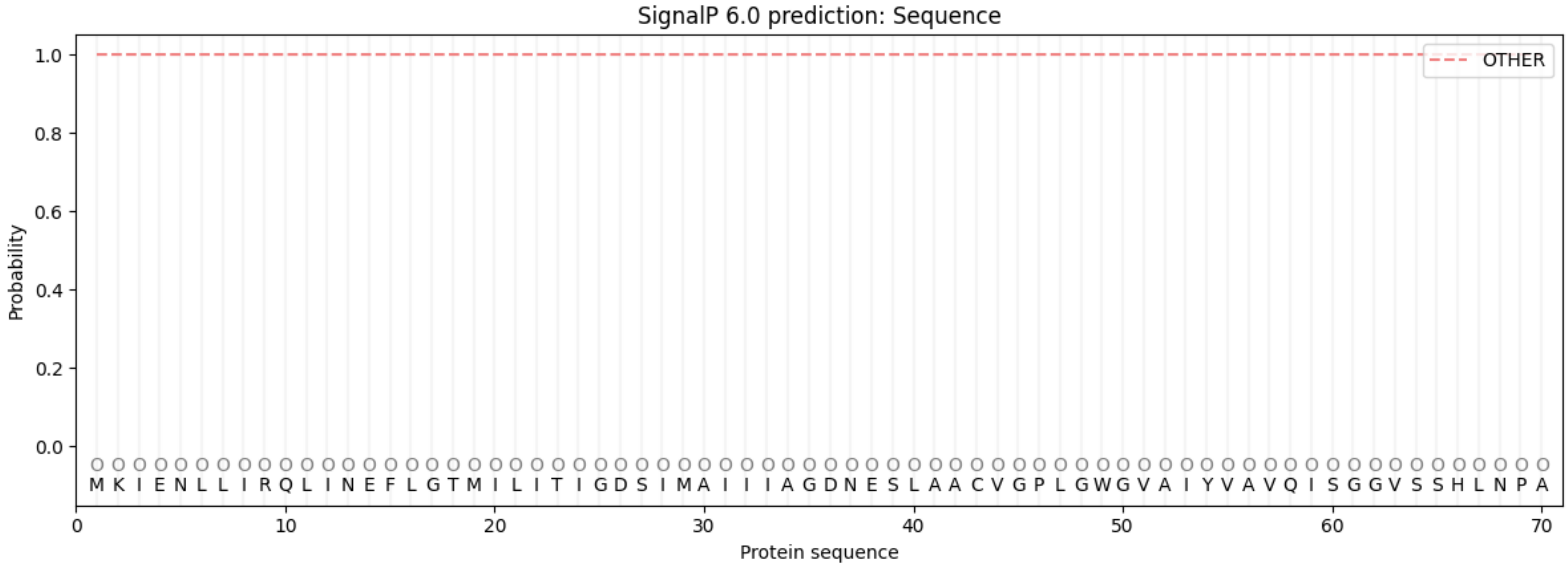
SignalP 6.0 prediction: Sequence
OTHER
Probability
1.0
0.8
0.6
0.4
0.2
0.0
M K I E N L L I R Q L I N E F L G T M I L I T I G D S I M A I I I A G D N E S L A A C V G P L G W G V A I Y V A V Q I S G G V S S H L N P A
0
10
20
30
40
50
60
70
Protein sequence

AQP1

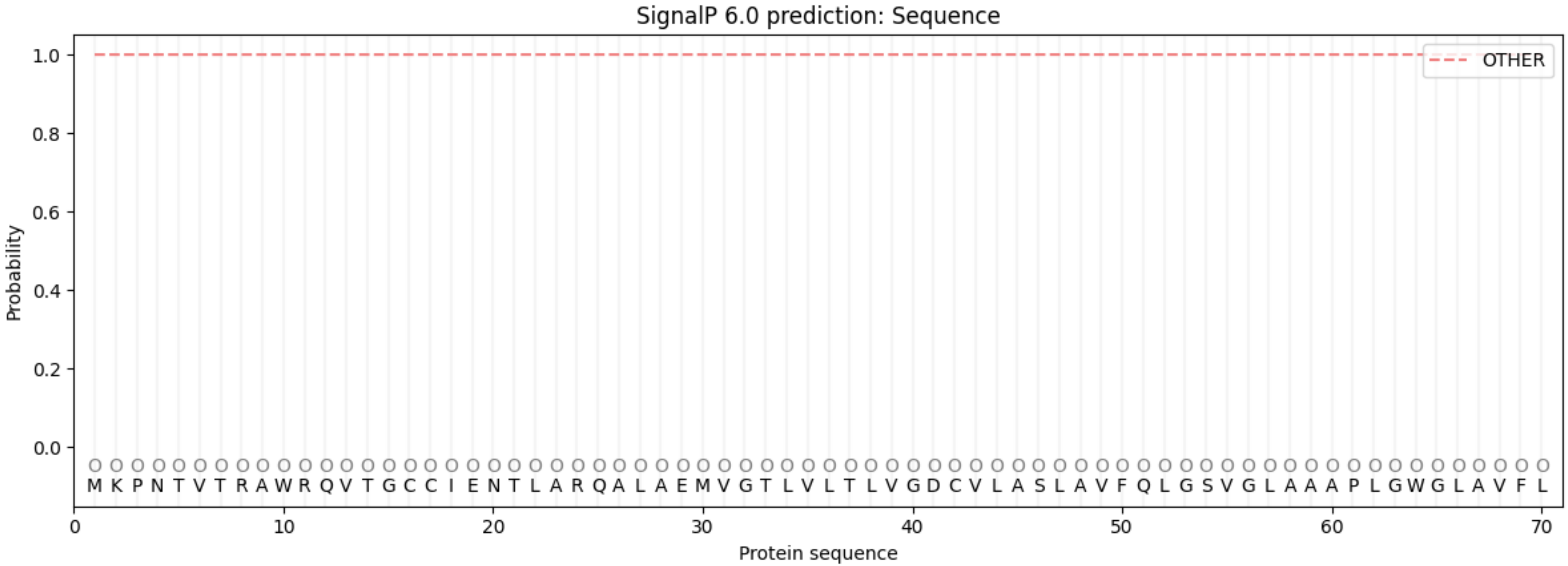

SignalP 6.0 prediction: Sequence
OTHER
Probability
Protein sequence
M K P N T V T R A W R Q V T G C C I E N T L A R Q A L A E M V G T L V L T L V G D C V L A S L A V F Q L G S V G L A A A P L G W G L A V F L

AQP2

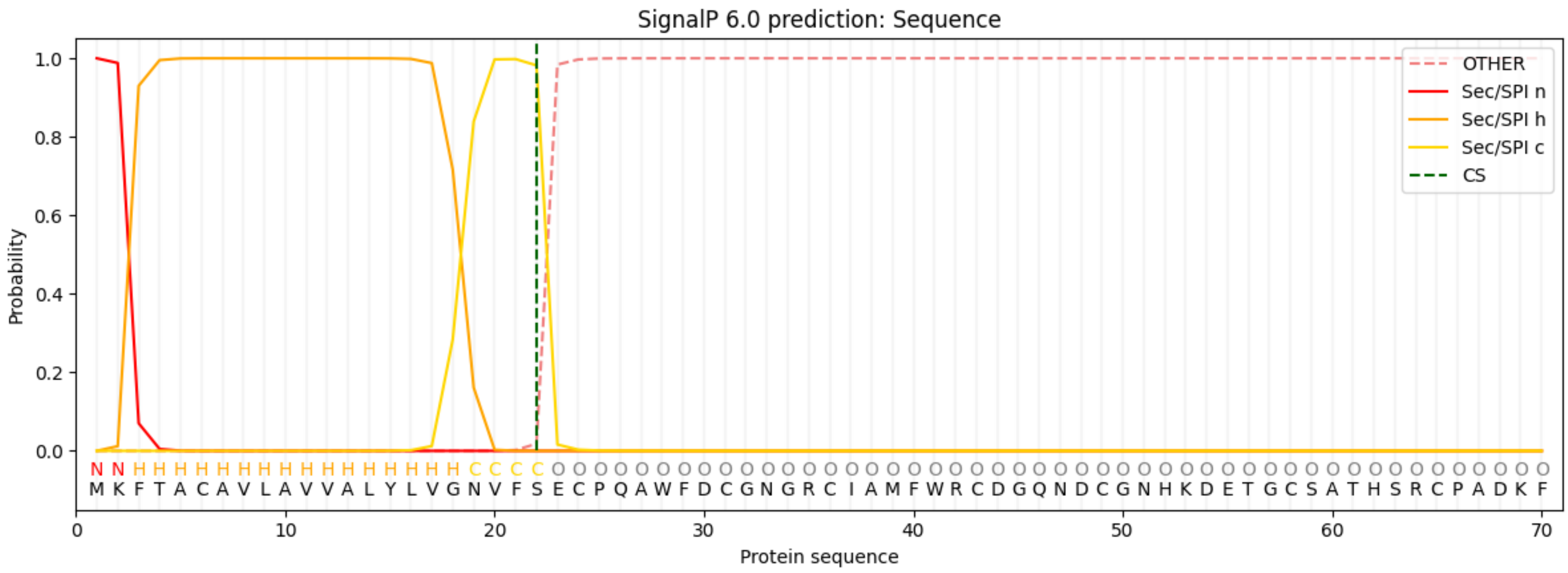

SignalP 6.0 prediction: Sequence
Probability
Protein sequence
OTHER
Sec/SPI n
Sec/SPI h
Sec/SPI c
CS
N N H H H H H H H H H H H H H H H H C C C C O O O O O O O O O O O O O O O O O O O O O O O O O O O O O O O O O O O O O O O O O O O O O O O O
M K F T A C A V L A V V A L Y L V G N V F S E C P Q A W F D C G N G R C I A M F W R C D G Q N D C G N H K D E T G C S A T H S R C P A D K F

VgR
