# Supplementary material for: Comparative immunoinformatic analysis of Rhipicephalus microplus cocktail vaccine targets
Source: Parasit Vectors. 2025 Dec 9;18:502. doi: 10.1186/s13071-025-07109-y (PMC12690872; doi:10.1186/s13071-025-07109-y)
Supplement: Supplementary file 3 — Additional file 3: Figure S3. GPI Anchor prediction of vaccine target R. microplus proteins (Bm86, AQP1, AQP2, and VgR). [file 13071_2025_7109_MOESM3_ESM.pdf]

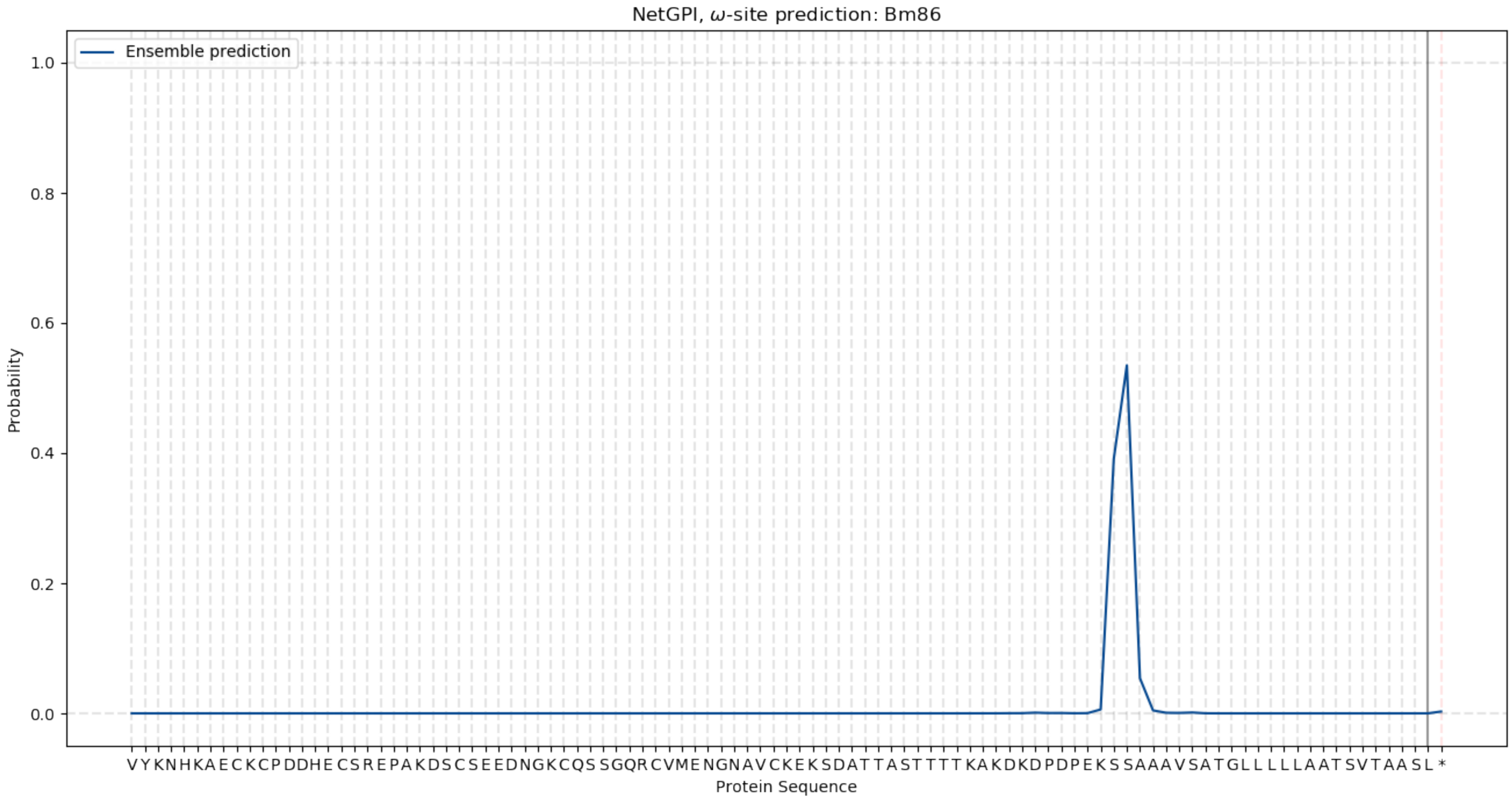
NetGPI, ω-site prediction: Bm86
Ensemble prediction
Probability
1.0
0.8
0.6
0.4
0.2
0.0
V Y K N H K A E C K C P D D H E C S R E P A K D S C S E E D N G K C Q S S G Q R C V M E N G N A V C K E K S D A T T A S T T T T K A K D K D P D P E K S S A A A V S A T G L L L L L A A T S V T A A S L *
Protein Sequence

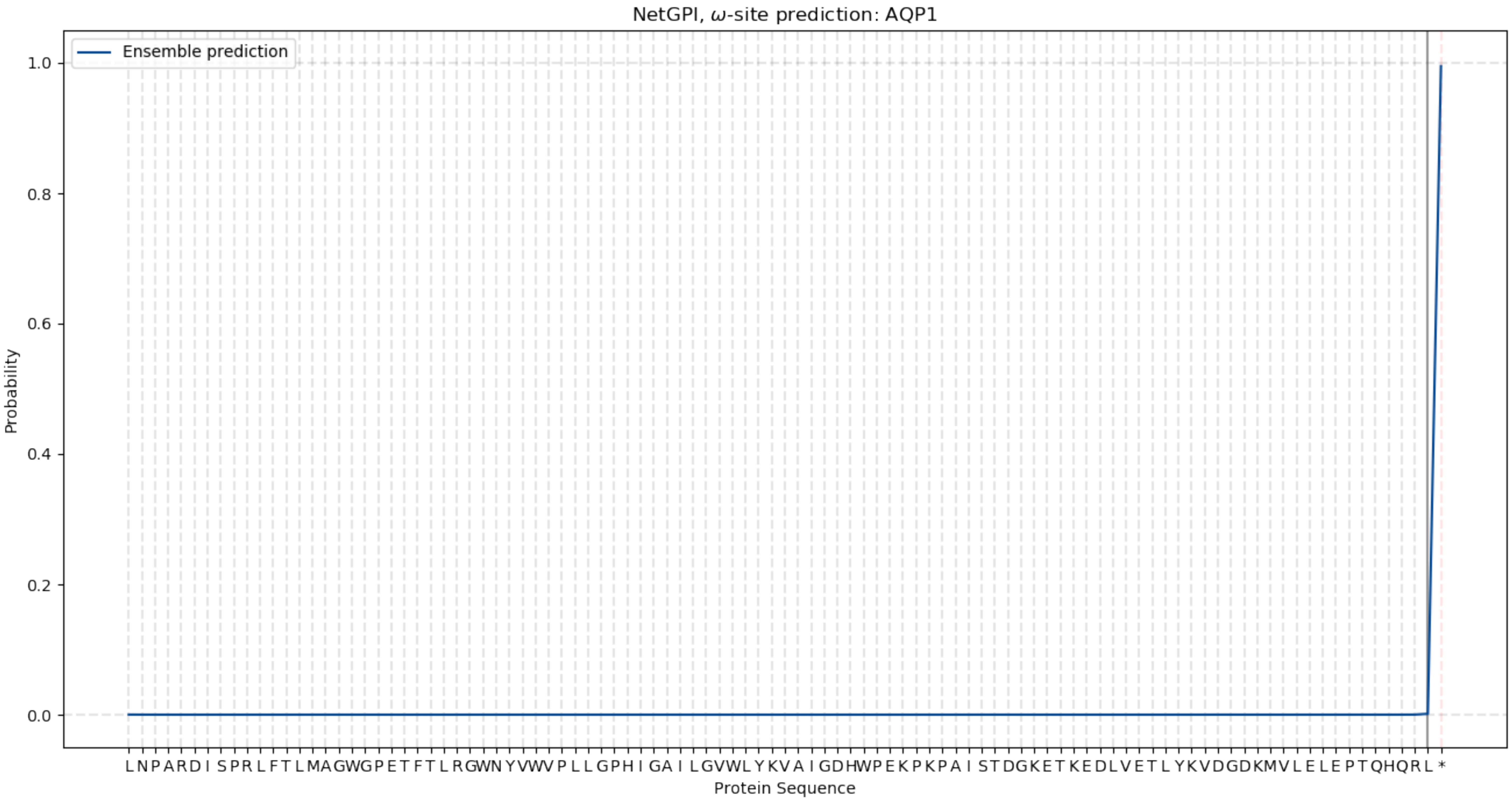

NetGPI, ω-site prediction: AQP1
Ensemble prediction
Probability
1.0
0.8
0.6
0.4
0.2
0.0
L N P A R D I S P R L F T L M A G W G P E T F T L R G W N Y V W V P L L G P H I G A I L G V W L Y K V A I G D H W P E K P K P A I S T D G K E T K E D L V E T L Y K V D G D K M V L E L E P T Q H Q R L *
Protein Sequence

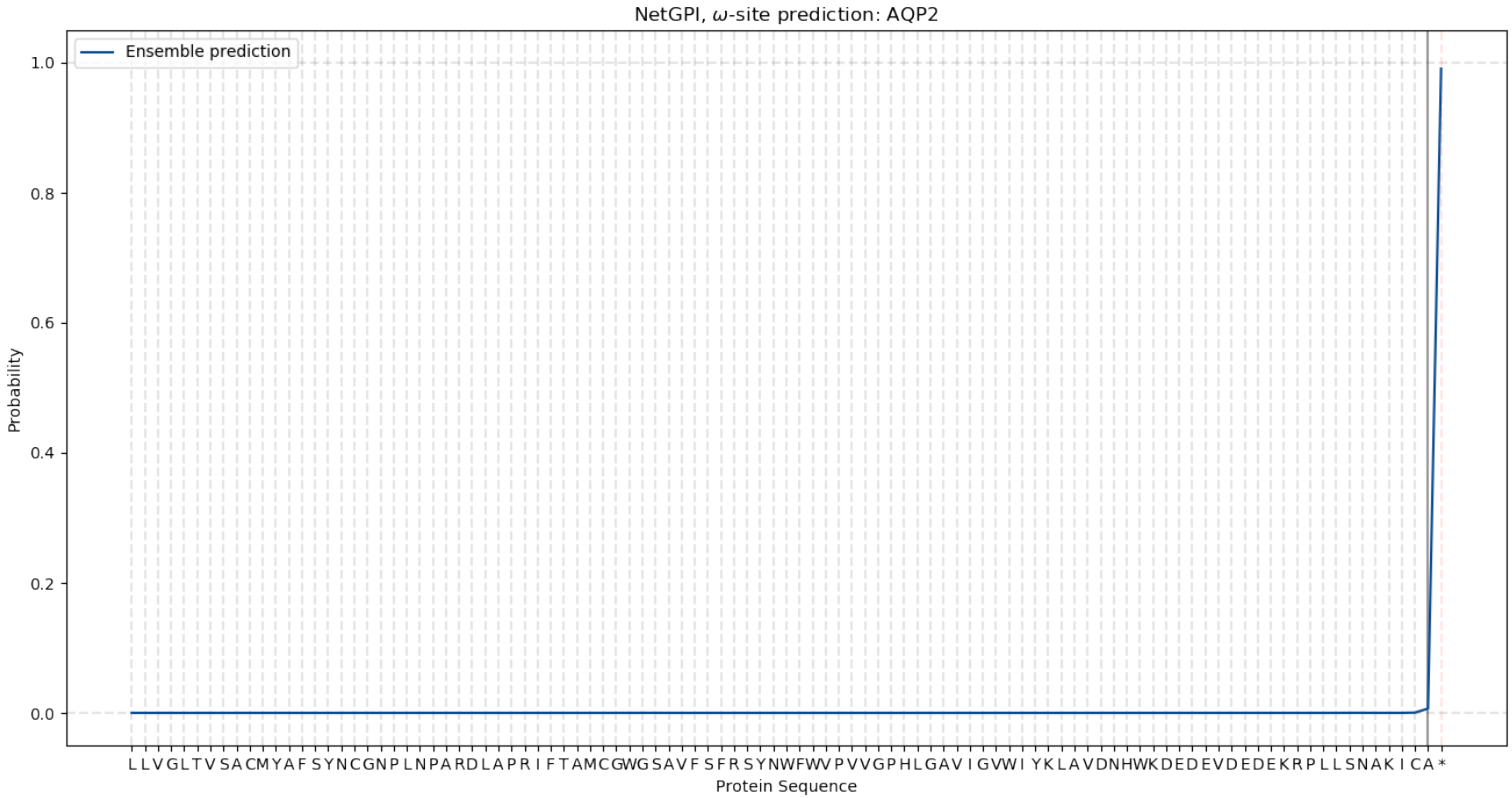
NetGPI, ω-site prediction: AQP2
Ensemble prediction
Probability
1.0
0.8
0.6
0.4
0.2
0.0
L L V G L T V S A C M Y A F S Y N C G N P L N P A R D L A P R I F T A M C G W G S A V F S F R S Y N W F W V P V V G P H L G A V I G V W I Y K L A V D N H W K D E D E V D E D E K R P L L S N A K I C A *
Protein Sequence

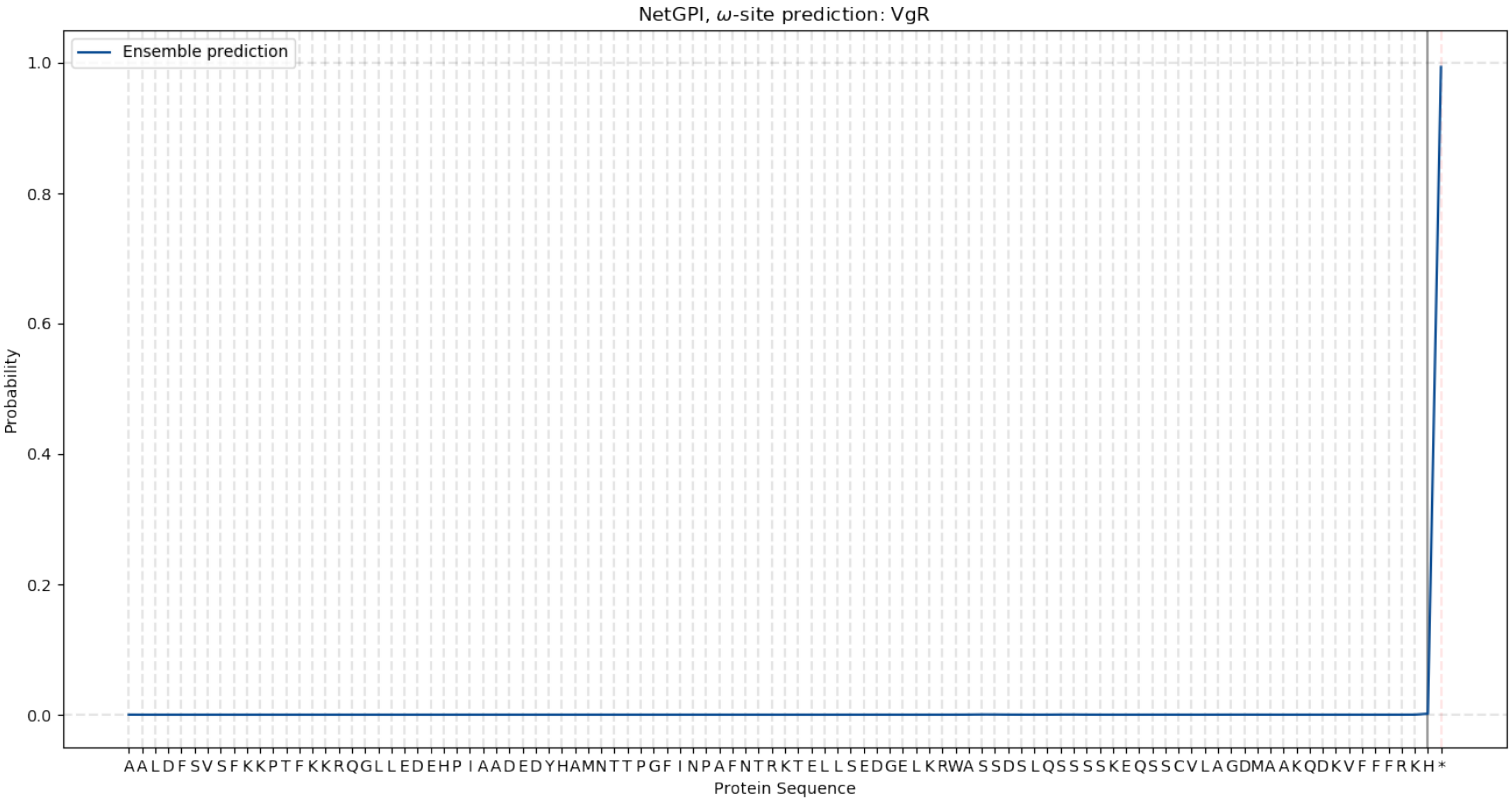
NetGPI, ω-site prediction: VgR
Ensemble prediction
Probability
1.0
0.8
0.6
0.4
0.2
0.0
AALDFSVSFKKPTFKKRQGLLEDEHPIAADEDYHAMNTTPGFINPAFNTRKTELLSEDGELKRWASSDSLQSSSSKEQSSCVLAGDMAAKQDKVFFFRKH*
Protein Sequence
