# Supplementary material for: Comparative immunoinformatic analysis of Rhipicephalus microplus cocktail vaccine targets
Source: Parasit Vectors. 2025 Dec 9;18:502. doi: 10.1186/s13071-025-07109-y (PMC12690872; doi:10.1186/s13071-025-07109-y)
Supplement: Supplementary file 5 — Additional file 5: Figure S5. N-glycosylation sites prediction of vaccine target R. microplus proteins (Bm86, AQP1, AQP2, and VgR). [file 13071_2025_7109_MOESM5_ESM.pdf]

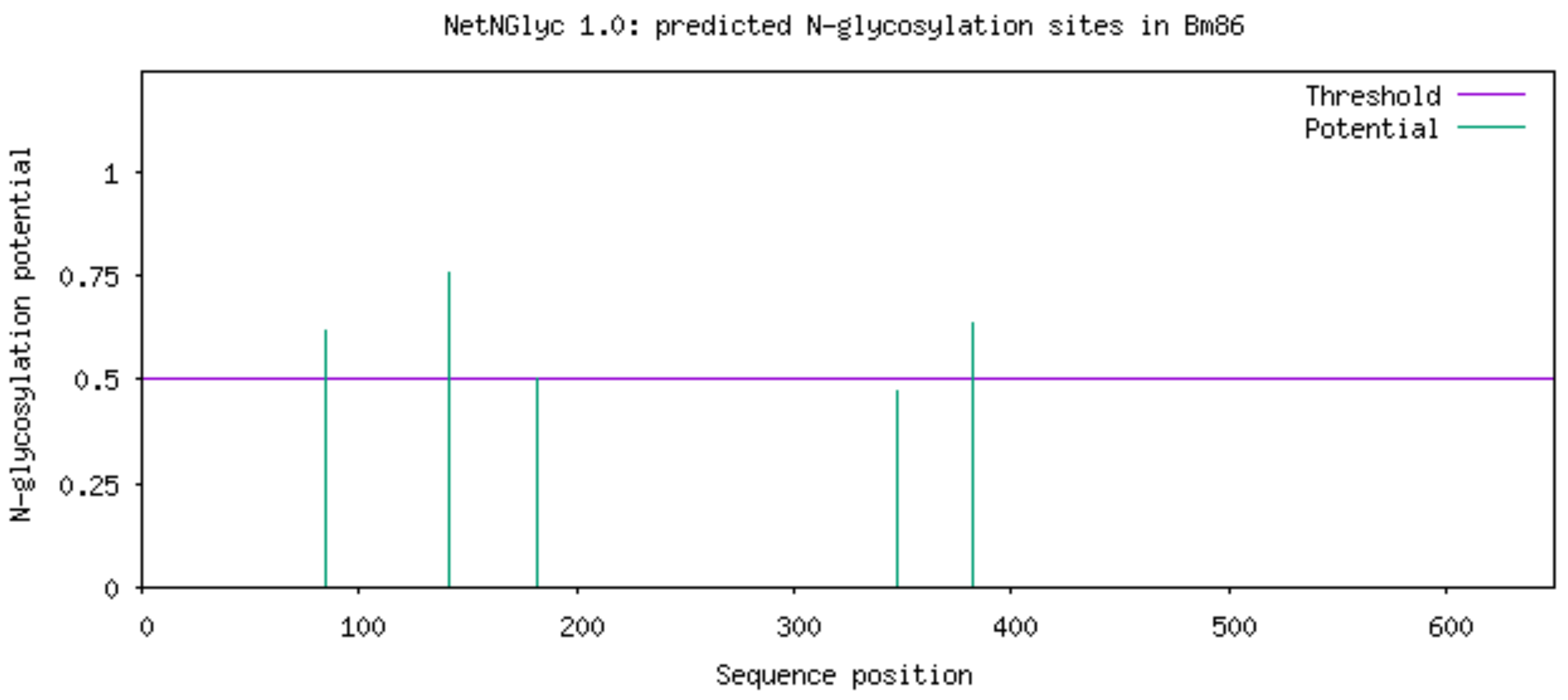
NetNGlyc 1.0: predicted N-glycosylation sites in Bm86
Threshold
Potential
N-glycosylation potential
1
0.75
0.5
0.25
0
0
100
200
300
400
500
600
Sequence position

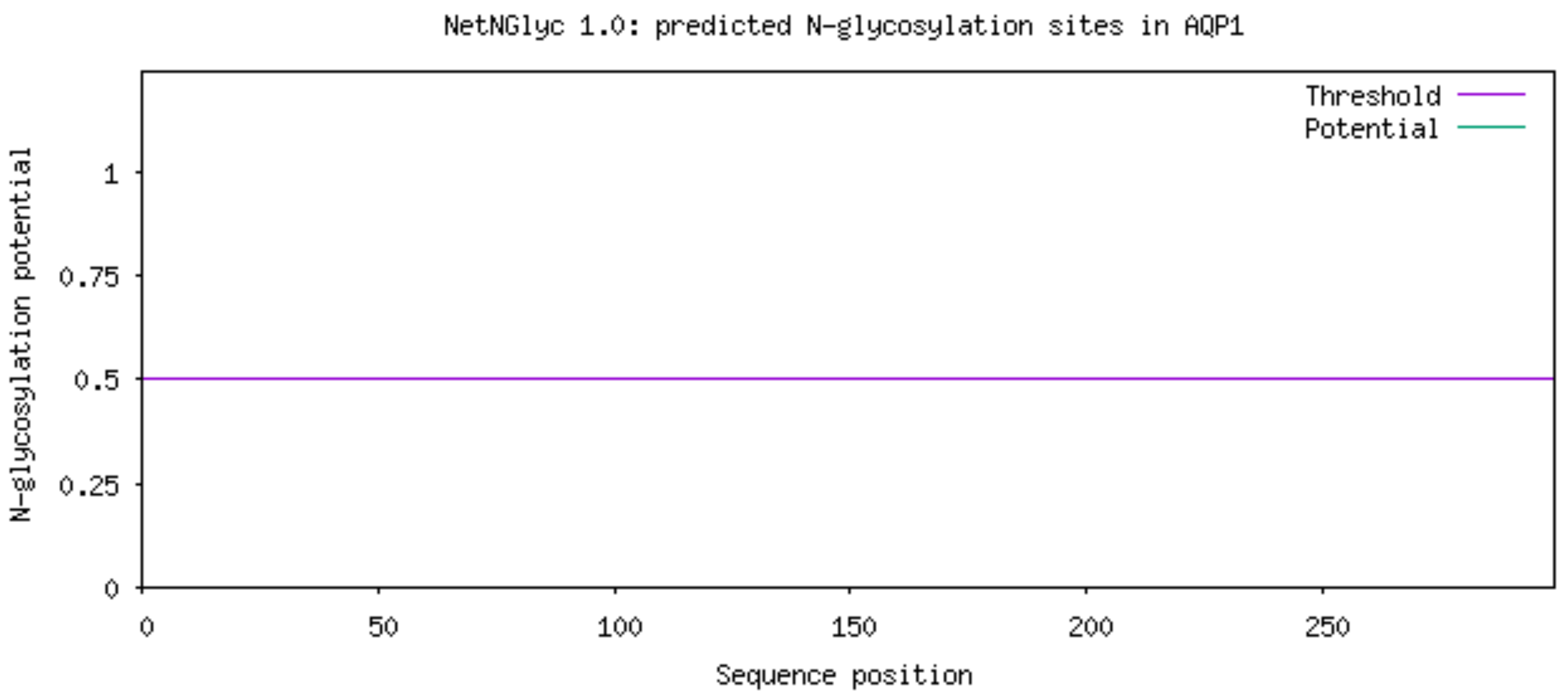
NetNGlyc 1.0: predicted N-glycosylation sites in AQP1
Threshold
Potential
N-glycosylation potential
1
0.75
0.5
0.25
0
0
50
100
150
200
250
Sequence position

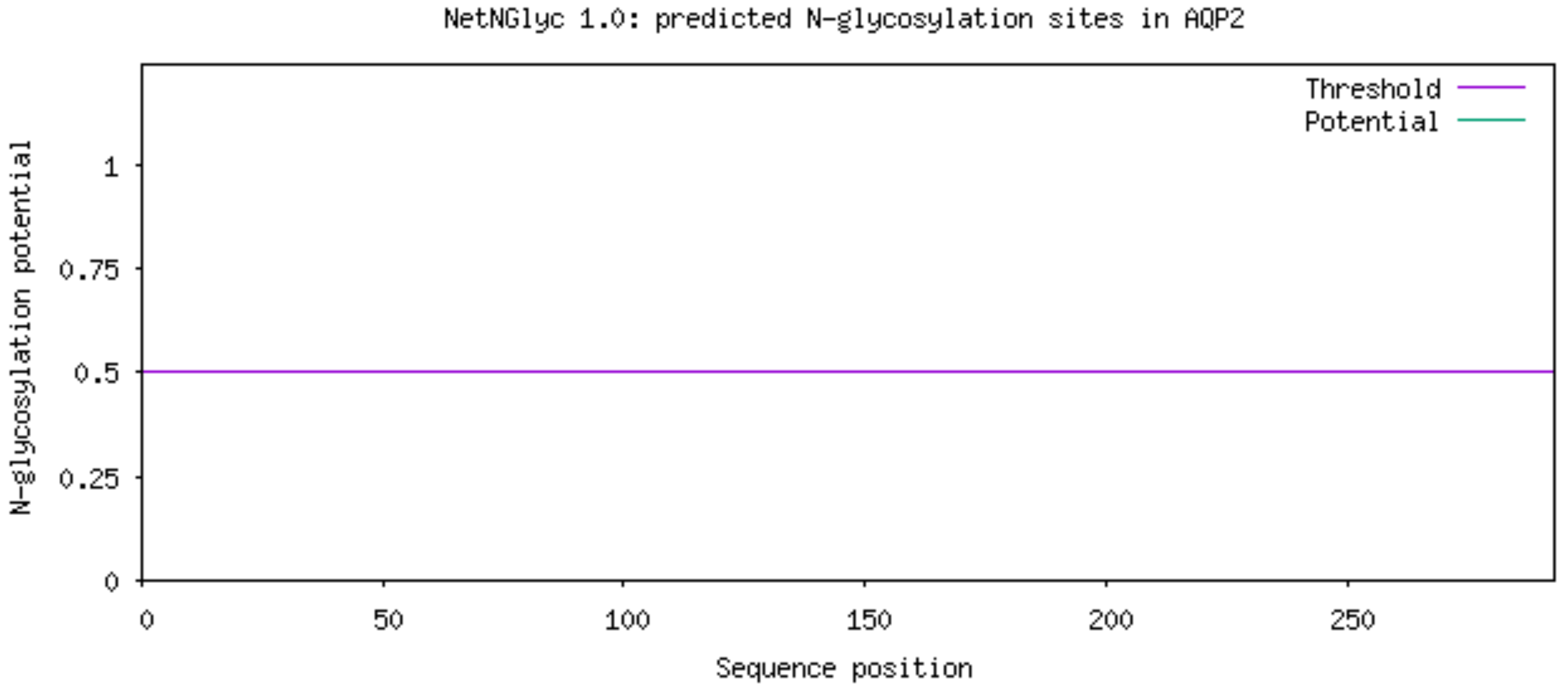
NetNGlyc 1.0: predicted N-glycosylation sites in AQP2
Threshold
Potential
N-glycosylation potential
1
0.75
0.5
0.25
0
0
50
100
150
200
250
Sequence position

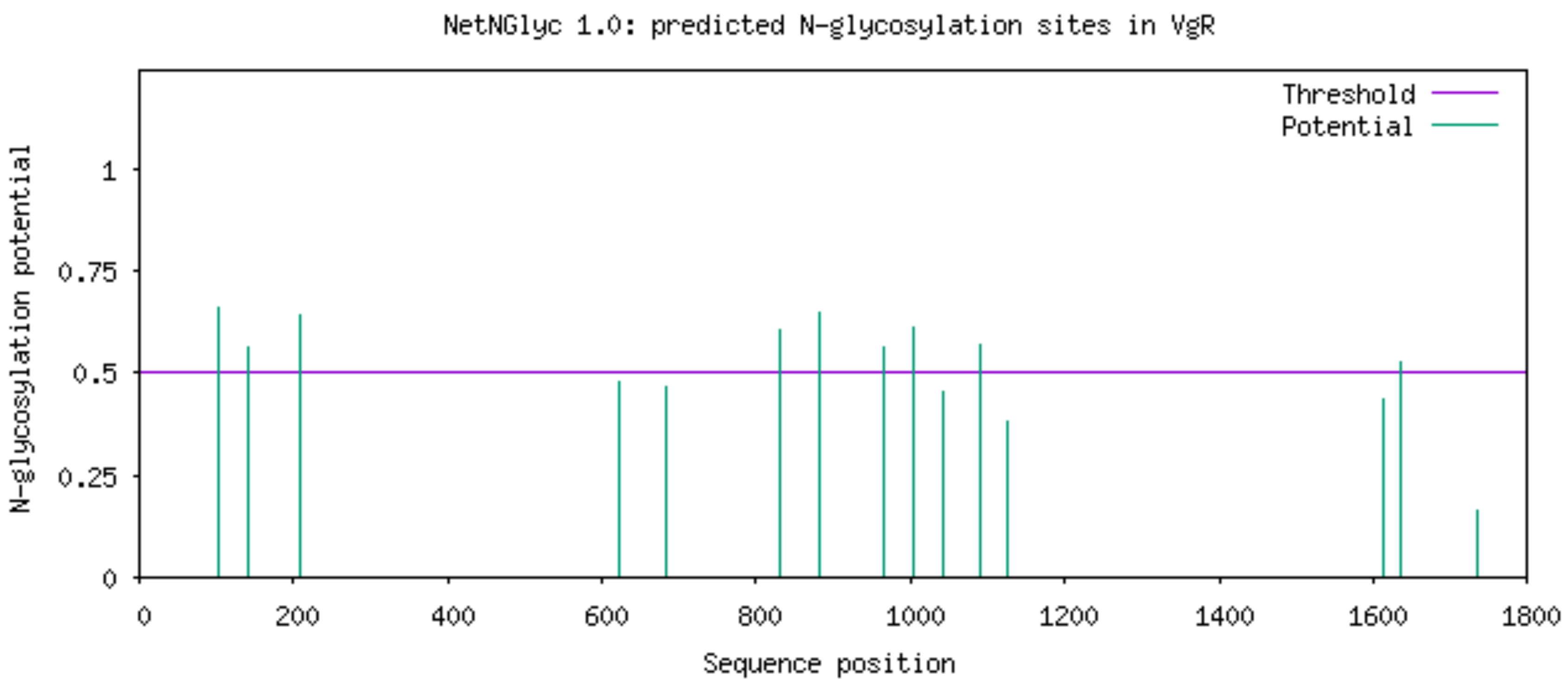
NetNGlyc 1.0: predicted N-glycosylation sites in VgR
Threshold
Potential
N-glycosylation potential
1
0.75
0.5
0.25
0
0
200
400
600
800
1000
1200
1400
1600
1800
Sequence position
