# Supplementary material for: Comparative immunoinformatic analysis of Rhipicephalus microplus cocktail vaccine targets
Source: Parasit Vectors. 2025 Dec 9;18:502. doi: 10.1186/s13071-025-07109-y (PMC12690872; doi:10.1186/s13071-025-07109-y)
Supplement: Supplementary file 6 — Additional file 6: Figure S6. Phosphorylation sites prediction of vaccine target R. microplus proteins (Bm86, AQP1, AQP2, and VgR). [file 13071_2025_7109_MOESM6_ESM.pdf]

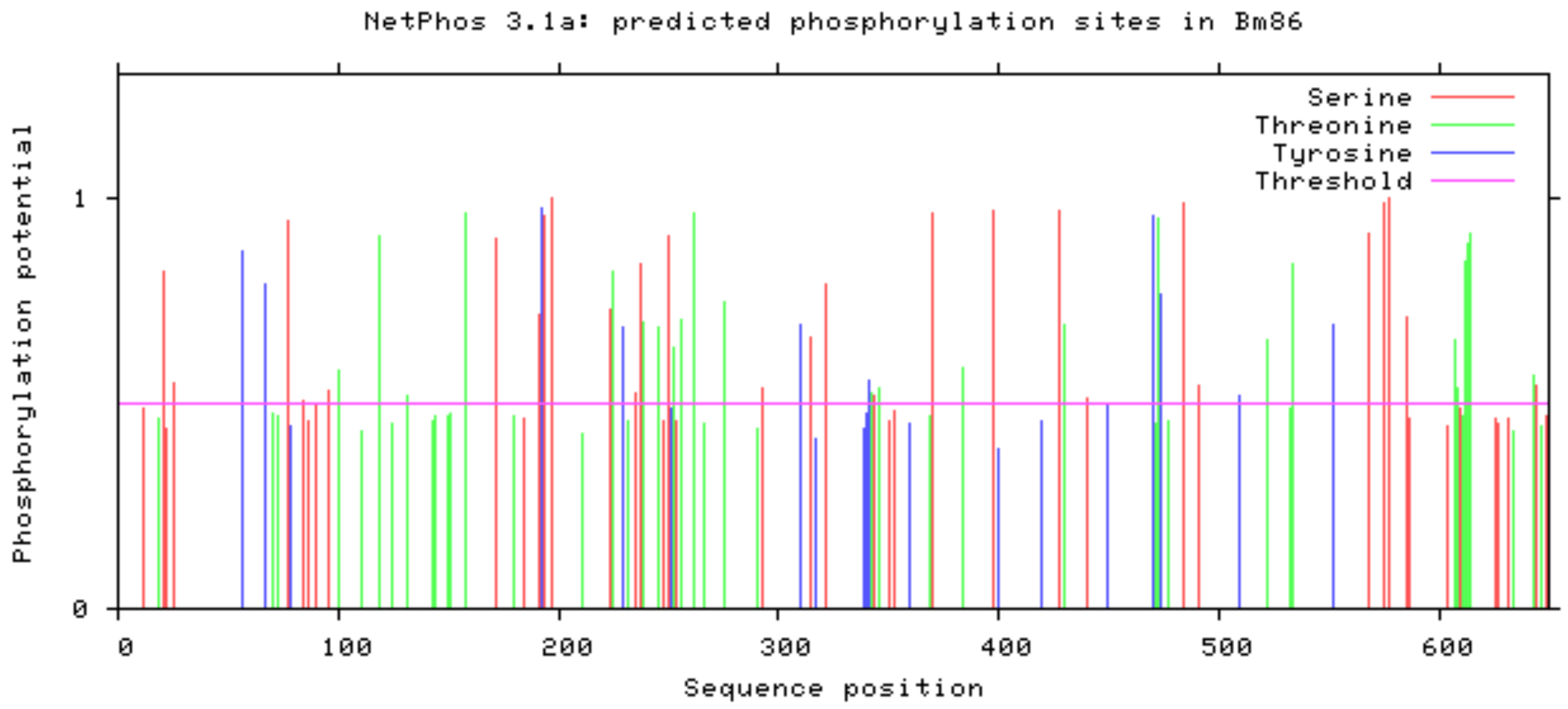
NetPhos 3.1a: predicted phosphorylation sites in Bm86
Serine
Threonine
Tyrosine
Threshold
Phosphorylation potential
1
0
0
100
200
300
400
500
600
Sequence position

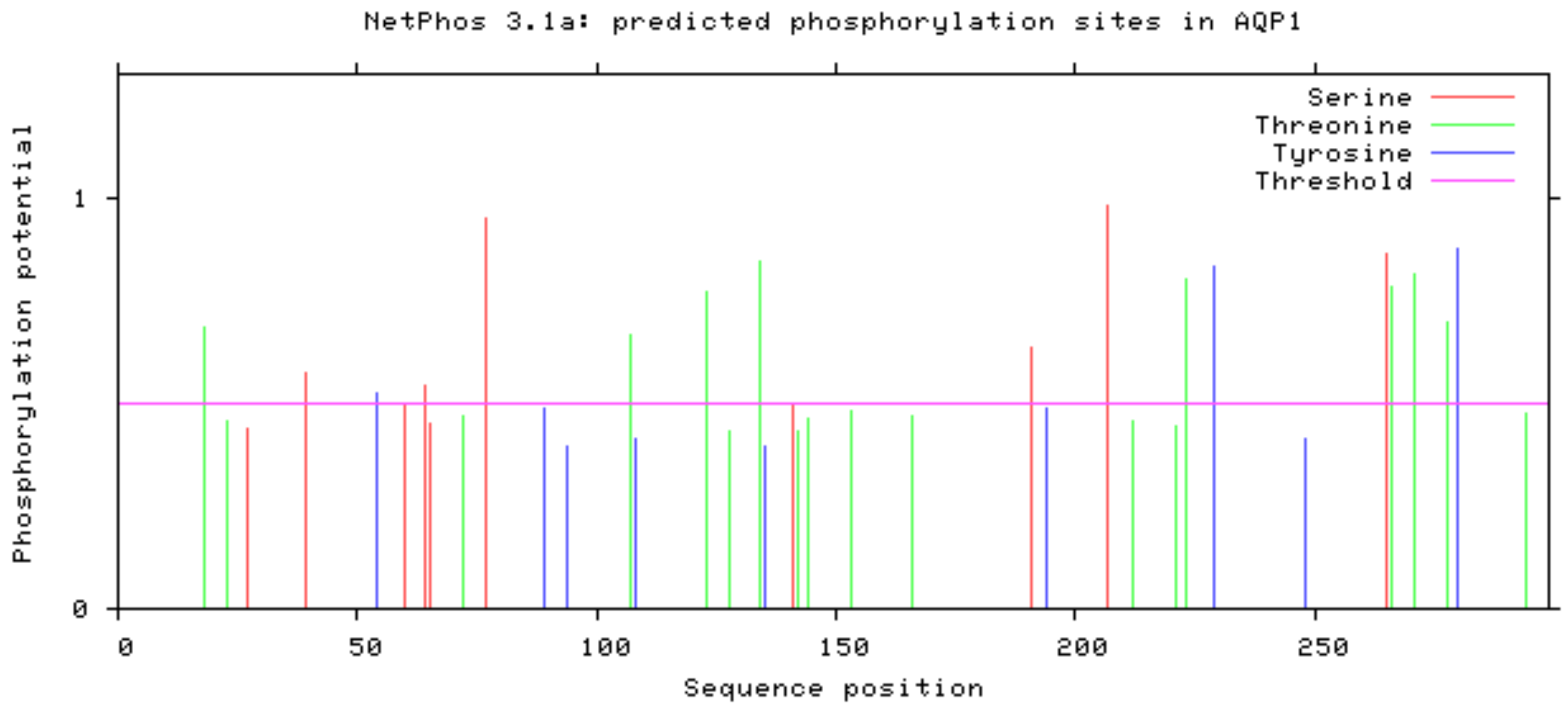
NetPhos 3.1a: predicted phosphorylation sites in AQP1
Serine
Threonine
Tyrosine
Threshold
Phosphorylation potential
1
0
0
50
100
150
200
250
Sequence position

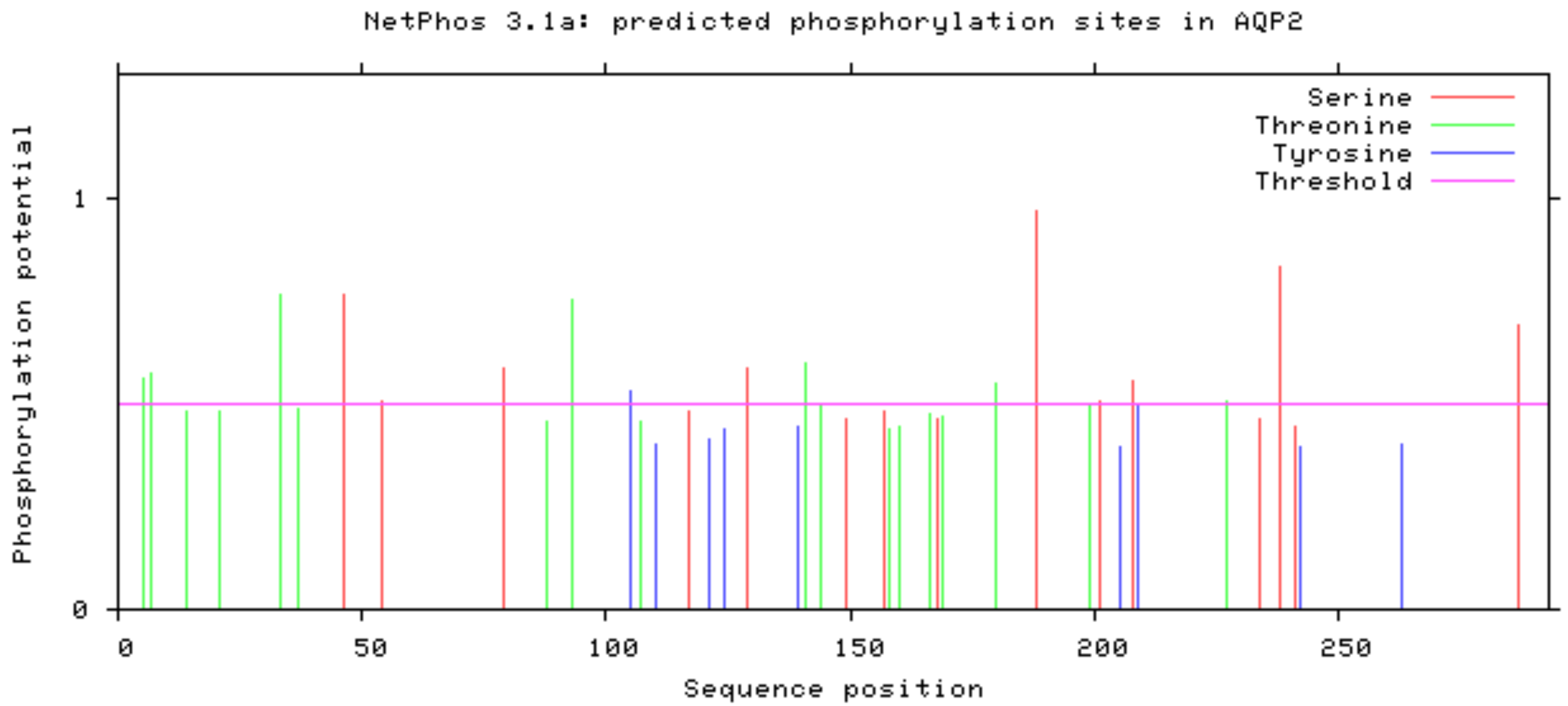

NetPhos 3.1a: predicted phosphorylation sites in AQP2
Serine
Threonine
Tyrosine
Threshold
Phosphorylation potential
1
0
0
50
100
150
200
250
Sequence position

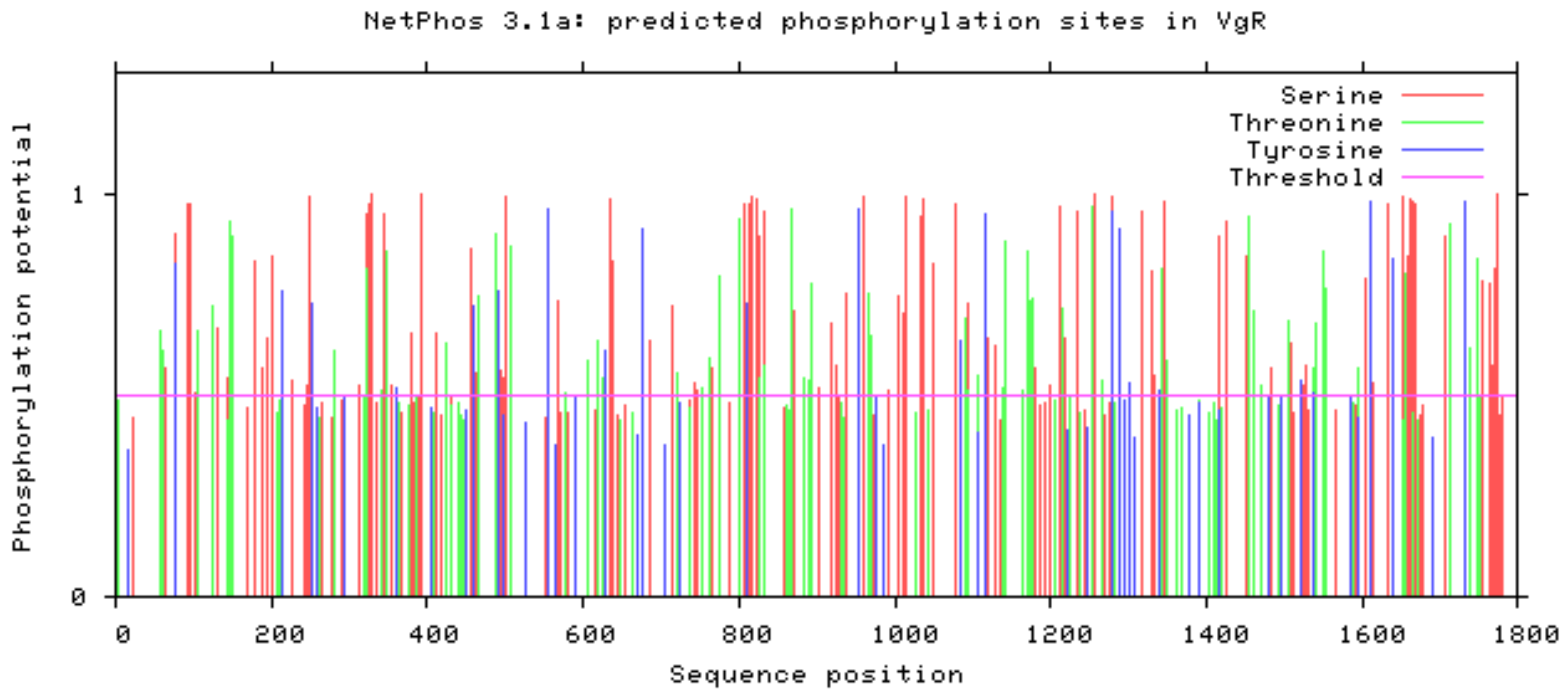
NetPhos 3.1a: predicted phosphorylation sites in VgR
Serine
Threonine
Tyrosine
Threshold
Phosphorylation potential
1
0
0
200
400
600
800
1000
1200
1400
1600
1800
Sequence position
