# Supplementary material for: Comparative immunoinformatic analysis of Rhipicephalus microplus cocktail vaccine targets
Source: Parasit Vectors. 2025 Dec 9;18:502. doi: 10.1186/s13071-025-07109-y (PMC12690872; doi:10.1186/s13071-025-07109-y)
Supplement: Supplementary file 7 — Additional file 7: Figure S7. Sequence identity matrix heatmaps for tick species and Bos taurus of vaccine target R. microplus proteins (Bm86, AQP1, AQP2, and VgR). [file 13071_2025_7109_MOESM7_ESM.pdf]

Bm86

	1	2	3	4	5	6	7	8	9
1	100	21.05	19.47	19.03	22.16	22.57	22.16	21.08	21.74
2	21.05	100	84.7	32.35	34.12	34.49	35.12	33.27	33.4
3	19.47	84.7	100	31.59	32.43	32.43	32.53	32.11	32.05
4	19.03	32.35	31.59	100	45.22	44.4	44.6	42.06	43.43
5	22.16	34.12	32.43	45.22	100	57.65	60.12	56.32	56.34
6	22.57	34.49	33.03	44.4	57.65	100	68.97	66.94	67.15
7	22.16	35.12	32.53	44.6	60.12	68.97	100	72.07	71.36
8	21.08	33.27	32.11	42.06	56.32	66.94	72.07	100	93.52
9	21.74	33.4	32.05	43.43	56.34	67.15	71.36	93.52	100

10								100

AQP1

	1	2	3	4	5	6	7
1	100	38.89	36.96	36.46	37.55	38.04	37.45
2	38.89	100	45.7	45.38	43.97	43.75	44.71
3	36.96	45.7	100	64.56	67.22	64.77	64.65
4	36.46	45.38	64.56	100	71.93	68.31	67.96
5	37.55	43.97	67.22	71.93	100	84.62	82.55
6	38.04	43.75	64.77	68.31	84.62	100	92.28
7	37.45	44.71	64.65	67.96	82.55	92.28	100

10						100

AQP2

	1	2	3	4	5	6	7
1	100	38.22	39.46	34.63	33.92	35.34	34.75
2	38.22	100	52.12	52.02	54.03	54.03	54.66
3	39.46	52.12	100	63.18	64.16	64.16	65.29
4	34.63	52.02	63.18	100	78.5	75.77	78.35
5	33.92	54.03	64.16	78.5	100	85.32	90.03
6	35.34	54.03	64.16	75.77	85.32	100	92.44
7	34.75	54.66	65.29	78.35	90.03	92.44	100

10						100

VgR

	1	2	3	4	5	6	7	8
1	100	67.27	17.6	19.22	19.14	17.7	19.53	19.92
2	67.27	100	18.23	19.13	19.35	20.95	21.52	21.9
3	17.6	18.23	100	32.67	32.33	33.31	33.35	33.18
4	19.22	19.13	32.67	100	58.61	61.8	60.76	61.37
5	17.7	19.35	32.33	58.61	100	81.61	74.03	75.1
6	19.14	20.95	33.31	61.8	81.61	100	84.54	85.82
7	19.53	21.52	33.35	60.76	74.03	84.54	100	92.38
8	19.92	21.9	33.18	61.37	75.1	85.82	92.38	100

10							100
