# Supplementary material for: Comparative immunoinformatic analysis of Rhipicephalus microplus cocktail vaccine targets
Source: Parasit Vectors. 2025 Dec 9;18:502. doi: 10.1186/s13071-025-07109-y (PMC12690872; doi:10.1186/s13071-025-07109-y)
Supplement: Supplementary file 8 — Additional file 8: Dataset S1. Protein sequence alignment of R. microplus proteins (Bm86, AQP1, AQP2, and VgR) with Bos taurus and tick species. [file 13071_2025_7109_MOESM8_ESM.pdf]

The similarity of protein sequence regions that might raise autoimmune concerns with the Bos taurus protein sequence or offer cross-protective potential with multiple tick species:

AQP1 against Bos taurus

```
CLUSTAL O(1.2.4) multiple sequence alignment


aquaporin-9,[Bostaurus],NP_001192762.2      MQPEMEQKKKSLKQRLVLKNTLAKETLSEFLGTFIMIVLGCGSVAQAVLSRGHFGGIITI        60
AQP1Rm*                                     ---------------MKIENLLIRQLINEFLGTMILITIGDSIMAIIIAGDNESLAACVG        45
                                                           : ::* * :: :.*****:*:*.:* . :*  : . ..  .  .

aquaporin-9,[Bostaurus],NP_001192762.2      NTGFSMAVAMAIYVSAGVSGGHINPAVSFALCLFGRMKWFKFPFYVGAQFLGAFAGAATL        120
AQP1Rm*                                     PLGWGVAIYVAVQISGG-VSSHLNPAVTLAQASVRKFPIAKVPLYFAAQYLGGFVGAALV        104
                                              *:.:*: :*: :*.*  ..*:****::* . . ::   *.*:*..**:**.*.*** :

aquaporin-9,[Bostaurus],NP_001192762.2      FGIYYDAFMSFAGGKLLIVGENATAHIFATYPAPYLSLVNAFAEQVVATMFLLIVIFAIF        180
AQP1Rm*                                     FATYKDAIEHFDQGIRQVTGEKATAGIFATYPRPHVSTLTCFIDQVIATGIMMVCVEAIG        164
                                            *. * **:  *  *   :.**:*** ****** *::* :..* :**:** :::: : **

aquaporin-9,[Bostaurus],NP_001192762.2      DSRNL-GVPRGLEPVVIGFLIVTIASSLGMNSGCAMNPARDLSPRLFTALAGWGFEVFTA        239
AQP1Rm*                                     DTRNFGGIPPHIHPICLGLMIMAIIFSFAYNCMCPLNPARDISPRLFTLMAGWGPETFTL        224
                                            *:**: *:*  :.*: :*::*::*  *:. *. * :*****:****** :**** *.**

aquaporin-9,[Bostaurus],NP_001192762.2      -GNNFWWIPVVGPLVGAAAGGFVYFLLIEIHHPDLNPD-LEAEQPEDKPEK----YELNA        293
AQP1Rm*                                     RGWNYVWVPLLGPHIGAILGVWLYKVAIGDHWPEKPKPAISTDGKETKEDLVETLYKVDG        284
                                             * *: *:*::** :**  * ::* : *  * *:     :.::  * * :     *:::.

aquaporin-9,[Bostaurus],NP_001192762.2      IM-------------     295
AQP1Rm*                                     DKMVLELEPTQHQRL     299
```

AQP1 against other tick species

```
CLUSTAL O(1.2.4) multiple sequence alignment


AQP1Ir,CAX48964.1                ------------------------------------------------------------   0
AQP1Is,XP_029845132.1            ------------------------------------------------------------   0
HypotheticalHl,KAH9370733.1      MLRQIALTSKVAPAVTCVVGAHFSAPQFVAPSVATPSNLGNQKRKRLQHVFKRHLRGAIC   60
AQP1Da,XP_050044617.1            ------------------------------------------------------------   0
AQP1Rm*                          ------------------------------------------------------------   0
AQP1Rs,XP_037510823.1            ------------------------------------------------------------   0


AQP1Ir,CAX48964.1                ------------------------------------------------------------   0
AQP1Is,XP_029845132.1            ------------------------------------------------------------   0
HypotheticalHl,KAH9370733.1      CDAFKSRKPKAKAPPTRVQAAPSALKSSCQMRALGNGPSAVEGARDRKPAAGEVYLPRNS   120
AQP1Da,XP_050044617.1            ------------------------------------------------------------   0
AQP1Rm*                          ------------------------------------------------------------   0
AQP1Rs,XP_037510823.1            ------------------------------------------------------------   0


AQP1Ir,CAX48964.1                --------------------------------------MQLFGNTVLAILNHVCMTFEKA   22
AQP1Is,XP_029845132.1            ------------------MKIESRLIRELINEFIGTMILVLIGDSILAIII-------AG   35
HypotheticalHl,KAH9370733.1      ERLESVQICGATATKSFNMKIQNPLIREFISEFIGTMVLILIGDSVLAVII-------AG   173
AQP1Da,XP_050044617.1            ------------------MKIENLLVRQLINEFLGTLVLILIGDSVLAIII-------AG   35
AQP1Rm*                          ------------------MKIENLLIRQLINEFLGTMILITIGDSIMAIII-------AG   35
AQP1Rs,XP_037510823.1            ------------------MKIESLLVRQLISEFLGTMILITIGDAIMAIII-------AG   35
                                                                       :  :*::::*::         .

AQP1Ir,CAX48964.1                STDGLAAC--FWGWGLALTLGVLVAGGASGAHLNPAIAVAVTTIGKFPWRKIVPYVIAQY   80
AQP1Is,XP_029845132.1            DNEPLAPIVGPVGWGTAIFVAVTIAGGV-SSHLNPAVTLALASTKKFPINKVPLYFAVQY   94
HypotheticalHl,KAH9370733.1      DNESIAAVVGPLGWGVAIFVAVSVAGGV-SAHLNPAVTLSLASARKFPLAKVPLYFAAQY   232
AQP1Da,XP_050044617.1            ENEPIAAVVGPLGWGVAIYVAVQIAGGV-TAHLNPAVTLALASVRKFPIAKVPLYFAAQY   94
AQP1Rm*                          DNESLAACVGPLGWGVAIYVAVQISGGV-SSHLNPAVTLAQASVRKFPIAKVPLYFAAQY   94
AQP1Rs,XP_037510823.1            DNESLAACVGPLGWGVAIYVAVQIAGGV-SSHLNPAVTLAQASVRKFPIAKVPLYFAAQY   94
                                 ..: :*      *** *: :.* ::**.  :*****:::: ::  ***  *:  *. .**

AQP1Ir,CAX48964.1                IGAFIASVVLFITYRGALDNFDGGNRIVTGVNGTAGIFASYPKEFLSTGNGLVDQIVGTA   140
AQP1Is,XP_029845132.1            LGAFVGAALVYLLYHDQIAHFDGGVRQITGKHGTAPIFATYPREQISTLTCFFDQLVGTG   154
HypotheticalHl,KAH9370733.1      LGAFVGAALVFITYRDQIAHFDKGQRMVTGKTATAGIFATYPKEHVSTLTCFIDQVIATG   292
AQP1Da,XP_050044617.1            LGAFVGAALVFVTYKDCIEHYDQGVRQVVGKKATAAIFATYPREHVSTLTCFIDQVISTG   154
AQP1Rm*                          LGGFVGAALVFATYKDAIEHFDQGIRQVTGEKATAGIFATYPRPHVSTLTCFIDQVIATG   154
AQP1Rs,XP_037510823.1            LGGFVGAALVFVTYKDAIEHFDQGVRQVTGEKSTAGIFATYPKPHVSTLTCFIDQVIATG   154
                                 :*.*:.:.:::  *:. : ::* * * :.*  .** ***:**:  :** . :.**::.*.

AQP1Ir,CAX48964.1                LLMLCILAITDARNMA-VPQGVQPLFIGFALAAIILSFGYNCGAPLNPARDLAPRVFTAM   199
AQP1Is,XP_029845132.1            ILMLTAEAITDPRNFGGMPKHLHPLALGFMIMALIFGFSYNCMAPLNPARDIGPRIFTAI   214
HypotheticalHl,KAH9370733.1      LLSLTAEAITDERNFGGIPKQMHPICLGLMIMALIFGFANNCMCPLNPARDLSPRIFTLL   352
AQP1Da,XP_050044617.1            ILMLTAEAITDPRNFGGIPVSIHPICLALMIMALIFSFAYNCMCPLNPARDISPRLFTLM   214
AQP1Rm*                          IMMVCVEAIGDTRNFGGIPPHIHPICLGLMIMAIIFSFAYNCMCPLNPARDISPRLFTLM   214
AQP1Rs,XP_037510823.1            IMMVCCEAIGDPRNFGGIPPHIHPICLGLLIMALIFSFAYNCMCPLNPARDIAPRLFTLM   214
                                 :: :   ** * **:. :*  ::*: :.: : *:*:.*. ** .*******:.**:** :

AQP1Ir,CAX48964.1                AGWGGEVFSYRDYNWFWVPILGPHIGAILGAWIYTLAVELHWPGATYEMDGG----NPVS   255
AQP1Is,XP_029845132.1            AGWGTEVFTYRNWNYIWVPIFGPHIGAIIGAWIYKVGIGDNFPDDEPKLNGDLERGKVV-   273
HypotheticalHl,KAH9370733.1      AGWSTETFTLRNWNYVWVPIVGPHIGAILGCVAVQGG--HRRPLAQPAATRR--------   402
AQP1Da,XP_050044617.1            AGWGPETFTLRGWNYVWVPVVGPHIGAILGAWLYKVAIADHWPDIKPKPTTSIDGKEAKE   274
AQP1Rm*                          AGWGPETFTLRGWNYVWVPLLGPHIGAILGVWLYKVAIGDHWPE-KPKPAISTDGKETKE   273
AQP1Rs,XP_037510823.1            AGWGPETFTLRGGNYVWVPLLGPHIGAILGVWLYKVAIGDHWPD-RPKPA-STNGKETKE   272
                                 ***. *.*: *. *:.***:.*******:*      .   . *

AQP1Ir,CAX48964.1                TKD-------GGYTGAIP---------------      266
AQP1Is,XP_029845132.1            ELQEAAYRPIDDKLN-LEAHTNEALKL------      299
HypotheticalHl,KAH9370733.1      -----LQYVSGRALPAVFFFPSHPHPRIRSVAL      430
AQP1Da,XP_050044617.1            ELVETLYKVDGDKLV-LELEPTQHQKL------      300
AQP1Rm*                          DLVETLYKVDGDKMV-LELEPTQHQRL------      299
AQP1Rs,XP_037510823.1            DLVETLYKVDGDKMV-LELEPAQQHQRL-----      299
                                           .     :
```

AQP2 against Bos taurus

```
CLUSTAL O(1.2.4) multiple sequence alignment


Bostaurus,aquaporin-9      MQPEMEQKKKSLKQRLVLKNTLAKETLSEFLGTFIMIVLGCGSVAQAVLSRGHFGGIITI 60
AQP2Rm*                    MKPNTVTRAWRQVTGCCIENTLARQALAEMVGTLVLTLVGDCVLASLAVFQLGSVGLAAA 60
                           *:*:   :         ::****:::*:*::**::: ::*   :*. .: :    *: :

Bostaurus,aquaporin-9      NTGFSMAVAMAIYVSAGVSGGHINPAVSFALCLFGRMKWFKFPFYVGAQFLGAFAGAATL 120
AQP2Rm*                    PLGWGLAVFLGVLVAGGVSGAHLNPAVTVALATIGKLGWCNVLAYVTAQYLGAFLASGLV 120
                             *:.:** :.: *:.****.*:****:.**. :*:: * :.  ** **:**** .:. :

Bostaurus,aquaporin-9      FGIYYDAFMSFAGGKLLIVGENATAHIFATYPAPYLSLVNAFAEQVVATMFLLIVIFAIF 180
AQP2Rm*                    YLVYADALSQVDVNLAIVYGTNATAPVFSCFPAPGVSTLTCLLDQTVSTAVLLLGICAIT 180
                           : :* **: ..  .  :: * **** :*: :*** :* :..: :*.*:* .**: * **

Bostaurus,aquaporin-9      DSRNLGVPRGLEPVVIGFLIVTIASSLGMNSGCAMNPARDLSPRLFTALAGWGFEVFTAG 240
AQP2Rm*                    DGRNMAVSRGQQPLLVGLTVSACMYAFSYNCGNPLNPARDLAPRIFTAMCGWGSAVFSFR 240
                           *.**:.* ** :*:::*: : :   ::. *.*  :******:**:***:.***  **:

Bostaurus,aquaporin-9      -NNFWWIPVVGPLVGAAAGGFVYFLLIEIHHPDLNPDLEAEQPEDKPEKYELNAIM--   295
AQP2Rm*                    SYNWFWVPVVGPHLGAVIGVWIYKLAVDNHWKDEDEVDED---EKRPL--LSNAKICA   293
                             *::*:***** :**. * ::* * :: *  * :   *    *.:*     ** :
```

AQP2 against other tick species

```
CLUSTAL O(1.2.4) multiple sequence alignment


AQP2Ir,CAX48964.1                   -----------------------------------MQLFGNTVLAILNHVCMTFEKASTD   25
AQP2Is,XP_029851087.2               MKPNSVARVWSRICNCRLESRSCRDVLAEFLGTLILTLIGDSVLASL-----TASRLGYF   55
hypotheticalHl,KAH9380169.1         MKPNAFIRAWQRATGCSIGKPLVRQSLAELVGTLVLTLVGDCVLASL-----TVSRLGAV   55
AQP2Da,XP_050050937.1               MKPNAVTRAWRRVCGCCIEKPLARQALAEMVGTLVLTLVGDCVLASL-----TVFQLGST   55
AQP2Rm*                             MKPNTVTRAWRQVTGCCIENTLARQALAEMVGTLVLTLVGDCVLASL-----AVFQLGSV   55
AQP2Rs,XP_037518224.1               MKPNAVTRAWRRVSGCCIEKPLARQALAEMVGTLVLTLVGDCVLASL-----AVFQLGSV   55
                                                                       : *.*: *** *     :  : .

AQP2Ir,CAX48964.1                   GLAACFWGWGLALTLGVLVAGGASGAHLNPAIAVAVTTIGKFPWRKIVPYVIAQYIGAFI   85
AQP2Is,XP_029851087.2               GIAAGPLGWGLAVYLGIIVAGGVSGGHLNPAVTLGLASAGKFKWAKVLPYIAAQYLGAFA   115
hypotheticalHl,KAH9380169.1         GLAAGPLGWGLAVFLGVLVAGGVSGAHLNPAVTVAMATVGKISWRKVLPYVASQYAGAFL   115
AQP2Da,XP_050050937.1               GLAAGPLGWGLAVFLGVLVAGGVSGAHLNPAVTVAMATVGKLAWCKVLAYVAAQYVGAFL   115
AQP2Rm*                             GLAAAPLGWGLAVFLGVLVAGGVSGAHLNPAVTVALATIGKLGWCNVLAYVTAQYLGAFL   115
AQP2Rs,XP_037518224.1               GLAAGPLGWGLAVFLGVLVAGGVSGAHLNPAVTVALATIGKLGWCNVLAYVAAQYLGAFL   115
                                    *:**   *****: **::****.**.*****:::.::: **: * ::: *: :** ***

AQP2Ir,CAX48964.1                   ASVVLFITYRGALDNF--DGGNRIVTGVNGTAGIFASYPKEFLSTGNGLVDQIVGTALLM   143
AQP2Is,XP_029851087.2               AAGLIFLLYQDALYKV--DGGMRVVYGPNATASVFSCFPAPEVRTLTCLLDQVVSTAVLL   173
hypotheticalHl,KAH9380169.1         ASCLVYMVYADALRLVRWTALLTAVYGENATAPVFSCFPAPGVSTFTCFLDQVVSTAVLL   175
AQP2Da,XP_050050937.1               ASCLVYVVYTDALSQI--DANLTTVYGINATAPVFSCFPAPGVSTFTCLVDQIVSTAVLL   173
AQP2Rm*                             ASGLVYLVYADALSQV--DVNLAIVYGTNATAPVFSCFPAPGVSTLTCLLDQTVSTAVLL   173
AQP2Rs,XP_037518224.1               ASGLVYLVYADALAQV--DANLDTVYGINATAPVFSCFPAPGVSTLTCLLDQTVSTAVLL   173
                                    *: :::: * .**  .        * * *.** :*:.:*   : * . ::** *.**:*:

AQP2Ir,CAX48964.1                   LCILAITDARNMAVPQGVQPLFIGFALAAIILSFGYNCGAPLNPARDLAPRVFTAMAGWG   203
AQP2Is,XP_029851087.2               VSICAIVDPKNMAVLKGHQPLLIGFAVAACMYAFSYNCGNPLNPARDLAPRIFTAMAGWG   233
hypotheticalHl,KAH9380169.1         LGICAVTDNRNMSVPRGQQPLLIGLTVSACMYAFSSNCGNPLNPARDLAPRIFTAISSWG   235
AQP2Da,XP_050050937.1               VGICAITDTRNMDVSRGQQPLLVGLTVSACMYAFSYNCGNPLNPARDLAPRIFTAMGGWG   233
AQP2Rm*                             LGICAITDGRNMAVSRGQQPLLVGLTVSACMYAFSYNCGNPLNPARDLAPRIFTAMCGWG   233
AQP2Rs,XP_037518224.1               LGICAITDTRNMAVPRGQQPLLVGLTVSACMYAFSYNCGNPLNPARDLAPRIFTAMCGWG   233
                                    : * *:.* :** * :* ***::*::::* : :*. *** ***********:***: .**

AQP2Ir,CAX48964.1                   GEVFSYRDYNWFWVPILGPHIGAILGAWIYTLAVELHWPGATYEMDGGNPVSTKDGGYTG   263
AQP2Is,XP_029851087.2               CDVFSIRGYNWFWVPVVGPHVGGVVGVWVYKFAVENHWATDAAAVVGKTPEEEHD--YEG   291
hypotheticalHl,KAH9380169.1         SAVFSFRSYNWFWVPVVGPHVGGIVGVWTYKLAVDNHWESEDDTDSC------------E   283
AQP2Da,XP_050050937.1               SAVFSFRAYNWFWVPVVGPHLGAVIGVWIYKLAVDNHWRADDEIDAA------------E   281
AQP2Rm*                             SAVFSFRSYNWFWVPVVGPHLGAVIGVWIYKLAVDNHWKDEDEVDED------------E   281
AQP2Rs,XP_037518224.1               SAVFSFRAYNWFWVPVVGPHLGAVIGVWIYKLAVDNHWRDEDEIDD-------------E   280
                                      *** * *******::***:*.::*.* *.:**: **

AQP2Ir,CAX48964.1                   AIP---------------     266
AQP2Is,XP_029851087.2               KRRLLRQDTHYIEADIAS     309
hypotheticalHl,KAH9380169.1         -RKLLRSSRGSNGPAA--     298
AQP2Da,XP_050050937.1               -RKPLLGSPKLVA-----     293
AQP2Rm*                             -KRPLLSNAKICA-----     293
AQP2Rs,XP_037518224.1               -KKPLLGNGKIA------     291
```

Bm86 against Bos Taurus

```
CLUSTAL O(1.2.4) multiple sequence alignment


Bm86Rm*                     ------------------------------------------------------------	0
Bostaurus,hemicentin-2      MRPRAPLLRLLLAVSAAAAAVVGEPGTALPPTTGGATLAIVFDVTGSMWDDLMQVMDGAS	60


Bm86Rm*                     ------------------------------------------------------------	0
Bostaurus,hemicentin-2      RILEHSLSRGSRVIANYALVPFHDPDIGPVTLTSDPAVFQRELRELYVQGGGDCPEMSLG	120


Bm86Rm*                     ------------------------------------------------------------	0
Bostaurus,hemicentin-2      AIKAAVEVANPGSFIYVFSDARAKDYHKKDEVLRLLQLKQPQVVFVLTGDCGDRTHPGYL	180


Bm86Rm*                     ------------------------------------------------------------	0
Bostaurus,hemicentin-2      AYEEIAATSSGQVFHLDKQQVTEVLKWVESALQASKVHLLSTDHEEGGEHTWKIPFDPSL	240


Bm86Rm*                     ------------------------------------------------------------	0
Bostaurus,hemicentin-2      KEVTISLSGPGPEIEVRDPLGRILQRDDGLNVLLSIPDSAKVVAFKPEHSGLWSIKIYSS	300


Bm86Rm*                     ------------------------------------------------------------	0
Bostaurus,hemicentin-2      GRHSVRITGISNIDFRAGFSTQPSLDLNHTIEWPLQGVPISLVINSTGLRVPGLLDSVEL	360


Bm86Rm*                     ------------------------------------------------------------	0
Bostaurus,hemicentin-2      SHSSGRSLLTLPTQPLSNGSTHQLWGGPPFHSPQERFYLKVKGKDHEGNPLLRVSGVSYR	420


Bm86Rm*                     ------------------------------------------------------------	0
Bostaurus,hemicentin-2      GVIPGAPLVSMPPRIHGYLHQPLQVSCSVHSALPFRLQLWRDGARLGEERRFRESGNSSW	480


Bm86Rm*                     ------------------------------------------------------------	0
Bostaurus,hemicentin-2      EIPRASKAEEGTYECTAVSRAGTGRAKTQIVVTGPPPQLVPVPNVTVSPGETAILSCLVL	540


Bm86Rm*                     ------------------------------------------------------------	0
Bostaurus,hemicentin-2      SEAPYNLTWVRDWRVLSASTGRVTQLADLSLEVRNTTPSDGGRYQCMASNSNGVTRASVW	600


Bm86Rm*                     ------------------------------------------------------------	0
Bostaurus,hemicentin-2      LLVREAPQVSIHTRSQRFSQGMEVRVRCSASGYPAPHISWSREGRALQEDSRVRVDAQGT	660


Bm86Rm*                     ------------------------------------------------------------	0
Bostaurus,hemicentin-2      LIIQGVAPEDAGSYSCQAANEIGRDEETVTLYYTDPPSVSAVNGVVLAAKGEEAVLECEA	720


Bm86Rm*                     ------------------------------------------------------------	0
Bostaurus,hemicentin-2      TGVPPPRVIWYRGDLEMILAPEDSISGMLRIPVVRERDAGVYTCRAVNELGDASAEIRLE	780


Bm86Rm*                     ------------------------------------------------------------	0
Bostaurus,hemicentin-2      VGHAPQLLELPQDVTVELGRSALLACRATGHPLPTITWHRGDDQPLGLRPGSRTGWPDSG	840


Bm86Rm*                     ------------------------------------------------------------	0
Bostaurus,hemicentin-2      VLFFESVVPEDQALYVCEAENVFGKVQAEAYLLVTGHVPPQIASSAPTVRVLERQPVSLP	900


Bm86Rm*                     ------------------------------------------------------------	0
Bostaurus,hemicentin-2      CIVLAGRPRPERRWLKAGMPLPPGSRHSIRADGSLHLDQALLEDAGRYSCVVSNTAGSHH	960


Bm86Rm*                     ------------------------------------------------------------	0
Bostaurus,hemicentin-2      RDVQLVVQVPPRIQPTATHHITNEGVPASLPCVASGVPTPTITWTKETNALASRDPHYNV	1020
```

```
Bm86Rm*                    ------------------------------------------------------------      0
Bostaurus,hemicentin-2     SKDGTLVIPRPSVQDAGAYVCTATNAVGFSSQEMRLSVNTKPRILVNGSHEADKPLRVTA      1080

Bm86Rm*                    ------------------------------------------------------------      0
Bostaurus,hemicentin-2     KAGDEVTLDCEAQGSPPPLVTWTKDSRHMLPITDRHHLLPPGSLHLAQAQVSDSGLYECT      1140

Bm86Rm*                    ------------------------------------------------------------      0
Bostaurus,hemicentin-2     ASNPAGSATQYYILRVQVPPQVQPGPRVLKVLVGEAVDLNCVAEGSPEPRVTWSKDGVAL      1200

Bm86Rm*                    ------------------------------------------------------------      0
Bostaurus,hemicentin-2     RGEGPEGSVHFAAIQTSHAGTYRCEASSSAGVDAWELDLRVLEPPHWGADETSGLLERVA      1260

Bm86Rm*                    ------------------------------------------------------------      0
Bostaurus,hemicentin-2     GENASLPCPARGTPKPQVTWRKGPSSEPLRDRPGLAVLDEGSLFLASVSPSDGGDYECQA      1320

Bm86Rm*                    ------------------------------------------------------------      0
Bostaurus,hemicentin-2     TNEAGSASRRAKLVVHVPPSLREDGRRANVSGMAGQSLTLECDANGFPAPEITWLKNGRQ      1380

Bm86Rm*                    ------------------------------------------------------------      0
Bostaurus,hemicentin-2     IPAVGSHRLLDGARALHFPRIQEGDSGLYSCRAENQAGTAQRDFDLLVLIPPSVLGAEAA      1440

Bm86Rm*                    ------------------------------------------------------------      0
Bostaurus,hemicentin-2     QEVVGLAGAGAELECRTLGVPTPQVEWTKDGQPVFLGEPRVQLQEDGQVLRITNSHLGDE      1500

Bm86Rm*                    ------------------------------------------------------------      0
Bostaurus,hemicentin-2     GWYQCVAFSPAGQQTKDFQLRIHAPPTIWGSNETSEVAVMEGHPVWFLCEARGVPTPDIT      1560

Bm86Rm*                    ------------------------------------------------------------      0
Bostaurus,hemicentin-2     WFKDGDPLVPSTEVVYTRGGRQLQLERAQGSDAGTYSCKASNAVGVVEKTTRLEVYVPPT      1620

Bm86Rm*                    ------------------------------------------------------------      0
Bostaurus,hemicentin-2     IEGTGEGPRVVKAVAGRPLTLECVARGYPPPTVSWYHEGLPVVDRNGTWLGAGGGVLSLE      1680

Bm86Rm*                    ------------------------------------------------------------      0
Bostaurus,hemicentin-2     SLGEASGGLYSCVASSPAGEAVLHYSVEVQVAPQLLVAEGLGQVTTLVGQSLYLPCYASG      1740

Bm86Rm*                    ------------------------------------------------------------      0
Bostaurus,hemicentin-2     SPVPTIQWLQNGHPAEELPGVHVTSEGTTLHIDHVELGHAGLFACQATNEAGTAAAEVEL      1800

Bm86Rm*                    ------------------------------------------------------------      0
Bostaurus,hemicentin-2     SVHELPSVVIVGGENITAPFLQPVTLRCAGTGVPTPSLRWWKDGVALAASGGSLQIEKVD      1860

Bm86Rm*                    ------------------------------------------------------------      0
Bostaurus,hemicentin-2     LKDEGTYTCVATNLAGESRKEVILRVLVPPNIEPGLVNKAVLENASVTLECLASGVPPPD      1920

Bm86Rm*                    ------------------------------------------------------------      0
Bostaurus,hemicentin-2     ISWFKGRQPISAWDRAMVSTDGRVLLIEQAQLSDAGSYRCVASNVVGSTELQYSLRVNVP      1980

Bm86Rm*                    ------------------------------------------------------------      0
Bostaurus,hemicentin-2     PRITLPPSLPGPVLLHSPVRLTCDAAGAPSPMLMWLKDGNPVSTAGTAGLQVFPGGRVLM      2040
```

```
Bm86Rm*                   ------------------------------------------------------------    0
Bostaurus,hemicentin-2    LASARVSDSGSYSCVAVSAVGEDRRDVVLRVHTPPSILGEEQNVSVVANESVALECRSHA    2100

Bm86Rm*                   ------------------------------------------------------------    0
Bostaurus,hemicentin-2    VPPPVLSWRKDGRPLEPRPGVYLSADKALLEVDRAEVGDTGRYTCEALNQVGRSEKHYNL    2160

Bm86Rm*                   ------------------------------------------------------------    0
Bostaurus,hemicentin-2    NVWVPPVFPSREPRTLTVTEGHPARLSCDCRGVPFPKISWKKDGQPLPGEGVSLAQVSAV    2220

Bm86Rm*                   ------------------------------------------------------------    0
Bostaurus,hemicentin-2    GRLLYLGRAQPAQEGTYTCECSNVAGNSSQDQQLVVYVPPRIAGPLEPYADVSVVQDEEA    2280

Bm86Rm*                   ------------------------------------------------------------    0
Bostaurus,hemicentin-2    SLECNATGKPAPRVTWERDGWPVGPESGLRLQNHGQSLHVERAQPAHAGRYSCVAENEAG    2340

Bm86Rm*                   ------------------------------------------------------------    0
Bostaurus,hemicentin-2    RAERRFSLSVLVPPELIGDLGPLTNVTATLHSPLSLFCEATGIPPPGIRWFRGEEPVSPG    2400

Bm86Rm*                   ------------------------------------------------------------    0
Bostaurus,hemicentin-2    ENTYLLAGGWMLKLTRAQEQDRGLYSCLASNEAGEARRDFSVEVLVPPSIEKEDVEDTVK    2460

Bm86Rm*                   ------------------------------------------------------------    0
Bostaurus,hemicentin-2    VPEGEMAHLMCNVSGHPQPKVTWFKDGQPLASGDAHHVSPDGALLWVLHANLSSAGHYAC    2520

Bm86Rm*                   ------------------------------------------------------------    0
Bostaurus,hemicentin-2    VAANAIGEKTKHFQLSVLVVPTILGVTEDSMDEEVTVTINNPISLICETRAFPTPTITWM    2580

Bm86Rm*                   ------------------------------------------------------------    0
Bostaurus,hemicentin-2    KDGVPFEALNNIQLLPAGTHGLQILNAQKEDAGQYTCVVTNELGEAMKNYHVEVLIPPSI    2640

Bm86Rm*                   ------------------------------------------------------------    0
Bostaurus,hemicentin-2    SKDDLFGEVSMKEVKTKVNSTLTLECESWAVPPPTISWYKDGQPVTPNQRVRILGEGRLL    2700

Bm86Rm*                   ------------------------------------------------------------    0
Bostaurus,hemicentin-2    QIQPTQVSDSGRYLCVATNVAGEDDQDFNVLIQVPPIFQKVADATADFETPFWGEEARGG    2760

Bm86Rm*                   ------------------------------------------------------------    0
Bostaurus,hemicentin-2    VTEYREIVENNPAYLYCDTNAVPAAELTWYRGDRPLSIEDGVSVLQGGRVLQIPLVRAED    2820

Bm86Rm*                   ------------------------------------------------------------    0
Bostaurus,hemicentin-2    AGRYSCKASNEVGEDWLHYELLVLTPPVILGETEELVEEVTVNANSTVSLHCPVLGNPAP    2880

Bm86Rm*                   ------------------------------------------------------------    0
Bostaurus,hemicentin-2    TISWLQNGLPFSPSPRLQVLENGRILQVSTAEVADAASYMCVAENPAGSTEKLFTLRVQV    2940

Bm86Rm*                   ------------------------------------------------------------    0
Bostaurus,hemicentin-2    PPRIAGLNPEQITATLNSSVSLPCDVRAHPSPEVTWYKDGWTLSLGEEIFLLPGTHTLQL    3000

Bm86Rm*                   ------------------------------------------------------------    0
Bostaurus,hemicentin-2    TRVQLLDSGMYICEALNAAGRDHKLVQLSVLVPPTFRQAPSRPQDVVLVSAGDKAVLSCE    3060

Bm86Rm*                   ------------------------------------------------------------    0
Bostaurus,hemicentin-2    TDAFPEPTVAWQKDGQPLVLPERIQALLGGQRLEIQDAQVSDKGLYSCRVSNAAGEAMRA    3120

Bm86Rm*                   ------------------------------------------------------------    0
Bostaurus,hemicentin-2    FSLTVQVPPTFENPETEMVSRVAGSPLVLSCDVTGVPAPAVTWLKDRMPVESSMARGVVS    3180

Bm86Rm*                   ------------------------------------------------------------    0
Bostaurus,hemicentin-2    RGGRLQLSHLQPDQAGTYTCVAENAQAEAHKDFVVAVLVAPRIQSSGTTQEHSVLEGQDV    3240

Bm86Rm*                   ------------------------------------------------------------    0
Bostaurus,hemicentin-2    QLDCEVDGQPPPYVVWLKDGSPLDESVGPYLWFYLNGSSLVLKDLRASDSGAYTCVARNA    3300

Bm86Rm*                   ------------------------------------------------------------    0
Bostaurus,hemicentin-2    AGEDAKLYTVNVLVPPTIEQGPEGAGTLVHRLGDLVSMACPVRGSPPIHVSWLKDGLPLP    3360

Bm86Rm*                   ------------------------------------------------------------    0
Bostaurus,hemicentin-2    LSHRTHLHSSGRTLRISQVQLADSGTFTCVAASPAGVANRHFILQVLVPPVLEPAEFQND    3420
```

```
Bm86Rm*                 ------------------------------------------------------------    0
Bostaurus,hemicentin-2  VAVVRGSLVFLPCEARGSPLPFVSWVKDGEPLLPQSLEHGPGLLLEAAEAGHAGTYSCVA    3480

Bm86Rm*                 ------------------------------------------------------------    0
Bostaurus,hemicentin-2  VSEAGEARRHFQLTVMDPPYIEDSDQPEELLLTPGTPLELLCDARGTPTPNITWHKDGRA    3540

Bm86Rm*                 ------------------------------------------------------------    0
Bostaurus,hemicentin-2  LSWPEDGQGARRVLRVEGVQVSDAGLYTCLAHSPAGEVEKSFRIRVQAPPNVIGSHGPRF    3600

Bm86Rm*                 ------------------------------------------------------------    0
Bostaurus,hemicentin-2  VVGLAPGQLVLECSVEADPAPEIEWHREGVLLQADAHTQFPEEGRFLQLQALSTADSGDY    3660

Bm86Rm*                 ------------------------------------------------------------    0
Bostaurus,hemicentin-2  SCTAHNAAGSTSLAFHVEVHTVPAIQSGPPTVNASVNQTTLLPCQADGVPQPLVSWLKDG    3720

Bm86Rm*                 ------------------------------------------------------------    0
Bostaurus,hemicentin-2  VPLDPGSTRFQVLPEGSLRIQPVLAEDAGHYLCLASNPAGSDRQGRDLQVFEPPAIAPGP    3780

Bm86Rm*                 ------------------------------------------------------------    0
Bostaurus,hemicentin-2  SNLTLTAYTPASLPCEASGSPKPLVAWWKDGQKLDFRLQQGAYRLLPSNALLLEEPSPQD    3840

Bm86Rm*                 ------------------------------------------------------------    0
Bostaurus,hemicentin-2  SALFECVVSNGVGEARKLYWVTVHVPPAIADDQTDFTATIMAPVVLTCHSTGVPAPVVSW    3900

Bm86Rm*                 ------------------------------------------------------------    0
Bostaurus,hemicentin-2  SKAGTRLGVRGNGYRVSPSGALEIGQALPIHTGRYTCAARNAAGMAHKHVVLTVQASPVV    3960

Bm86Rm*                 ------------------------------------------------------------    0
Bostaurus,hemicentin-2  KPLPSVVWAVAKEEVLLPCEASGIPRPSIAWQKEGLSVPAGAGTQVLPGGQLRIIHVSPE    4020

Bm86Rm*                 ------------------------------------------------------------    0
Bostaurus,hemicentin-2  DAGNYFCLAQNSAGSAVRKTRLVVQVPPAIKTGLPDLSITEGAHALLPCTATGSPEPKVT    4080

Bm86Rm*                 ------------------------------------------------------------    0
Bostaurus,hemicentin-2  WEKDGQPVSGAKGKFTIQPSGELLVKNSESQDAGTYTCTAENAVGRARRRVHLTILALPV    4140

Bm86Rm*                 ------------------------------------------------------------    0
Bostaurus,hemicentin-2  FTTLPGDRSLRLGDKLWLHCAARGSPTPQIGWTVNNRLVTEGVSEQDGGSTLQRVAVTRE    4200

Bm86Rm*                 ------------------------------------------------------------    0
Bostaurus,hemicentin-2  DSGTYVCWAENRVGRVQAVSFVHVKEAPVLQGESFSYLVEPVGGSIRLDCVVRGDPTPDI    4260

Bm86Rm*                 ------------------------------------------------------------    0
Bostaurus,hemicentin-2  YWIKDGLPLRGSRLRHWLQNGSLIIRRTEMDDAGQYQCLAENELGAVEKVVVLALQSVPV    4320

Bm86Rm*                 ------------------------------------------------------------    0
Bostaurus,hemicentin-2  FQVKPQDVTVRSGDSVALRCQASGEPAPTMEWLRAGQPVRASQRLQTLPDGSLWLERVEA    4380

Bm86Rm*                 ------------------------------------------------------------    0
Bostaurus,hemicentin-2  RDAGPYECVAHNLLGSATARAFLVVRGEPRGSRGSMIGVINGQKFGAAVLNTSVQQEARS    4440
```

```
Bm86Rm*                     ------------------------------------------------------------    0
Bostaurus,hemicentin-2      GVPTIHSSISHIPTSVGPLMRVLVVTIAPIYWALAGESGDALNGHSLTGGSFQQESHVEF    4500


Bm86Rm*                     ------------------------------------------------------------    0
Bostaurus,hemicentin-2      ATGELLTMTQVARGLDPNGLLLLDVVVNGIVPESLADADLQVQDFQERYVQTGPGRLFVG    4560


Bm86Rm*                     -----------------------------------MRGIA-----------L-FVAA---    10
Bostaurus,hemicentin-2      STQSFLQDSLRSFLRCNHSIRYSAAQGPQPQLVQHLRASAISSAFDPEAEALHFQLSTAL    4620
                                                               :*. *           * *  :

Bm86Rm*                     --VSLIVECTAESSICSDFGNEFCRNA-ECEV--------VPGAEDDFVCKCPRDNMYFN    59
Bostaurus,hemicentin-2      QAEENEVGCPE--GFELDAQGEFCVDRDECSDSPRPCSHSCHNAPGRFSCSCPVGFALA-    4677
                               .  * *    .:  *  .*** :  **.           .* . * *.** .

Bm86Rm*                     AAEKQCEY---------------------------------------------KDTC--K    72
Bostaurus,hemicentin-2      RDERTCRDVDECAWEADLCQEDQRCVNLLGSYHCLPDCRPGFRVAADGASCEDVDECLEQ    4737
                              *: *.                                               * *  :

Bm86Rm*                     TRECSYGRCVESNPSKGSCVCEASDDL---TLQCKIKNDFATDCRNRGGTAKLRTDGF-I    128
Bostaurus,hemicentin-2      TDECHYNQTCENIPGGHRCGCPRGYQVQDPGLPCLDIN----ECLQLPGPCAYQCHNLQG    4793
                            * ** *.:  *. *.   * *  . ::    * *   *    :* :  * .  : ..:

Bm86Rm*                     GATCDCGEWGAMNKTTRNCVPTTCLRPDLTCKDLCE-KN-----LLQR---DSRCCQGWN    179
Bostaurus,hemicentin-2      GYRCLCPPGQTLLRDGKTCTPLERSGPNVTTVSHVSHRDPLVAWLQARGPKPRGSYHAWV    4853
                            *  * *    :: :  :.*.*     *::*  .  . ::     *  *      . :.*

Bm86Rm*                     TANC--SAAPPADSYCSPGSPKGPDGQCKNACRTKEAGFVCKHGCRSTDKAYECTCPSGS    237
Bostaurus,hemicentin-2      SLRPGPRALSMGRAWCPPGFIR-QNGVCTDL-DECRVRNLCQHACRNTEGSYQCLCPAGY    4911
                            : .    *   . ::* **  :  :* *.:     ..  :*:*.**.*: :*:* **:*

Bm86Rm*                     TVAEDGITCKSISYTVSCTVEQKQTCRPTEDCRVQKGTVLCE-----CPWNQHLVGDTCI    292
Bostaurus,hemicentin-2      RLLPSGKNCQDINECE----EDGIECGPSQMCFNTRGSYQCVDTPCPATYRQGSSPGTCF    4967
                             :  .* .*:.*.       *:   * *:: *   :*:  *      . :.*    .**:

Bm86Rm*                     SDCVDKKCHEEFMDCGVYMNRQSCYCPWKSRKPGPNVNINECLLNEYYYTVSFTPNISFD    352
Bostaurus,hemicentin-2      RRCSQDC-----------------------SSGGPSTLQYK-LL-----PLPLGVRALHD    4998
                              * :.                         . **..   : **      : :  .  .*

Bm86Rm*                     SDHCKRYEDRVLEAIRTSIGKEVFKVEILNCTQDIKARLIAEKPLSKYVLRKLQACEHPI    412
Bostaurus,hemicentin-2      VARLAAFSEAGIPANRT------------------ELSVLEPDPRSPFALRPLRAGHGAI    5040
                              :   :.:  : * **                  :  ::  .* * :.** *:* .  *

Bm86Rm*                     GEWCMMYPKLLIKKNSATE-------IEEENLCDSLLKNQEAAYKGQNKCVKVDNLFWFQ    465
Bostaurus,hemicentin-2      ------YTRRALTRAGLYRLTVRAAAPRHQSVFVLLIAVSPYPY----------------    5078
                                  * :  :.: .  .        ..:.:   *:  .   *

Bm86Rm*                     CADGYTTTYEMTRGRLRRSVCKAGVSCNENEQLECANKGQICVYENGKANCQCPPDTKPG    525
Bostaurus,hemicentin-2      ------------------------------------------------------------    5078


Bm86Rm*                     EIGCIERTTCNPKEIQECQDKKLECVYKNHKAECKCPDDHECSREPAKDSCSEEDNGKCQ    585
Bostaurus,hemicentin-2      ------------------------------------------------------------    5078


Bm86Rm*                     SSGQRCVMENGNAVCKEKSDATTASTTTTKAKDKDPDPEKSSAAAVSATGLLLLLAATSV    645
Bostaurus,hemicentin-2      ------------------------------------------------------------    5078


Bm86Rm*                     TAASL       650
Bostaurus,hemicentin-2      -----       5078
```

Bm86 against other tick species

```
CLUSTAL O(1.2.4) multiple sequence alignment


Bm86Is,XP_040071062.1      MRPLIVFALAVFAWDARTSPVLGLALQNEANEDQ--TIHEDPKGICKRVGSALCGDLAKC 58
Bm86Ir,ADR01316.1          MKPLLVFALAVFAWNARTSPVLGLALQNEANEDQ--AIHEDPKGICKRVGSSICGDLATC 58
Bm86Hl,BAF56919.1          MRICVGFLAGVLLISG-------TVADGDV---GVPEEAVSQGVACESFGNRFCN-GPCK 49
Bm86Da,XP_050035013.1      MRSLTLFVAAISLIGG-------CASKDIPPDALQDPPAPPTPDACSHFGNKFCGTKKCE 53
Bm86Hd,ADY76577.1          ----------------------------------------SSICSDFGKQFCQSAECE 18
Bm86Rs,ABN11119.1          MRSLALFVAAVSLIGG-------CAAQ---------------SSVCSDFGNEFCRNAECE 38
Bm86Rm*                    MRGIALFVAAVSLIVE-------CTAE---------------SSICSDFGNEFCRNAECE 38
Bm86Ra,ACL27210.1          MRGIALFVAAVSLIVE-------GTAK---------------SSICSDFGNEFCRNAECE 38
                                                                        *. .*. :*

Bm86Is,XP_040071062.1      VALPAIDSFQCSSPKGG-FYDANDKTCKHWQSCGPLPCKIGYCDDGNGSKKRHCKCGGIP 117
Bm86Ir,ADR01316.1          VALPAIDSFQCSCRNGE-FYDANDKRCKHWQSCGPLPCKLGNCIDENGSKKRHCSCRAIP 117
Bm86Hl,BAF56919.1          QTPGSNDTFSCVCPSDEFYFDAVQRICLYKTSCVTQKCSIGFCKD-G-RNKVFCNCEGIP 107
Bm86Da,XP_050035013.1      VVPDRTDAFVCRCERNDMYYDAAEGTCLFKRTCKTTECTYGTCIEHG-PNKASCGCPKVD 112
Bm86Hd,ADY76577.1          VIPGTGEDFVCKCPNDDMYYNAAEKQCEYKRTCKTVECSYGYCYEIR-PGRTGCGCQGVD 77
Bm86Rs,ABN11119.1          VVSGAEHDFVCRCPRDDMYYNAAEKHCEYKSTCKTKECSYGRCTEIS-PSKAVCACDGVD 97
Bm86Rm*                    VVPGAEDDFVCKCPRDNMYFNAAEKQCEYKDTCKTRECSYGRCVESN-PSKGSCVCEASD 97
Bm86Ra,ACL27210.1          VVPGAEDDFVCKCPRDNMYFNAAEKQCEYKDTCKTRECSYGRCVESN-PSKASCVCEASD 97
                                 . * * .  .  :::* :  * .  :*    *. * * :     :  * *

Bm86Is,XP_040071062.1      GLTEFCKINKEAKFKCQEYGATAVLKGDE---VSCDCGPAE---KFINKKCVPVACLNFN 171
Bm86Ir,ADR01316.1          GLTEFCRINNDAKFKCEEYGATAVLKGDK---VTCDCGPAK---KLINKKCVPVACLNFT 171
Bm86Hl,BAF56919.1          YMTLNCKVQKFYQDECEERGGQALMPTRPAEGPRCACGEWAKADIEN-KKCVPSTCLRPT 166
Bm86Da,XP_050035013.1      SLSLNCKIRDWFIDDCKRKGGTAVLDKEWSFGAKCACGEWAAADRSK-DKCVPTTCLRPD 171
Bm86Hd,ADY76577.1          TLTLKCGIQEWFANDCGRKGGTAVLRTDGFLGARCDCGEWGKMDKAQDGKCVPTTCIRPD 137
Bm86Rs,ABN11119.1          SLTLRCTIQNWYADECRRMGGTAKLRTDGFLGARCDCGEWSAMDRNK-SKRVPTTCLRPD 156
Bm86Rm*                    DLTLQCKIKNDFATDCRNRGGTAKLRTDGFIGATCDCGEWGAMNKTT-RNCVPTTCLRPD 156
Bm86Ra,ACL27210.1          DLTLQCKIKYDYATDCRNRGGTAKLRTDGFIGATCDCGEWGAMNKTT-RNCVPTTCLRPD 156
                            ::  * :.     .* . *. * :         * **           : ** :*:.

Bm86Is,XP_040071062.1      MTCEELCQKKLLDKDDRCCQGWNIENCSKTPDETRYCKPGYISR-NDSCVDACTAKVADP 230
Bm86Ir,ADR01316.1          MSCEELCQKKLLDKDDRCCQGWNVEDCSKTPDEKGYCKTGYISR-NNSCVDACTANVADP 230
Bm86Hl,BAF56919.1          VTCKELCEKGLLGKDPRCCQGWNETDCTREPENGTYCSPGSLINKQGRCQDACSARQTNL 226
Bm86Da,XP_050035013.1      LTCKELCEKNLLEKDNRCCQGWNSADCSAAPPNDTYCSPGSIMRANGKCQDACTAREDKF 231
Bm86Hd,ADY76577.1          LTCKDLCEKNLLGKDTRCCEGWSSTDCSVVPHEDTYCSPGSIKGEDGKCKDACTTKEALL 197
Bm86Rs,ABN11119.1          LTCKYLCENDLLEKDSRCCQGWNSANCSAAPEEGSYCSPGHLKGKDGKCQDACSAKEATF 216
Bm86Rm*                    LTCKDLCEKNLLQRDSRCCQGWNTANCSAAPPADSYCSPGSPKGPDGQCKNACRTKEAGF 216
Bm86Ra,ACL27210.1          LTCKDLCEKNLLQRDSRCCQGWNSPKCSA--PADSYCSPGSPKEPDGQCKDACKTKEAGF 214
                           ::*: **:: ** :* ***:**.  .*:       **. *     :. * :** :.

Bm86Is,XP_040071062.1      LCPDGCKSS-ATDRPFQCICKKGFQLAEDGITCTER---RVCNEEEKKRCRKDQICGIVN 286
Bm86Ir,ADR01316.1          LCPDGCKSP-ATGRPFECKCKKGFQLAEDGLTCTER---RVCDKDEKKRCREDQICGIVN 286
Bm86Hl,BAF56919.1          ICPYGCQPTPGGKTAYQCWCPLRKEVAEDGVSCKEVPKVRLCREDEQKACRPGQSCEVRD 286
Bm86Da,XP_050035013.1      VCSDGCRRIESSARAYECRCKINYVVAEDGITCKAVRMAHACSEEQKKTCLPSQTCLVDK 291
Bm86Hd,ADY76577.1          LCKEGCIKGQKPGKAYECRCRQGYEIAEDGITCKRVPGIVDCTEEQKAACLPGQQCRVHK 257
Bm86Rs,ABN11119.1          VCKHGCKRSSKPMRAYECRCTSDDEVAEDGITCKGIPYTGGCSDEQKKTCRPSENCLLKK 276
Bm86Rm*                    VCKHGCRSTD---KAYECTCPSGSTVAEDGITCKSISYTVSCTVEQKQTCRPTEDCRVQK 273
Bm86Ra,ACL27210.1          VCKHGCRSTD---KAYECTCPRGFTVAEDGITCKSIPYTGGCTVEQKQTCRPTENCRVHA 271
                           :*  **         ::* *     :****::*.       *  :::  *   : * :

Bm86Is,XP_040071062.1      EEVTCSCKSHQQEKDGVCTNECFVKKCSDPFANCEVYLGTEKCFCAR----PLFPAGNNT 342
Bm86Ir,ADR01316.1          AEAKCSCKVHQQEKDGVCTDQCLVKECSSPFANCEVYLGMETCFCTP----PLSSIGNSK 342
Bm86Hl,BAF56919.1          EKIICRCRLHEHLVDGVCTEKCRPNNCHEHFLKCVISNGKEQCRCPWKDWRK--VFINDE 344
Bm86Da,XP_050035013.1      DKITCACPRNHQLVNGECTSRCTENKCHENFTDCNVYMEKQSCSCPWTTRKPD-RIFIRE 350
Bm86Hd,ADY76577.1          GNSVCECPSDQQLLDGKCASECVEGRCHENFTDCGVYMNKQGCYCPWTTRKPPRGVEISR 317
Bm86Rs,ABN11119.1          EKVVCECPMRQHLVNDECISDCVDDKCHENFMECGVYMWKQNCYCPWRTRKPRPNVFINE 336
Bm86Rm*                    GTVLCECPWNQHLVGDTCISDCVDKKCHEEFMDCGVYMNRQSCYCPWKSRKPGPNVNINE 333
Bm86Ra,ACL27210.1          GKVLCVCPWNQHLVGDKCIGDCVENKCHEEFTDCGVYMNRQSCYCPWKSRKPGPNVNINE 331
                               * *   .:  .. *   *   .* . * .* :    : * *
```

```
Bm86Is,XP_040071062.1     CGLEKYSYILTFRTNDTSPYTDDFCESKKEDIQKALKTLFGAQLVDDTLLSCKEDFVIRL 402
Bm86Ir,ADR01316.1         CGLGKYSYILRFRTNDTSPYSDDFCKSKEEDIRKALRTLFGAQLVDDTFLSCEEDIVIRL 402
Bm86Hl,BAF56919.1         CTLKRYYYTVSFKPNI--SLENDDCDFYGQRVFQAMRTALGSRVHMVQILSCTKKIEARL 402
Bm86Da,XP_050035013.1     CTLKEYYYTVSFTPNI--SLDADNCNLYEGRVLEAIKTSIGTEVFKVEILNCTQKIKARL 408
Bm86Hd,ADY76577.1         CVLNEYYYTVSFTPNI--SLNSDHCEWYEARVLEAIRTAIGEEVFKVEILNCTQDIKARL 375
Bm86Rs,ABN11119.1         CVLNEYYYTVSFTPNI--SLNSDSCDSYAARVLDAIRTSIGSEVFKVEILNCTEDIKARL 394
Bm86Rm*                   CLLNEYYYTVSFTPNI--SFDSDHCKRYEDRVLEAIRTSIGKEVFKVEILNCTQDIKARL 391
Bm86Ra,ACL27210.1         CLLNEYYYTVSFTPNI--SLDSDHCDWYEDRVLEAIRTSIGKEVFKVEILNCTQDIKARL 389
                          * * .* * : *  *       * *.     : .*::* :* .:    :*.* :.:  **

Bm86Is,XP_040071062.1     AFSQKQDPAVLKRIETCQYLRGDVCIFPPRLYIKEGSA-KLEEEDLCTEFFQKQLNQSNG 461
Bm86Ir,ADR01316.1         TFSQKQDPAVLKRIETCQYLRGDVCIFPPRLYIKEGSA-KLEEEDLCTEYFQEQLNKSKG 461
Bm86Hl,BAF56919.1         IFNEPLPELLRKKLQMCEYQEGEYCMLYPSLPIEKGSATEIEEENLCESVLKEQEVAYNG 462
Bm86Da,XP_050035013.1     ISAKPLSKYLLKKLQTCEHPDGDLCMLYPKLPIKKDTATEIEEENLCDSLLKGQEEAYNG 468
Bm86Hd,ADY76577.1         IASKPLSKHVLKKLQACEHPVAEFCMLYPKLPIKKGSVTGIEEENLCESLLKNQEKAYKG 435
Bm86Rs,ABN11119.1         IASKPLSKHVLKRLQACEHPVGDLCMLYPKLPIKKGSATEIEEENLCDSILKTQENGYKG 454
Bm86Rm*                   IAEKPLSKYVLRKLQACEHPIGEWCMMYPKLLIKKNSATEIEEENLCDSLLKNQEAAYKG 451
Bm86Ra,ACL27210.1         IAEKPLSNHVLRKLQACEHPIGEWCMMYPKLLIKKNSATEIEEENLCDSLLKNQEAAYKG 449
                             :     : :::: *::  .: *:: * * *::.:.  :***:** . :: *    :*

Bm86Is,XP_040071062.1     MYVCQKVGDNYSFKCRDGLFSYNEVTSGRLTRWSCADKREDIPPASSES----------- 510
Bm86Ir,ADR01316.1         MYVCQKVGDNYLFKCRDGLFSYNKVTSGRLTHWSCADKREDIPPESSES----------- 510
Bm86Hl,BAF56919.1         TNECVKDGNIFWFKCAPGFKEVNQQSVGRLRRSICEPGTEDPTVEPADTDD--------- 513
Bm86Da,XP_050035013.1     QNECVKFEDFFWFKCAAGFREVDRVTRGRLRRSVCEPGVSCTQDEELECASKGQICVYEN 528
Bm86Hd,ADY76577.1         VNKCAKVGDLYWFQCADGYRAVNEVARGRLRRSVCKAGVSCTEKEQLDCANKGQICVFEN 495
Bm86Rs,ABN11119.1         QNKCVKVDNFFWFQCADGYRAVDEIERGRLRRSVCVAGLSCSDKEQLECVNKGQICVYEN 514
Bm86Rm*                   QNKCVKVDNLFWFQCADGYTTTYEMTRGRLRRSVCKAGVSCNENEQLECANKGQICVYEN 511
Bm86Ra,ACL27210.1         QNKCVKVDNLFWFQCADGYTTTYEMTRGRLRRSVCKAGVSCNENEQSECADKGQIFVYEN 509
                             * *  : : *:*  *     .   *** :  *    .       :

Bm86Is,XP_040071062.1     --PK-------------------------------------------------------- 512
Bm86Ir,ADR01316.1         --PKSVTDKEPQPGTEGSGTKNTPVPDVTD-KEP---------------------QPGTE 546
Bm86Hl,BAF56919.1         --PNVE-P-------AGAD----------------------------------------- 522
Bm86Da,XP_050035013.1     EEPKCQCPPGMVTGQGGCSAA--VVPESCNDEETNKCRSSGQRCVMENQKAVCKEASNTP 586
Bm86Hd,ADY76577.1         EKPNCQCPPGTVFGQAGCAAR-----TTCNPKEIRECEDEKKECVYRDQKAECKCPKGTV 550
Bm86Rs,ABN11119.1         EKANCQCPPGTVAGQAGCTAR-----TTCNPKEVRECQDQKRECVYKDQKAECKCPEGTV 569
Bm86Rm*                   GKANCQCPPDTKPGEIGCIER-----TTCNPKEIQECQDKKLECVYKNHKAECKCPDDHE 566
Bm86Ra,ACL27210.1         GKANCQCPPDTKPGEIGCIER-----TTCNPKEIQECQDKKLECVYKNHKAECECPDDHE 564
                             :

Bm86Is,XP_040071062.1     ------------LDPCSPS---PC-VNGGVCKPGTGKTFTCEC-----------PKGFKG 545
Bm86Ir,ADR01316.1         GTGTKS--TPVPDDPCSSS---PC-LNGGVCKPGTGKIFTCEC-----------PKGFKG 589
Bm86Hl,BAF56919.1         --------DPA-VEPATQESKGDPATKGTTQ------H-ASKETNP-------EPASKGT 559
Bm86Da,XP_050035013.1     VT---EVVDPA-PSQCNEEMRKKCTKKGAECVTVDGKA-ICRCPEGKVE-------TPQG 634
Bm86Hd,ADY76577.1         DDGQRCSGEPA-EASCTEENIAACRSKGQRCAIENGRP-VCKETSGVTTAEATTTQATKA 608
Bm86Rs,ABN11119.1         DDGDGC---SA-EESCSEDEIGKCRSKGQRCVMENGKP-VCKEISDATTATTTTTKAKDK 624
Bm86Rm*                   CS--RE---PA-KDSCSEEDNGKCQSSGQRCVMENGNA-VCKEKSDATTASTTTTKAKDK 619
Bm86Ra,ACL27210.1         CY--RE---PA-KDSCSEEDNGKCQSSGQRCVMENGKA-VCKETSEATTAATTTTKAKNK 617
                                         .. .       .*            ..

Bm86Is,XP_040071062.1     NLCEEKNSGVSVQAPVTAILGIAMSLLLLR------------   575
Bm86Ir,ADR01316.1         NLCEEKNSGVSVQAPVTTILGIAISLLLLR------------   619
Bm86Hl,BAF56919.1         TAEPEKSAGSSPFKSHF------LELLLLYG-GVVTLLQILH   594
Bm86Da,XP_050035013.1     CSDPGPSSAVSVSST---------TLLLLAAMTVAAAAAA--   665
Bm86Hd,ADY76577.1         DPDPGKSGGVAVSAT---------TLL---------------   626
Bm86Rs,ABN11119.1         NQNPGKSSAAAISAT---------WLLLLIAATSVAAA----   653
Bm86Rm*                   DPDPEKSSAAAVSAT---------GLLLLLAATSVTAASL--   650
Bm86Ra,ACL27210.1         DPDPGKSSAAAVSAT---------GLLLLLAATSVTAASL--   648
                                ... :              **
```

VgR against Bos Taurus

```
CLUSTAL O(1.2.4) multiple sequence alignment


VgRRm*          ----MKFTACAVLAVVALYLVGNVFSECPQAWFDCGNGRCIAMFWRCDGQNDCGNHKDET     56
LDLP,[Bos       MERWAAAAACTLLLAFAACLAPASGGGCRSNEFLCQPGLCITASWRCDGTRDCPNGADEI     60
                       :**::* ..*  *.    . * .  * *  * **:  ***** .** *  **

VgRRm*          GCSATHSRCPADKFACRDSSYCVPQIWVCDGEADCHDSSDEQD-CHSSN--CTGFRCHNN     113
LDLP,[Bos       DCPT--SSCHSNQFLCPNEQLCIPESWVCDGEDDCNDGADERRHCPGITCSSRQFTCENG     118
                .* :  * * :::* * :.. *:*: ****** **:*.:**:  * . .  .  * *.*.

VgRRm*          ECIPAHWRCDQTEDCADASDELDCGGVQNSSTTT--------------------------     147
LDLP,[Bos       ECIPGEFRCDHSTDCLDGTDEKNCRYPVCEQLTCADGACYNTSQRCNGQVDCRDASDERN     178
                ****..:***:: ** *.:** :*     .. *

VgRRm*          PTPRC------------------------------------DVDQGRFPCLDGQCLL-     168
LDLP,[Bos       CTHRCTRTEFQCGSGQCIPRTYVCDHEIDCEDGSDEHSCPAYRTCKGNEFTCPNGVCIAQ     238
                 * **                                        . ..* * :* *:

VgRRm*          ---------------------------------------------PSKVCDGRKDCSDGAD     184
LDLP,[Bos       SWVCDGESDCVDNADEDGCESKINRTFECYPNEWACPKSGKCIPISKVCDGTLDCPGGED     298
                                                             ******  ** .* *

VgRRm*          E-----GSFCKVNECSQKKCSQGCFVATNGSTCYCNAGFRLLADH-ISCADVDECAEDPH     238
LDLP,[Bos       ESNITAGQQCDVNLCPSLGCEYQCHRSPGGGMCYCPSGFIVNQNRTNNCVDFDDCQI-WG     357
                *     *. *.** * .  *.  *. : .*. *** :** :  ::  .*.*.*:*

VgRRm*          VCSHGCINSPGSYSCHCLEGYQLTDNSFCKARDP--EPLLVFSTTKEIRGLWLRSNRYFE     296
LDLP,[Bos       VCDQLCEDRIGHHRCNCVEGYVLERQKHCRANSSFGEAFVIFSNGRNLLKGSIRGNNFQI     417
                **.: * :  * : *:*:*** *  :..*:*..   * :::**. :::    :*.*.:

VgRRm*          I--HPAEAQAVGVEFDSDQHRVFWTDVSTRRSSIHSCRLDGSDFKTLFSAEKTLLEDLSL     354
LDLP,[Bos       LAESQNRGLAVGVDFHYRLRRVFWTDIVQKK--VFSVDISGSRIREVLGVSIEDPENLAV     475
                :     .. ****:*.   :******:  ::  :.*  :.** :: ::...    *:*::

VgRRm*          DWVANNLYITDSLVKRILVCTTDGASCSALITDSVDSPRAIIVNPPQKVVYWTDWGS---     411
LDLP,[Bos       DWVNNKLYIVETRVNCIDVVDLDGSHRITLISEYLGHPRGIAVDPTVGYLFFSDWQNVFG     535
                *** *:***.:: *: * *   **:   :**:: :. **.* *:*    ::::** .

VgRRm*          RPAIMHASMDGTNIQQLVSTDLGWPNGLTLDHTTNRLYWCDAKLSSLEYLELSTLKRDVV     471
LDLP,[Bos       VPRIERAYMDGSNRKDLVTTKLGWPGGITLDLVSKRVYWVDSRFDYIETVTYDGLKRMTV     595
                 * * :* ***:* ::**:*.****.*:*** .::*:** *:::. :* :  . *** .*

VgRRm*          MHE--EVFHPFALAVFEDTVYWSDWASYSLDSSNKRTGKQHHRVLRENGHHIMGVHVYHP     529
LDLP,[Bos       IHGGADIPHPFSISLFEGQLFFTDWTKMAVLKANKFKET-NPRLYYRSSLKPFGVTVYHG     654
                :*   :: ***:::**. ::::**:. :: .:** . . : *:  ... : :** ***

VgRRm*          VLRQRGIQNPCW--DNPCDHICVLSAD------SYMCLCRIGYKLSANKHSCAVTKDFSF     581
LDLP,[Bos       L-RQPYARNPCAHENGGCQHICVLSHRTFNGGLGYRCKCRLGYIPDWDDYHCVAAERFLL     713
                : **   :***   :. *:******        .* * **:**  . :.: *..:: * :

VgRRm*          VIVAEEDLVYKIDLNKVGAPVP------VTLPVHN-LGIISALTFDWANQTLHYSDNRHS     634
LDLP,[Bos       ---------FSSNLAVRGIPLTLSHQTEVILPVTGSSSIFLGIDFDAREKAIFFSDTKKN     764
                         :. :*   * *:       * *** .  .*: .: **  ::::.:**.::.

VgRRm*          VLSAINVKSFE-QWTVHDHIGSVFGIDFDVTHQLLYWVDADKYTLEACHANGSGHVIIRD     693
LDLP,[Bos       IIYRQKLDGTGREIITANRVPAVQSLSFDWISRNLYWTDSSYRSVSVIRLGDKSRRTIIQ     824
                ::   ::..   :  . ::: :* .:.**   : ***.*:.  ::.. : ....:  * :

VgRRm*          DLHRPVGVALYPFAGVLFVLSAGDKPTITSYTMDGQNPRVLPLSTLLLPVSISVDLVART     753
LDLP,[Bos       NLNNPQSIVVHPTAGYIFFSVWYRPAKILRAWADGSNILPIVNTTLGWPSGLSIDWSSSR     884
                :*:.* .:.::* ** :*.       .*     **.*   :  :**  * .:*:*  :

VgRRm*          LVWADAVRGTIESLDLQKVFTGTPFIVQQVKAHISSVCAAHNE---IHWTSRDKASLEYI     810
LDLP,[Bos       LYWVDAFFDKIEHSNLDGSDRKALTNVHQLTHPFG-LAVSQDYIYVTDWRR---GIIRFG     940
                * *.**. ..**  :*:     :   *:*:.  :. :..:::     .*     . :.:

VgRRm*          DRSSEPSVHRHVSLRSTR-----NGTFSRRVIVAAQVPPFAPGPCGLNNGGCSHTCLPVR     865
LDLP,[Bos       KYNP----GQSIILRSGVGSVMRAKVYDSRVQTGS----NACSRPTNPNGDCSHFCFPVP     992
                . .      : : ***          .:. ** ..:     * .     **.*** *:**

VgRRm*          TTDRSCFCPPGMALNADNRTCRVETSTCRPHELPCAGSCIAAVNWCDGHKDCSDNADEES     925
LDLP,[Bos       NSQRVCGCPYGMSLASDHLTCVENASREPP------------------------------     1022
                .::* * ** **:* :*: **  ::*   *

VgRRm*          CGSATCPATDFSCSNGRCIEKEWQCDGYNDCGDSSDERNCTVT--TCASHQYTC-RGGVC     982
LDLP,[Bos       --VEQCGTLSFSCHNGRCVPLQYRCDGFDDCLDNSDEAQCTTSNATCSPLAFECKREGHC     1080
                     * : .*** ****:  :::***::** *.*** :**.:  **:   : * * * *
```

```
VgRRm*          LPLYWRCDGSEDCPDGDDELNCSSV---RCPSAHSRCDNGQCIPQDWTCDGHSDCSDSSD     1039
LDLP,[Bos       IPSMWRCDGEDDCLDGSDEQNCPTRAPTSCRADQFTCDNNFCIPRSWVCDTDNDCKDGSD     1140
                :*  *****.:** **.** ** :     * : :  ***. ***:.*.** ..**.*.**

VgRRm*          EKNCTEQPSCFEDDFHCANGQCVDKRLRCDHDNDCEDSSDEVGCDYAKANRTKCSTGMVD     1099
LDLP,[Bos       EKSCNYTQTCSPTQFHCPDHRCIALTFVCDGTKDCADGSDEIGCVINCTAS-----QFTC     1195
                **.*.   :*   :*** : :*:   : **  :** *.***:**    :         :.

VgRRm*          CGDGQCIYTHDMCDGYIDCHNSRDERNCSA---PICHSAEFFCTGTKRCILQNWLCDGDD     1156
LDLP,[Bos       VSNGQCISKTYRCDGVFDCDDHSDETDCPTRPPGMCHQDEFQCQEDGICIPKTWECDGHE     1255
                 .:**** .   *** :**.:  ** :* :    :**. ** *     ** :.* ***.:

VgRRm*          DCGDGMDETLP----RCHPTTKVS--------------------------TTSVPACAS     1185
LDLP,[Bos       DCLQGSDEHNGCPPKTCHPSHFVCQNGNCIYRNWLCDGDNDCGDMSDEKDCPTQPFQCPS     1315
                ** :* **        ***:  *.                            *.   * *

VgRRm*          NEFKC-GSRECIAWSRVCDGRTDCADFSDEGTHCV-SYCGTTNGGCAHLCRESPTGPLCS     1243
LDLP,[Bos       WQWQCPGHSICVNLSAVCDGVSDCPGGTDESPLCNQNSCSDSNGGCTHQCIQGPYGAQCQ     1375
                 :::* *   *:  * **** :** . :**.  *  . *. :****:* * :.* *  *.

VgRRm*          CHPGYRLNTDRKSCDDIDECATPGHCSHFCQNSKGSYKCTCADGYALGADRRYCKVQYGE     1303
LDLP,[Bos       CPLGYLLGNDSKTCEDIDECRTPGFCSQYCYNMRGSFRCWCDSEYTLDADRRTCKATESE     1435
                *  ** *..* *:*:***** ***.**:* * :**::* * . *:*.**** **.  .*

VgRRm*          PFLLYMLPNQIRSFSMHGHAQHLL-AEDSLSDMHGMDYRVTDKSIFWTEMDEGVIN-VMT     1361
LDLP,[Bos       TLLLVASQSQLVAGNMTQNGYFVYPVIQHGSHIVAVDFDSVSGRIFWSDGTQGKIWSAFQ     1495
                 :**    .*: : .*  :. .:  . :  *.: .:*:  ..  ***::  :* *  .:

VgRRm*          LGNGKQFTLLEDIHKPYHIAVDWVAGNIYFTDGWVHIQAC-EPTFKHCTDVIDTAYSHLN     1420
LDLP,[Bos       NGTDKKLILDSGVSMTGSIAVDWIGRNLYWTDISLRTIDVAKLDGSHKTVLISENITNLG     1555
                 *..*:: * ..:     *****:. *:*:**  ::     :   .* * :*.   ::*.

VgRRm*          TFA--LSANDGLMFWAVWHEVVQKDHGLIERSNMDGTARVVLLTDKILWPCSITVDAVHK     1478
LDLP,[Bos       GLAVDPRATDRVMFWSDWGS-----HPRIERASMDGSQRTIIVQEKIYWPNGLALDYPNR     1610
                 :*    *.* :***: * .     *  ***:.***: *.::: :** ** .:::*  ::

VgRRm*          RIYWSDANKNVIESATYDGKDRKLVRGT--GLSSPFSIALFEDWLYWSDWGSDSLMACNR     1536
LDLP,[Bos       LLYFMDGYLDYLDFCHYDGSNRRQVIASDLILRHPYSISLFEDTVYWSDRATHEVMKANK     1670
                 :*: *.  : :: . ***.:*: * .:   *  *:**:**** :**** .:..:* .*:

VgRRm*          YTGTHVGLVHHGTAKATVLKVLHAVHQPSGVNRCARNQCAHICLLNP-NAYTCACSHGYT     1595
LDLP,[Bos       WHGGNQSVVMTLH-QPLGIVVVHPAKQPVSSNPCSYTRCSHLCLLSSKSLYSCACPSGWT     1729
                : * : .:*     :   : *:* .:** . * *: .:*:*:***.  . *:***  *:*

VgRRm*          LAEDAHKCVESDERYHINSSD--ILGQFCNPVC--LNGGRCISGNDS-------------     1638
LDLP,[Bos       LARDSVTCVRDDQAFLIVVRNSIIFGISLNPDVKTFDGMVPISGIRNGYDVAVDYSEQFI     1789
                **.*: .**..*: : *   :  *:*   **    ::*   ***  .

VgRRm*          YFCKCADGFKGPS--CTDTSVVSMLSQKSTSSSTALASILVSALCVALLVLGYVLYRRN-     1695
LDLP,[Bos       YWLENPGEIHRVKTDGTNRTVFAPLSSLGASASL------------ALDWLSRNLYFTDH     1837
                *: :  . ::  .   *: :*.: **. .:*:*             **  *.  **  :

VgRRm*          ---RDKLAALDFSVSFKKPTFKKRQGLLEDEHP-------IAADEDYHAMNTTPGFINPA     1745
LDLP,[Bos       VTRSIKVMTLQGDVSYRKTLIANDGTSLGVGLPVGITIDPINGKLYWSDRGTNSGL-PPK     1896
                     *: :*: .**::*  : :    *    *       * ..  :   .*. *:  *

VgRRm*          FN-----TRK------------TELLSEDGELKRWASSDSLQ--SSSSKEQSSCVLAGD     1785
LDLP,[Bos       IASANMDGKSPRTLFTGSLENVAFITLDIEEQKLYWAVSSTGVIERGNVDGTNRMILVNH     1956
                :       :.                 *. : :   ** *.:     .. .  .  :*...

VgRRm*          MA-----AKQDKVFFFRKH-----------------------------------------     1799
LDLP,[Bos       LSYPWGLAVHGRFLYYSDEEYEVIERVDKATGANKVVLRNNLPDLRGLKIYQRRGSESSN     2016
                ::     * :.:.::: ..

VgRRm*          ------------------------------------------------------------     1799
LDLP,[Bos       GCSNNMNACQQICLPVPGRLFSCACATGFKLNPDHQTCSPYNSFIVVSTLRTIRGFSLQL     2076


VgRRm*          ------------------------------------------------------------     1799
LDLP,[Bos       SDHSEAMVPVAGPGRNALHVDVDVSSGFIYWCDFNISVASNNAIRRIKPDGSNFTNIVTD     2136
```

```
VgRRm*         ------------------------------------------------------------     1799
LDLP,[Bos      GIGVNGVRGIAVDWVAGNLYFTNAFRSETLIEVLRINTTHRHILLKTIVDMPRDIVVDPK     2196

VgRRm*         ------------------------------------------------------------     1799
LDLP,[Bos      NRYLFWSDYGQNPKIERSFLDCTNRTVLVSDITATPRGLALDHSSNYIYWVDDAVDLIAR     2256

VgRRm*         ------------------------------------------------------------     1799
LDLP,[Bos      ISIEGGETEVIRFGSHYPAPYAITVFGNSIIWVDRNLKKILQASKEPNRADRPTVIRDNI     2316

VgRRm*         ------------------------------------------------------------     1799
LDLP,[Bos      DWLRDVTIFDQSVQPRSPAEVNNNPCLENNGGCAQFCFALPKSQTPKCDCAFGTLQADGK     2376

VgRRm*         ------------------------------------------------------------     1799
LDLP,[Bos      SCAISSENFLIFALDDSLRSLRFDPKDYSQPFPAISVERMAVALDYDSIDNRIYFTQLLP     2436

VgRRm*         ------------------------------------------------------------     1799
LDLP,[Bos      SGKGQISYINLNSRSSPPTVVVSGIGSPEGIAFDWINKRIYYSDYTNQMIKSIATDGSRH     2496

VgRRm*         ------------------------------------------------------------     1799
LDLP,[Bos      TLIAQVPKPRGIVLDPCQGYMYWTDWGTNAKIEMATMGGYSRRSLVDRGLVWPNGLTLDY     2556

VgRRm*         ------------------------------------------------------------     1799
LDLP,[Bos      EQNLIYWADANLEKIERMDLEHYLREVIVSRANSPFGLAIYGQYVYWTDLLTQKIYRANK     2616

VgRRm*         ------------------------------------------------------------     1799
LDLP,[Bos      VDGSGQTAVTVTLPFRPKGIRAVVKDQQQCLSPCDRFNGGCSHICAPGPNGAECQCPHEG     2676

VgRRm*         ------------------------------------------------------------     1799
LDLP,[Bos      RWYLANNNKYCIQDNGTRCDSSKFTCLSGKCIPDQLQCNDIDDCGDSSDELETLCAFHSC     2736

VgRRm*         ------------------------------------------------------------     1799
LDLP,[Bos      PSTSFTCANGRCVPYSDRCDHYNDCGDNSDEAGCHFRACNRTEFTCSNGRCIPSELVCDG     2796

VgRRm*         ------------------------------------------------------------     1799
LDLP,[Bos      VDNCLDNSASDEKNCPERTCHTGYVKCTNSTICIPRSFLCDGDNDCGDMSDENPLFCATR     2856

VgRRm*         ------------------------------------------------------------     1799
LDLP,[Bos      SCGSDEFHCTSGPCIPARWYCDHEKDCSDGSDEPPTCEFSQSTCASDYFKCDNNRCIPMM     2916

VgRRm*         ------------------------------------------------------------     1799
LDLP,[Bos      WVCDGDNDCGDMSDEDERHNCTSENRNCSSSEFACEVGVRPHRGCIPKSWVCDGEADCLD     2976

VgRRm*         ------------------------------------------------------------     1799
LDLP,[Bos      ALDEHQNCTRRSCFGTEFVCNNGLCIPNHFRCDRNNDCGDYSDERGCVYPTCDETLFTCQ     3036

VgRRm*         ------------------------------------------------------------     1799
LDLP,[Bos      NGLCINKAYVCDGDNDCKDNSDELEHLCHTPETTCPPHQFRCDNGNCIEMMKVCNNFPDC     3096

VgRRm*         ------------------------------------------------------------     1799
LDLP,[Bos      SDNSDEKGCGINECNDPTLSGCNQNCTDTLTSFYCSCNPGYKLLSDKRTCVDIDECEETP     3156
```

```
VgRRm*        ------------------------------------------------------------     1799
LDLP,[Bos     LILQGLGNVVALDFDRVEKRLYWLDIENKVIERMFVNTTNRETVLKYNLPGAESLAVDWV     3276

VgRRm*        ------------------------------------------------------------     1799
LDLP,[Bos     TRKLYWVDSYLNCLSVSDLNGRYRRKLAEHCVDANNTFCFENPRGLALHPRYGHVYWADW     3336

VgRRm*        ------------------------------------------------------------     1799
LDLP,[Bos     GDRAYIGRVGMDGTLKSLIISTKIMWPNGLTIDYTNDLLYWADAHLGYIEFSDLEGRHRH     3396

VgRRm*        ------------------------------------------------------------     1799
LDLP,[Bos     TVYETGTLSHPFAITIFEDTIYWTDWNTKTVEKGNKYNGSDRVALLNVTHRPYDIRVYHP     3456

VgRRm*        ------------------------------------------------------------     1799
LDLP,[Bos     YRQPIVPNPCGTNNGGCSHLCLIKEGGVGFTCECPDNFYTVQRGPNTQCLPMCSSTQFLC     3516

VgRRm*        ------------------------------------------------------------     1799
LDLP,[Bos     ADSEMCIPIWWKCDGRRDCLDGSDEPITCPQRFCALGMFQCNDGNCTNSHSLCNLRQDCP     3576

VgRRm*        ------------------------------------------------------------     1799
LDLP,[Bos     DGSDEDPVLCEHHQCEPYEWQCANKRCIPESWQCDMQDDCDDNSDEDSSHCASRTCRPGY     3636

VgRRm*        ------------------------------------------------------------     1799
LDLP,[Bos     FKCANGHCIPQIWKCDVDNDCGDYSDEPLQECLGPSYRCDNYTEFSCKTNYRCIPKWAVC     3696

VgRRm*        ------------------------------------------------------------     1799
LDLP,[Bos     NGVDDCRDNSDEQDCESMTCKPSGEFRCTNHRCIPLRWRCDGQNDCGDRSDEENCAPRKC     3756

VgRRm*        ------------------------------------------------------------     1799
LDLP,[Bos     TESEFRCDDQSCIPSRWVCDQTNDCGDNSDERDCEMMTCRPGYFQCDSGHCISEHMKCNG     3816

VgRRm*        ------------------------------------------------------------     1799
LDLP,[Bos     VADCRDASDEANCPTRFPNGAYCPATMFECKNHVCIHSSWKCDGDNDCGDGSDEELHLCL     3876

VgRRm*        ------------------------------------------------------------     1799
LDLP,[Bos     NVACDSPYRFRCDNNRCIYRHEVCNQEDDCGDGSDEKKELCVEPTPRPCTPDEFKCSNGR     3936

VgRRm*        ------------------------------------------------------------     1799
LDLP,[Bos     CIPQHRVCDHVNDCGDNFDETGCNTGKDRSCAENLCEHNCTQLREGGFICSCRPGFKPNS     3996

VgRRm*        ------------------------------------------------------------     1799
LDLP,[Bos     IDRNLCEDINECMQFGSCPQICHNTKGSYECSCAEGFTSLSDRYGERCAADGSPPLLLLP     4056

VgRRm*        ------------------------------------------------------------     1799
LDLP,[Bos     ENVRIRKYNLSSLQFSEYLEDQERIKAMDYDWDPEGTGLSVVYYTVLGEGSNSGAIKRAY     4116

VgRRm*        ------------------------------------------------------------     1799
LDLP,[Bos     IPNFESGSNNPVMEINLDLKYIVQPDGLAVDWVGRHIYWSDARRQRIEVAELDGRYRKWL     4176

VgRRm*        ------------------------------------------------------------     1799
LDLP,[Bos     ISTELGQPAAIVVNPKLGFMYWTDWGENPKIESAWMDGQRRKVLVQEDLGWPTGLCIDYM     4236

VgRRm*        ------------------------------------------------------------     1799
LDLP,[Bos     NGDRIYWSDLKDNIVETIKYDGTDRRIVVTSAVNPYSLDIFESQLYWTSKDKGEVWIQDK     4296

VgRRm*        ------------------------------------------------------------     1799
LDLP,[Bos     FGRNKKEKLLTVNPWLTQVRVFHQRKYNHSVPNRCKDVCSHLCLLRPKGYTCACPQGSRF     4356

VgRRm*        ------------------------------------------------------------     1799
LDLP,[Bos     LEGSVTVCDAAIVGAVSMPPPCRCMNRGSCYFDENNLPKCKCSSGYVGEYCEMGLSQGVP     4416

VgRRm*        ------------------------------------------------------------     1799
LDLP,[Bos     PGTTASVLLTVILIVIIAALATLGFLHYRKTGSILPSLPKLSSLSHLKSSENGNGVTFRS     4476

VgRRm*        ------------------------------------------------------------     1799
LDLP,[Bos     GDDVNMDIGVTGFGPESAIDRSLAMNEHFAADFGKSPIIFENPTYSSKDTAITVAQPTTA     4536

VgRRm*        ------------------------------------------------------------     1799
LDLP,[Bos     PVTESATVYNKNYGSPINPAELVTDTKPTSSSDETQPTKWNIFKRKPKQNTNFENPFYSE     4596

VgRRm*        -----------------------------------------------------     1799
LDLP,[Bos     MENEPKVGAAVTPPPSPSPPAKVSWKKGSTPGYSATEDTFKDTANLVREDSEA     4649
```

VgR against other tick species

```
CLUSTAL O(1.2.4) multiple sequence alignment


VgRRa,Bm86-like,ACR19242.1      ------------------------------------------------------------    0
VgRHd,Bm86-like,ADY76577.1      ------------------------------------------------------------    0
VgRIs,XP_029830196.2            MTTAAYSVLLATAFYLVSSASAECPQGWFDCENGRCIAMFWKCDGQNDCGNHNDEKNCRD    60
VgRHl,BAG14342.1                MKSVACVVLAAVALYLVGDAACECQRGWFDCGNDRCISMFWRWDGQNDCGSHKDETGCGD    60
VgRDa,XP_050052543.1            MKSVACVVLAAVALYLVGDAACECQQGWFDCGNDRCITMFWRCDGQNDCGNHKDETGCSD    60
VgRRm*                          MKFTACAVLAVVALYLVGNVFSECPQAWFDCGNGRCIAMFWRCDGQNDCGNHKDETGCSA    60
VgRRs,XP_037521270.1            MKATACAVLAIAALYLVGDVSSECPQAWFDCGNGRCIAMFWRCDGQNDCGNHKDETGCSA    60


VgRRa,Bm86-like,ACR19242.1      ------------------------------------------------------------    0
VgRHd,Bm86-like,ADY76577.1      ------------------------------------------------------------    0
VgRIs,XP_029830196.2            EPEECPVEKFNCHDRSYCIPKVWLCDGEADCGDSSDEVGCT-VNCTGFSCKSNECIPLNW    119
VgRHl,BAG14342.1                HAHRCRSDKYACRDGSYCVAEIWVCDGEADCHDSCDELDCHSSNCTGYRCHNIECIPNHW    120
VgRDa,XP_050052543.1            HAHRCPSDKFACRDGSYCVPEIWVCDGEADCHDSSDELDCHSNNCTGYRCHNNECIPNHW    120
VgRRm*                          THSRCPADKFACRDSSYCVPQIWVCDGEADCHDSSDEQDCHSSNCTGFRCHNNECIPAHW    120
VgRRs,XP_037521270.1            TQSRCPADKFACHDSSYCVPQIWVCDGEADCHDSSDEQDCHSSNCTGYRCHNNECIPAPW    120


VgRRa,Bm86-like,ACR19242.1      ------------------------------------------------------------    0
VgRHd,Bm86-like,ADY76577.1      ------------------------------------------------------------    0
VgRIs,XP_029830196.2            RCDESEDCLDGTDELGCANATAAKLVQQSPKCDVDSGHFPCLDGKCLLPEKVCDGTKDCG    179
VgRHl,BAG14342.1                HCVVTEDCADASDVLNCHAATYSSTTTVAPRCRVDQGRYPCLDGQCLLRSKVCDGRKDC-    179
VgRDa,XP_050052543.1            HCDETEDCADASDELNCHAATNSSPTTVAPRCDVDQGRFPCLDGQCLLPSKVCDGRKDCG    180
VgRRm*                          RCDQTEDCADASDELDCGGVQNSSTTTPTPRCDVDQGRFPCLDGQCLLPSKVCDGRKDCS    180
VgRRs,XP_037521270.1            RCDQTEDCIDASDELDCGGAKNSSATTLAPRCDVDQGRFPCLDGQCLLPSKVCDGRKDCS    180


VgRRa,Bm86-like,ACR19242.1      ------------------------------------------------------------    0
VgRHd,Bm86-like,ADY76577.1      ------------------------------------------------------------    0
VgRIs,XP_029830196.2            DGADEGSFCKVNECSSKKCSQGCFVSANGSSCYCNTGFRLMDDGISCIDNDECTERPHVC    239
VgRHl,BAG14342.1                -------------------SEGCFVATNGSTCYCNTGFGLMADHISCADVDECAVEPYVC    220
VgRDa,XP_050052543.1            DGADEGAFCKVNECSQKKCSQGCFVATNGSTCYCNPGFRLMADHISCADVDECAEEPYVC    240
VgRRm*                          DGADEGSFCKVNECSQKKCSQGCFVATNGSTCYCNAGFRLLADHISCADVDECAEDPHVC    240
VgRRs,XP_037521270.1            DGADEGAFCKVNECSQKKCSQGCFVATNGSTCYCNAGFRLMADHISCADIDECVEDPHVC    240


VgRRa,Bm86-like,ACR19242.1      ------------------------------------------------------------    0
VgRHd,Bm86-like,ADY76577.1      ------------------------------------------------------------    0
VgRIs,XP_029830196.2            SQMCTNTPGSYHCSCLNGYLLTDSTFCKAKDPEPLMIFSDSKQVRGLWLRTNRYFEIHSA    299
VgRHl,BAG14342.1                SHGCTNSPGSYSCHCLEGYQLADKCFCKARDPEPRLLYSNTKEFRGLWLRSNRYFDIHPA    280
VgRDa,XP_050052543.1            SHGCTNSPGSYSCHCLEGYQLADKSFCKARDPEPLLLYSNTKEIRGLWLRSNRYFEIHPA    300
VgRRm*                          SHGCINSPGSYSCHCLEGYQLTDNSFCKARDPEPLLVFSTTKEIRGLWLRSNRYFEIHPA    300
VgRRs,XP_037521270.1            SHGCINSPGSYSCHCLEGYQLADKSFCKARDPEPLLVFSTTKEIRGLWLRSNRYFEIHPA    300


VgRRa,Bm86-like,ACR19242.1      ------------------------------------------------------------    0
VgRHd,Bm86-like,ADY76577.1      ------------------------------------------------------------    0
VgRIs,XP_029830196.2            SGQAVGVDFDNSARRVFWTDVTSKKSAIHSCLLDGTGFKVLISEDYSLMEDVALDWLANN    359
VgRHl,BAG14342.1                EAQAVGVEFDSEQRRVYWTDVPTLKSSVYSGRLDGSGLKSLFSAELTLLEDLSLDWVTNN    340
VgRDa,XP_050052543.1            EAQAVGVEFDSDQRRVYWTDVSTQESSVYSCRLDGSGLKTLFSAEKTLLEDLSLDWVTNT    360
VgRRm*                          EAQAVGVEFDSDQHRVFWTDVSTRRSSIHSCRLDGSDFKTLFSAEKTLLEDLSLDWVANN    360
VgRRs,XP_037521270.1            EAQAVGVEFDSDQHRVFWTDVSTWKSSIHSCRLDGSDFKTLFSAEKTLLEDLSLDWVTNN    360


VgRRa,Bm86-like,ACR19242.1      ------------------------------------------------------------    0
VgRHd,Bm86-like,ADY76577.1      ------------------------------------------------------------    0
VgRIs,XP_029830196.2            LYITDSALKRILVCKSDGSACATVVTAFLDSPRAIILNPSKRLMYWTDWGKSPAVMAAGM    419
VgRHl,BAG14342.1                LYITDSLVKRIVVCTTDGLSCSAVITDSIDSRRAIIVNPARKTMYWTDWSAQPAVMHSIM    400
VgRDa,XP_050052543.1            LYITDSLVKRILVCTTDGLSCSALITDSIDSPRAIIVNPAQKTMYWTDWGAQPAVMHSNM    420
VgRRm*                          LYITDSLVKRILVCTTDGASCSALITDSVDSPRAIIVNPPQKVVYWTDWGSRPAIMHASM    420
VgRRs,XP_037521270.1            LYITDSLVKRILVCTTDGVSCSALITDSVDSPRAIIVNPPQKAVYWTDWGPRPAIMHSST    420


VgRRa,Bm86-like,ACR19242.1      ------------------------------------------------------------    0
VgRHd,Bm86-like,ADY76577.1      ------------------------------------------------------------    0
VgRIs,XP_029830196.2            DGSNVRALVSENLGWPNGLAYDHSTDRLYWCDAELAKIEYMDLLTMKRNVVIQDAVFHPF    479
VgRHl,BAG14342.1                DGTNVGQLVSTDRGWPNGLTLDHTTNRLYWCDAKLSRLQYLELSTLRRAVVMAEDVFHRF    460
VgRDa,XP_050052543.1            DGTNVAQLVSTDLGWPNGLTLDHTTNRLYWCDAKLSRLEYLELATLRRAVVMDEDVFHPF    480
VgRRm*                          DGTNIQQLVSTDLGWPNGLTLDHTTNRLYWCDAKLSSLEYLELSTLKRDVVMHEEVFHPF    480
VgRRs,XP_037521270.1            DGTNIQQLVSTDLGWPNGLTLDHTTNRLYWCDAKLSRLEYLELATLKRDVVMDEDVFHPF    480


VgRRa,Bm86-like,ACR19242.1      ------------------------------------------------------------    0
VgRHd,Bm86-like,ADY76577.1      ------------------------------------------------------------    0
VgRIs,XP_029830196.2            SLTVFEDTLYWSDWLAYSLDSSNKFDGKHHHRILREDSKHIMGVHIYHPLLRSKDLVNPC    539
VgRHl,BAG14342.1                ALAIFEDTVYWSDWASYSLDSSNKRTGKHHHRVLREIGNHIMGVHVYHPVLRFQGIHYPC    520
VgRDa,XP_050052543.1            ALAIFEDTVYWSDWASYSLDSSNKRTGKHHHRVLRENGNHIMGVHVYHPVLRLRGIHNPC    540
VgRRm*                          ALAVFEDTVYWSDWASYSLDSSNKRTGKQHHRVLRENGHHIMGVHVYHPVLRQRGIQNPC    540
VgRRs,XP_037521270.1            ALTVFEDTVYWSDWASYSLDSSNKRTGKQHHRILRENSHHIMGVHVYHPVLRQRGIQNQC    540


VgRRa,Bm86-like,ACR19242.1      ------------------------------------------------------------    0
VgRHd,Bm86-like,ADY76577.1      ------------------------------------------------------------    0
VgRIs,XP_029830196.2            WDNPCEDICVLAADGYECLCRLGYEPSPKDKHTCTVTRLSSYAVVSEENVLYRVRHESIG    599
VgRHl,BAG14342.1                WAIRCHHICVLSGNSYTCLCRIRYKLL-ADKRSCTVTKDFGFVVVAEEDLLYNFDIDRVG    579
VgRDa,XP_050052543.1            WANPCDHICVLSGDSYTCLCRIGYKLS-ADKRSCTVTKDFRFVVVAEEDLLYKIDLDRVG    599
VgRRm*                          WDNPCDHICVLSADSYMCLCRIGYKLS-ANKHSCAVTKDFSFVIVAEEDLVYKIDLNKVG    599
VgRRs,XP_037521270.1            WDNPCDHICVLSGDSYMCLCRIGYKLA-ANKHSCTVTKDFSFVIVAEEDLLYKIDLNKVG    599
```

```
VgRRa,Bm86-like,ACR19242.1    ------------------------------------------------------------    0
VgRHd,Bm86-like,ADY76577.1    ------------------------------------------------------------    0
VgRIs,XP_029830196.2          GESVSKMPVNRLIMVGAMTLDWATQTLFLSDNPREMILAVDMNTYNVSTIREHHVGSIFG    659
VgRHl,BAG14342.1              PPLRVALPFSNLGMISALAFDWSNQTLHYSDNRHEMLSAINVNTFDQWTVH-DHNGSVFG    638
VgRDa,XP_050052543.1          PPLPVALPFSNLGMISALAFDWSNQTLHYSDNSHEILSAINVNTFNQWTVH-DHIGSVFG    658
VgRRm*                        APVPVTLPVHNLGIISALTFDWANQTLHYSDNRHSVLSAINVKSFEQWTVH-DHIGSVFG    658
VgRRs,XP_037521270.1          APVPKTLPVTNLGMISALAFDWSNQTLHYSDNRHSVLSAINVNTFEQWTVH-DHIGSVFG    658


VgRRa,Bm86-like,ACR19242.1    ------------------------------------------------------------    0
VgRHd,Bm86-like,ADY76577.1    ------------------------------------------------------------    0
VgRIs,XP_029830196.2          MDFDGQNRNLYWVDADKKTIEVCRSNGSGHIILRSDLRRPVDLALHPKAGVMYVLSAGDT    719
VgRHl,BAG14342.1              MDFDDAHQLLYWVDADSYTLEVCRAIGSGHAIIRDDLRRPVPLAPYPFAGILFVLCAGDA    698
VgRDa,XP_050052543.1          MDFDVAHQLLYWVDADKYTLEVCRANGSGHAIIRDDLRRPVGVALYPFAGILFVLSAGDA    718
VgRRm*                        IDFDVTHQLLYWVDADKYTLEACHANGSGHVIIRDDLHRPVGVALYPFAGVLFVLSAGDK    718
VgRRs,XP_037521270.1          MDFDVTHQLLYWVDADKYTLEACHANGSGHTIIRDDLHRPVGVALYPFAGILFVLSAGDK    718


VgRRa,Bm86-like,ACR19242.1    ------------------------------------------------------------    0
VgRHd,Bm86-like,ADY76577.1    ------------------------------------------------------------    0
VgRIs,XP_029830196.2          PSIVRYTMDGLSPIRVQLTSMLLGLSLSTDLVAERLFWGDSVRATIEFIDLKNG-SSTPF    778
VgRHl,BAG14342.1              PTITSYTMDGQSPRILRLATLLLPVSLSVDLVAPKLVWADATRGTIESLDLRNLFSATPF    758
VgRDa,XP_050052543.1          PTITSYTMDGQSPRILPLATLLLPVSLSVDLVARKLVWADATRGTIESLDLRNLFSATPF    778
VgRRm*                        PTITSYTMDGQNPRVLPLSTLLLPVSISVDLVARTLVWADAVRGTIESLDLQKVFTGTPF    778
VgRRs,XP_037521270.1          PTITSYTMDGQNPRVLPLSTLLLPVSLSVDLVARTLVWADAVRGTIESLDLQRVSTGTPF    778


VgRRa,Bm86-like,ACR19242.1    ------------------------------------------------------------    0
VgRHd,Bm86-like,ADY76577.1    ------------------------------------------------------------    0
VgRIs,XP_029830196.2          IVEQIHGHITSVSASHGLVHWTTREDGALYYVDLHVKPTSVHSVSLP-PRNGTVARKVIY    837
VgRHl,BAG14342.1              IVQQEKAHVSSVSAAHNDFHWISRDHASLEYIDVSVERNLRRHVPLPSKRKGTYSRRRII    818
VgRDa,XP_050052543.1          IVQQVKAHVSSVSAAHNEIHWISRDHSSLEYIDLSVEPNLRRHVPLPSKRNGTYSRRVII    838
VgRRm*                        IVQQVKAHISSVCAAHNEIHWTSRDKASLEYIDRSSEPSVHRHVSLRSTRNGTFSRRVIV    838
VgRRs,XP_037521270.1          IVQQVKAHISSVSAAHNEIHWASRDKASLEYIDLSSEPSVRRHVSLRSTRNGTFSRRVIV    838


VgRRa,Bm86-like,ACR19242.1    ------------------------------------------------------------    0
VgRHd,Bm86-like,ADY76577.1    ------------------------------------------------------------    0
VgRIs,XP_029830196.2          AAQIPDYAPGPCSVKNGGCSHVCLPAL-TSRSCLCPPRMVLSANNVTCKDENGTCSAHEL    896
VgRHl,BAG14342.1              ASQVAPFAPGRCGQNNGGCSHTCLPVRTTDRSCFCPPGMALNADATTCRVDNGTCRPHEL    878
VgRDa,XP_050052543.1          ASQVPPFAPGPCGQNNGGCSHTCLPVRTADRSCFCPPGMALNTDTTTCRVENGTCRPHEL    898
VgRRm*                        AAQVPPFAPGPCGLNNGGCSHTCLPVRTTDRSCFCPPGMALNADNRTCRVETSTCRPHEL    898
VgRRs,XP_037521270.1          AAQVPPFAPGPCGQSNGGCSHTCLPVRTTDRSCFCPPGMALNADNITCRVENGTCRPHEL    898


VgRRa,Bm86-like,ACR19242.1    ------------------------------------------------------------    0
VgRHd,Bm86-like,ADY76577.1    ------------------------------------------------------------    0
VgRIs,XP_029830196.2          SCAGTCIPATYWCDGYQDCSDNSDENNCGTVTCPANGFRCGNGKCVENAWLCDGYNDCGD    956
VgRHl,BAG14342.1              PCAGVCIAAIYWCDGRQDCPDNADEKACDAATCPSDDFSCANGHCIGKAYHCDGYDDCGD    938
VgRDa,XP_050052543.1          PCAGRCIAATYWCDGHKDCSDNADEASCGPAKCPSTDFTCSNGRCIEKEWHCDGYNDCGD    958
VgRRm*                        PCAGSCIAAVNWCDGHKDCSDNADEESCGSATCPATDFSCSNGRCIEKEWQCDGYNDCGD    958
VgRRs,XP_037521270.1          PCAGRCIAATNWCDGHKDCSDNADEESCGSTACPSTDFTCSNGRCIEKEWQCDGYNDCGD    958


VgRRa,Bm86-like,ACR19242.1    ---------------------------------------------ESSICSDFGNEFCRN    15
VgRHd,Bm86-like,ADY76577.1    ----------------------------------------------SSICSDFGKQFCQS    14
VgRIs,XP_029830196.2          HSDEINCTRRNCTSEEYQCRANYCIPVYWRCDGEKDCPDNDDEGNCSLIECPSGYDRCRD    1016
VgRHl,BAG14342.1              HSDEKNCTRQTCTPNQFTCTSGGCTPFLWRCDGEKDCPAGEDELNCGNIRCPNGHDRCAN    998
VgRDa,XP_050052543.1          LSDEKNCTRQTCATHQYTCRSGVCVPLYWRCDGSEDCPDGDDELNCSGVRCPSGHDRCAN    1018
VgRRm*                        SSDERNCTVTTCASHQYTCRGGVCLPLYWRCDGSEDCPDGDDELNCSSVRCPSAHSRCDN    1018
VgRRs,XP_037521270.1          SSDERNCTRQTCASHQYTCRSGVCVPLYWRCDGSEDCPDGDDELNCRSVRCPSGHNRCAN    1018
                                                                              : .  . . * .

VgRRa,Bm86-like,ACR19242.1    AECEV-----------------------VPGAEDDFVCK----------CPRDN------    36
VgRHd,Bm86-like,ADY76577.1    AECEV-----------------------IPGTGEDFVCK----------CPNDD------    35
VgRIs,XP_029830196.2          GQCFSRDWRCDGQADCKDSSDELDCGATTGCLPDDFRCTNKECIDPRFRCDRRVDCEDGS    1076
VgRHl,BAG14342.1              GQCIPHDYWCDGHADCTDSSDERNCTEPSDCFPDDFHCKNGQCLDKRLRCDHDEDCEDST    1058
VgRDa,XP_050052543.1          GQCIPHDWTCDGHADCADSSDEKNCTEALTCLVDDFRCTNGQCLDKRLRCDRDNDCEDSS    1078
VgRRm*                        GQCIPQDWTCDGHSDCSDSSDEKNCTEQPSCFEDDFHCANGQCVDKRLRCDHDNDCEDSS    1078
VgRRs,XP_037521270.1          GQCIPQDWTCDGHADCTDSSDEKNCTESPSCFEDDFHCANGQCVDKRLRCDHDDDCEDSS    1078
                              .:*                              :** *           * .
```

```
VgRRa,Bm86-like,ACR19242.1      -------MYFNAAEKQCEYKDTCKTRECSYGRCVESN----------PSKA-----SCVC    74
VgRHd,Bm86-like,ADY76577.1      -------MYYNAAEKQCEYKRTCKTVECSYGYCYEIR----------PGRT-----GCGC    73
VgRIs,XP_029830196.2            DENGCDSSYTANTTSQCP----RGMVNCGDGQCIYEHDICDGYVDCHLGQDERNCTSAMC    1132
VgRHl,BAG14342.1                DELGCD--YGKSNKSKCV----HGMIDCGDGHCIYAHDLCDGFADCHSGRDESNCTSAIC    1112
VgRDa,XP_050052543.1            DEVGCD--YAKVNRSQCS----TGMVDCGDGHCIYAHDMCDGYVDCHNGRDERNCSAPIC    1132
VgRRm*                          DEVGCD--YAKANRTKCS----TGMVDCGDGQCIYTHDMCDGYIDCHNSRDERNCSAPIC    1132
VgRRs,XP_037521270.1            DEVGCD--FAKVNRTKCS----TGMVDCGDGQCIYTHDMCDGYVDCHTGRDERNCSAPIC    1132
                                        :     .:*         :*. * *   .           .:      .  *

VgRRa,Bm86-like,ACR19242.1      E---ASDDLTLQCKIKNEYATDCRNRGGTAKLRTDGVIGATCDCGEWGAMSKTT-RNCVP    130
VgRHd,Bm86-like,ADY76577.1      Q---GVDTLTLKCGIQEWFANDCGRKGGTAVLRTDGFLGARCDCGEWGKMDKAQDGKCVP    130
VgRIs,XP_029830196.2            SSKEVFCINTKRCILEAWLCDGEDD------------------CGD--NMDET-LPRCHP    1171
VgRHl,BAG14342.1                HSAQFFCPYTKRCILQSWLCDGDDD------------------CGD--NMDEL-LPICHP    1151
VgRDa,XP_050052543.1            QSAEFFCTGTKRCILQSWLCDGDDD------------------CGD--GMDEL-LPRCHP    1171
VgRRm*                          HSAEFFCTGTKRCILQNWLCDGDDD------------------CGD--GMDET-LPRCHP    1171
VgRRs,XP_037521270.1            RSAEFFCTGTKRCILQSWFCDGDDD------------------CGD--GMDEH-LERCHP    1171
                                         * :* ::   . .                     **:   *.:     * *

VgRRa,Bm86-like,ACR19242.1      TTCLRPDLTCKDLCEKNLLQRDSRCCQGWNSP-----KCSAAPPADSYCSPGSPKGPDGQ    185
VgRHd,Bm86-like,ADY76577.1      TTCIRPDLTCKDLCEKNLLGKDTRCCEGWSST-----DCSVVPHEDTYCSPGSIKGEDGK    185
VgRIs,XP_029830196.2            TTTPS-TTTTEAACWSDEFRCGSHECVPWSRVCDRHLDCMDASDEGSHCDTHC-GKDNGG    1229
VgRHl,BAG14342.1                TTPSP-IATTDSACWSDEFRCGSKECIPWSRVCDMHLDCADYSDESSHCETHC-GTANGG    1209
VgRDa,XP_050052543.1            TTQVA--ATTVAACWGNEFQCGSHECIAWTRVCDGRTDCADFSDEGSHCVKHC-GTANGG    1228
VgRRm*                          TTKVS--TTSVPACASNEFKCGSRECIAWSRVCDGRTDCADFSDEGTHCVSYC-GTTNGG    1228
VgRRs,XP_037521270.1            TTKVS--ATPTPACGSNEFKCGSRECISWSRVCDGRTDCADFSDEGSHCVNYC-GTTNGG    1228
                                **      *    *  : :  .:: *  *.       .*      .::*   .    :*

VgRRa,Bm86-like,ACR19242.1      CKDACRTKEAGF---------------------------VCKHGCRS---TDKAYECTCP    215
VgRHd,Bm86-like,ADY76577.1      CKDACTTKEALL---------------------------LCKEGCIKGQKPGKAYECRCR    218
VgRIs,XP_029830196.2            CAHICRESPTGPKCSCHPGYYLTGDFKTCEDLDECQKPGHCSHFC---TNTKGSFKCTCA    1286
VgRHl,BAG14342.1                CAHICRESPLGPQCSCHPGYRLNADSKACDDVDECGTPGHCSHFC---QNSKGSYKCTCA    1266
VgRDa,XP_050052543.1            CAHICRESPIGPICSCQPGYRLNSDHKSCDDVDECATPGHCSHFC---QNSKGSFKCTCA    1285
VgRRm*                          CAHLCRESPTGPLCSCHPGYRLNTDRKSCDDIDECATPGHCSHFC---QNSKGSYKCTCA    1285
VgRRs,XP_037521270.1            CAHLCRESPAGPLCSCHPGYRLNTDKKSCDDIDECATPAHCSHFC---QNSKGSYKCTCA    1285
                                * . *  .                                *.. *        :::* *

VgRRa,Bm86-like,ACR19242.1      RGFTVAEDGITCKSIPYTGGCTAEQKQTCRPTESCRVHTGKVLCECPWNQHLVGDKCIGD    275
VgRHd,Bm86-like,ADY76577.1      QGYEIAEDGITCKRVPGIVDCTEEQKAACLPGQQCRVHKGNSVCECPSDQQLLDGKCASE    278
VgRIs,XP_029830196.2            EGYAIAADRQYCKAQSGEASLL-----YMLPNQIR------SFSMRGHAQHLLAQDDLAD    1335
VgRHl,BAG14342.1                DGYSLAADHRSCKVQHGEAFLL-----YMLPNQIR------SFSMRGHAQHILARDDFSD    1315
VgRDa,XP_050052543.1            DGYALGVDRRYCKVQYGEAFLL-----YMLPNQIR------SFSMHGHAQHLLAEDSLSD    1334
VgRRm*                          DGYALGADRRYCKVQYGEPFLL-----YMLPNQIR------SFSMHGHAQHLLAEDSLSD    1334
VgRRs,XP_037521270.1            DGYALGADRRYCKVQYGEPFLL-----YMLPNQIR------SFSMHGHAQHLLAEDSLSD    1334
                                 *: :. *   **                 * :         ..     *:::  .  .:

VgRRa,Bm86-like,ACR19242.1      CVDNKCHEEFTDCGVYMNRQSCYCPWKSRKPGPN----------VNIN-----------E    314
VgRHd,Bm86-like,ADY76577.1      CVEGRCHENFTDCGVYMNKQGCYCPWTTRKPPRG----------VEIS-----------R    317
VgRIs,XP_029830196.2            MHGLD--YHFDDKAIY---------WTEMKEGTINVMLLAKKTPQTVLEDVHRPFHVAVD    1384
VgRHl,BAG14342.1                MYGMD--YRVADKSIY---------WTEMDEGTINVMTLGNGKHFTLLEQIYKPFHIAVD    1364
VgRDa,XP_050052543.1            MHGMD--YRVADKSIF---------WTEMDEGIINVMTVGNGKQFTLLEDIHKPHHIAVD    1383
VgRRm*                          MHGMD--YRVTDKSIF---------WTEMDEGVINVMTLGNGKQFTLLEDIHKPYHIAVD    1383
VgRRs,XP_037521270.1            MHGMD--YRVTDKSIF---------WTEMDEGVINVMTLGNGKQFTLLEEIHKPYHIAVD    1383
                                        .. * .::         *.  .                :

VgRRa,Bm86-like,ACR19242.1      CLLNEYYYTVSFTPNISLDSDHCDWYEDRVLEAIRTSIGKEVFKVEILNCTQDIKARLIA    374
VgRHd,Bm86-like,ADY76577.1      CVLNEYYYTVSFTPNISLNSDHCEWYEARVLEAIRTAIGEEVFKVEILNCTQDIKARLIA    377
VgRIs,XP_029830196.2            WIAGNIYFTDGWV--------------------------------HIQACDYSFKHCTDV    1412
VgRHl,BAG14342.1                WVANNVYFTDGWV--------------------------------HIQACEPTFKHCADV    1392
VgRDa,XP_050052543.1            WVAGNIYFTDGWV--------------------------------HIQACEPTFQHCTDV    1411
VgRRm*                          WVAGNIYFTDGWV--------------------------------HIQACEPTFKHCTDV    1411
VgRRs,XP_037521270.1            WVAGNIYFTDGWV--------------------------------HIQACEPTFKHCTDV    1411
                                 : .: *:* .:.                                .*  *   ::    .

VgRRa,Bm86-like,ACR19242.1      EKPLSNHVLRKLQTCEHPIGEWCMMYPKLLIKKNSATEIEEENLCD---SLLKN------    425
VgRHd,Bm86-like,ADY76577.1      SKPLSKHVLKKLQACEHPVAEFCMLYPKLPIKKGSVTGIEEENLCE---SLLKN------    428
VgRIs,XP_029830196.2            LETTYSHVNSFTLAANHGIMFWCVWSDVLGKSDG---IIERSNMDGSGRVILVSDKILWP    1469
VgRHl,BAG14342.1                VDTTYPHVNSFTLAANDGLMFWAVWMDVVRQPHG---LIERANMDGTARTILLTDKILWP    1449
VgRDa,XP_050052543.1            VDTTYSHLNTFALSANDGLMFWAVWHEVVNKNHG---LIERSNMDGTTRVVLLTDKILWP    1468
VgRRm*                          IDTAYSHLNTFALSANDGLMFWAVWHEVVQKDHG---LIERSNMDGTARVVLLTDKILWP    1468
VgRRs,XP_037521270.1            LDTAYSHLNTFALSANDGLMFWAVWHEVVNKDYG---LIERSNMDGTARVALLTDKILWP    1468
                                 .    *:     :.:. :  :.:    :    .    **. *:       * .

VgRRa,Bm86-like,ACR19242.1      ----------------------QEAAYKGQNKC--------------VKVDNLFWFQCAD    449
VgRHd,Bm86-like,ADY76577.1      ----------------------QEKAYKGVNKC--------------AKVGDLYWFQCAD    452
VgRIs,XP_029830196.2            CSLTVDHVHKLLYWSDSNKNVIESATFDGKDRKTIVMGAISSPFSIALYEDWLYWADWGS    1529
VgRHl,BAG14342.1                CSVTVDAVHKLIYWADANKNILESATYDGKNRNTVLGVGISSPFSIALFQDWLYWSDWGS    1509
VgRDa,XP_050052543.1            CSITVDAVHKRIYWSDANKNVIESATYDGKQRKLVRGAGLSSPFSIALFEDWLYWSDWGS    1528
VgRRm*                          CSITVDAVHKRIYWSDANKNVIESATYDGKDRKLVRGTGLSSPFSIALFEDWLYWSDWGS    1528
VgRRs,XP_037521270.1            CSITIDAVHKRIYWSDANKNVIESATYDGKDRKVVRGAGLSSPFSIALFEDWLYWSDWGS    1528
                                                      :. ::.* ::                  . *:* : ..
```

```
VgRRa,Bm86-like,ACR19242.1      QCPPDTKPGEIGCIERTTCNPKEIQECQDKKLECVYKNHKAECKCPD-----DRECSREP    552
VgRHd,Bm86-like,ADY76577.1      QCPPGTVFGQAGCAARTTCNPKEIRECEDEKKECVYRDQKAECKCPKGTVDDGQRCSGEP    560
VgRIs,XP_029830196.2            ACAHGYKLMEDS------------HSCFDMENETAYSLQSS-------------DVLKQP    1624
VgRHl,BAG14342.1                ACAHGFTLAKDS------------HTCVESD-DRSFNLSSS-------------DVLAQP    1603
VgRDa,XP_050052543.1            ACSHGYSLAHDA------------HTCVESD-DH-YNLTSS-------------DVLGQF    1621
VgRRm*                          ACSHGYTLAEDA------------HKCVESD-ER-YHINSS-------------DILGQF    1621
VgRRs,XP_037521270.1            ACSHGYTLAEDS------------HSCVESD-EH-YHLNSS-------------DILGQF    1621
                                 *  .    . .            : * : . :  :   .:                 :

VgRRa,Bm86-like,ACR19242.1      AKDSCSEEDNGKCQSSGQRCVMENGKAVCKEKSEATTAATATTKAKNKDPDPGKSSA---    609
VgRHd,Bm86-like,ADY76577.1      AEASCTEENIAACRSKGQRCAIENGRPVCKETSGVTTAEATTTQATKADPDPGKSGGVAV    620
VgRIs,XP_029830196.2            C---------DPECVNGGICVHNKNKLYCQCPKEFEGLSCEDPVVLALPVADQASNSMWL    1675
VgRHl,BAG14342.1                C---------NPVCLNGGRCLSENSSYFCKCSDDFQGPSCELPVVVAMPHVQSSSRSTAL    1654
VgRDa,XP_050052543.1            C---------NPVCLNGGRCISANGSYFCKCLNGFNGLSCEDTMVISPLPQQSGSHSTAL    1672
VgRRm*                          C---------NPVCLNGGRCISGNDSYFCKCADGFKGPSCTDTSVVSMLSQKSTSSSTAL    1672
VgRRs,XP_037521270.1            C---------NPVCLNGGRCISANDSYFCTCAGGFQGPSCADAVVVSMLPQLSNSSSTAL    1672
                                .              .*  *   :.   *               .         * .

VgRRa,Bm86-like,ACR19242.1      ------------------------------------------------------------    609
VgRHd,Bm86-like,ADY76577.1      SAT--TLL----------------------------------------------------    626
VgRIs,XP_029830196.2            ASVLIFIVCVALLAFGYLFYRRNRHKLSAIDFTVGFKTPTFAAKDEGLLENEHPIAEDYH    1735
VgRHl,BAG14342.1                AAILLTVLCVALLVLGYILYRRHKNKLAALDFSVSFKKPAFG-KREGLLDNEHPVAAADE    1713
VgRDa,XP_050052543.1            ASILVSILCVALLVLGYVLYRRNRQKLAALDFSISFKKPTFR-KRQGLLEDEHPVAADED    1731
VgRRm*                          ASILVSALCVALLVLGYVLYRRNRDKLAALDFSVSFKKPTFK-KRQGLLEDEHPIAADED    1731
VgRRs,XP_037521270.1            ASILISVLCVALLVLGYVLYRRNRDKLAALDFSVSFKKPNFR-KRQGLLEDEHPIAADED    1731


VgRRa,Bm86-like,ACR19242.1      ------------------------------------------------------------    609
VgRHd,Bm86-like,ADY76577.1      ------------------------------------------------------------    626
VgRIs,XP_029830196.2            V-VAASHPGFKNPLFLDGHKSHLLTENGEFQRWSSNESVVSSVGVDDSQATLAATEDETK    1794
VgRHl,BAG14342.1                CATASPDSGFMNPAF-GGRKSQLLTDDGEFKRWSSDESIQSSSVHSSVRTDPG--PSTSA    1770
VgRDa,XP_050052543.1            YHAMTPAPGFVNPAF-SNRKSQLLADDGQFKRWSSSESLQSSSSKDKSTCSLA--TDSAA    1788
VgRRm*                          YHAMNTTPGFINPAF-NTRKTELLSEDGELKRWASSDSLQSSSSKEQSSCVLA--GDMAA    1788
VgRRs,XP_037521270.1            YHAMNTAPGFINPAF-DTRKGQLLSENGEFKRWASSDSLQSSSSKEQSSCVLA--GDSAA    1788


VgRRa,Bm86-like,ACR19242.1      -----------     609
VgRHd,Bm86-like,ADY76577.1      -----------     626
VgRIs,XP_029830196.2            QKKQVFFFRKV     1805
VgRHl,BAG14342.1                KHDQVFFFRKV     1781
VgRDa,XP_050052543.1            KN-EVFFFRKH     1798
VgRRm*                          KQDKVFFFRKH     1799
VgRRs,XP_037521270.1            KQDQVFFFRKH     1799
```
