# Supplementary material for: Comparative immunoinformatic analysis of Rhipicephalus microplus cocktail vaccine targets
Source: Parasit Vectors. 2025 Dec 9;18:502. doi: 10.1186/s13071-025-07109-y (PMC12690872; doi:10.1186/s13071-025-07109-y)
Supplement: Supplementary file 10 — Additional file 10: Tables S1-5. B-cell and CD4 T-cell epitopes predicted peptides of R. microplus proteins (Bm86, AQP1, AQP2, and VgR). [file 13071_2025_7109_MOESM10_ESM.pdf]

S-Table 1: Bm86 B- cell epitopes predicted peptides.

No.	Start	End	Bm86 Predicted peptides	aa No.
1	19	34	AESSICSDFGNEFCRN	16
2	38	45	EVVPGAED	8
3	54	77	DNMYFNAAEKQCEYKDTCKTRECS	24
4	96	129	SDDLTLQCKIKNDFATDCRNRGGTAKLRTDGFIG	34
5	139	147	AMNKTTRNC	9
6	152	188	CLRPDLTCKDLCEKNLLQRDSRCCQGWNTANCSAAPP	37
7	195	218	PGSPKGPDGQCKNACRTKEAGFVC	24
8	238	264	TVAEDGITCKSISYTVSCTVEQKQTCR	27
9	283	303	NQHLVGDTCISDCVDKKCHEE	21
10	306	311	DCGVYM	6
11	321	461	KSRKPGPNVNINECLLNEYYYTVSFTPNISFDSDHCKRYEDRVLEAIRTSIGKEV FKVEILNCTQDIKARLIAEKPLSKYVLRKLQACEHPIGEWCMMYPKLLIKKNSA TEIEEENLCDSLLKNQEAAYKGQNKCVKVDNL	141
12	470	504	YTTTYEMTRGRLRRSVCKAGVSCNENEQLECANKG	35
13	522	556	TKPGEIGCIERTTCNPKEIQECQDKKLECVYKNHK	35
14	565	589	HECSREPAKDSCSEEDNGKCQSSGQ	25
15	606	626	ATTASTTTTKAKDKDPDPEKS	21

S-Table 2: AQP1 B-cell epitopes predicted peptides:

No.	Start	End	AQP1 Predicted peptides	aa No.
1	35	40	GDNESL	6
2	111	127	AIEHFDQGIRQVTGEKA	17
3	166	175	TRNFGGIPPH	10
4	218	227	GPETFTLRGW	10
5	254	296	DHWPEKPKPAISTDGKETKEDLVETLYKV DGDKMVLELEPTQH	43

S-Table 3: AQP2 B- cell epitopes predicted peptides:

No.	Start	End	AQP2 Predicted peptides	aa No.
1	5	20	TVTRAWRQVTGCCIEN	16
2	49	55	VFQLGSV	7
3	128	156	LSQVDVNLAIVYGTNATAPVFSCFPAPGV	29
4	182	193	GRNMAVSRGQQP	12
5	233	243	GSAVFSFRSYN	11
6	268	289	DNHWKDEDEVDEDEKRPLLSNA	22

S-Table 4: B- cell epitopes of VgR predicted peptides:

No.	Start	End	VgR Predicted peptides	aa No.
1	9	26	LAVVALYLVGNVFSECPQ	18
2	36	206	CIAMFWRCDGQNDCGNHKDETGCSATHSRCPADKFACRDSSYCVPQIWVCDGEADCH DSSDEQDCHSSNCTGFRCHNNECIPAHWRCDQTEDCADASDELDCGGVQNSSTTTPTPR CDVDQGRFPCLDGQCLLPSKVCDGRKDCSDGADEGSFCKVNECSQKKCSQGCFVA	171
3	217	252	GFRLLADHISCADVDECAEDPHVCSHGCINSPGSYS	36
4	256	329	LEGYQLTDNSFCKARDPEPLLVFSTTKEIRGLWLRSNRYFEIHPAEAQAVGVEFDSDQHRV FWTDVSTRRSSIH	74
5	334	349	DGSDFKTLFSAEKTLL	16
6	422	428	GTNIQQL	7
7	502	519	SNKRTGKQHHRVLRENGH	18
8	529	543	PVLRQRGIQNPCWDN	15
9	555	577	SYMCLCRIGYKLSANKHSCAVTK	23
10	596	602	NKVGAPV	7
11	643	655	SFEQWTVHDHIGS	13
12	804	939	KASLEYIDRSSEPSVHRHVSLRSTRNGTFSRRVIVAAQVPPFAPGPCGLNNGGCSHTCLPV RTTDRSCFCPPGMALNADNRTCRVETSTCRPHELPCAGSCIAAVNWCDGHKDCSDNADE ESCGSATCPATDFSCS	136
13	941	978	GRCIEKEWQCDGYNDCGDSSDERNCTVTTCASHQYTCR	38
14	981	1239	VCLPLYWRCDGSEDCPDGDDELNCSSVRCPSAHSRCDNGQCIPQDWTCDGHSDCSDSS DEKNCTEQPSCFEDDFHCANGQCVDKRLRCDHDNDCEDSSDEVGCDYAKANRTKCSTG MVDCGDGQCIYTHDMCDGYIDCHNSRDERNCSAPICHSAEFFCTGTKRCILQNWLCDGD DDCGDGMDETLPRCHPTTKVSTTSVPACASNEFKCGSRECIAWSRVCDGRTDCADFSDE GTHCVSYCGTTNGGCAHLCRESPTG	259
15	1247	1280	GYRLNTDRKSCDDIDECATPGHCSHFCQNSKGSY	34
16	1288	1352	YALGADRRYCKVQYGEPFLLYMLPNQIRSFSMHGHAQHLLAEDSLSDMHGMDYRVTDK SIFWTEM	65
17	1364	1376	NGKQFTLLEDIHK	13
18	1387	1394	GNIYFTDG	8
19	1438	1445	EVVQKDHG	8
20	1536	1578	RYTGTHVGLVHHGTAKATVLKVLHAVHQPSGVNRCARNQCAHI	43
21	1595	1638	TLAEDAHKCVESDERYHINSSDILGQFCNPVCLNGGRCISGNDS	44
22	1646	1667	GFKGPSCTDTSVVSMLSQKSTS	22
23	1694	1743	RNRDKLAALDFSVSFKKPTFKKRQGLLEDEHPIAADEDYHAMNTTPGFIN	50
24	1748	1793	TRKTELLSEDGELKRWASSDSLQSSSSKEQSSCVLAGDMAAKQDKV	46

S-Table 5: The most promiscuous predicted CD4 T-cell epitopes

Protein	MHC Allele	Starting position	Strong Binders CD4 T-cell epitope	Phosphorylation site	Kinases	O-Gly	N-Gly
**Bm68**	BoLA-DRB3_00101	384	TQDIKARLIAEKPLS	384/Threonine	DNAPK	-	-
**Bm68**	BoLA-DRB3_01101	613	TTKAKDKDPDPEKSS	613/Threonine	PKC	-	-
**AQP1**	BoLA-DRB3_00101	109	KDAIEHFDQGIRQVT	107/Threonine	PKC	-	-
**VgR**	BoLA-DRB3_00101	312	DQHRVFWTDVSTRRS	311/Serine	CKII	-	-
**VgR**	BoLA-DRB3_00101	1738	TPGFINPAFNTRKTE	-		-	391/+
**VgR**	BoLA-DRB3_00101<br>BoLA-DRB3_01501<br>BoLA-DRB3_01601<br>BoLA-DRB3_01801	391	SPRAIIVNPPQKVVY	391/Serine	Unsp	-	-
**VgR**	BoLA-DRB3_01001<br>BoLA-DRB3_02002	775	GTPFIVQQVKAHISS	776/Threonine	Unsp	-	-
**VgR**	BoLA-DRB3_01201<br>BoLA-DRB3_02601	1510	SPFSIALFEDWLYWS	1510/ Serine	Unsp	1510/+	-
**VgR**	BoLA-DRB3_01201	311	SDQHRVFWTDVSTRR	311/Serine	CKII	311/+	-
**VgR**	BoLA-DRB3_01201	292	NRYFEIHPAEAQAVG	-	-	291/+	-
**VgR**	BoLA-DRB3_01201	638	AINVKSFEQWTVHDH	637/ Serine	Unsp	-	-
**VgR**	BoLA-DRB3_01201<br>BoLA-DRB3_01801<br>BoLA-DRB3_02002	1302	GEPFLLYMLPNQIRS	1301/ Y	EGFR	-	-
**VgR**	BoLA-DRB3_01201<br>BoLA-DRB3_02601	578	DFSFVIVAEEDLVYK	576/Threonine	PKC	-	-
**VgR**	BoLA-DRB3_01201	1344	DKSIFWTEMDEGVIN	1343/Threonine	PKC	-	-
**VgR**	BoLA-DRB3_01201	491	WSDWASYSLDSSNKR	492/Serine	CKII	-	-
**VgR**	BoLA-DRB3_01201	718	KPTITSYTMDGQNPR	720/Threonine	PKC	-	-
**VgR**	BoLA-DRB3_01201<br>BoLA-DRB3_01601<br>BoLA-DRB3_02601	1606	SDERYHINSSDILGQ	1606/Serine	Unsp	-	-
**VgR**	BoLA-DRB3_01101	575	VTKDFSFVIVAEEDL	576/Threonine	PKC	-	-
**VgR**	BoLA-DRB3_01101<br>BoLA-DRB3_01801<br>BoLA-DRB3_02002	455	LSSLEYLELSTLKRD	456/Threonine	CKII	-	-
**VgR**	BoLA-DRB3_01101	1552	ATVLKVLHAVHQPSG	1553/Threonine	PKC	-	-
**VgR**	BoLA-DRB3_01101	1463	DKILWPCSITVDAVH	1463/Threonine	PKC	-	-
**VgR**	BoLA-DRB3_01501<br>BoLA-DRB3_01801	834	RRVIVAAQVPPFAPG	833/Serine	Unsp	-	-
**VgR**	BoLA-DRB3_01501	1449	RSNMDGTARVVLLTD	1450/Serine	Unsp	-	-
**VgR**	BoLA-DRB3_01601<br>BoLA-DRB3_02601	1344	DKSIFWTEMDEGVIN	1343/Threonine	PKC	-	-
**VgR**	BoLA-DRB3_02601	487	DTVYWSDWASYSLDS	488/Threonine	Unsp	-	-
**VgR**	BoLA-DRB3_02601	834	RRVIVAAQVPPFAPG	833/Serine	Unsp	833/+	-
