# Supplementary material for: Comparative immunoinformatic analysis of Rhipicephalus microplus cocktail vaccine targets
Source: Parasit Vectors. 2025 Dec 9;18:502. doi: 10.1186/s13071-025-07109-y (PMC12690872; doi:10.1186/s13071-025-07109-y)
Supplement: Supplementary file 11 — Additional file 11: Dataset S2. T-cell epitopes peptides of R. microplus proteins (Bm86, AQP1, AQP2, and VgR) predicted by NetMHCIIpan Version 4.3d. [file 13071_2025_7109_MOESM11_ESM.pdf]

```
# NetMHCIIpan version 4.3d

# Input is in FASTA format

# Peptide length 15

# Prediction Mode: EL+BA with Context

# Threshold for Strong binding peptides (%Rank)  2.00%
# Threshold for Weak binding peptides (%Rank)    10.00%

# BoLA-DRB3_00101 : Distance to training data  0.000 (using nearest neighbor BoLA-DRB3_00101)

# Allele: BoLA-DRB3_00101
```

Pos	MHC	Peptide	Of	Core	Core_Rel	Inverted	Identity	Score_EL	%Rank_EL	Exp_Bind	Score_BA	%Rank_BA	Affinity(nM)	BindLevel
384	BoLA-DRB3_00101	TQDIKARLIAEKPLS	3	IKARLIAEK	1.000	0	Bm86_650_bp	0.631768	1.26	NA	0.350296	2.43	1129.64	<= SB
386	BoLA-DRB3_00101	DIKARLIAEKPLSKY	5	LIAEKPLSK	0.860	0	Bm86_650_bp	0.591412	1.50	NA	0.424877	0.60	504.06	<= SB
544	BoLA-DRB3_00101	QDKKLECVYKNHKAE	4	LECVYKNHK	1.000	0	Bm86_650_bp	0.575137	1.62	NA	0.229035	15.07	4195.09	<= SB
439	BoLA-DRB3_00101	DSLLKNQEAAYKGQN	3	LKNQEAAYK	0.690	0	Bm86_650_bp	0.483893	2.41	NA	0.255900	10.40	3136.92	<= WB
360	BoLA-DRB3_00101	EDRVLEAIRTSIGKE	3	VLEAIRTSI	0.850	0	Bm86_650_bp	0.461895	2.63	NA	0.284459	6.87	2303.06	<= WB
446	BoLA-DRB3_00101	EAAYKGQNKCVKVDN	3	YKGQNKCVK	0.990	0	Bm86_650_bp	0.307721	4.86	NA	0.237721	13.41	3818.79	<= WB
346	BoLA-DRB3_00101	TPNISFDSDHCKRYE	3	ISFDSDHCK	0.810	0	Bm86_650_bp	0.299085	5.04	NA	0.224117	16.10	4424.37	<= WB
613	BoLA-DRB3_00101	TTKAKDKDPDPEKSS	3	AKDKDPDPE	0.230	0	Bm86_650_bp	0.297976	5.06	NA	0.027859	96.53	36988.06	<= WB
419	BoLA-DRB3_00101	YPKLLIKKNSATEIE	5	IKKNSATEI	0.550	0	Bm86_650_bp	0.271264	5.73	NA	0.314743	4.34	1659.59	<= WB
54	BoLA-DRB3_00101	DNMYFNAAEKQCEYK	4	FNAAEKQCE	0.510	0	Bm86_650_bp	0.250128	6.30	NA	0.198005	22.64	5868.81	<= WB
471	BoLA-DRB3_00101	TTTYEMTRGRLRRSV	3	YEMTRGRLR	0.880	0	Bm86_650_bp	0.229630	6.94	NA	0.394712	1.09	698.60	<= WB

```
 365   BoLA-DRB3_00101      EAIRTSIGKEVFKVE    2   IRTSIGKEV     0.760
0     Bm86_650_bp      0.215657     7.39        NA      0.314647     4.35
1661.32   <= WB
 468   BoLA-DRB3_00101      DGYTTTYEMTRGRLR    2   YTTTYEMTR     0.500
0     Bm86_650_bp      0.182723     8.71        NA      0.301317     5.35
1919.07   <= WB
----------------------------------------------------------------------------
--------------------------------------------------------------
Number of strong binders: 3 Number of weak binders: 10
----------------------------------------------------------------------------
--------------------------------------------------------------
----------------------------------------------------------------------------
--------------------------------------------------------------
 Pos              MHC             Peptide   Of        Core  Core_Rel
Inverted        Identity      Score_EL %Rank_EL  Exp_Bind      Score_BA
%Rank_BA  Affinity(nM)  BindLevel
----------------------------------------------------------------------------
--------------------------------------------------------------
  68   BoLA-DRB3_00101      NPAVTLAQASVRKFP    3   VTLAQASVR     0.870
0     AQP1_299_bp      0.762666     0.61        NA      0.411490     0.79
582.63   <= SB
 285   BoLA-DRB3_00101      DKMVLELEPTQHQRL    6   LEPTQHQRL     0.440
0     AQP1_299_bp      0.578346     1.60        NA      0.201148    21.75
5672.59   <= SB
 109   BoLA-DRB3_00101      KDAIEHFDQGIRQVT    3   IEHFDQGIR     1.000
0     AQP1_299_bp      0.575987     1.61        NA      0.242584    12.51
3623.06   <= SB
 259   BoLA-DRB3_00101      KPKPAISTDGKETKE    5   ISTDGKETK     0.980
0     AQP1_299_bp      0.448113     2.78        NA      0.167116    32.91
8197.79   <= WB
  65   BoLA-DRB3_00101      SHLNPAVTLAQASVR    2   LNPAVTLAQ     0.800
0     AQP1_299_bp      0.388725     3.50        NA      0.292865     6.06
2102.84   <= WB
 284   BoLA-DRB3_00101      GDKMVLELEPTQHQR    5   LELEPTQHQ     0.600
0     AQP1_299_bp      0.356815     3.97        NA      0.178521    28.93
7246.11   <= WB
 128   BoLA-DRB3_00101      TAGIFATYPRPHVST    3   IFATYPRPH     0.790
0     AQP1_299_bp      0.271220     5.73        NA      0.304633     5.10
1851.44   <= WB
  71   BoLA-DRB3_00101      VTLAQASVRKFPIAK    2   LAQASVRKF     0.820
0     AQP1_299_bp      0.254243     6.18        NA      0.343746     2.71
1212.60   <= WB
 116   BoLA-DRB3_00101      DQGIRQVTGEKATAG    3   IRQVTGEKA     0.820
0     AQP1_299_bp      0.250876     6.28        NA      0.235981    13.72
3891.37   <= WB
  74   BoLA-DRB3_00101      AQASVRKFPIAKVPL    4   VRKFPIAKV     0.850
0     AQP1_299_bp      0.207779     7.67        NA      0.368800     1.76
924.68   <= WB
 272   BoLA-DRB3_00101      KEDLVETLYKVDGDK    4   VETLYKVDG     0.450
0     AQP1_299_bp      0.187103     8.52        NA      0.162099    34.89
8655.09   <= WB
----------------------------------------------------------------------------
--------------------------------------------------------------
Number of strong binders: 3 Number of weak binders: 8
----------------------------------------------------------------------------
--------------------------------------------------------------
```

Pos	MHC	Peptide	Of	Core	Core_Rel	Inverted	Identity	Score_EL	%Rank_EL	Exp_Bind	Score_BA	%Rank_BA	Affinity(nM)	BindLevel
84	BoLA-DRB3_00101	NPAVTVALATIGKLG	5	VALATIGKL	0.810	0	AQP2_293_bp	0.355114	4.00	NA	0.266563	8.96	2795.11	<= WB
74	BoLA-DRB3_00101	VAGGVSGAHLNPAVT	4	VSGAHLNPA	0.990	0	AQP2_293_bp	0.329851	4.45	NA	0.173539	30.61	7647.43	<= WB
279	BoLA-DRB3_00101	EDEKRPLLSNAKICA	3	KRPLLSNAK	0.390	0	AQP2_293_bp	0.179221	8.86	NA	0.178977	28.77	7210.45	<= WB
262	BoLA-DRB3_00101	IYKLAVDNHWKDEDE	3	LAVDNHWKD	0.840	0	AQP2_293_bp	0.157867	9.93	NA	0.192119	24.44	6254.73	<= WB

Number of strong binders: 0 Number of weak binders: 4

Pos	MHC	Peptide	Of	Core	Core_Rel	Inverted	Identity	Score_EL	%Rank_EL	Exp_Bind	Score_BA	%Rank_BA	Affinity(nM)	BindLevel
1738	BoLA-DRB3_00101	TPGFINPAFNTRKTE	4	INPAFNTRK	0.990	0	VgR_1799_bp	0.820893	0.39	NA	0.318826	4.09	1587.88	<= SB
271	BoLA-DRB3_00101	DPEPLLVFSTTKEIR	6	VFSTTKEIR	0.710	0	VgR_1799_bp	0.810884	0.42	NA	0.358782	2.11	1030.54	<= SB
312	BoLA-DRB3_00101	DQHRVFWTDVSTRRS	4	VFWTDVSTR	0.980	0	VgR_1799_bp	0.708569	0.81	NA	0.345130	2.65	1194.58	<= SB
391	BoLA-DRB3_00101	SPRAIIVNPPQKVVY	4	IIVNPPQKV	0.860	0	VgR_1799_bp	0.647941	1.16	NA	0.364663	1.89	967.01	<= SB
1698	BoLA-DRB3_00101	KLAALDFSVSFKKPT	4	LDFSVSFKK	1.000	0	VgR_1799_bp	0.634933	1.24	NA	0.396642	1.06	684.17	<= SB
1487	BoLA-DRB3_00101	KNVIESATYDGKDRK	3	IESATYDGK	0.880	0	VgR_1799_bp	0.629645	1.27	NA	0.206626	20.26	5346.14	<= SB
794	BoLA-DRB3_00101	HNEIHWTSRDKASLE	3	IHWTSRDKA	0.990	0	VgR_1799_bp	0.544366	1.86	NA	0.288027	6.51	2215.85	<= SB
294	BoLA-DRB3_00101	YFEIHPAEAQAVGVE	3	IHPAEAQAV	0.960	0	VgR_1799_bp	0.515615	2.11	NA	0.268858	8.63	2726.56	<= WB

686 BoLA-DRB3_00101 SGHVIIRDDLHRPVG 3 VIIRDDLHR 0.590
0 VgR_1799_bp 0.509649 2.17 NA 0.279804 7.36
2422.03 <= WB
803 BoLA-DRB3_00101 DKASLEYIDRSSEPS 4 LEYIDRSSE 1.000
0 VgR_1799_bp 0.505671 2.20 NA 0.194461 23.69
6098.23 <= WB
1785 BoLA-DRB3_00101 DMAAKQDKVFFFRKH 3 AKQDKVFFF 0.390
0 VgR_1799_bp 0.500179 2.26 NA 0.193596 23.95
6155.57 <= WB
1470 BoLA-DRB3_00101 SITVDAVHKRIYWSD 1 ITVDAVHKR 0.470
0 VgR_1799_bp 0.491747 2.34 NA 0.349322 2.47
1141.61 <= WB
815 BoLA-DRB3_00101 EPSVHRHVSLRSTRN 3 VHRHVSLRS 0.890
0 VgR_1799_bp 0.475275 2.49 NA 0.383993 1.36
784.51 <= WB
523 BoLA-DRB3_00101 GVHVYHPVLRQRGIQ 4 YHPVLRQRG 0.390
0 VgR_1799_bp 0.474934 2.49 NA 0.360975 2.03
1006.37 <= WB
775 BoLA-DRB3_00101 GTPFIVQQVKAHISS 4 IVQQVKAHI 0.620
0 VgR_1799_bp 0.455630 2.70 NA 0.322916 3.84
1519.14 <= WB
718 BoLA-DRB3_00101 KPTITSYTMDGQNPR 3 ITSYTMDGQ 0.690
0 VgR_1799_bp 0.455090 2.71 NA 0.155144 37.81
9331.54 <= WB
585 BoLA-DRB3_00101 AEEDLVYKIDLNKVG 4 LVYKIDLNK 0.890
0 VgR_1799_bp 0.436866 2.90 NA 0.264853 9.19
2847.30 <= WB
521 BoLA-DRB3_00101 IMGVHVYHPVLRQRG 3 VHVYHPVLR 0.670
0 VgR_1799_bp 0.422256 3.07 NA 0.382766 1.38
794.99 <= WB
1554 BoLA-DRB3_00101 VLKVLHAVHQPSGVN 3 VLHAVHQPS 0.800
0 VgR_1799_bp 0.416500 3.15 NA 0.284118 6.90
2311.58 <= WB
1477 BoLA-DRB3_00101 HKRIYWSDANKNVIE 3 IYWSDANKN 0.950
0 VgR_1799_bp 0.378355 3.65 NA 0.285287 6.78
2282.52 <= WB
495 BoLA-DRB3_00101 ASYSLDSSNKRTGKQ 4 LDSSNKRTG 0.890
0 VgR_1799_bp 0.373095 3.73 NA 0.231725 14.54
4074.75 <= WB
1310 BoLA-DRB3_00101 LPNQIRSFSMHGHAQ 4 IRSFSMHGH 0.940
0 VgR_1799_bp 0.354587 4.00 NA 0.359992 2.07
1017.13 <= WB
358 BoLA-DRB3_00101 ANNLYITDSLVKRIL 3 LYITDSLVK 0.680
0 VgR_1799_bp 0.346208 4.15 NA 0.367050 1.81
942.35 <= WB
623 BoLA-DRB3_00101 NQTLHYSDNRHSVLS 3 LHYSDNRHS 0.990
0 VgR_1799_bp 0.341017 4.25 NA 0.255717 10.43
3143.14 <= WB
1471 BoLA-DRB3_00101 ITVDAVHKRIYWSDA 2 VDAVHKRIY 0.930
0 VgR_1799_bp 0.326555 4.50 NA 0.361688 2.00
998.64 <= WB
1365 BoLA-DRB3_00101 GKQFTLLEDIHKPYH 3 FTLLEDIHK 0.510
0 VgR_1799_bp 0.324557 4.54 NA 0.260473 9.77
2985.49 <= WB
1551 BoLA-DRB3_00101 KATVLKVLHAVHQPS 3 VLKVLHAVH 0.640
0 VgR_1799_bp 0.308842 4.84 NA 0.356752 2.19
1053.42 <= WB

1702 BoLA-DRB3_00101 LDFSVSFKKPTFKKR 4 VSFKKPTFK 0.720
0 VgR_1799_bp 0.306122 4.89 NA 0.419008 0.67
537.11 <= WB
862 BoLA-DRB3_00101 LPVRTTDRSCFCPPG 2 VRTTDRSCF 0.950
0 VgR_1799_bp 0.268381 5.80 NA 0.243133 12.41
3601.60 <= WB
834 BoLA-DRB3_00101 RRVIVAAQVPPFAPG 3 IVAAQVPPF 0.940
0 VgR_1799_bp 0.248262 6.35 NA 0.259754 9.87
3008.80 <= WB
1286 BoLA-DRB3_00101 DGYALGADRRYCKVQ 4 LGADRRYCK 0.590
0 VgR_1799_bp 0.244372 6.46 NA 0.305642 5.03
1831.33 <= WB
1353 BoLA-DRB3_00101 DEGVINVMTLGNGKQ 4 INVMTLGNG 0.510
0 VgR_1799_bp 0.239273 6.62 NA 0.208866 19.68
5218.13 <= WB
1539 BoLA-DRB3_00101 GTHVGLVHHGTAKAT 5 LVHHGTAKA 0.560
0 VgR_1799_bp 0.239163 6.63 NA 0.261519 9.64
2951.89 <= WB
1599 BoLA-DRB3_00101 DAHKCVESDERYHIN 5 VESDERYHI 0.950
0 VgR_1799_bp 0.234047 6.79 NA 0.159382 36.03
8913.31 <= WB
467 BoLA-DRB3_00101 KRDVVMHEEVFHPFA 3 VVMHEEVFH 0.900
0 VgR_1799_bp 0.229767 6.93 NA 0.201488 21.65
5651.76 <= WB
435 BoLA-DRB3_00101 WPNGLTLDHTTNRLY 4 LTLDHTTNR 0.920
0 VgR_1799_bp 0.220341 7.24 NA 0.251308 11.05
3296.72 <= WB
688 BoLA-DRB3_00101 HVIIRDDLHRPVGVA 3 IRDDLHRPV 0.530
0 VgR_1799_bp 0.210443 7.57 NA 0.269266 8.57
2714.55 <= WB
518 BoLA-DRB3_00101 GHHIMGVHVYHPVLR 3 IMGVHVYHP 0.460
0 VgR_1799_bp 0.199128 8.01 NA 0.303408 5.20
1876.14 <= WB
1376 BoLA-DRB3_00101 KPYHIAVDWVAGNIY 4 IAVDWVAGN 0.780
0 VgR_1799_bp 0.189613 8.42 NA 0.217953 17.46
4729.50 <= WB
821 BoLA-DRB3_00101 HVSLRSTRNGTFSRR 3 LRSTRNGTF 0.950
0 VgR_1799_bp 0.185406 8.60 NA 0.289331 6.39
2184.80 <= WB
1437 BoLA-DRB3_00101 HEVVQKDHGLIERSN 3 VQKDHGLIE 0.430
0 VgR_1799_bp 0.181611 8.76 NA 0.247139 11.71
3448.83 <= WB
337 BoLA-DRB3_00101 DFKTLFSAEKTLLED 4 LFSAEKTLL 0.860
0 VgR_1799_bp 0.180515 8.81 NA 0.240047 12.98
3723.89 <= WB

Number of strong binders: 7 Number of weak binders: 35

BoLA-DRB3_01001 : Distance to training data 0.000 (using nearest neighbor BoLA-DRB3_01001)

Allele: BoLA-DRB3_01001

Pos	MHC	Peptide	Of	Core	Core_Rel	Inverted	Identity	Score_EL	%Rank_EL	Exp_Bind	Score_BA	%Rank_BA	Affinity(nM)	BindLevel
613	BoLA-DRB3_01001	TTKAKDKDPDPEKSS	3	AKDKDPDPE	0.240	0	Bm86_650_bp	0.356480	2.66	NA	0.073052	97.59	22683.02	<= WB
2	BoLA-DRB3_01001	RGIALFVAAVSLIVE	5	FVAAVSLIV	0.950	0	Bm86_650_bp	0.178073	5.36	NA	0.382106	36.38	800.69	<= WB
98	BoLA-DRB3_01001	DLTLQCKIKNDFATD	3	LQCKIKNDF	1.000	0	Bm86_650_bp	0.117411	7.36	NA	0.338036	50.78	1289.88	<= WB
419	BoLA-DRB3_01001	YPKLLIKKNSATEIE	5	IKKNSATEI	0.700	0	Bm86_650_bp	0.113938	7.51	NA	0.562368	2.84	113.87	<= WB
501	BoLA-DRB3_01001	ANKGQICVYENGKAN	5	ICVYENGKA	0.990	0	Bm86_650_bp	0.113654	7.53	NA	0.376338	38.22	852.25	<= WB
360	BoLA-DRB3_01001	EDRVLEAIRTSIGKE	3	VLEAIRTSI	0.990	0	Bm86_650_bp	0.112443	7.59	NA	0.429774	22.68	478.05	<= WB
336	BoLA-DRB3_01001	LNEYYYTVSFTPNIS	5	YTVSFTPNI	0.750	0	Bm86_650_bp	0.109852	7.72	NA	0.530288	5.19	161.12	<= WB
341	BoLA-DRB3_01001	YTVSFTPNISFDSDH	2	VSFTPNISF	0.770	0	Bm86_650_bp	0.074772	9.97	NA	0.592181	1.42	82.48	<= WB

Number of strong binders: 0 Number of weak binders: 8

Pos	MHC	Peptide	Of	Core	Core_Rel	Inverted	Identity	Score_EL	%Rank_EL	Exp_Bind	Score_BA	%Rank_BA	Affinity(nM)	BindLevel
127	BoLA-DRB3_01001	ATAGIFATYPRPHVS	5	FATYPRPHV	1.000	0	AQP1_299_bp	0.954353	0.11	NA	0.621532	0.63	60.04	<= SB
74	BoLA-DRB3_01001	AQASVRKFPIAKVPL	4	VRKFPIAKV	1.000	0	AQP1_299_bp	0.727000	0.68	NA	0.700904	0.04	25.44	<= SB
285	BoLA-DRB3_01001	DKMVLELEPTQHQRL	6	LEPTQHQRL	0.710	0	AQP1_299_bp	0.670789	0.89	NA	0.382891	36.12	793.92	<= SB
224	BoLA-DRB3_01001	LRGWNYVWVPLLGPH	3	WNYVWVPLL	0.640	0	AQP1_299_bp	0.338606	2.83	NA	0.637219	0.38	50.66	<= WB
86	BoLA-DRB3_01001	VPLYFAAQYLGGFVG	4	FAAQYLGGF	0.720	0	AQP1_299_bp	0.314542	3.07	NA	0.534586	4.80	153.80	<= WB

```
 100   BoLA-DRB3_01001       GAALVFATYKDAIEH    5   FATYKDAIE     0.720
0     AQP1_299_bp      0.231575     4.23         NA      0.557800     3.11
119.64   <= WB
 272   BoLA-DRB3_01001       KEDLVETLYKVDGDK    4   VETLYKVDG     0.850
0     AQP1_299_bp      0.228923     4.28         NA      0.399355    31.14
664.37   <= WB
 110   BoLA-DRB3_01001       DAIEHFDQGIRQVTG    2   IEHFDQGIR     0.710
0     AQP1_299_bp      0.145937     6.26         NA      0.383956    35.78
784.82   <= WB
 244   BoLA-DRB3_01001       GVWLYKVAIGDHWPE    4   YKVAIGDHW     0.840
0     AQP1_299_bp      0.111548     7.63         NA      0.529292     5.28
162.87   <= WB
 226   BoLA-DRB3_01001       GWNYVWVPLLGPHIG    5   WVPLLGPHI     0.580
0     AQP1_299_bp      0.096536     8.43         NA      0.563098     2.79
112.98   <= WB
 284   BoLA-DRB3_01001       GDKMVLELEPTQHQR    3   MVLELEPTQ     0.470
0     AQP1_299_bp      0.094871     8.52         NA      0.336466    51.30
1311.98   <= WB
 276   BoLA-DRB3_01001       VETLYKVDGDKMVLE    4   YKVDGDKMV     0.920
0     AQP1_299_bp      0.089105     8.90         NA      0.346784    47.87
1173.39   <= WB
 112   BoLA-DRB3_01001       IEHFDQGIRQVTGEK    3   FDQGIRQVT     0.890
0     AQP1_299_bp      0.085393     9.15         NA      0.329414    53.65
1416.00   <= WB
 140   BoLA-DRB3_01001       VSTLTCFIDQVIATG    3   LTCFIDQVI     0.960
0     AQP1_299_bp      0.084593     9.21         NA      0.365383    41.74
959.50   <= WB
-----------------------------------------------------------------------------
--------------------------------------------------------------
Number of strong binders: 3 Number of weak binders: 11
-----------------------------------------------------------------------------
--------------------------------------------------------------
-----------------------------------------------------------------------------
--------------------------------------------------------------
 Pos              MHC              Peptide   Of        Core  Core_Rel
Inverted        Identity      Score_EL %Rank_EL  Exp_Bind      Score_BA
%Rank_BA  Affinity(nM)  BindLevel
-----------------------------------------------------------------------------
--------------------------------------------------------------
 144   BoLA-DRB3_01001       TAPVFSCFPAPGVST    4   FSCFPAPGV     1.000
0     AQP2_293_bp      0.466287     1.89         NA      0.449769    17.95
385.05   <= SB
 239   BoLA-DRB3_01001       FRSYNWFWVPVVGPH    3   YNWFWVPVV     0.950
0     AQP2_293_bp      0.416180     2.21         NA      0.608194     0.91
69.36   <= WB
  89   BoLA-DRB3_01001       VALATIGKLGWCNVL    2   LATIGKLGW     1.000
0     AQP2_293_bp      0.365693     2.57         NA      0.587216     1.58
87.03   <= WB
 279   BoLA-DRB3_01001       EDEKRPLLSNAKICA    3   KRPLLSNAK     0.320
0     AQP2_293_bp      0.294344     3.32         NA      0.395467    32.28
692.92   <= WB
 116   BoLA-DRB3_01001       ASGLVYLVYADALSQ    5   YLVYADALS     0.970
0     AQP2_293_bp      0.278020     3.54         NA      0.477138    12.42
286.36   <= WB
 258   BoLA-DRB3_01001       IGVWIYKLAVDNHWK    5   YKLAVDNHW     0.830
0     AQP2_293_bp      0.174336     5.44         NA      0.573376     2.24
101.09   <= WB
```

43 BoLA-DRB3_01001 VLASLAVFQLGSVGL 4 LAVFQLGSV 0.880
0 AQP2_293_bp 0.168154 5.61 NA 0.303197 62.24
1880.43 <= WB
241 BoLA-DRB3_01001 SYNWFWVPVVGPHLG 5 WVPVVGPHL 0.610
0 AQP2_293_bp 0.136318 6.59 NA 0.538290 4.47
147.76 <= WB
135 BoLA-DRB3_01001 LAIVYGTNATAPVFS 4 YGTNATAPV 0.900
0 AQP2_293_bp 0.091187 8.76 NA 0.466839 14.37
320.12 <= WB

Number of strong binders: 1 Number of weak binders: 8

Pos MHC Peptide Of Core Core_Rel
Inverted Identity Score_EL %Rank_EL Exp_Bind Score_BA
%Rank_BA Affinity(nM) BindLevel
----

474 BoLA-DRB3_01001 EEVFHPFALAVFEDT 3 FHPFALAVF 1.000
0 VgR_1799_bp 0.913103 0.19 NA 0.628841 0.49
55.47 <= SB
1337 BoLA-DRB3_01001 GMDYRVTDKSIFWTE 3 YRVTDKSIF 1.000
0 VgR_1799_bp 0.894746 0.23 NA 0.527417 5.45
166.21 <= SB
775 BoLA-DRB3_01001 GTPFIVQQVKAHISS 4 IVQQVKAHI 0.980
0 VgR_1799_bp 0.892481 0.23 NA 0.601727 1.09
74.38 <= SB
1428 BoLA-DRB3_01001 DGLMFWAVWHEVVQK 5 WAVWHEVVQ 0.670
0 VgR_1799_bp 0.755938 0.60 NA 0.590994 1.46
83.54 <= SB
1785 BoLA-DRB3_01001 DMAAKQDKVFFFRKH 1 MAAKQDKVF 0.300
0 VgR_1799_bp 0.671385 0.89 NA 0.427996 23.13
487.34 <= SB
1684 BoLA-DRB3_01001 LLVLGYVLYRRNRDK 5 YVLYRRNRD 1.000
0 VgR_1799_bp 0.641754 1.01 NA 0.591808 1.43
82.81 <= SB
578 BoLA-DRB3_01001 DFSFVIVAEEDLVYK 3 FVIVAEEDL 0.880
0 VgR_1799_bp 0.560709 1.34 NA 0.418595 25.67
539.52 <= SB
794 BoLA-DRB3_01001 HNEIHWTSRDKASLE 5 WTSRDKASL 0.910
0 VgR_1799_bp 0.543357 1.43 NA 0.482346 11.52
270.67 <= SB
638 BoLA-DRB3_01001 AINVKSFEQWTVHDH 3 VKSFEQWTV 0.810
0 VgR_1799_bp 0.524461 1.53 NA 0.531484 5.08
159.05 <= SB
658 BoLA-DRB3_01001 GIDFDVTHQLLYWVD 3 FDVTHQLLY 0.990
0 VgR_1799_bp 0.479054 1.81 NA 0.472206 13.32
302.06 <= SB
641 BoLA-DRB3_01001 VKSFEQWTVHDHIGS 3 FEQWTVHDH 0.800
0 VgR_1799_bp 0.474154 1.84 NA 0.492593 9.81
242.26 <= SB
1701 BoLA-DRB3_01001 ALDFSVSFKKPTFKK 4 SVSFKKPTF 0.630
0 VgR_1799_bp 0.456472 1.94 NA 0.541502 4.21
142.71 <= SB

271 BoLA-DRB3_01001 DPEPLLVFSTTKEIR 5 LVFSTTKEI 0.500
0 VgR_1799_bp 0.408672 2.26 NA 0.536500 4.63
150.65 <= WB
718 BoLA-DRB3_01001 KPTITSYTMDGQNPR 3 ITSYTMDGQ 0.920
0 VgR_1799_bp 0.389162 2.39 NA 0.372387 39.49
889.48 <= WB
487 BoLA-DRB3_01001 DTVYWSDWASYSLDS 4 WSDWASYSL 1.000
0 VgR_1799_bp 0.380027 2.45 NA 0.457350 16.26
354.73 <= WB
391 BoLA-DRB3_01001 SPRAIIVNPPQKVVY 4 IIVNPPQKV 0.940
0 VgR_1799_bp 0.379569 2.46 NA 0.570790 2.37
103.95 <= WB
523 BoLA-DRB3_01001 GVHVYHPVLRQRGIQ 4 YHPVLRQRG 0.900
0 VgR_1799_bp 0.377120 2.47 NA 0.517623 6.50
184.79 <= WB
644 BoLA-DRB3_01001 FEQWTVHDHIGSVFG 3 WTVHDHIGS 1.000
0 VgR_1799_bp 0.345860 2.76 NA 0.439664 20.29
429.54 <= WB
1427 BoLA-DRB3_01001 NDGLMFWAVWHEVVQ 5 FWAVWHEVV 0.500
0 VgR_1799_bp 0.343546 2.78 NA 0.548086 3.72
132.90 <= WB
290 BoLA-DRB3_01001 RSNRYFEIHPAEAQA 5 FEIHPAEAQ 0.810
0 VgR_1799_bp 0.341777 2.80 NA 0.489125 10.37
251.53 <= WB
1478 BoLA-DRB3_01001 KRIYWSDANKNVIES 4 WSDANKNVI 0.790
0 VgR_1799_bp 0.332660 2.88 NA 0.497493 9.11
229.76 <= WB
1743 BoLA-DRB3_01001 NPAFNTRKTELLSED 3 FNTRKTELL 0.990
0 VgR_1799_bp 0.297975 3.28 NA 0.414517 26.85
563.85 <= WB
1301 BoLA-DRB3_01001 YGEPFLLYMLPNQIR 4 FLLYMLPNQ 0.930
0 VgR_1799_bp 0.289224 3.39 NA 0.619088 0.67
61.64 <= WB
1344 BoLA-DRB3_01001 DKSIFWTEMDEGVIN 5 WTEMDEGVI 0.580
0 VgR_1799_bp 0.281328 3.49 NA 0.398782 31.31
668.51 <= WB
553 BoLA-DRB3_01001 ADSYMCLCRIGYKLS 3 YMCLCRIGY 1.000
0 VgR_1799_bp 0.247214 3.96 NA 0.518856 6.37
182.34 <= WB
1455 BoLA-DRB3_01001 TARVVLLTDKILWPC 4 VLLTDKILW 0.960
0 VgR_1799_bp 0.237467 4.13 NA 0.525512 5.65
169.67 <= WB
1365 BoLA-DRB3_01001 GKQFTLLEDIHKPYH 3 FTLLEDIHK 0.690
0 VgR_1799_bp 0.237418 4.13 NA 0.464305 14.87
329.01 <= WB
834 BoLA-DRB3_01001 RRVIVAAQVPPFAPG 3 IVAAQVPPF 0.990
0 VgR_1799_bp 0.234241 4.18 NA 0.550051 3.59
130.11 <= WB
752 BoLA-DRB3_01001 RTLVWADAVRGTIES 4 WADAVRGTI 1.000
0 VgR_1799_bp 0.221279 4.42 NA 0.493646 9.65
239.52 <= WB
665 BoLA-DRB3_01001 HQLLYWVDADKYTLE 5 WVDADKYTL 0.850
0 VgR_1799_bp 0.214100 4.56 NA 0.475068 12.78
292.85 <= WB
1470 BoLA-DRB3_01001 SITVDAVHKRIYWSD 3 VDAVHKRIY 0.800
0 VgR_1799_bp 0.211249 4.62 NA 0.477013 12.44
286.75 <= WB

1606 BoLA-DRB3_01001 SDERYHINSSDILGQ 4 YHINSSDIL 0.990
0 VgR_1799_bp 0.210782 4.63 NA 0.363880 42.25
975.23 <= WB
803 BoLA-DRB3_01001 DKASLEYIDRSSEPS 4 LEYIDRSSE 0.930
0 VgR_1799_bp 0.208969 4.66 NA 0.340798 49.86
1251.90 <= WB
467 BoLA-DRB3_01001 KRDVVMHEEVFHPFA 3 VVMHEEVFH 0.900
0 VgR_1799_bp 0.199557 4.86 NA 0.411359 27.75
583.45 <= WB
306 BoLA-DRB3_01001 GVEFDSDQHRVFWTD 3 FDSDQHRVF 0.990
0 VgR_1799_bp 0.197875 4.90 NA 0.348796 47.22
1148.12 <= WB
399 BoLA-DRB3_01001 PPQKVVYWTDWGSRP 4 VVYWTDWGS 0.990
0 VgR_1799_bp 0.196172 4.93 NA 0.385430 35.30
772.41 <= WB
491 BoLA-DRB3_01001 WSDWASYSLDSSNKR 3 WASYSLDSS 0.890
0 VgR_1799_bp 0.195697 4.94 NA 0.445319 18.97
404.04 <= WB
337 BoLA-DRB3_01001 DFKTLFSAEKTLLED 4 LFSAEKTLL 0.900
0 VgR_1799_bp 0.166372 5.66 NA 0.449767 17.95
385.06 <= WB
730 BoLA-DRB3_01001 NPRVLPLSTLLLPVS 3 VLPLSTLLL 0.960
0 VgR_1799_bp 0.163840 5.73 NA 0.515009 6.81
190.09 <= WB
478 BoLA-DRB3_01001 HPFALAVFEDTVYWS 4 LAVFEDTVY 0.980
0 VgR_1799_bp 0.159195 5.85 NA 0.445818 18.86
401.87 <= WB
1491 BoLA-DRB3_01001 ESATYDGKDRKLVRG 4 YDGKDRKLV 1.000
0 VgR_1799_bp 0.152841 6.03 NA 0.315194 58.35
1651.52 <= WB
1756 BoLA-DRB3_01001 EDGELKRWASSDSLQ 4 LKRWASSDS 0.930
0 VgR_1799_bp 0.146151 6.26 NA 0.345810 48.19
1185.82 <= WB
1471 BoLA-DRB3_01001 ITVDAVHKRIYWSDA 3 DAVHKRIYW 0.390
0 VgR_1799_bp 0.144897 6.30 NA 0.506809 7.86
207.73 <= WB
1413 BoLA-DRB3_01001 DTAYSHLNTFALSAN 3 YSHLNTFAL 0.980
0 VgR_1799_bp 0.143779 6.34 NA 0.478659 12.15
281.69 <= WB
1783 BoLA-DRB3_01001 AGDMAAKQDKVFFFR 3 MAAKQDKVF 0.730
0 VgR_1799_bp 0.135588 6.62 NA 0.456509 16.45
357.97 <= WB
686 BoLA-DRB3_01001 SGHVIIRDDLHRPVG 3 VIIRDDLHR 0.630
0 VgR_1799_bp 0.134488 6.66 NA 0.411107 27.82
585.05 <= WB
1310 BoLA-DRB3_01001 LPNQIRSFSMHGHAQ 4 IRSFSMHGH 1.000
0 VgR_1799_bp 0.127433 6.93 NA 0.571180 2.35
103.52 <= WB
862 BoLA-DRB3_01001 LPVRTTDRSCFCPPG 2 VRTTDRSCF 0.980
0 VgR_1799_bp 0.125788 6.99 NA 0.432930 21.89
462.00 <= WB
1376 BoLA-DRB3_01001 KPYHIAVDWVAGNIY 2 YHIAVDWVA 0.690
0 VgR_1799_bp 0.120383 7.23 NA 0.511157 7.29
198.18 <= WB
792 BoLA-DRB3_01001 AAHNEIHWTSRDKAS 5 IHWTSRDKA 0.950
0 VgR_1799_bp 0.114536 7.48 NA 0.384309 35.66
781.83 <= WB

```
 663   BoLA-DRB3_01001      VTHQLLYWVDADKYT    4   LLYWVDADK     0.470
0     VgR_1799_bp      0.112036     7.61        NA      0.467076    14.32
319.30   <= WB
1434   BoLA-DRB3_01001      AVWHEVVQKDHGLIE    5   VVQKDHGLI     0.870
0     VgR_1799_bp      0.110453     7.69        NA      0.482177    11.55
271.17   <= WB
 697   BoLA-DRB3_01001      RPVGVALYPFAGVLF    6   LYPFAGVLF     0.830
0     VgR_1799_bp      0.109872     7.72        NA      0.516187     6.67
187.68   <= WB
1314   BoLA-DRB3_01001      IRSFSMHGHAQHLLA    3   FSMHGHAQH     0.940
0     VgR_1799_bp      0.107058     7.86        NA      0.516380     6.65
187.29   <= WB
1510   BoLA-DRB3_01001      SPFSIALFEDWLYWS    4   IALFEDWLY     0.990
0     VgR_1799_bp      0.102556     8.10        NA      0.379043    37.36
827.67   <= WB
 580   BoLA-DRB3_01001      SFVIVAEEDLVYKID    3   IVAEEDLVY     0.770
0     VgR_1799_bp      0.095930     8.46        NA      0.397143    31.79
680.47   <= WB
1729   BoLA-DRB3_01001      DEDYHAMNTTPGFIN    3   YHAMNTTPG     0.900
0     VgR_1799_bp      0.094539     8.54        NA      0.356313    44.73
1058.44   <= WB
 294   BoLA-DRB3_01001      YFEIHPAEAQAVGVE    3   IHPAEAQAV     0.620
0     VgR_1799_bp      0.092205     8.69        NA      0.408112    28.66
604.32   <= WB
1552   BoLA-DRB3_01001      ATVLKVLHAVHQPSG    3   LKVLHAVHQ     0.820
0     VgR_1799_bp      0.091290     8.75        NA      0.529754     5.24
162.06   <= WB
1709   BoLA-DRB3_01001      KKPTFKKRQGLLEDE    4   FKKRQGLLE     0.810
0     VgR_1799_bp      0.091244     8.76        NA      0.378487    37.54
832.67   <= WB
 445   BoLA-DRB3_01001      TNRLYWCDAKLSSLE    4   YWCDAKLSS     0.910
0     VgR_1799_bp      0.089002     8.90        NA      0.451754    17.50
376.87   <= WB
1516   BoLA-DRB3_01001      LFEDWLYWSDWGSDS    4   WLYWSDWGS     1.000
0     VgR_1799_bp      0.088750     8.92        NA      0.290770    66.14
2151.05   <= WB
1245   BoLA-DRB3_01001      HPGYRLNTDRKSCDD    3   YRLNTDRKS     0.940
0     VgR_1799_bp      0.084983     9.18        NA      0.305428    61.53
1835.58   <= WB
1051   BoLA-DRB3_01001      EDDFHCANGQCVDKR    3   FHCANGQCV     1.000
0     VgR_1799_bp      0.083042     9.32        NA      0.301549    62.77
1914.26   <= WB
1449   BoLA-DRB3_01001      RSNMDGTARVVLLTD    3   MDGTARVVL     0.980
0     VgR_1799_bp      0.079866     9.56        NA      0.390763    33.67
729.10   <= WB
  33   BoLA-DRB3_01001      NGRCIAMFWRCDGQN    4   IAMFWRCDG     0.970
0     VgR_1799_bp      0.078076     9.70        NA      0.438898    20.47
433.11   <= WB
1472   BoLA-DRB3_01001      TVDAVHKRIYWSDAN    1   VDAVHKRIY     0.470
0     VgR_1799_bp      0.076802     9.81        NA      0.463271    15.07
332.72   <= WB
----------------------------------------------------------------------------
--------------------------------------------------------------
Number of strong binders: 12 Number of weak binders: 55
----------------------------------------------------------------------------
--------------------------------------------------------------
```

```
# BoLA-DRB3_01101 : Distance to training data  0.000 (using nearest neighbor
BoLA-DRB3_01101)

# Allele: BoLA-DRB3_01101
--------------------------------------------------------------------------------
--------------------------------------------------------------
 Pos               MHC              Peptide   Of        Core  Core_Rel
Inverted        Identity      Score_EL %Rank_EL  Exp_Bind      Score_BA
%Rank_BA  Affinity(nM)  BindLevel
--------------------------------------------------------------------------------
--------------------------------------------------------------
 613   BoLA-DRB3_01101      TTKAKDKDPDPEKSS    3   AKDKDPDPE     0.580
0     Bm86_650_bp      0.490696     1.97         NA      0.046179    96.98
30337.26   <= SB
 374   BoLA-DRB3_01101      EVFKVEILNCTQDIK    4   VEILNCTQD     1.000
0     Bm86_650_bp      0.381918     2.95         NA      0.372641     4.18
887.04   <= WB
 636   BoLA-DRB3_01101      LLLLLAATSVTAASL    4   LAATSVTAA     0.610
0     Bm86_650_bp      0.248213     4.93         NA      0.218058    47.37
4724.13   <= WB
 360   BoLA-DRB3_01101      EDRVLEAIRTSIGKE    4   LEAIRTSIG     0.970
0     Bm86_650_bp      0.164599     7.36         NA      0.270639    26.55
2674.52   <= WB
   2   BoLA-DRB3_01101      RGIALFVAAVSLIVE    6   VAAVSLIVE     0.760
0     Bm86_650_bp      0.161804     7.47         NA      0.304697    16.17
1850.15   <= WB
 419   BoLA-DRB3_01101      YPKLLIKKNSATEIE    4   LIKKNSATE     0.650
0     Bm86_650_bp      0.135561     8.62         NA      0.308662    15.10
1772.46   <= WB
--------------------------------------------------------------------------------
--------------------------------------------------------------
Number of strong binders: 1 Number of weak binders: 5
--------------------------------------------------------------------------------
--------------------------------------------------------------
--------------------------------------------------------------------------------
--------------------------------------------------------------
 Pos               MHC              Peptide   Of        Core  Core_Rel
Inverted        Identity      Score_EL %Rank_EL  Exp_Bind      Score_BA
%Rank_BA  Affinity(nM)  BindLevel
--------------------------------------------------------------------------------
--------------------------------------------------------------
  24   BoLA-DRB3_01101      IGDSIMAIIIAGDNE    4   IMAIIIAGD     1.000
0     AQP1_299_bp      0.972288     0.03         NA      0.386652     3.02
762.26   <= SB
 285   BoLA-DRB3_01101      DKMVLELEPTQHQRL    4   LELEPTQHQ     0.310
0     AQP1_299_bp      0.775162     0.53         NA      0.276055    24.75
2522.30   <= SB
 284   BoLA-DRB3_01101      GDKMVLELEPTQHQR    3   MVLELEPTQ     0.390
0     AQP1_299_bp      0.581480     1.37         NA      0.289045    20.60
2191.57   <= SB
 272   BoLA-DRB3_01101      KEDLVETLYKVDGDK    3   LVETLYKVD     0.850
0     AQP1_299_bp      0.522701     1.74         NA      0.344229     7.82
1206.28   <= SB
  74   BoLA-DRB3_01101      AQASVRKFPIAKVPL    4   VRKFPIAKV     1.000
0     AQP1_299_bp      0.445944     2.34         NA      0.469080     0.21
312.45   <= WB
```

```
 135   BoLA-DRB3_01101      YPRPHVSTLTCFIDQ    5   VSTLTCFID     0.980
0     AQP1_299_bp      0.353259     3.29         NA      0.285319    21.74
2281.73   <= WB
 224   BoLA-DRB3_01101      LRGWNYVWVPLLGPH    3   WNYVWVPLL     0.540
0     AQP1_299_bp      0.346865     3.37         NA      0.420420     1.18
528.97   <= WB
 154   BoLA-DRB3_01101      GIMMVCVEAIGDTRN    3   MVCVEAIGD     0.960
0     AQP1_299_bp      0.336613     3.49         NA      0.277917    24.12
2471.99   <= WB
 116   BoLA-DRB3_01101      DQGIRQVTGEKATAG    3   IRQVTGEKA     0.970
0     AQP1_299_bp      0.268115     4.54         NA      0.241372    37.63
3670.88   <= WB
   3   BoLA-DRB3_01101      IENLLIRQLINEFLG    4   LIRQLINEF     0.400
0     AQP1_299_bp      0.183930     6.64         NA      0.383448     3.26
789.15   <= WB
  99   BoLA-DRB3_01101      VGAALVFATYKDAIE    3   ALVFATYKD     0.610
0     AQP1_299_bp      0.179630     6.79         NA      0.366255     4.86
950.49   <= WB
 100   BoLA-DRB3_01101      GAALVFATYKDAIEH    5   FATYKDAIE     0.580
0     AQP1_299_bp      0.173484     7.01         NA      0.430798     0.84
472.78   <= WB
  80   BoLA-DRB3_01101      KFPIAKVPLYFAAQY    3   IAKVPLYFA     0.950
0     AQP1_299_bp      0.167176     7.26         NA      0.406345     1.76
615.98   <= WB
 244   BoLA-DRB3_01101      GVWLYKVAIGDHWPE    3   LYKVAIGDH     0.720
0     AQP1_299_bp      0.165699     7.32         NA      0.422489     1.11
517.26   <= WB
  86   BoLA-DRB3_01101      VPLYFAAQYLGGFVG    4   FAAQYLGGF     0.650
0     AQP1_299_bp      0.162609     7.44         NA      0.330380    10.23
1401.28   <= WB
 241   BoLA-DRB3_01101      AILGVWLYKVAIGDH    4   VWLYKVAIG     0.480
0     AQP1_299_bp      0.128237     9.01         NA      0.438530     0.65
434.84   <= WB
 226   BoLA-DRB3_01101      GWNYVWVPLLGPHIG    3   YVWVPLLGP     0.620
0     AQP1_299_bp      0.112169    10.00         NA      0.410448     1.56
589.23   <= WB
----------------------------------------------------------------------------
-------------------------------------------------------------
Number of strong binders: 4 Number of weak binders: 13
----------------------------------------------------------------------------
-------------------------------------------------------------
----------------------------------------------------------------------------
-------------------------------------------------------------
 Pos              MHC             Peptide   Of        Core  Core_Rel
Inverted        Identity      Score_EL %Rank_EL  Exp_Bind      Score_BA
%Rank_BA  Affinity(nM)  BindLevel
----------------------------------------------------------------------------
-------------------------------------------------------------
 262   BoLA-DRB3_01101      IYKLAVDNHWKDEDE    3   LAVDNHWKD     0.960
0     AQP2_293_bp      0.381931     2.95         NA      0.350548     6.89
1126.56   <= WB
 256   BoLA-DRB3_01101      AVIGVWIYKLAVDNH    4   VWIYKLAVD     0.820
0     AQP2_293_bp      0.378692     2.99         NA      0.484251     0.11
265.15   <= WB
 125   BoLA-DRB3_01101      ADALSQVDVNLAIVY    3   LSQVDVNLA     1.000
0     AQP2_293_bp      0.262689     4.65         NA      0.311953    14.29
1710.46   <= WB
```

```
 279   BoLA-DRB3_01101       EDEKRPLLSNAKICA    3   KRPLLSNAK     0.570
0     AQP2_293_bp      0.257123     4.76         NA      0.186151    60.84
6671.94   <= WB
  43   BoLA-DRB3_01101       VLASLAVFQLGSVGL    4   LAVFQLGSV     0.630
0     AQP2_293_bp      0.227930     5.39         NA      0.152611    74.43
9590.81   <= WB
  74   BoLA-DRB3_01101       VAGGVSGAHLNPAVT    4   VSGAHLNPA     1.000
0     AQP2_293_bp      0.222784     5.51         NA      0.278193    24.03
2464.62   <= WB
 170   BoLA-DRB3_01101       AVLLLGICAITDGRN    3   LLGICAITD     0.910
0     AQP2_293_bp      0.195398     6.28         NA      0.372886     4.15
884.69   <= WB
  18   BoLA-DRB3_01101       IENTLARQALAEMVG    4   LARQALAEM     0.930
0     AQP2_293_bp      0.178317     6.84         NA      0.335173     9.33
1330.46   <= WB
  84   BoLA-DRB3_01101       NPAVTVALATIGKLG    5   VALATIGKL     0.880
0     AQP2_293_bp      0.164583     7.36         NA      0.288136    20.88
2213.24   <= WB
 259   BoLA-DRB3_01101       GVWIYKLAVDNHWKD    3   IYKLAVDNH     0.620
0     AQP2_293_bp      0.152958     7.83         NA      0.454630     0.38
365.32   <= WB
  39   BoLA-DRB3_01101       VGDCVLASLAVFQLG    5   LASLAVFQL     0.980
0     AQP2_293_bp      0.148920     7.99         NA      0.261143    29.85
2963.92   <= WB
 151   BoLA-DRB3_01101       FPAPGVSTLTCLLDQ    5   VSTLTCLLD     1.000
0     AQP2_293_bp      0.144615     8.19         NA      0.217964    47.41
4728.94   <= WB
 116   BoLA-DRB3_01101       ASGLVYLVYADALSQ    3   LVYLVYADA     0.450
0     AQP2_293_bp      0.134767     8.66         NA      0.387711     2.94
753.58   <= WB
 118   BoLA-DRB3_01101       GLVYLVYADALSQVD    4   LVYADALSQ     0.810
0     AQP2_293_bp      0.122800     9.33         NA      0.326120    11.08
1467.38   <= WB
-----------------------------------------------------------------------------
--------------------------------------------------------------
Number of strong binders: 0 Number of weak binders: 14
-----------------------------------------------------------------------------
--------------------------------------------------------------
-----------------------------------------------------------------------------
--------------------------------------------------------------
 Pos                MHC               Peptide   Of        Core  Core_Rel
Inverted        Identity      Score_EL %Rank_EL  Exp_Bind      Score_BA
%Rank_BA  Affinity(nM)  BindLevel
-----------------------------------------------------------------------------
--------------------------------------------------------------
 271   BoLA-DRB3_01101       DPEPLLVFSTTKEIR    4   LLVFSTTKE     0.950
0     VgR_1799_bp      0.883352     0.22         NA      0.400885     2.05
653.47   <= SB
 575   BoLA-DRB3_01101       VTKDFSFVIVAEEDL    4   FSFVIVAEE     0.990
0     VgR_1799_bp      0.750084     0.61         NA      0.453862     0.39
368.37   <= SB
1353   BoLA-DRB3_01101       DEGVINVMTLGNGKQ    4   INVMTLGNG     0.760
0     VgR_1799_bp      0.698113     0.82         NA      0.327098    10.88
1451.93   <= SB
 455   BoLA-DRB3_01101       LSSLEYLELSTLKRD    3   LEYLELSTL     0.810
0     VgR_1799_bp      0.613447     1.20         NA      0.404508     1.85
628.35   <= SB
```

1323 BoLA-DRB3_01101 AQHLLAEDSLSDMHG 3 LLAEDSLSD 0.990
0 VgR_1799_bp 0.576745 1.39 NA 0.335453 9.28
1326.44 <= SB
1552 BoLA-DRB3_01101 ATVLKVLHAVHQPSG 3 LKVLHAVHQ 0.900
0 VgR_1799_bp 0.568028 1.45 NA 0.411699 1.51
581.31 <= SB
803 BoLA-DRB3_01101 DKASLEYIDRSSEPS 4 LEYIDRSSE 0.990
0 VgR_1799_bp 0.558427 1.51 NA 0.325811 11.14
1472.29 <= SB
718 BoLA-DRB3_01101 KPTITSYTMDGQNPR 3 ITSYTMDGQ 0.990
0 VgR_1799_bp 0.553536 1.54 NA 0.294900 18.83
2057.04 <= SB
737 BoLA-DRB3_01101 STLLLPVSISVDLVA 3 LLPVSISVD 1.000
0 VgR_1799_bp 0.548882 1.57 NA 0.413265 1.44
571.54 <= SB
339 BoLA-DRB3_01101 KTLFSAEKTLLEDLS 3 FSAEKTLLE 1.000
0 VgR_1799_bp 0.548062 1.58 NA 0.330390 10.23
1401.13 <= SB
1785 BoLA-DRB3_01101 DMAAKQDKVFFFRKH 3 AKQDKVFFF 0.480
0 VgR_1799_bp 0.508387 1.83 NA 0.248701 34.66
3391.03 <= SB
1463 BoLA-DRB3_01101 DKILWPCSITVDAVH 3 LWPCSITVD 0.980
0 VgR_1799_bp 0.495250 1.93 NA 0.382901 3.31
793.83 <= SB
747 BoLA-DRB3_01101 VDLVARTLVWADAVR 3 VARTLVWAD 1.000
0 VgR_1799_bp 0.475817 2.09 NA 0.356336 6.08
1058.18 <= WB
757 BoLA-DRB3_01101 ADAVRGTIESLDLQK 3 VRGTIESLD 1.000
0 VgR_1799_bp 0.452295 2.29 NA 0.303409 16.52
1876.12 <= WB
474 BoLA-DRB3_01101 EEVFHPFALAVFEDT 3 FHPFALAVF 0.860
0 VgR_1799_bp 0.415915 2.61 NA 0.458017 0.33
352.18 <= WB
760 BoLA-DRB3_01101 VRGTIESLDLQKVFT 4 IESLDLQKV 1.000
0 VgR_1799_bp 0.404902 2.72 NA 0.330716 10.16
1396.19 <= WB
775 BoLA-DRB3_01101 GTPFIVQQVKAHISS 3 FIVQQVKAH 0.780
0 VgR_1799_bp 0.404409 2.72 NA 0.411226 1.53
584.29 <= WB
730 BoLA-DRB3_01101 NPRVLPLSTLLLPVS 3 VLPLSTLLL 0.820
0 VgR_1799_bp 0.396159 2.81 NA 0.413398 1.44
570.72 <= WB
1344 BoLA-DRB3_01101 DKSIFWTEMDEGVIN 3 IFWTEMDEG 0.890
0 VgR_1799_bp 0.389728 2.87 NA 0.319023 12.63
1584.49 <= WB
834 BoLA-DRB3_01101 RRVIVAAQVPPFAPG 3 IVAAQVPPF 0.990
0 VgR_1799_bp 0.378877 2.98 NA 0.407240 1.71
610.04 <= WB
585 BoLA-DRB3_01101 AEEDLVYKIDLNKVG 5 VYKIDLNKV 0.930
0 VgR_1799_bp 0.376477 3.01 NA 0.367632 4.71
936.44 <= WB
457 BoLA-DRB3_01101 SLEYLELSTLKRDVV 4 LELSTLKRD 0.810
0 VgR_1799_bp 0.314859 3.79 NA 0.403310 1.91
636.54 <= WB
1310 BoLA-DRB3_01101 LPNQIRSFSMHGHAQ 4 IRSFSMHGH 1.000
0 VgR_1799_bp 0.299068 4.01 NA 0.409445 1.61
595.66 <= WB

297 BoLA-DRB3_01101 IHPAEAQAVGVEFDS 5 AQAVGVEFD 0.620
0 VgR_1799_bp 0.294772 4.08 NA 0.260105 30.24
2997.40 <= WB
1431 BoLA-DRB3_01101 MFWAVWHEVVQKDHG 4 VWHEVVQKD 1.000
0 VgR_1799_bp 0.256246 4.77 NA 0.396679 2.31
683.89 <= WB
1302 BoLA-DRB3_01101 GEPFLLYMLPNQIRS 4 LLYMLPNQI 0.610
0 VgR_1799_bp 0.248983 4.92 NA 0.484499 0.11
264.44 <= WB
1365 BoLA-DRB3_01101 GKQFTLLEDIHKPYH 3 FTLLEDIHK 0.800
0 VgR_1799_bp 0.248671 4.92 NA 0.337641 8.89
1295.40 <= WB
292 BoLA-DRB3_01101 NRYFEIHPAEAQAVG 3 FEIHPAEAQ 0.600
0 VgR_1799_bp 0.238141 5.15 NA 0.325050 11.31
1484.46 <= WB
1415 BoLA-DRB3_01101 AYSHLNTFALSANDG 4 LNTFALSAN 0.930
0 VgR_1799_bp 0.230330 5.33 NA 0.374478 3.99
869.58 <= WB
1361 BoLA-DRB3_01101 TLGNGKQFTLLEDIH 4 GKQFTLLED 0.940
0 VgR_1799_bp 0.203593 6.04 NA 0.346480 7.49
1177.26 <= WB
764 BoLA-DRB3_01101 IESLDLQKVFTGTPF 3 LDLQKVFTG 0.960
0 VgR_1799_bp 0.193673 6.33 NA 0.324029 11.52
1500.95 <= WB
663 BoLA-DRB3_01101 VTHQLLYWVDADKYT 5 LYWVDADKY 0.730
0 VgR_1799_bp 0.189233 6.46 NA 0.391189 2.68
725.75 <= WB
467 BoLA-DRB3_01101 KRDVVMHEEVFHPFA 3 VVMHEEVFH 0.590
0 VgR_1799_bp 0.187890 6.50 NA 0.333761 9.59
1350.94 <= WB
686 BoLA-DRB3_01101 SGHVIIRDDLHRPVG 4 IIRDDLHRP 0.770
0 VgR_1799_bp 0.173083 7.02 NA 0.268037 27.42
2750.88 <= WB
638 BoLA-DRB3_01101 AINVKSFEQWTVHDH 3 VKSFEQWTV 0.800
0 VgR_1799_bp 0.172838 7.03 NA 0.353176 6.51
1094.98 <= WB
1427 BoLA-DRB3_01101 NDGLMFWAVWHEVVQ 3 LMFWAVWHE 0.830
0 VgR_1799_bp 0.169630 7.16 NA 0.307782 15.34
1789.42 <= WB
391 BoLA-DRB3_01101 SPRAIIVNPPQKVVY 4 IIVNPPQKV 0.860
0 VgR_1799_bp 0.163040 7.42 NA 0.316536 13.20
1627.71 <= WB
294 BoLA-DRB3_01101 YFEIHPAEAQAVGVE 3 IHPAEAQAV 0.850
0 VgR_1799_bp 0.162408 7.45 NA 0.266601 27.90
2793.96 <= WB
388 BoLA-DRB3_01101 SVDSPRAIIVNPPQK 4 PRAIIVNPP 0.790
0 VgR_1799_bp 0.152258 7.86 NA 0.210149 50.75
5146.19 <= WB
1554 BoLA-DRB3_01101 VLKVLHAVHQPSGVN 4 LHAVHQPSG 0.590
0 VgR_1799_bp 0.151500 7.89 NA 0.365546 4.93
957.81 <= WB
580 BoLA-DRB3_01101 SFVIVAEEDLVYKID 3 IVAEEDLVY 0.900
0 VgR_1799_bp 0.150730 7.92 NA 0.304163 16.31
1860.88 <= WB
289 BoLA-DRB3_01101 LRSNRYFEIHPAEAQ 3 NRYFEIHPA 0.460
0 VgR_1799_bp 0.147354 8.06 NA 0.409882 1.59
592.85 <= WB

1376   BoLA-DRB3_01101   KPYHIAVDWVAGNIY   4   IAVDWVAGN   0.640
0   VgR_1799_bp   0.146478   8.10   NA   0.349847   6.99
1135.14   <= WB
 516   BoLA-DRB3_01101   ENGHHIMGVHVYHPV   5   IMGVHVYHP   0.740
0   VgR_1799_bp   0.144013   8.22   NA   0.363695   5.15
977.19   <= WB
  79   BoLA-DRB3_01101   VPQIWVCDGEADCHD   3   IWVCDGEAD   0.970
0   VgR_1799_bp   0.132784   8.77   NA   0.216173   48.17
4821.47   <= WB
 706   BoLA-DRB3_01101   FAGVLFVLSAGDKPT   3   VLFVLSAGD   0.960
0   VgR_1799_bp   0.128024   9.02   NA   0.300268   17.36
1940.97   <= WB
 606   BoLA-DRB3_01101   LPVHNLGIISALTFD   5   LGIISALTF   0.590
0   VgR_1799_bp   0.123706   9.28   NA   0.308995   15.01
1766.09   <= WB
1539   BoLA-DRB3_01101   GTHVGLVHHGTAKAT   5   LVHHGTAKA   0.710
0   VgR_1799_bp   0.119901   9.50   NA   0.271357   26.31
2653.82   <= WB
1485   BoLA-DRB3_01101   ANKNVIESATYDGKD   4   VIESATYDG   0.530
0   VgR_1799_bp   0.118831   9.57   NA   0.177573   64.47
7320.82   <= WB
1729   BoLA-DRB3_01101   DEDYHAMNTTPGFIN   3   YHAMNTTPG   0.950
0   VgR_1799_bp   0.114523   9.85   NA   0.191933   58.40
6267.33   <= WB

Number of strong binders: 12 Number of weak binders: 38

BoLA-DRB3_01201 : Distance to training data 0.000 (using nearest neighbor BoLA-DRB3_01201)

Allele: BoLA-DRB3_01201

 Pos   MHC   Peptide   Of   Core Core_Rel
Inverted   Identity   Score_EL %Rank_EL Exp_Bind   Score_BA
%Rank_BA Affinity(nM) BindLevel

----
 501   BoLA-DRB3_01201   ANKGQICVYENGKAN   5   ICVYENGKA   1.000
0   Bm86_650_bp   0.485434   2.36   NA   0.333553   24.29
1353.99   <= WB
 613   BoLA-DRB3_01201   TTKAKDKDPDPEKSS   5   DKDPDPEKS   0.250
0   Bm86_650_bp   0.421815   2.92   NA   0.068648   96.91
23790.04   <= WB
 458   BoLA-DRB3_01201   VDNLFWFQCADGYTT   4   FWFQCADGY   0.880
0   Bm86_650_bp   0.271611   4.89   NA   0.391321   10.19
724.71   <= WB
 336   BoLA-DRB3_01201   LNEYYYTVSFTPNIS   5   YTVSFTPNI   0.830
0   Bm86_650_bp   0.261615   5.07   NA   0.481076   1.49
274.42   <= WB
   2   BoLA-DRB3_01201   RGIALFVAAVSLIVE   5   FVAAVSLIV   0.910
0   Bm86_650_bp   0.207518   6.27   NA   0.281721   42.91
2372.31   <= WB

```
 346   BoLA-DRB3_01201       TPNISFDSDHCKRYE    2   NISFDSDHC     0.700
0     Bm86_650_bp      0.203552     6.37        NA      0.331504    24.93
1384.34   <= WB
 636   BoLA-DRB3_01201       LLLLLAATSVTAASL    4   LAATSVTAA     0.370
0     Bm86_650_bp      0.181005     7.04        NA      0.278547    44.13
2455.20   <= WB
 341   BoLA-DRB3_01201       YTVSFTPNISFDSDH    2   VSFTPNISF     0.660
0     Bm86_650_bp      0.138673     8.69        NA      0.549844     0.20
130.40   <= WB
 377   BoLA-DRB3_01201       KVEILNCTQDIKARL    4   LNCTQDIKA     0.960
0     Bm86_650_bp      0.119397     9.68        NA      0.373775    13.59
876.22   <= WB
-----------------------------------------------------------------------------
---------------------------------------------------------------
Number of strong binders: 0 Number of weak binders: 9
-----------------------------------------------------------------------------
---------------------------------------------------------------
-----------------------------------------------------------------------------
---------------------------------------------------------------
 Pos              MHC              Peptide   Of        Core  Core_Rel
Inverted        Identity      Score_EL %Rank_EL  Exp_Bind      Score_BA
%Rank_BA  Affinity(nM)  BindLevel
-----------------------------------------------------------------------------
---------------------------------------------------------------
 140   BoLA-DRB3_01201       VSTLTCFIDQVIATG    3   LTCFIDQVI     1.000
0     AQP1_299_bp      0.792341     0.63        NA      0.399111     8.83
666.13   <= SB
 285   BoLA-DRB3_01201       DKMVLELEPTQHQRL    4   LELEPTQHQ     0.540
0     AQP1_299_bp      0.736478     0.84        NA      0.337252    23.14
1300.87   <= SB
  74   BoLA-DRB3_01201       AQASVRKFPIAKVPL    4   VRKFPIAKV     1.000
0     AQP1_299_bp      0.736452     0.84        NA      0.552971     0.18
126.06   <= SB
 276   BoLA-DRB3_01201       VETLYKVDGDKMVLE    4   YKVDGDKMV     0.940
0     AQP1_299_bp      0.669744     1.15        NA      0.393308     9.84
709.30   <= SB
   3   BoLA-DRB3_01201       IENLLIRQLINEFLG    5   IRQLINEFL     1.000
0     AQP1_299_bp      0.608615     1.48        NA      0.519205     0.48
181.65   <= SB
 102   BoLA-DRB3_01201       ALVFATYKDAIEHFD    3   FATYKDAIE     1.000
0     AQP1_299_bp      0.589525     1.61        NA      0.470335     1.95
308.23   <= SB
 224   BoLA-DRB3_01201       LRGWNYVWVPLLGPH    3   WNYVWVPLL     0.860
0     AQP1_299_bp      0.471879     2.46        NA      0.514234     0.57
191.69   <= WB
 129   BoLA-DRB3_01201       AGIFATYPRPHVSTL    3   FATYPRPHV     1.000
0     AQP1_299_bp      0.430445     2.84        NA      0.436326     4.18
445.34   <= WB
 116   BoLA-DRB3_01201       DQGIRQVTGEKATAG    3   IRQVTGEKA     1.000
0     AQP1_299_bp      0.396486     3.19        NA      0.362163    16.30
993.52   <= WB
 244   BoLA-DRB3_01201       GVWLYKVAIGDHWPE    4   YKVAIGDHW     0.960
0     AQP1_299_bp      0.351649     3.70        NA      0.460069     2.46
344.44   <= WB
  26   BoLA-DRB3_01201       DSIMAIIIAGDNESL    5   IIIAGDNES     0.870
0     AQP1_299_bp      0.331380     3.95        NA      0.379299    12.48
825.38   <= WB
```

86 BoLA-DRB3_01201 VPLYFAAQYLGGFVG 4 FAAQYLGGF 0.710
0 AQP1_299_bp 0.209150 6.22 NA 0.419519 5.96
534.15 <= WB
59 BoLA-DRB3_01201 ISGGVSSHLNPAVTL 4 VSSHLNPAV 0.980
0 AQP1_299_bp 0.182316 7.00 NA 0.386731 11.01
761.61 <= WB
226 BoLA-DRB3_01201 GWNYVWVPLLGPHIG 5 WVPLLGPHI 0.740
0 AQP1_299_bp 0.177071 7.18 NA 0.494248 1.04
237.97 <= WB
51 BoLA-DRB3_01201 VAIYVAVQISGGVSS 3 YVAVQISGG 0.870
0 AQP1_299_bp 0.133260 8.95 NA 0.389043 10.59
742.79 <= WB

Number of strong binders: 6 Number of weak binders: 9

Pos MHC Peptide Of Core Core_Rel
Inverted Identity Score_EL %Rank_EL Exp_Bind Score_BA
%Rank_BA Affinity(nM) BindLevel

----
118 BoLA-DRB3_01201 GLVYLVYADALSQVD 3 YLVYADALS 1.000
0 AQP2_293_bp 0.876041 0.36 NA 0.434948 4.31
452.03 <= SB
145 BoLA-DRB3_01201 APVFSCFPAPGVSTL 3 FSCFPAPGV 1.000
0 AQP2_293_bp 0.739046 0.83 NA 0.449965 3.11
384.24 <= SB
259 BoLA-DRB3_01201 GVWIYKLAVDNHWKD 4 YKLAVDNHW 0.960
0 AQP2_293_bp 0.652626 1.23 NA 0.537105 0.29
149.67 <= SB
239 BoLA-DRB3_01201 FRSYNWFWVPVVGPH 3 YNWFWVPVV 0.980
0 AQP2_293_bp 0.531020 2.02 NA 0.491384 1.12
245.45 <= WB
241 BoLA-DRB3_01201 SYNWFWVPVVGPHLG 5 WVPVVGPHL 0.530
0 AQP2_293_bp 0.221164 5.93 NA 0.459068 2.51
348.20 <= WB
43 BoLA-DRB3_01201 VLASLAVFQLGSVGL 4 LAVFQLGSV 0.830
0 AQP2_293_bp 0.220170 5.95 NA 0.207823 71.22
5277.35 <= WB
135 BoLA-DRB3_01201 LAIVYGTNATAPVFS 4 YGTNATAPV 0.820
0 AQP2_293_bp 0.208221 6.25 NA 0.449280 3.16
387.09 <= WB
279 BoLA-DRB3_01201 EDEKRPLLSNAKICA 3 KRPLLSNAK 0.510
0 AQP2_293_bp 0.202407 6.40 NA 0.225279 64.97
4369.09 <= WB
156 BoLA-DRB3_01201 VSTLTCLLDQTVSTA 3 LTCLLDQTV 1.000
0 AQP2_293_bp 0.191595 6.71 NA 0.327042 26.38
1452.81 <= WB
133 BoLA-DRB3_01201 VNLAIVYGTNATAPV 4 IVYGTNATA 0.600
0 AQP2_293_bp 0.140493 8.60 NA 0.399101 8.83
666.20 <= WB

Number of strong binders: 3 Number of weak binders: 7

Pos	MHC	Peptide	Of	Core	Core_Rel	Inverted	Identity	Score_EL	%Rank_EL	Exp_Bind	Score_BA	%Rank_BA	Affinity(nM)	BindLevel
578	BoLA-DRB3_01201	DFSFVIVAEEDLVYK	3	FVIVAEEDL	0.760	0	VgR_1799_bp	0.975999	0.06	NA	0.533991	0.31	154.80	<= SB
1344	BoLA-DRB3_01201	DKSIFWTEMDEGVIN	4	FWTEMDEGV	0.950	0	VgR_1799_bp	0.913679	0.25	NA	0.488173	1.22	254.13	<= SB
491	BoLA-DRB3_01201	WSDWASYSLDSSNKR	3	WASYSLDSS	1.000	0	VgR_1799_bp	0.867930	0.38	NA	0.510065	0.66	200.53	<= SB
718	BoLA-DRB3_01201	KPTITSYTMDGQNPR	3	ITSYTMDGQ	0.970	0	VgR_1799_bp	0.860005	0.40	NA	0.439461	3.91	430.48	<= SB
478	BoLA-DRB3_01201	HPFALAVFEDTVYWS	4	LAVFEDTVY	1.000	0	VgR_1799_bp	0.851748	0.43	NA	0.463189	2.31	333.01	<= SB
1365	BoLA-DRB3_01201	GKQFTLLEDIHKPYH	3	FTLLEDIHK	1.000	0	VgR_1799_bp	0.774948	0.69	NA	0.430361	4.75	475.02	<= SB
1606	BoLA-DRB3_01201	SDERYHINSSDILGQ	4	YHINSSDIL	1.000	0	VgR_1799_bp	0.769206	0.71	NA	0.396319	9.30	686.56	<= SB
399	BoLA-DRB3_01201	PPQKVVYWTDWGSRP	4	VVYWTDWGS	0.970	0	VgR_1799_bp	0.689849	1.05	NA	0.383263	11.71	790.73	<= SB
474	BoLA-DRB3_01201	EEVFHPFALAVFEDT	3	FHPFALAVF	1.000	0	VgR_1799_bp	0.679210	1.09	NA	0.478296	1.61	282.79	<= SB
580	BoLA-DRB3_01201	SFVIVAEEDLVYKID	3	IVAEEDLVY	0.700	0	VgR_1799_bp	0.650404	1.25	NA	0.482517	1.44	270.17	<= SB
1373	BoLA-DRB3_01201	DIHKPYHIAVDWVAG	5	YHIAVDWVA	0.990	0	VgR_1799_bp	0.645958	1.27	NA	0.333597	24.28	1353.34	<= SB
1510	BoLA-DRB3_01201	SPFSIALFEDWLYWS	4	IALFEDWLY	0.990	0	VgR_1799_bp	0.633197	1.33	NA	0.359683	16.94	1020.54	<= SB
1785	BoLA-DRB3_01201	DMAAKQDKVFFFRKH	1	MAAKQDKVF	0.450	0	VgR_1799_bp	0.624824	1.38	NA	0.324571	27.19	1492.18	<= SB
663	BoLA-DRB3_01201	VTHQLLYWVDADKYT	5	LYWVDADKY	0.460	0	VgR_1799_bp	0.622067	1.40	NA	0.501074	0.86	221.02	<= SB
1428	BoLA-DRB3_01201	DGLMFWAVWHEVVQK	5	WAVWHEVVQ	0.610	0	VgR_1799_bp	0.618890	1.42	NA	0.456695	2.66	357.25	<= SB
311	BoLA-DRB3_01201	SDQHRVFWTDVSTRR	3	HRVFWTDVS	0.910	0	VgR_1799_bp	0.601746	1.53	NA	0.444408	3.50	408.05	<= SB

1743 BoLA-DRB3_01201 NPAFNTRKTELLSED 3 FNTRKTELL 1.000
0 VgR_1799_bp 0.554746 1.84 NA 0.342624 21.53
1227.41 <= SB
292 BoLA-DRB3_01201 NRYFEIHPAEAQAVG 3 FEIHPAEAQ 0.900
0 VgR_1799_bp 0.544408 1.92 NA 0.482879 1.42
269.11 <= SB
638 BoLA-DRB3_01201 AINVKSFEQWTVHDH 3 VKSFEQWTV 0.920
0 VgR_1799_bp 0.536000 1.98 NA 0.394857 9.56
697.51 <= SB
1302 BoLA-DRB3_01201 GEPFLLYMLPNQIRS 3 FLLYMLPNQ 0.680
0 VgR_1799_bp 0.534895 1.99 NA 0.602046 0.03
74.13 <= SB
1478 BoLA-DRB3_01201 KRIYWSDANKNVIES 3 YWSDANKNV 0.900
0 VgR_1799_bp 0.533142 2.00 NA 0.470255 1.96
308.50 <= WB
487 BoLA-DRB3_01201 DTVYWSDWASYSLDS 4 WSDWASYSL 0.960
0 VgR_1799_bp 0.504580 2.22 NA 0.391897 10.09
720.21 <= WB
271 BoLA-DRB3_01201 DPEPLLVFSTTKEIR 4 LLVFSTTKE 0.820
0 VgR_1799_bp 0.487729 2.34 NA 0.449837 3.12
384.77 <= WB
215 BoLA-DRB3_01201 NAGFRLLADHISCAD 3 FRLLADHIS 1.000
0 VgR_1799_bp 0.480404 2.40 NA 0.433880 4.40
457.28 <= WB
1413 BoLA-DRB3_01201 DTAYSHLNTFALSAN 3 YSHLNTFAL 1.000
0 VgR_1799_bp 0.468949 2.48 NA 0.425529 5.28
500.52 <= WB
685 BoLA-DRB3_01201 GSGHVIIRDDLHRPV 3 HVIIRDDLH 0.860
0 VgR_1799_bp 0.462013 2.54 NA 0.379796 12.38
820.96 <= WB
1729 BoLA-DRB3_01201 DEDYHAMNTTPGFIN 3 YHAMNTTPG 0.990
0 VgR_1799_bp 0.451368 2.64 NA 0.289723 39.93
2175.56 <= WB
1417 BoLA-DRB3_01201 SHLNTFALSANDGLM 5 FALSANDGL 0.990
0 VgR_1799_bp 0.450392 2.65 NA 0.539875 0.27
145.25 <= WB
1518 BoLA-DRB3_01201 EDWLYWSDWGSDSLM 2 WLYWSDWGS 0.510
0 VgR_1799_bp 0.423079 2.91 NA 0.366967 15.11
943.20 <= WB
391 BoLA-DRB3_01201 SPRAIIVNPPQKVVY 4 IIVNPPQKV 0.990
0 VgR_1799_bp 0.418537 2.95 NA 0.502655 0.82
217.27 <= WB
1245 BoLA-DRB3_01201 HPGYRLNTDRKSCDD 3 YRLNTDRKS 1.000
0 VgR_1799_bp 0.412396 3.01 NA 0.284808 41.77
2294.38 <= WB
641 BoLA-DRB3_01201 VKSFEQWTVHDHIGS 3 FEQWTVHDH 0.970
0 VgR_1799_bp 0.384938 3.32 NA 0.342755 21.49
1225.67 <= WB
730 BoLA-DRB3_01201 NPRVLPLSTLLLPVS 3 VLPLSTLLL 1.000
0 VgR_1799_bp 0.371869 3.46 NA 0.465120 2.21
326.13 <= WB
775 BoLA-DRB3_01201 GTPFIVQQVKAHISS 3 FIVQQVKAH 0.700
0 VgR_1799_bp 0.333090 3.92 NA 0.442614 3.65
416.04 <= WB
1286 BoLA-DRB3_01201 DGYALGADRRYCKVQ 2 YALGADRRY 1.000
0 VgR_1799_bp 0.310762 4.25 NA 0.310535 32.05
1736.90 <= WB

834 BoLA-DRB3_01201 RRVIVAAQVPPFAPG 3 IVAAQVPPF 0.990
0 VgR_1799_bp 0.309847 4.26 NA 0.472735 1.85
300.33 <= WB
1454 BoLA-DRB3_01201 GTARVVLLTDKILWP 4 VVLLTDKIL 0.980
0 VgR_1799_bp 0.254188 5.22 NA 0.372126 13.95
891.99 <= WB
116 BoLA-DRB3_01201 IPAHWRCDQTEDCAD 4 WRCDQTEDC 0.990
0 VgR_1799_bp 0.243132 5.44 NA 0.194004 75.89
6128.46 <= WB
294 BoLA-DRB3_01201 YFEIHPAEAQAVGVE 3 IHPAEAQAV 0.620
0 VgR_1799_bp 0.238981 5.53 NA 0.419695 5.94
533.13 <= WB
1756 BoLA-DRB3_01201 EDGELKRWASSDSLQ 4 LKRWASSDS 0.940
0 VgR_1799_bp 0.231259 5.70 NA 0.305424 33.94
1835.66 <= WB
1310 BoLA-DRB3_01201 LPNQIRSFSMHGHAQ 4 IRSFSMHGH 0.990
0 VgR_1799_bp 0.221472 5.92 NA 0.449568 3.14
385.89 <= WB
1722 BoLA-DRB3_01201 DEHPIAADEDYHAMN 4 IAADEDYHA 0.990
0 VgR_1799_bp 0.215538 6.06 NA 0.277051 44.72
2495.26 <= WB
1051 BoLA-DRB3_01201 EDDFHCANGQCVDKR 3 FHCANGQCV 1.000
0 VgR_1799_bp 0.214450 6.09 NA 0.276594 44.90
2507.63 <= WB
455 BoLA-DRB3_01201 LSSLEYLELSTLKRD 3 LEYLELSTL 0.530
0 VgR_1799_bp 0.210963 6.18 NA 0.387076 10.95
758.77 <= WB
587 BoLA-DRB3_01201 EDLVYKIDLNKVGAP 4 YKIDLNKVG 0.760
0 VgR_1799_bp 0.210681 6.19 NA 0.408621 7.40
601.00 <= WB
662 BoLA-DRB3_01201 DVTHQLLYWVDADKY 5 LLYWVDADK 0.700
0 VgR_1799_bp 0.204628 6.34 NA 0.448210 3.23
391.60 <= WB
467 BoLA-DRB3_01201 KRDVVMHEEVFHPFA 3 VVMHEEVFH 0.770
0 VgR_1799_bp 0.196180 6.57 NA 0.357298 17.52
1047.22 <= WB
256 BoLA-DRB3_01201 LEGYQLTDNSFCKAR 3 YQLTDNSFC 0.950
0 VgR_1799_bp 0.177937 7.15 NA 0.389884 10.45
736.07 <= WB
1689 BoLA-DRB3_01201 YVLYRRNRDKLAALD 3 YRRNRDKLA 1.000
0 VgR_1799_bp 0.169911 7.42 NA 0.426183 5.21
496.99 <= WB
760 BoLA-DRB3_01201 VRGTIESLDLQKVFT 4 IESLDLQKV 1.000
0 VgR_1799_bp 0.169819 7.42 NA 0.373475 13.65
879.07 <= WB
1427 BoLA-DRB3_01201 NDGLMFWAVWHEVVQ 5 FWAVWHEVV 0.520
0 VgR_1799_bp 0.168191 7.47 NA 0.393101 9.87
710.89 <= WB
658 BoLA-DRB3_01201 GIDFDVTHQLLYWVD 3 FDVTHQLLY 0.820
0 VgR_1799_bp 0.150770 8.15 NA 0.388353 10.72
748.36 <= WB
351 BoLA-DRB3_01201 DLSLDWVANNLYITD 3 LDWVANNLY 0.860
0 VgR_1799_bp 0.145332 8.38 NA 0.378068 12.71
836.45 <= WB
1385 BoLA-DRB3_01201 VAGNIYFTDGWVHIQ 3 NIYFTDGWV 0.800
0 VgR_1799_bp 0.136147 8.81 NA 0.459778 2.47
345.53 <= WB

356   BoLA-DRB3_01201   WVANNLYITDSLVKR   3   NNLYITDSL   0.610
0   VgR_1799_bp   0.133737   8.92   NA   0.435727   4.24
448.23   <= WB
1277   BoLA-DRB3_01201   KGSYKCTCADGYALG   3   YKCTCADGY   1.000
0   VgR_1799_bp   0.133511   8.93   NA   0.313357   30.99
1684.67   <= WB
821   BoLA-DRB3_01201   HVSLRSTRNGTFSRR   3   LRSTRNGTF   1.000
0   VgR_1799_bp   0.133510   8.93   NA   0.392559   9.97
715.07   <= WB
247   BoLA-DRB3_01201   SPGSYSCHCLEGYQL   4   YSCHCLEGY   1.000
0   VgR_1799_bp   0.128694   9.18   NA   0.335667   23.63
1323.37   <= WB
1519   BoLA-DRB3_01201   DWLYWSDWGSDSLMA   4   WSDWGSDSL   0.880
0   VgR_1799_bp   0.114043   9.98   NA   0.250568   55.20
3323.21   <= WB

Number of strong binders: 20 Number of weak binders: 39

BoLA-DRB3_01501 : Distance to training data 0.000 (using nearest neighbor BoLA-DRB3_01501)

Allele: BoLA-DRB3_01501

Pos   MHC   Peptide   Of   Core Core_Rel
Inverted   Identity   Score_EL %Rank_EL Exp_Bind   Score_BA
%Rank_BA Affinity(nM) BindLevel

----
360   BoLA-DRB3_01501   EDRVLEAIRTSIGKE   3   VLEAIRTSI   1.000
0   Bm86_650_bp   0.976904   0.08   NA   0.636594   0.87
51.01   <= SB
2   BoLA-DRB3_01501   RGIALFVAAVSLIVE   4   LFVAAVSLI   0.630
0   Bm86_650_bp   0.626670   1.75   NA   0.532999   6.72
156.47   <= SB
363   BoLA-DRB3_01501   VLEAIRTSIGKEVFK   4   IRTSIGKEV   0.990
0   Bm86_650_bp   0.518434   2.67   NA   0.578091   3.14
96.06   <= WB
419   BoLA-DRB3_01501   YPKLLIKKNSATEIE   5   IKKNSATEI   0.880
0   Bm86_650_bp   0.482582   3.03   NA   0.607051   1.74
70.22   <= WB
341   BoLA-DRB3_01501   YTVSFTPNISFDSDH   2   VSFTPNISF   0.970
0   Bm86_650_bp   0.372007   4.43   NA   0.558452   4.49
118.80   <= WB
496   BoLA-DRB3_01501   EQLECANKGQICVYE   2   LECANKGQI   1.000
0   Bm86_650_bp   0.350032   4.82   NA   0.355042   42.94
1073.10   <= WB
636   BoLA-DRB3_01501   LLLLLAATSVTAASL   4   LAATSVTAA   0.590
0   Bm86_650_bp   0.326735   5.26   NA   0.544076   5.66
138.79   <= WB
613   BoLA-DRB3_01501   TTKAKDKDPDPEKSS   3   AKDKDPDPE   0.310
0   Bm86_650_bp   0.254493   6.87   NA   0.061771   97.59
25627.72   <= WB

```
  98   BoLA-DRB3_01501       DLTLQCKIKNDFATD    3   LQCKIKNDF     0.990
0     Bm86_650_bp      0.221483     7.95         NA      0.339539    47.25
1269.07   <= WB
 628   BoLA-DRB3_01501       AAAVSATGLLLLLAA    3   VSATGLLLL     0.950
0     Bm86_650_bp      0.206885     8.47         NA      0.586853     2.64
87.37   <= WB
 389   BoLA-DRB3_01501       ARLIAEKPLSKYVLR    3   IAEKPLSKY     0.930
0     Bm86_650_bp      0.186330     9.23         NA      0.470396    15.59
308.03   <= WB
 136   BoLA-DRB3_01501       EWGAMNKTTRNCVPT    4   MNKTTRNCV     1.000
0     Bm86_650_bp      0.176492     9.66         NA      0.418175    26.43
541.97   <= WB
-----------------------------------------------------------------------------
--------------------------------------------------------------
Number of strong binders: 2 Number of weak binders: 10
-----------------------------------------------------------------------------
--------------------------------------------------------------
-----------------------------------------------------------------------------
--------------------------------------------------------------
 Pos              MHC              Peptide   Of        Core  Core_Rel
Inverted        Identity      Score_EL %Rank_EL  Exp_Bind      Score_BA
%Rank_BA  Affinity(nM)  BindLevel
-----------------------------------------------------------------------------
--------------------------------------------------------------
 285   BoLA-DRB3_01501       DKMVLELEPTQHQRL    6   LEPTQHQRL     0.970
0     AQP1_299_bp      0.920393     0.25         NA      0.450890    19.28
380.41   <= SB
 100   BoLA-DRB3_01501       GAALVFATYKDAIEH    4   VFATYKDAI     1.000
0     AQP1_299_bp      0.877171     0.39         NA      0.644843     0.71
46.65   <= SB
  74   BoLA-DRB3_01501       AQASVRKFPIAKVPL    4   VRKFPIAKV     0.980
0     AQP1_299_bp      0.626276     1.75         NA      0.650953     0.59
43.67   <= SB
  68   BoLA-DRB3_01501       NPAVTLAQASVRKFP    5   LAQASVRKF     1.000
0     AQP1_299_bp      0.560437     2.29         NA      0.584495     2.77
89.63   <= WB
   3   BoLA-DRB3_01501       IENLLIRQLINEFLG    4   LIRQLINEF     0.520
0     AQP1_299_bp      0.352434     4.78         NA      0.525917     7.48
168.93   <= WB
  86   BoLA-DRB3_01501       VPLYFAAQYLGGFVG    4   FAAQYLGGF     0.720
0     AQP1_299_bp      0.350996     4.80         NA      0.519980     8.14
180.14   <= WB
 147   BoLA-DRB3_01501       IDQVIATGIMMVCVE    3   VIATGIMMV     0.960
0     AQP1_299_bp      0.311122     5.57         NA      0.576829     3.21
97.38   <= WB
 128   BoLA-DRB3_01501       TAGIFATYPRPHVST    4   FATYPRPHV     0.910
0     AQP1_299_bp      0.277142     6.31         NA      0.466625    16.27
320.86   <= WB
 152   BoLA-DRB3_01501       ATGIMMVCVEAIGDT    3   IMMVCVEAI     0.980
0     AQP1_299_bp      0.269504     6.50         NA      0.587970     2.58
86.32   <= WB
 116   BoLA-DRB3_01501       DQGIRQVTGEKATAG    3   IRQVTGEKA     0.990
0     AQP1_299_bp      0.258797     6.76         NA      0.407289    29.02
609.72   <= WB
 224   BoLA-DRB3_01501       LRGWNYVWVPLLGPH    3   WNYVWVPLL     0.880
0     AQP1_299_bp      0.250077     6.98         NA      0.539645     6.07
145.61   <= WB
```

16 BoLA-DRB3_01501 LGTMILITIGDSIMA 4 ILITIGDSI 0.840
0 AQP1_299_bp 0.206641 8.48 NA 0.517218 8.47
185.60 <= WB
26 BoLA-DRB3_01501 DSIMAIIIAGDNESL 6 IIAGDNESL 0.600
0 AQP1_299_bp 0.198069 8.78 NA 0.462947 16.94
333.88 <= WB
44 BoLA-DRB3_01501 VGPLGWGVAIYVAVQ 3 LGWGVAIYV 0.660
0 AQP1_299_bp 0.191805 8.99 NA 0.506431 9.87
208.58 <= WB
56 BoLA-DRB3_01501 AVQISGGVSSHLNPA 3 ISGGVSSHL 0.970
0 AQP1_299_bp 0.180354 9.48 NA 0.534164 6.61
154.51 <= WB

Number of strong binders: 3 Number of weak binders: 12

Pos MHC Peptide Of Core Core_Rel
Inverted Identity Score_EL %Rank_EL Exp_Bind Score_BA
%Rank_BA Affinity(nM) BindLevel

----

84 BoLA-DRB3_01501 NPAVTVALATIGKLG 5 VALATIGKL 1.000
0 AQP2_293_bp 0.676230 1.41 NA 0.590592 2.44
83.91 <= SB
80 BoLA-DRB3_01501 GAHLNPAVTVALATI 3 LNPAVTVAL 1.000
0 AQP2_293_bp 0.619313 1.80 NA 0.589012 2.52
85.35 <= SB
27 BoLA-DRB3_01501 LAEMVGTLVLTLVGD 3 MVGTLVLTL 0.980
0 AQP2_293_bp 0.446556 3.41 NA 0.559313 4.42
117.70 <= WB
89 BoLA-DRB3_01501 VALATIGKLGWCNVL 2 LATIGKLGW 0.990
0 AQP2_293_bp 0.332491 5.15 NA 0.614027 1.51
65.11 <= WB
279 BoLA-DRB3_01501 EDEKRPLLSNAKICA 6 LLSNAKICA 0.530
0 AQP2_293_bp 0.286635 6.09 NA 0.402474 30.19
642.33 <= WB
43 BoLA-DRB3_01501 VLASLAVFQLGSVGL 4 LAVFQLGSV 0.800
0 AQP2_293_bp 0.238625 7.35 NA 0.411714 27.98
581.22 <= WB
18 BoLA-DRB3_01501 IENTLARQALAEMVG 4 LARQALAEM 0.770
0 AQP2_293_bp 0.198208 8.77 NA 0.470896 15.51
306.37 <= WB
74 BoLA-DRB3_01501 VAGGVSGAHLNPAVT 4 VSGAHLNPA 0.990
0 AQP2_293_bp 0.183858 9.33 NA 0.425770 24.62
499.22 <= WB
273 BoLA-DRB3_01501 DEDEVDEDEKRPLLS 4 VDEDEKRPL 0.990
0 AQP2_293_bp 0.177676 9.60 NA 0.205934 82.25
5386.32 <= WB

Number of strong binders: 2 Number of weak binders: 7

Pos	MHC	Peptide	Of	Core	Core_Rel	Inverted	Identity	Score_EL	%Rank_EL	Exp_Bind	Score_BA	%Rank_BA	Affinity(nM)	BindLevel
337	BoLA-DRB3_01501	DFKTLFSAEKTLLED	4	LFSAEKTLL	1.000	0	VgR_1799_bp	0.969809	0.10	NA	0.587132	2.62	87.11	<= SB
775	BoLA-DRB3_01501	GTPFIVQQVKAHISS	4	IVQQVKAHI	1.000	0	VgR_1799_bp	0.967936	0.11	NA	0.642768	0.75	47.71	<= SB
271	BoLA-DRB3_01501	DPEPLLVFSTTKEIR	5	LVFSTTKEI	0.960	0	VgR_1799_bp	0.917293	0.26	NA	0.639975	0.80	49.18	<= SB
834	BoLA-DRB3_01501	RRVIVAAQVPPFAPG	3	IVAAQVPPF	1.000	0	VgR_1799_bp	0.906809	0.29	NA	0.671929	0.31	34.80	<= SB
294	BoLA-DRB3_01501	YFEIHPAEAQAVGVE	3	IHPAEAQAV	1.000	0	VgR_1799_bp	0.797911	0.73	NA	0.588518	2.55	85.81	<= SB
391	BoLA-DRB3_01501	SPRAIIVNPPQKVVY	4	IIVNPPQKV	0.930	0	VgR_1799_bp	0.790526	0.76	NA	0.670634	0.32	35.29	<= SB
578	BoLA-DRB3_01501	DFSFVIVAEEDLVYK	4	VIVAEEDLV	0.420	0	VgR_1799_bp	0.774736	0.84	NA	0.518402	8.33	183.24	<= SB
1785	BoLA-DRB3_01501	DMAAKQDKVFFFRKH	1	MAAKQDKVF	0.430	0	VgR_1799_bp	0.714269	1.17	NA	0.440804	21.39	424.27	<= SB
862	BoLA-DRB3_01501	LPVRTTDRSCFCPPG	2	VRTTDRSCF	1.000	0	VgR_1799_bp	0.708592	1.21	NA	0.551511	5.04	128.07	<= SB
794	BoLA-DRB3_01501	HNEIHWTSRDKASLE	3	IHWTSRDKA	0.970	0	VgR_1799_bp	0.686582	1.35	NA	0.487375	12.74	256.34	<= SB
1337	BoLA-DRB3_01501	GMDYRVTDKSIFWTE	3	YRVTDKSIF	1.000	0	VgR_1799_bp	0.673880	1.43	NA	0.475125	14.77	292.67	<= SB
1449	BoLA-DRB3_01501	RSNMDGTARVVLLTD	3	MDGTARVVL	1.000	0	VgR_1799_bp	0.669602	1.45	NA	0.488253	12.60	253.91	<= SB
745	BoLA-DRB3_01501	ISVDLVARTLVWADA	2	VDLVARTLV	1.000	0	VgR_1799_bp	0.505370	2.80	NA	0.571160	3.57	103.54	<= WB
1470	BoLA-DRB3_01501	SITVDAVHKRIYWSD	3	VDAVHKRIY	0.990	0	VgR_1799_bp	0.503650	2.82	NA	0.517213	8.47	185.61	<= WB
474	BoLA-DRB3_01501	EEVFHPFALAVFEDT	3	FHPFALAVF	1.000	0	VgR_1799_bp	0.485083	3.00	NA	0.564590	4.01	111.17	<= WB
1478	BoLA-DRB3_01501	KRIYWSDANKNVIES	4	WSDANKNVI	0.890	0	VgR_1799_bp	0.471087	3.15	NA	0.458763	17.74	349.35	<= WB

658 BoLA-DRB3_01501 GIDFDVTHQLLYWVD 3 FDVTHQLLY 0.880
0 VgR_1799_bp 0.469698 3.17 NA 0.552691 4.95
126.44 <= WB
587 BoLA-DRB3_01501 EDLVYKIDLNKVGAP 3 VYKIDLNKV 0.990
0 VgR_1799_bp 0.464507 3.22 NA 0.490854 12.18
246.87 <= WB
1428 BoLA-DRB3_01501 DGLMFWAVWHEVVQK 4 FWAVWHEVV 0.770
0 VgR_1799_bp 0.444111 3.44 NA 0.547461 5.37
133.80 <= WB
821 BoLA-DRB3_01501 HVSLRSTRNGTFSRR 3 LRSTRNGTF 1.000
0 VgR_1799_bp 0.437074 3.52 NA 0.518147 8.36
183.74 <= WB
1455 BoLA-DRB3_01501 TARVVLLTDKILWPC 4 VLLTDKILW 0.920
0 VgR_1799_bp 0.427075 3.64 NA 0.565343 3.96
110.27 <= WB
803 BoLA-DRB3_01501 DKASLEYIDRSSEPS 4 LEYIDRSSE 1.000
0 VgR_1799_bp 0.404095 3.94 NA 0.333162 49.06
1359.73 <= WB
455 BoLA-DRB3_01501 LSSLEYLELSTLKRD 3 LEYLELSTL 0.980
0 VgR_1799_bp 0.395311 4.06 NA 0.477150 14.44
286.32 <= WB
1434 BoLA-DRB3_01501 AVWHEVVQKDHGLIE 5 VVQKDHGLI 0.990
0 VgR_1799_bp 0.387501 4.18 NA 0.474100 14.94
295.93 <= WB
730 BoLA-DRB3_01501 NPRVLPLSTLLLPVS 3 VLPLSTLLL 0.890
0 VgR_1799_bp 0.386971 4.19 NA 0.610658 1.61
67.53 <= WB
1599 BoLA-DRB3_01501 DAHKCVESDERYHIN 5 VESDERYHI 1.000
0 VgR_1799_bp 0.383534 4.24 NA 0.329234 50.19
1418.76 <= WB
638 BoLA-DRB3_01501 AINVKSFEQWTVHDH 3 VKSFEQWTV 0.920
0 VgR_1799_bp 0.382195 4.27 NA 0.496308 11.32
232.72 <= WB
580 BoLA-DRB3_01501 SFVIVAEEDLVYKID 3 IVAEEDLVY 0.580
0 VgR_1799_bp 0.379046 4.32 NA 0.504704 10.11
212.51 <= WB
665 BoLA-DRB3_01501 HQLLYWVDADKYTLE 5 WVDADKYTL 0.560
0 VgR_1799_bp 0.369676 4.46 NA 0.461610 17.19
338.75 <= WB
686 BoLA-DRB3_01501 SGHVIIRDDLHRPVG 5 IRDDLHRPV 0.770
0 VgR_1799_bp 0.365311 4.54 NA 0.473389 15.06
298.21 <= WB
663 BoLA-DRB3_01501 VTHQLLYWVDADKYT 5 LYWVDADKY 0.930
0 VgR_1799_bp 0.318627 5.42 NA 0.458250 17.83
351.29 <= WB
752 BoLA-DRB3_01501 RTLVWADAVRGTIES 4 WADAVRGTI 0.880
0 VgR_1799_bp 0.293780 5.93 NA 0.521466 7.97
177.26 <= WB
1302 BoLA-DRB3_01501 GEPFLLYMLPNQIRS 4 LLYMLPNQI 0.950
0 VgR_1799_bp 0.291204 5.98 NA 0.560957 4.30
115.62 <= WB
1365 BoLA-DRB3_01501 GKQFTLLEDIHKPYH 5 LLEDIHKPY 0.840
0 VgR_1799_bp 0.288167 6.05 NA 0.460212 17.46
343.91 <= WB
606 BoLA-DRB3_01501 LPVHNLGIISALTFD 5 LGIISALTF 0.960
0 VgR_1799_bp 0.284191 6.15 NA 0.556976 4.61
120.71 <= WB

1743   BoLA-DRB3_01501   NPAFNTRKTELLSED   3   FNTRKTELL   0.820
0   VgR_1799_bp   0.283360   6.17   NA   0.445063   20.48
405.17   <= WB
709   BoLA-DRB3_01501   VLFVLSAGDKPTITS   4   LSAGDKPTI   1.000
0   VgR_1799_bp   0.273175   6.41   NA   0.495476   11.45
234.82   <= WB
632   BoLA-DRB3_01501   RHSVLSAINVKSFEQ   4   LSAINVKSF   0.880
0   VgR_1799_bp   0.270197   6.48   NA   0.517678   8.42
184.68   <= WB
760   BoLA-DRB3_01501   VRGTIESLDLQKVFT   4   IESLDLQKV   1.000
0   VgR_1799_bp   0.266176   6.58   NA   0.446492   20.18
398.95   <= WB
358   BoLA-DRB3_01501   ANNLYITDSLVKRIL   5   ITDSLVKRI   0.940
0   VgR_1799_bp   0.265128   6.61   NA   0.571248   3.56
103.44   <= WB
1701   BoLA-DRB3_01501   ALDFSVSFKKPTFKK   3   FSVSFKKPT   0.740
0   VgR_1799_bp   0.256030   6.83   NA   0.489507   12.39
250.49   <= WB
1554   BoLA-DRB3_01501   VLKVLHAVHQPSGVN   3   VLHAVHQPS   0.790
0   VgR_1799_bp   0.246152   7.11   NA   0.508552   9.57
203.84   <= WB
394   BoLA-DRB3_01501   AIIVNPPQKVVYWTD   3   VNPPQKVVY   0.620
0   VgR_1799_bp   0.245454   7.13   NA   0.590886   2.43
83.64   <= WB
718   BoLA-DRB3_01501   KPTITSYTMDGQNPR   3   ITSYTMDGQ   0.950
0   VgR_1799_bp   0.232823   7.55   NA   0.320175   52.78
1564.87   <= WB
1316   BoLA-DRB3_01501   SFSMHGHAQHLLAED   3   MHGHAQHLL   1.000
0   VgR_1799_bp   0.228455   7.70   NA   0.424197   24.97
507.79   <= WB
807   BoLA-DRB3_01501   LEYIDRSSEPSVHRH   3   IDRSSEPSV   0.990
0   VgR_1799_bp   0.222962   7.90   NA   0.371269   38.46
900.30   <= WB
467   BoLA-DRB3_01501   KRDVVMHEEVFHPFA   3   VVMHEEVFH   0.700
0   VgR_1799_bp   0.221058   7.97   NA   0.426006   24.56
497.94   <= WB
1344   BoLA-DRB3_01501   DKSIFWTEMDEGVIN   3   IFWTEMDEG   0.770
0   VgR_1799_bp   0.190733   9.04   NA   0.394314   32.25
701.62   <= WB
575   BoLA-DRB3_01501   VTKDFSFVIVAEEDL   6   FVIVAEEDL   0.850
0   VgR_1799_bp   0.186045   9.24   NA   0.447111   20.05
396.29   <= WB
306   BoLA-DRB3_01501   GVEFDSDQHRVFWTD   3   FDSDQHRVF   0.980
0   VgR_1799_bp   0.178808   9.55   NA   0.332861   49.15
1364.16   <= WB

Number of strong binders: 12 Number of weak binders: 38

BoLA-DRB3_01601 : Distance to training data 0.000 (using nearest neighbor BoLA-DRB3_01601)

Allele: BoLA-DRB3_01601

Pos	MHC	Peptide	Of	Core	Core_Rel	Inverted	Identity	Score_EL	%Rank_EL	Exp_Bind	Score_BA	%Rank_BA	Affinity(nM)	BindLevel
377	BoLA-DRB3_01601	KVEILNCTQDIKARL	4	LNCTQDIKA	1.000	0	Bm86_650_bp	0.411484	2.60	NA	0.387494	8.61	755.35	<= WB
2	BoLA-DRB3_01601	RGIALFVAAVSLIVE	5	FVAAVSLIV	1.000	0	Bm86_650_bp	0.386997	2.84	NA	0.256767	43.68	3107.63	<= WB
54	BoLA-DRB3_01601	DNMYFNAAEKQCEYK	3	YFNAAEKQC	0.940	0	Bm86_650_bp	0.379264	2.92	NA	0.286275	33.15	2258.25	<= WB
336	BoLA-DRB3_01601	LNEYYYTVSFTPNIS	5	YTVSFTPNI	0.710	0	Bm86_650_bp	0.370270	3.01	NA	0.478591	1.38	281.89	<= WB
613	BoLA-DRB3_01601	TTKAKDKDPDPEKSS	4	KDKDPDPEK	0.360	0	Bm86_650_bp	0.318346	3.67	NA	0.085432	95.40	19839.36	<= WB
346	BoLA-DRB3_01601	TPNISFDSDHCKRYE	5	FDSDHCKRY	0.760	0	Bm86_650_bp	0.305874	3.84	NA	0.369333	11.52	919.36	<= WB
341	BoLA-DRB3_01601	YTVSFTPNISFDSDH	4	FTPNISFDS	0.540	0	Bm86_650_bp	0.290932	4.06	NA	0.526912	0.41	167.12	<= WB
419	BoLA-DRB3_01601	YPKLLIKKNSATEIE	5	IKKNSATEI	0.590	0	Bm86_650_bp	0.228539	5.20	NA	0.498631	0.84	226.94	<= WB
248	BoLA-DRB3_01601	SISYTVSCTVEQKQT	3	YTVSCTVEQ	0.990	0	Bm86_650_bp	0.226122	5.26	NA	0.410543	5.65	588.63	<= WB
458	BoLA-DRB3_01601	VDNLFWFQCADGYTT	5	WFQCADGYT	0.590	0	Bm86_650_bp	0.166421	6.95	NA	0.386501	8.75	763.51	<= WB
636	BoLA-DRB3_01601	LLLLLAATSVTAASL	4	LAATSVTAA	0.340	0	Bm86_650_bp	0.145492	7.77	NA	0.259562	42.68	3015.06	<= WB
501	BoLA-DRB3_01601	ANKGQICVYENGKAN	5	ICVYENGKA	0.990	0	Bm86_650_bp	0.129581	8.52	NA	0.297124	29.52	2008.14	<= WB
340	BoLA-DRB3_01601	YYTVSFTPNISFDSD	3	VSFTPNISF	0.760	0	Bm86_650_bp	0.109465	9.66	NA	0.419143	4.81	536.33	<= WB
28	BoLA-DRB3_01601	GNEFCRNAECEVVPG	3	FCRNAECEV	0.980	0	Bm86_650_bp	0.105611	9.90	NA	0.267737	39.76	2759.83	<= WB

Number of strong binders: 0 Number of weak binders: 14

Pos	MHC	Peptide	Of	Core	Core_Rel	Inverted	Identity	Score_EL	%Rank_EL	Exp_Bind	Score_BA	%Rank_BA	Affinity(nM)	BindLevel

----

276 BoLA-DRB3_01601 VETLYKVDGDKMVLE 4 YKVDGDKMV 1.000
0 AQP1_299_bp 0.983476 0.02 NA 0.484897 1.19
263.30 <= SB
285 BoLA-DRB3_01601 DKMVLELEPTQHQRL 4 LELEPTQHQ 0.560
0 AQP1_299_bp 0.785516 0.48 NA 0.379248 9.84
825.84 <= SB
284 BoLA-DRB3_01601 GDKMVLELEPTQHQR 3 MVLELEPTQ 0.510
0 AQP1_299_bp 0.632313 1.12 NA 0.381806 9.46
803.29 <= SB
224 BoLA-DRB3_01601 LRGWNYVWVPLLGPH 3 WNYVWVPLL 0.890
0 AQP1_299_bp 0.443026 2.32 NA 0.479886 1.34
277.97 <= WB
116 BoLA-DRB3_01601 DQGIRQVTGEKATAG 3 IRQVTGEKA 1.000
0 AQP1_299_bp 0.408904 2.63 NA 0.364348 12.44
970.31 <= WB
28 BoLA-DRB3_01601 IMAIIIAGDNESLAA 3 IIIAGDNES 0.870
0 AQP1_299_bp 0.400450 2.71 NA 0.362042 12.89
994.82 <= WB
86 BoLA-DRB3_01601 VPLYFAAQYLGGFVG 4 FAAQYLGGF 0.580
0 AQP1_299_bp 0.268245 4.41 NA 0.397301 7.23
679.30 <= WB
3 BoLA-DRB3_01601 IENLLIRQLINEFLG 5 IRQLINEFL 0.930
0 AQP1_299_bp 0.235245 5.05 NA 0.381879 9.44
802.66 <= WB
74 BoLA-DRB3_01601 AQASVRKFPIAKVPL 4 VRKFPIAKV 1.000
0 AQP1_299_bp 0.205359 5.79 NA 0.443030 3.01
414.18 <= WB
51 BoLA-DRB3_01601 VAIYVAVQISGGVSS 3 YVAVQISGG 0.910
0 AQP1_299_bp 0.180901 6.49 NA 0.341307 17.29
1245.03 <= WB
226 BoLA-DRB3_01601 GWNYVWVPLLGPHIG 5 WVPLLGPHI 0.630
0 AQP1_299_bp 0.160647 7.16 NA 0.470805 1.65
306.67 <= WB
140 BoLA-DRB3_01601 VSTLTCFIDQVIATG 3 LTCFIDQVI 0.980
0 AQP1_299_bp 0.144165 7.82 NA 0.268944 39.34
2724.02 <= WB
102 BoLA-DRB3_01601 ALVFATYKDAIEHFD 3 FATYKDAIE 0.980
0 AQP1_299_bp 0.128920 8.55 NA 0.391108 8.09
726.38 <= WB

Number of strong binders: 3 Number of weak binders: 10

Pos MHC Peptide Of Core Core_Rel
Inverted Identity Score_EL %Rank_EL Exp_Bind Score_BA
%Rank_BA Affinity(nM) BindLevel

----

258 BoLA-DRB3_01601 IGVWIYKLAVDNHWK 5 YKLAVDNHW 0.980
0 AQP2_293_bp 0.762512 0.57 NA 0.564081 0.14
111.78 <= SB

```
 135   BoLA-DRB3_01601       LAIVYGTNATAPVFS    4   YGTNATAPV     0.820
0     AQP2_293_bp      0.516751     1.75         NA      0.451320     2.57
378.64   <= SB
 116   BoLA-DRB3_01601       ASGLVYLVYADALSQ    5   YLVYADALS     0.960
0     AQP2_293_bp      0.353109     3.22         NA      0.342430    17.02
1229.99   <= WB
 145   BoLA-DRB3_01601       APVFSCFPAPGVSTL    3   FSCFPAPGV     1.000
0     AQP2_293_bp      0.307296     3.82         NA      0.366618    12.00
946.77   <= WB
 133   BoLA-DRB3_01601       VNLAIVYGTNATAPV    4   IVYGTNATA     0.850
0     AQP2_293_bp      0.269270     4.39         NA      0.398849     7.02
668.02   <= WB
 279   BoLA-DRB3_01601       EDEKRPLLSNAKICA    3   KRPLLSNAK     0.380
0     AQP2_293_bp      0.199242     5.96         NA      0.278721    35.77
2450.58   <= WB
 241   BoLA-DRB3_01601       SYNWFWVPVVGPHLG    5   WVPVVGPHL     0.910
0     AQP2_293_bp      0.181773     6.46         NA      0.420314     4.70
529.57   <= WB
 156   BoLA-DRB3_01601       VSTLTCLLDQTVSTA    3   LTCLLDQTV     1.000
0     AQP2_293_bp      0.162738     7.08         NA      0.307132    26.37
1802.05   <= WB
  80   BoLA-DRB3_01601       GAHLNPAVTVALATI    3   LNPAVTVAL     1.000
0     AQP2_293_bp      0.147206     7.70         NA      0.344137    16.65
1207.48   <= WB
  84   BoLA-DRB3_01601       NPAVTVALATIGKLG    5   VALATIGKL     0.880
0     AQP2_293_bp      0.144502     7.81         NA      0.373018    10.87
883.42   <= WB
 212   BoLA-DRB3_01601       GNPLNPARDLAPRIF    3   LNPARDLAP     0.980
0     AQP2_293_bp      0.139549     8.02         NA      0.328471    20.42
1430.52   <= WB
 239   BoLA-DRB3_01601       FRSYNWFWVPVVGPH    3   YNWFWVPVV     0.770
0     AQP2_293_bp      0.111295     9.55         NA      0.367056    11.93
942.29   <= WB
-----------------------------------------------------------------------------
--------------------------------------------------------------
Number of strong binders: 2 Number of weak binders: 10
-----------------------------------------------------------------------------
--------------------------------------------------------------
-----------------------------------------------------------------------------
--------------------------------------------------------------
 Pos                MHC              Peptide   Of        Core  Core_Rel
Inverted        Identity      Score_EL %Rank_EL  Exp_Bind      Score_BA
%Rank_BA  Affinity(nM)  BindLevel
-----------------------------------------------------------------------------
--------------------------------------------------------------
 578   BoLA-DRB3_01601       DFSFVIVAEEDLVYK    3   FVIVAEEDL     0.550
0     VgR_1799_bp      0.953917     0.07         NA      0.447792     2.76
393.38   <= SB
1374   BoLA-DRB3_01601       IHKPYHIAVDWVAGN    4   YHIAVDWVA     1.000
0     VgR_1799_bp      0.912056     0.15         NA      0.428596     4.00
484.18   <= SB
1365   BoLA-DRB3_01601       GKQFTLLEDIHKPYH    3   FTLLEDIHK     1.000
0     VgR_1799_bp      0.870878     0.25         NA      0.493669     0.95
239.46   <= SB
1478   BoLA-DRB3_01601       KRIYWSDANKNVIES    3   YWSDANKNV     1.000
0     VgR_1799_bp      0.864757     0.26         NA      0.493650     0.95
239.51   <= SB
```

1245 BoLA-DRB3_01601 HPGYRLNTDRKSCDD 3 YRLNTDRKS 1.000
0 VgR_1799_bp 0.864461 0.26 NA 0.422975 4.45
514.54 <= SB
1344 BoLA-DRB3_01601 DKSIFWTEMDEGVIN 4 FWTEMDEGV 0.910
0 VgR_1799_bp 0.861570 0.27 NA 0.412173 5.48
578.34 <= SB
587 BoLA-DRB3_01601 EDLVYKIDLNKVGAP 4 YKIDLNKVG 1.000
0 VgR_1799_bp 0.800152 0.43 NA 0.447392 2.78
395.08 <= SB
580 BoLA-DRB3_01601 SFVIVAEEDLVYKID 3 IVAEEDLVY 0.990
0 VgR_1799_bp 0.765645 0.55 NA 0.435848 3.48
447.64 <= SB
1606 BoLA-DRB3_01601 SDERYHINSSDILGQ 4 YHINSSDIL 0.990
0 VgR_1799_bp 0.733175 0.68 NA 0.344945 16.47
1196.97 <= SB
215 BoLA-DRB3_01601 NAGFRLLADHISCAD 3 FRLLADHIS 1.000
0 VgR_1799_bp 0.704981 0.80 NA 0.502544 0.76
217.54 <= SB
391 BoLA-DRB3_01601 SPRAIIVNPPQKVVY 4 IIVNPPQKV 1.000
0 VgR_1799_bp 0.644550 1.06 NA 0.496801 0.88
231.48 <= SB
665 BoLA-DRB3_01601 HQLLYWVDADKYTLE 4 YWVDADKYT 0.960
0 VgR_1799_bp 0.600560 1.26 NA 0.379266 9.84
825.68 <= SB
730 BoLA-DRB3_01601 NPRVLPLSTLLLPVS 3 VLPLSTLLL 1.000
0 VgR_1799_bp 0.584278 1.35 NA 0.479726 1.35
278.45 <= SB
271 BoLA-DRB3_01601 DPEPLLVFSTTKEIR 5 LVFSTTKEI 0.870
0 VgR_1799_bp 0.575820 1.40 NA 0.437081 3.40
441.71 <= SB
1413 BoLA-DRB3_01601 DTAYSHLNTFALSAN 3 YSHLNTFAL 1.000
0 VgR_1799_bp 0.572859 1.41 NA 0.433032 3.68
461.49 <= SB
1286 BoLA-DRB3_01601 DGYALGADRRYCKVQ 2 YALGADRRY 1.000
0 VgR_1799_bp 0.562621 1.47 NA 0.445609 2.87
402.78 <= SB
1689 BoLA-DRB3_01601 YVLYRRNRDKLAALD 3 YRRNRDKLA 1.000
0 VgR_1799_bp 0.532366 1.65 NA 0.538937 0.30
146.73 <= SB
256 BoLA-DRB3_01601 LEGYQLTDNSFCKAR 3 YQLTDNSFC 1.000
0 VgR_1799_bp 0.530595 1.66 NA 0.473698 1.55
297.22 <= SB
1729 BoLA-DRB3_01601 DEDYHAMNTTPGFIN 3 YHAMNTTPG 0.990
0 VgR_1799_bp 0.530053 1.66 NA 0.297330 29.46
2003.67 <= SB
821 BoLA-DRB3_01601 HVSLRSTRNGTFSRR 3 LRSTRNGTF 1.000
0 VgR_1799_bp 0.526894 1.68 NA 0.503299 0.75
215.77 <= SB
1785 BoLA-DRB3_01601 DMAAKQDKVFFFRKH 3 AKQDKVFFF 0.510
0 VgR_1799_bp 0.484448 1.96 NA 0.332922 19.29
1363.26 <= SB
339 BoLA-DRB3_01601 KTLFSAEKTLLEDLS 3 FSAEKTLLE 1.000
0 VgR_1799_bp 0.411483 2.60 NA 0.381694 9.47
804.27 <= WB
562 BoLA-DRB3_01601 IGYKLSANKHSCAVT 2 YKLSANKHS 0.990
0 VgR_1799_bp 0.404239 2.67 NA 0.510445 0.63
199.71 <= WB

775 BoLA-DRB3_01601 GTPFIVQQVKAHISS 3 FIVQQVKAH 0.810
0 VgR_1799_bp 0.378544 2.92 NA 0.431339 3.80
470.02 <= WB
116 BoLA-DRB3_01601 IPAHWRCDQTEDCAD 4 WRCDQTEDC 1.000
0 VgR_1799_bp 0.373246 2.98 NA 0.190802 68.71
6344.50 <= WB
685 BoLA-DRB3_01601 GSGHVIIRDDLHRPV 3 HVIIRDDLH 0.800
0 VgR_1799_bp 0.370610 3.00 NA 0.373401 10.81
879.77 <= WB
1722 BoLA-DRB3_01601 DEHPIAADEDYHAMN 4 IAADEDYHA 1.000
0 VgR_1799_bp 0.370564 3.00 NA 0.274100 37.47
2576.22 <= WB
834 BoLA-DRB3_01601 RRVIVAAQVPPFAPG 3 IVAAQVPPF 1.000
0 VgR_1799_bp 0.370248 3.01 NA 0.433153 3.67
460.89 <= WB
455 BoLA-DRB3_01601 LSSLEYLELSTLKRD 5 YLELSTLKR 0.820
0 VgR_1799_bp 0.365786 3.06 NA 0.424400 4.34
506.67 <= WB
632 BoLA-DRB3_01601 RHSVLSAINVKSFEQ 3 VLSAINVKS 0.930
0 VgR_1799_bp 0.357739 3.16 NA 0.460334 2.10
343.46 <= WB
79 BoLA-DRB3_01601 VPQIWVCDGEADCHD 4 WVCDGEADC 0.930
0 VgR_1799_bp 0.348085 3.28 NA 0.243374 48.83
3592.22 <= WB
1051 BoLA-DRB3_01601 EDDFHCANGQCVDKR 3 FHCANGQCV 1.000
0 VgR_1799_bp 0.335360 3.44 NA 0.329519 20.15
1414.39 <= WB
292 BoLA-DRB3_01601 NRYFEIHPAEAQAVG 3 FEIHPAEAQ 0.710
0 VgR_1799_bp 0.325969 3.57 NA 0.424735 4.31
504.84 <= WB
1455 BoLA-DRB3_01601 TARVVLLTDKILWPC 3 VVLLTDKIL 1.000
0 VgR_1799_bp 0.319515 3.66 NA 0.404508 6.33
628.35 <= WB
290 BoLA-DRB3_01601 RSNRYFEIHPAEAQA 4 YFEIHPAEA 0.520
0 VgR_1799_bp 0.310045 3.79 NA 0.388861 8.41
744.26 <= WB
1760 BoLA-DRB3_01601 LKRWASSDSLQSSSS 3 WASSDSLQS 1.000
0 VgR_1799_bp 0.304521 3.86 NA 0.377172 10.18
844.60 <= WB
1402 BoLA-DRB3_01601 EPTFKHCTDVIDTAY 3 FKHCTDVID 1.000
0 VgR_1799_bp 0.283270 4.18 NA 0.289908 31.89
2171.21 <= WB
1591 BoLA-DRB3_01601 SHGYTLAEDAHKCVE 3 YTLAEDAHK 1.000
0 VgR_1799_bp 0.278621 4.25 NA 0.367612 11.83
936.64 <= WB
1428 BoLA-DRB3_01601 DGLMFWAVWHEVVQK 4 FWAVWHEVV 0.840
0 VgR_1799_bp 0.268422 4.40 NA 0.396148 7.38
687.83 <= WB
1743 BoLA-DRB3_01601 NPAFNTRKTELLSED 3 FNTRKTELL 1.000
0 VgR_1799_bp 0.268244 4.41 NA 0.313147 24.63
1688.50 <= WB
653 BoLA-DRB3_01601 IGSVFGIDFDVTHQL 4 FGIDFDVTH 0.920
0 VgR_1799_bp 0.265844 4.44 NA 0.437721 3.36
438.66 <= WB
1062 BoLA-DRB3_01601 VDKRLRCDHDNDCED 4 LRCDHDNDC 1.000
0 VgR_1799_bp 0.260712 4.53 NA 0.184903 70.82
6762.64 <= WB

718 BoLA-DRB3_01601 KPTITSYTMDGQNPR 6 YTMDGQNPR 0.690
0 VgR_1799_bp 0.254000 4.67 NA 0.304577 27.15
1852.56 <= WB
294 BoLA-DRB3_01601 YFEIHPAEAQAVGVE 3 IHPAEAQAV 0.870
0 VgR_1799_bp 0.238237 4.98 NA 0.360989 13.09
1006.22 <= WB
658 BoLA-DRB3_01601 GIDFDVTHQLLYWVD 3 FDVTHQLLY 0.770
0 VgR_1799_bp 0.231550 5.13 NA 0.345158 16.42
1194.22 <= WB
474 BoLA-DRB3_01601 EEVFHPFALAVFEDT 3 FHPFALAVF 0.990
0 VgR_1799_bp 0.217881 5.45 NA 0.401997 6.64
645.65 <= WB
1552 BoLA-DRB3_01601 ATVLKVLHAVHQPSG 3 LKVLHAVHQ 0.990
0 VgR_1799_bp 0.216658 5.48 NA 0.360635 13.16
1010.08 <= WB
622 BoLA-DRB3_01601 ANQTLHYSDNRHSVL 4 LHYSDNRHS 0.960
0 VgR_1799_bp 0.211570 5.62 NA 0.402776 6.54
640.23 <= WB
1417 BoLA-DRB3_01601 SHLNTFALSANDGLM 5 FALSANDGL 1.000
0 VgR_1799_bp 0.208212 5.71 NA 0.477806 1.41
284.30 <= WB
983 BoLA-DRB3_01601 LPLYWRCDGSEDCPD 4 WRCDGSEDC 0.940
0 VgR_1799_bp 0.199567 5.95 NA 0.232599 52.92
4036.40 <= WB
1302 BoLA-DRB3_01601 GEPFLLYMLPNQIRS 4 LLYMLPNQI 0.500
0 VgR_1799_bp 0.198271 5.99 NA 0.514765 0.56
190.59 <= WB
1535 BoLA-DRB3_01601 NRYTGTHVGLVHHGT 2 YTGTHVGLV 1.000
0 VgR_1799_bp 0.181006 6.48 NA 0.313825 24.43
1676.16 <= WB
1134 BoLA-DRB3_01601 SAEFFCTGTKRCILQ 3 FFCTGTKRC 1.000
0 VgR_1799_bp 0.179429 6.53 NA 0.423180 4.43
513.40 <= WB
333 BoLA-DRB3_01601 LDGSDFKTLFSAEKT 5 FKTLFSAEK 1.000
0 VgR_1799_bp 0.171706 6.78 NA 0.398636 7.05
669.56 <= WB
673 BoLA-DRB3_01601 ADKYTLEACHANGSG 3 YTLEACHAN 0.910
0 VgR_1799_bp 0.160273 7.18 NA 0.298234 29.19
1984.16 <= WB
478 BoLA-DRB3_01601 HPFALAVFEDTVYWS 4 LAVFEDTVY 0.970
0 VgR_1799_bp 0.157976 7.27 NA 0.312202 24.90
1705.86 <= WB
399 BoLA-DRB3_01601 PPQKVVYWTDWGSRP 4 VVYWTDWGS 0.960
0 VgR_1799_bp 0.153617 7.44 NA 0.315044 24.07
1654.20 <= WB
445 BoLA-DRB3_01601 TNRLYWCDAKLSSLE 4 YWCDAKLSS 0.870
0 VgR_1799_bp 0.149877 7.59 NA 0.412333 5.46
577.34 <= WB
306 BoLA-DRB3_01601 GVEFDSDQHRVFWTD 3 FDSDQHRVF 0.930
0 VgR_1799_bp 0.137104 8.14 NA 0.269365 39.19
2711.64 <= WB
1337 BoLA-DRB3_01601 GMDYRVTDKSIFWTE 3 YRVTDKSIF 0.960
0 VgR_1799_bp 0.136927 8.15 NA 0.375075 10.53
863.98 <= WB
493 BoLA-DRB3_01601 DWASYSLDSSNKRTG 4 YSLDSSNKR 0.930
0 VgR_1799_bp 0.133141 8.34 NA 0.400343 6.84
657.31 <= WB

```
1147   BoLA-DRB3_01601      LQNWLCDGDDDCGDG    3   WLCDGDDDC     1.000
0     VgR_1799_bp      0.132982     8.35         NA      0.134245    86.39
11699.22   <= WB
 368   BoLA-DRB3_01601      VKRILVCTTDGASCS    4   LVCTTDGAS     0.820
0     VgR_1799_bp      0.131860     8.40         NA      0.330415    19.93
1400.75   <= WB
1582   BoLA-DRB3_01601      NPNAYTCACSHGYTL    4   YTCACSHGY     1.000
0     VgR_1799_bp      0.129683     8.51         NA      0.322981    21.90
1518.07   <= WB
 358   BoLA-DRB3_01601      ANNLYITDSLVKRIL    4   YITDSLVKR     0.770
0     VgR_1799_bp      0.129171     8.54         NA      0.543568     0.26
139.56   <= WB
1510   BoLA-DRB3_01601      SPFSIALFEDWLYWS    4   IALFEDWLY     0.960
0     VgR_1799_bp      0.124161     8.80         NA      0.256787    43.67
3106.96   <= WB
 487   BoLA-DRB3_01601      DTVYWSDWASYSLDS    4   WSDWASYSL     0.480
0     VgR_1799_bp      0.112414     9.48         NA      0.323353    21.80
1511.97   <= WB
-----------------------------------------------------------------------------
---------------------------------------------------------------
Number of strong binders: 21 Number of weak binders: 46
-----------------------------------------------------------------------------
---------------------------------------------------------------

# BoLA-DRB3_01801 : Distance to training data  0.000 (using nearest neighbor
BoLA-DRB3_01801)

# Allele: BoLA-DRB3_01801
-----------------------------------------------------------------------------
---------------------------------------------------------------
 Pos              MHC              Peptide   Of        Core  Core_Rel
Inverted        Identity      Score_EL %Rank_EL  Exp_Bind      Score_BA
%Rank_BA  Affinity(nM)  BindLevel
-----------------------------------------------------------------------------
---------------------------------------------------------------
 363   BoLA-DRB3_01801      VLEAIRTSIGKEVFK    4   IRTSIGKEV     1.000
0     Bm86_650_bp      0.682776     0.80         NA      0.437671     2.98
438.90   <= SB
   2   BoLA-DRB3_01801      RGIALFVAAVSLIVE    4   LFVAAVSLI     0.860
0     Bm86_650_bp      0.680958     0.81         NA      0.402248     5.41
643.90   <= SB
 628   BoLA-DRB3_01801      AAAVSATGLLLLLAA    3   VSATGLLLL     0.990
0     Bm86_650_bp      0.589320     1.27         NA      0.507733     0.71
205.66   <= SB
 636   BoLA-DRB3_01801      LLLLLAATSVTAASL    4   LAATSVTAA     0.660
0     Bm86_650_bp      0.482452     1.99         NA      0.400797     5.54
654.09   <= SB
 419   BoLA-DRB3_01801      YPKLLIKKNSATEIE    5   IKKNSATEI     0.970
0     Bm86_650_bp      0.465151     2.15         NA      0.428346     3.51
485.50   <= WB
 360   BoLA-DRB3_01801      EDRVLEAIRTSIGKE    3   VLEAIRTSI     0.890
0     Bm86_650_bp      0.319225     3.88         NA      0.372711     8.50
886.36   <= WB
 341   BoLA-DRB3_01801      YTVSFTPNISFDSDH    2   VSFTPNISF     0.970
0     Bm86_650_bp      0.288722     4.43         NA      0.398598     5.73
669.84   <= WB
```

```
 247   BoLA-DRB3_01801      KSISYTVSCTVEQKQ    2   ISYTVSCTV     0.990
0     Bm86_650_bp      0.285844     4.48         NA      0.424472     3.75
506.28   <= WB
 613   BoLA-DRB3_01801      TTKAKDKDPDPEKSS    3   AKDKDPDPE     0.460
0     Bm86_650_bp      0.193158     6.81         NA      0.031934    97.54
35392.64   <= WB
--------------------------------------------------------------------------------
-------------------------------------------------------------
Number of strong binders: 4 Number of weak binders: 5
--------------------------------------------------------------------------------
-------------------------------------------------------------
--------------------------------------------------------------------------------
-------------------------------------------------------------
 Pos              MHC              Peptide   Of        Core  Core_Rel
Inverted         Identity      Score_EL %Rank_EL  Exp_Bind      Score_BA
%Rank_BA  Affinity(nM)  BindLevel
--------------------------------------------------------------------------------
-------------------------------------------------------------
 285   BoLA-DRB3_01801      DKMVLELEPTQHQRL    6   LEPTQHQRL     0.950
0     AQP1_299_bp      0.871239     0.19         NA      0.346317    12.36
1179.33   <= SB
  68   BoLA-DRB3_01801      NPAVTLAQASVRKFP    5   LAQASVRKF     0.950
0     AQP1_299_bp      0.546428     1.52         NA      0.440363     2.84
426.30   <= SB
 152   BoLA-DRB3_01801      ATGIMMVCVEAIGDT    3   IMMVCVEAI     1.000
0     AQP1_299_bp      0.545975     1.53         NA      0.479385     1.31
279.48   <= SB
 116   BoLA-DRB3_01801      DQGIRQVTGEKATAG    3   IRQVTGEKA     1.000
0     AQP1_299_bp      0.473052     2.08         NA      0.333033    14.71
1361.63   <= WB
 147   BoLA-DRB3_01801      IDQVIATGIMMVCVE    3   VIATGIMMV     0.940
0     AQP1_299_bp      0.436462     2.42         NA      0.448756     2.43
389.30   <= WB
  16   BoLA-DRB3_01801      LGTMILITIGDSIMA    4   ILITIGDSI     0.960
0     AQP1_299_bp      0.419411     2.59         NA      0.400567     5.56
655.72   <= WB
  74   BoLA-DRB3_01801      AQASVRKFPIAKVPL    4   VRKFPIAKV     0.990
0     AQP1_299_bp      0.393252     2.88         NA      0.458283     2.02
351.17   <= WB
 172   BoLA-DRB3_01801      IPPHIHPICLGLMIM    4   IHPICLGLM     1.000
0     AQP1_299_bp      0.348219     3.44         NA      0.426033     3.65
497.80   <= WB
 100   BoLA-DRB3_01801      GAALVFATYKDAIEH    4   VFATYKDAI     0.840
0     AQP1_299_bp      0.327217     3.76         NA      0.421950     3.90
520.28   <= WB
   3   BoLA-DRB3_01801      IENLLIRQLINEFLG    4   LIRQLINEF     0.540
0     AQP1_299_bp      0.306875     4.09         NA      0.401903     5.44
646.31   <= WB
  56   BoLA-DRB3_01801      AVQISGGVSSHLNPA    3   ISGGVSSHL     1.000
0     AQP1_299_bp      0.262307     4.98         NA      0.371657     8.63
896.53   <= WB
  26   BoLA-DRB3_01801      DSIMAIIIAGDNESL    2   IMAIIIAGD     0.380
0     AQP1_299_bp      0.255486     5.14         NA      0.373554     8.40
878.32   <= WB
 232   BoLA-DRB3_01801      VPLLGPHIGAILGVW    3   LGPHIGAIL     0.550
0     AQP1_299_bp      0.227831     5.80         NA      0.461309     1.90
339.85   <= WB
```

```
 224   BoLA-DRB3_01801       LRGWNYVWVPLLGPH    3   WNYVWVPLL     0.870
0     AQP1_299_bp      0.226380     5.84        NA      0.447212     2.50
395.85   <= WB
  86   BoLA-DRB3_01801       VPLYFAAQYLGGFVG    4   FAAQYLGGF     0.600
0     AQP1_299_bp      0.207613     6.35        NA      0.377121     7.99
845.06   <= WB
  21   BoLA-DRB3_01801       LITIGDSIMAIIIAG    3   IGDSIMAII     1.000
0     AQP1_299_bp      0.178761     7.33        NA      0.422560     3.87
516.86   <= WB
  44   BoLA-DRB3_01801       VGPLGWGVAIYVAVQ    3   LGWGVAIYV     0.810
0     AQP1_299_bp      0.168802     7.71        NA      0.356914    10.69
1051.58   <= WB
 236   BoLA-DRB3_01801       GPHIGAILGVWLYKV    6   ILGVWLYKV     0.590
0     AQP1_299_bp      0.159391     8.08        NA      0.473565     1.48
297.65   <= WB
 284   BoLA-DRB3_01801       GDKMVLELEPTQHQR    5   LELEPTQHQ     0.510
0     AQP1_299_bp      0.133986     9.41        NA      0.224280    44.34
4416.57   <= WB
 142   BoLA-DRB3_01801       TLTCFIDQVIATGIM    5   IDQVIATGI     0.980
0     AQP1_299_bp      0.132133     9.51        NA      0.350876    11.62
1122.57   <= WB
  59   BoLA-DRB3_01801       ISGGVSSHLNPAVTL    4   VSSHLNPAV     0.640
0     AQP1_299_bp      0.126939     9.83        NA      0.320084    17.29
1566.41   <= WB
-----------------------------------------------------------------------------
--------------------------------------------------------------
Number of strong binders: 3 Number of weak binders: 18
-----------------------------------------------------------------------------
--------------------------------------------------------------
-----------------------------------------------------------------------------
--------------------------------------------------------------
 Pos               MHC              Peptide   Of        Core  Core_Rel
Inverted         Identity      Score_EL %Rank_EL  Exp_Bind      Score_BA
%Rank_BA  Affinity(nM)  BindLevel
-----------------------------------------------------------------------------
--------------------------------------------------------------
  84   BoLA-DRB3_01801       NPAVTVALATIGKLG    5   VALATIGKL     1.000
0     AQP2_293_bp      0.912151     0.11        NA      0.493890     0.95
238.89   <= SB
  81   BoLA-DRB3_01801       AHLNPAVTVALATIG    2   LNPAVTVAL     0.950
0     AQP2_293_bp      0.647904     0.96        NA      0.441920     2.77
419.18   <= SB
  27   BoLA-DRB3_01801       LAEMVGTLVLTLVGD    3   MVGTLVLTL     1.000
0     AQP2_293_bp      0.643762     0.98        NA      0.482236     1.22
270.99   <= SB
  74   BoLA-DRB3_01801       VAGGVSGAHLNPAVT    4   VSGAHLNPA     1.000
0     AQP2_293_bp      0.428319     2.50        NA      0.315060    18.41
1653.91   <= WB
 116   BoLA-DRB3_01801       ASGLVYLVYADALSQ    4   VYLVYADAL     0.910
0     AQP2_293_bp      0.414684     2.65        NA      0.435993     3.07
446.94   <= WB
 101   BoLA-DRB3_01801       NVLAYVTAQYLGAFL    2   LAYVTAQYL     0.920
0     AQP2_293_bp      0.351807     3.39        NA      0.462376     1.86
335.95   <= WB
 125   BoLA-DRB3_01801       ADALSQVDVNLAIVY    3   LSQVDVNLA     0.980
0     AQP2_293_bp      0.290637     4.39        NA      0.408335     4.89
602.86   <= WB
```

43 BoLA-DRB3_01801 VLASLAVFQLGSVGL 4 LAVFQLGSV 0.760
0 AQP2_293_bp 0.283613 4.53 NA 0.264047 31.46
2872.24 <= WB
251 BoLA-DRB3_01801 GPHLGAVIGVWIYKL 6 VIGVWIYKL 0.650
0 AQP2_293_bp 0.279380 4.62 NA 0.525103 0.45
170.42 <= WB
18 BoLA-DRB3_01801 IENTLARQALAEMVG 4 LARQALAEM 0.640
0 AQP2_293_bp 0.270444 4.81 NA 0.373439 8.42
879.41 <= WB
120 BoLA-DRB3_01801 VYLVYADALSQVDVN 3 VYADALSQV 0.600
0 AQP2_293_bp 0.228581 5.79 NA 0.426160 3.65
497.12 <= WB
247 BoLA-DRB3_01801 VPVVGPHLGAVIGVW 3 VGPHLGAVI 0.610
0 AQP2_293_bp 0.190820 6.89 NA 0.452082 2.29
375.53 <= WB
279 BoLA-DRB3_01801 EDEKRPLLSNAKICA 5 PLLSNAKIC 0.390
0 AQP2_293_bp 0.183851 7.14 NA 0.192942 55.42
6199.28 <= WB
39 BoLA-DRB3_01801 VGDCVLASLAVFQLG 5 LASLAVFQL 0.690
0 AQP2_293_bp 0.173512 7.53 NA 0.418041 4.17
542.76 <= WB
128 BoLA-DRB3_01801 LSQVDVNLAIVYGTN 3 VDVNLAIVY 0.500
0 AQP2_293_bp 0.146501 8.72 NA 0.367977 9.08
932.95 <= WB
112 BoLA-DRB3_01801 GAFLASGLVYLVYAD 3 LASGLVYLV 0.520
0 AQP2_293_bp 0.139180 9.12 NA 0.473688 1.48
297.25 <= WB

Number of strong binders: 3 Number of weak binders: 13

Pos MHC Peptide Of Core Core_Rel
Inverted Identity Score_EL %Rank_EL Exp_Bind Score_BA
%Rank_BA Affinity(nM) BindLevel

----

271 BoLA-DRB3_01801 DPEPLLVFSTTKEIR 5 LVFSTTKEI 1.000
0 VgR_1799_bp 0.929805 0.08 NA 0.532703 0.37
156.97 <= SB
294 BoLA-DRB3_01801 YFEIHPAEAQAVGVE 3 IHPAEAQAV 1.000
0 VgR_1799_bp 0.929670 0.08 NA 0.492065 0.99
243.65 <= SB
834 BoLA-DRB3_01801 RRVIVAAQVPPFAPG 3 IVAAQVPPF 1.000
0 VgR_1799_bp 0.797374 0.39 NA 0.547874 0.23
133.21 <= SB
578 BoLA-DRB3_01801 DFSFVIVAEEDLVYK 4 VIVAEEDLV 0.620
0 VgR_1799_bp 0.781414 0.44 NA 0.423494 3.81
511.66 <= SB
391 BoLA-DRB3_01801 SPRAIIVNPPQKVVY 4 IIVNPPQKV 0.960
0 VgR_1799_bp 0.716590 0.67 NA 0.498224 0.86
227.95 <= SB
587 BoLA-DRB3_01801 EDLVYKIDLNKVGAP 3 VYKIDLNKV 0.990
0 VgR_1799_bp 0.627473 1.05 NA 0.396814 5.89
682.89 <= SB

1785 BoLA-DRB3_01801 DMAAKQDKVFFFRKH 3 AKQDKVFFF 0.460
0 VgR_1799_bp 0.583536 1.31 NA 0.253697 34.61
3212.59 <= SB
1302 BoLA-DRB3_01801 GEPFLLYMLPNQIRS 4 LLYMLPNQI 1.000
0 VgR_1799_bp 0.570379 1.38 NA 0.507200 0.72
206.85 <= SB
455 BoLA-DRB3_01801 LSSLEYLELSTLKRD 3 LEYLELSTL 1.000
0 VgR_1799_bp 0.539445 1.57 NA 0.357508 10.60
1044.84 <= SB
358 BoLA-DRB3_01801 ANNLYITDSLVKRIL 5 ITDSLVKRI 0.980
0 VgR_1799_bp 0.486560 1.95 NA 0.456208 2.11
359.14 <= SB
1344 BoLA-DRB3_01801 DKSIFWTEMDEGVIN 3 IFWTEMDEG 0.930
0 VgR_1799_bp 0.418249 2.61 NA 0.301138 21.58
1922.79 <= WB
663 BoLA-DRB3_01801 VTHQLLYWVDADKYT 5 LYWVDADKY 0.960
0 VgR_1799_bp 0.417049 2.62 NA 0.366944 9.22
943.43 <= WB
775 BoLA-DRB3_01801 GTPFIVQQVKAHISS 4 IVQQVKAHI 0.860
0 VgR_1799_bp 0.400154 2.81 NA 0.435582 3.10
448.93 <= WB
759 BoLA-DRB3_01801 AVRGTIESLDLQKVF 5 IESLDLQKV 1.000
0 VgR_1799_bp 0.391677 2.90 NA 0.413979 4.46
567.15 <= WB
730 BoLA-DRB3_01801 NPRVLPLSTLLLPVS 3 VLPLSTLLL 0.590
0 VgR_1799_bp 0.379077 3.05 NA 0.508930 0.69
203.01 <= WB
606 BoLA-DRB3_01801 LPVHNLGIISALTFD 5 LGIISALTF 0.980
0 VgR_1799_bp 0.369871 3.16 NA 0.415279 4.36
559.22 <= WB
338 BoLA-DRB3_01801 FKTLFSAEKTLLEDL 3 LFSAEKTLL 0.980
0 VgR_1799_bp 0.329661 3.72 NA 0.365255 9.45
960.83 <= WB
1545 BoLA-DRB3_01801 VHHGTAKATVLKVLH 5 AKATVLKVL 0.520
0 VgR_1799_bp 0.309221 4.05 NA 0.374234 8.32
871.88 <= WB
417 BoLA-DRB3_01801 HASMDGTNIQQLVST 3 MDGTNIQQL 1.000
0 VgR_1799_bp 0.300034 4.22 NA 0.333504 14.62
1354.71 <= WB
821 BoLA-DRB3_01801 HVSLRSTRNGTFSRR 3 LRSTRNGTF 1.000
0 VgR_1799_bp 0.290046 4.40 NA 0.317600 17.84
1609.08 <= WB
412 BoLA-DRB3_01801 RPAIMHASMDGTNIQ 3 IMHASMDGT 0.970
0 VgR_1799_bp 0.286151 4.48 NA 0.336489 14.05
1311.65 <= WB
1428 BoLA-DRB3_01801 DGLMFWAVWHEVVQK 4 FWAVWHEVV 0.520
0 VgR_1799_bp 0.255310 5.14 NA 0.417447 4.21
546.26 <= WB
718 BoLA-DRB3_01801 KPTITSYTMDGQNPR 3 ITSYTMDGQ 0.920
0 VgR_1799_bp 0.252002 5.22 NA 0.238915 39.41
3769.78 <= WB
632 BoLA-DRB3_01801 RHSVLSAINVKSFEQ 4 LSAINVKSF 0.840
0 VgR_1799_bp 0.240218 5.50 NA 0.346880 12.27
1172.17 <= WB
1443 BoLA-DRB3_01801 DHGLIERSNMDGTAR 4 IERSNMDGT 0.990
0 VgR_1799_bp 0.238978 5.53 NA 0.221891 45.15
4532.22 <= WB

1409 BoLA-DRB3_01801 TDVIDTAYSHLNTFA 5 TAYSHLNTF 0.510
0 VgR_1799_bp 0.231500 5.71 NA 0.345765 12.46
1186.40 <= WB
1376 BoLA-DRB3_01801 KPYHIAVDWVAGNIY 6 VDWVAGNIY 0.510
0 VgR_1799_bp 0.222594 5.94 NA 0.392944 6.26
712.10 <= WB
1477 BoLA-DRB3_01801 HKRIYWSDANKNVIE 3 IYWSDANKN 0.890
0 VgR_1799_bp 0.218567 6.04 NA 0.300283 21.78
1940.66 <= WB
474 BoLA-DRB3_01801 EEVFHPFALAVFEDT 3 FHPFALAVF 0.890
0 VgR_1799_bp 0.215849 6.12 NA 0.400262 5.58
657.89 <= WB
1669 BoLA-DRB3_01801 STALASILVSALCVA 3 LASILVSAL 1.000
0 VgR_1799_bp 0.202857 6.49 NA 0.409730 4.78
593.83 <= WB
807 BoLA-DRB3_01801 LEYIDRSSEPSVHRH 3 IDRSSEPSV 1.000
0 VgR_1799_bp 0.190253 6.91 NA 0.251369 35.36
3294.54 <= WB
638 BoLA-DRB3_01801 AINVKSFEQWTVHDH 3 VKSFEQWTV 0.880
0 VgR_1799_bp 0.182187 7.20 NA 0.336630 14.02
1309.65 <= WB
1544 BoLA-DRB3_01801 LVHHGTAKATVLKVL 5 TAKATVLKV 0.620
0 VgR_1799_bp 0.168240 7.73 NA 0.435255 3.11
450.53 <= WB
1487 BoLA-DRB3_01801 KNVIESATYDGKDRK 3 IESATYDGK 0.770
0 VgR_1799_bp 0.166220 7.81 NA 0.234933 40.73
3935.74 <= WB
286 BoLA-DRB3_01801 GLWLRSNRYFEIHPA 3 LRSNRYFEI 0.960
0 VgR_1799_bp 0.165181 7.85 NA 0.471941 1.53
302.92 <= WB
686 BoLA-DRB3_01801 SGHVIIRDDLHRPVG 5 IRDDLHRPV 0.680
0 VgR_1799_bp 0.162048 7.97 NA 0.285424 25.50
2279.14 <= WB
658 BoLA-DRB3_01801 GIDFDVTHQLLYWVD 3 FDVTHQLLY 0.560
0 VgR_1799_bp 0.158503 8.13 NA 0.390187 6.54
733.66 <= WB
575 BoLA-DRB3_01801 VTKDFSFVIVAEEDL 6 FVIVAEEDL 0.640
0 VgR_1799_bp 0.157593 8.17 NA 0.344048 12.76
1208.64 <= WB
1353 BoLA-DRB3_01801 DEGVINVMTLGNGKQ 3 VINVMTLGN 0.540
0 VgR_1799_bp 0.147388 8.67 NA 0.311602 19.18
1716.97 <= WB
387 BoLA-DRB3_01801 DSVDSPRAIIVNPPQ 2 VDSPRAIIV 0.820
0 VgR_1799_bp 0.144770 8.81 NA 0.285706 25.43
2272.20 <= WB
1554 BoLA-DRB3_01801 VLKVLHAVHQPSGVN 3 VLHAVHQPS 0.630
0 VgR_1799_bp 0.143503 8.88 NA 0.357454 10.61
1045.45 <= WB
467 BoLA-DRB3_01801 KRDVVMHEEVFHPFA 5 MHEEVFHPF 0.580
0 VgR_1799_bp 0.139992 9.07 NA 0.301085 21.59
1923.89 <= WB
600 BoLA-DRB3_01801 APVPVTLPVHNLGII 3 PVTLPVHNL 0.390
0 VgR_1799_bp 0.138861 9.14 NA 0.331327 15.03
1386.99 <= WB
351 BoLA-DRB3_01801 DLSLDWVANNLYITD 3 LDWVANNLY 0.960
0 VgR_1799_bp 0.130184 9.63 NA 0.326313 16.02
1464.32 <= WB

423 BoLA-DRB3_01801 TNIQQLVSTDLGWPN 2 IQQLVSTDL 0.820
0 VgR_1799_bp 0.127142 9.82 NA 0.345510 12.51
1189.68 <= WB
741 BoLA-DRB3_01801 LPVSISVDLVARTLV 4 ISVDLVART 0.670
0 VgR_1799_bp 0.125929 9.89 NA 0.409046 4.84
598.24 <= WB

Number of strong binders: 10 Number of weak binders: 36

BoLA-DRB3_02002 : Distance to training data 0.000 (using nearest neighbor BoLA-DRB3_02002)

Allele: BoLA-DRB3_02002

Pos MHC Peptide Of Core Core_Rel
Inverted Identity Score_EL %Rank_EL Exp_Bind Score_BA
%Rank_BA Affinity(nM) BindLevel
----
633 BoLA-DRB3_02002 ATGLLLLLAATSVTA 4 LLLLAATSV 0.690
0 Bm86_650_bp 0.509370 2.35 NA 0.485458 9.11
261.71 <= WB
2 BoLA-DRB3_02002 RGIALFVAAVSLIVE 5 FVAAVSLIV 0.850
0 Bm86_650_bp 0.239256 5.51 NA 0.423243 18.79
513.05 <= WB
360 BoLA-DRB3_02002 EDRVLEAIRTSIGKE 4 LEAIRTSIG 0.940
0 Bm86_650_bp 0.236669 5.56 NA 0.431534 17.26
469.03 <= WB
412 BoLA-DRB3_02002 IGEWCMMYPKLLIKK 3 WCMMYPKLL 1.000
0 Bm86_650_bp 0.200098 6.38 NA 0.556712 3.12
121.06 <= WB
213 BoLA-DRB3_02002 EAGFVCKHGCRSTDK 3 FVCKHGCRS 0.990
0 Bm86_650_bp 0.197798 6.43 NA 0.367327 32.20
939.53 <= WB
399 BoLA-DRB3_02002 KYVLRKLQACEHPIG 3 LRKLQACEH 0.980
0 Bm86_650_bp 0.187530 6.70 NA 0.582997 1.91
91.09 <= WB
613 BoLA-DRB3_02002 TTKAKDKDPDPEKSS 4 KDKDPDPEK 0.180
0 Bm86_650_bp 0.164852 7.35 NA 0.081882 96.67
20616.21 <= WB
374 BoLA-DRB3_02002 EVFKVEILNCTQDIK 4 VEILNCTQD 0.870
0 Bm86_650_bp 0.160901 7.47 NA 0.502193 7.25
218.36 <= WB
358 BoLA-DRB3_02002 RYEDRVLEAIRTSIG 3 DRVLEAIRT 0.810
0 Bm86_650_bp 0.140747 8.16 NA 0.502155 7.25
218.45 <= WB
470 BoLA-DRB3_02002 YTTTYEMTRGRLRRS 4 YEMTRGRLR 0.990
0 Bm86_650_bp 0.129326 8.60 NA 0.467567 11.45
317.60 <= WB
636 BoLA-DRB3_02002 LLLLLAATSVTAASL 3 LLAATSVTA 0.310
0 Bm86_650_bp 0.114441 9.25 NA 0.358044 34.77
1038.80 <= WB

Number of strong binders: 0 Number of weak binders: 11

Pos	MHC	Peptide	Of	Core	Core_Rel	Inverted	Identity	Score_EL	%Rank_EL	Exp_Bind	Score_BA	%Rank_BA	Affinity(nM)	BindLevel
226	BoLA-DRB3_02002	GWNYVWVPLLGPHIG	5	WVPLLGPHI	0.990	0	AQP1_299_bp	0.885437	0.42	NA	0.707291	0.11	23.74	<= SB
116	BoLA-DRB3_02002	DQGIRQVTGEKATAG	3	IRQVTGEKA	1.000	0	AQP1_299_bp	0.859942	0.51	NA	0.524831	5.14	170.92	<= SB
206	BoLA-DRB3_02002	ISPRLFTLMAGWGPE	4	LFTLMAGWG	0.500	0	AQP1_299_bp	0.833816	0.61	NA	0.636165	0.64	51.24	<= SB
207	BoLA-DRB3_02002	SPRLFTLMAGWGPET	4	FTLMAGWGP	0.560	0	AQP1_299_bp	0.781464	0.82	NA	0.662332	0.36	38.61	<= SB
224	BoLA-DRB3_02002	LRGWNYVWVPLLGPH	3	WNYVWVPLL	0.760	0	AQP1_299_bp	0.689997	1.25	NA	0.636810	0.63	50.89	<= SB
3	BoLA-DRB3_02002	IENLLIRQLINEFLG	5	IRQLINEFL	0.990	0	AQP1_299_bp	0.560464	1.99	NA	0.560869	2.89	115.73	<= SB
285	BoLA-DRB3_02002	DKMVLELEPTQHQRL	3	VLELEPTQH	0.470	0	AQP1_299_bp	0.543972	2.10	NA	0.376486	29.76	850.89	<= WB
51	BoLA-DRB3_02002	VAIYVAVQISGGVSS	3	YVAVQISGG	1.000	0	AQP1_299_bp	0.432460	2.98	NA	0.525050	5.12	170.52	<= WB
74	BoLA-DRB3_02002	AQASVRKFPIAKVPL	4	VRKFPIAKV	0.990	0	AQP1_299_bp	0.282376	4.76	NA	0.565474	2.65	110.11	<= WB
36	BoLA-DRB3_02002	DNESLAACVGPLGWG	4	LAACVGPLG	1.000	0	AQP1_299_bp	0.255109	5.21	NA	0.449434	14.13	386.45	<= WB
86	BoLA-DRB3_02002	VPLYFAAQYLGGFVG	3	YFAAQYLGG	0.410	0	AQP1_299_bp	0.196652	6.46	NA	0.560785	2.90	115.84	<= WB
165	BoLA-DRB3_02002	DTRNFGGIPPHIHPI	4	FGGIPPHIH	1.000	0	AQP1_299_bp	0.191672	6.59	NA	0.375465	30.02	860.34	<= WB
272	BoLA-DRB3_02002	KEDLVETLYKVDGDK	4	VETLYKVDG	0.910	0	AQP1_299_bp	0.182156	6.84	NA	0.414546	20.58	563.68	<= WB
80	BoLA-DRB3_02002	KFPIAKVPLYFAAQY	3	IAKVPLYFA	0.970	0	AQP1_299_bp	0.174954	7.04	NA	0.559541	2.96	117.41	<= WB
26	BoLA-DRB3_02002	DSIMAIIIAGDNESL	3	MAIIIAGDN	0.760	0	AQP1_299_bp	0.159006	7.53	NA	0.393194	25.55	710.17	<= WB

```
 135   BoLA-DRB3_02002      YPRPHVSTLTCFIDQ    5   VSTLTCFID     0.980
0     AQP1_299_bp      0.146252     7.95        NA      0.359787    34.29
1019.39   <= WB
  24   BoLA-DRB3_02002      IGDSIMAIIIAGDNE    4   IMAIIIAGD     0.500
0     AQP1_299_bp      0.113492     9.30        NA      0.450175    14.00
383.36   <= WB
-----------------------------------------------------------------------------
---------------------------------------------------------------
Number of strong binders: 6 Number of weak binders: 11
-----------------------------------------------------------------------------
---------------------------------------------------------------
-----------------------------------------------------------------------------
---------------------------------------------------------------
 Pos              MHC              Peptide   Of        Core  Core_Rel
Inverted        Identity      Score_EL %Rank_EL  Exp_Bind      Score_BA
%Rank_BA  Affinity(nM)  BindLevel
-----------------------------------------------------------------------------
---------------------------------------------------------------
  66   BoLA-DRB3_02002      LAVFLGVLVAGGVSG    4   LGVLVAGGV     0.920
0     AQP2_293_bp      0.597956     1.75        NA      0.531996     4.61
158.17   <= SB
 222   BoLA-DRB3_02002      APRIFTAMCGWGSAV    4   FTAMCGWGS     1.000
0     AQP2_293_bp      0.548488     2.07        NA      0.545669     3.76
136.42   <= WB
 241   BoLA-DRB3_02002      SYNWFWVPVVGPHLG    5   WVPVVGPHL     0.970
0     AQP2_293_bp      0.492980     2.47        NA      0.565833     2.63
109.68   <= WB
 144   BoLA-DRB3_02002      TAPVFSCFPAPGVST    4   FSCFPAPGV     0.980
0     AQP2_293_bp      0.484895     2.54        NA      0.501038     7.36
221.11   <= WB
  39   BoLA-DRB3_02002      VGDCVLASLAVFQLG    5   LASLAVFQL     1.000
0     AQP2_293_bp      0.295180     4.56        NA      0.404690    22.80
627.11   <= WB
 279   BoLA-DRB3_02002      EDEKRPLLSNAKICA    3   KRPLLSNAK     0.660
0     AQP2_293_bp      0.241102     5.47        NA      0.339203    40.11
1273.69   <= WB
 239   BoLA-DRB3_02002      FRSYNWFWVPVVGPH    3   YNWFWVPVV     0.740
0     AQP2_293_bp      0.208094     6.18        NA      0.531745     4.63
158.60   <= WB
 192   BoLA-DRB3_02002      QPLLVGLTVSACMYA    3   LVGLTVSAC     1.000
0     AQP2_293_bp      0.128129     8.65        NA      0.395745    24.92
690.84   <= WB
  23   BoLA-DRB3_02002      ARQALAEMVGTLVLT    4   LAEMVGTLV     1.000
0     AQP2_293_bp      0.126570     8.72        NA      0.441120    15.53
422.82   <= WB
 116   BoLA-DRB3_02002      ASGLVYLVYADALSQ    3   LVYLVYADA     0.630
0     AQP2_293_bp      0.125677     8.75        NA      0.518507     5.69
183.03   <= WB
 258   BoLA-DRB3_02002      IGVWIYKLAVDNHWK    4   IYKLAVDNH     0.580
0     AQP2_293_bp      0.104539     9.75        NA      0.571082     2.39
103.63   <= WB
-----------------------------------------------------------------------------
---------------------------------------------------------------
Number of strong binders: 1 Number of weak binders: 10
-----------------------------------------------------------------------------
---------------------------------------------------------------
```

Pos	MHC	Peptide	Of	Core	Core_Rel	Inverted	Identity	Score_EL	%Rank_EL	Exp_Bind	Score_BA	%Rank_BA	Affinity(nM)	BindLevel
1413	BoLA-DRB3_02002	DTAYSHLNTFALSAN	3	YSHLNTFAL	1.000	0	VgR_1799_bp	0.968424	0.11	NA	0.638550	0.60	49.94	<= SB
1551	BoLA-DRB3_02002	KATVLKVLHAVHQPS	4	LKVLHAVHQ	1.000	0	VgR_1799_bp	0.951623	0.18	NA	0.666695	0.31	36.83	<= SB
455	BoLA-DRB3_02002	LSSLEYLELSTLKRD	5	YLELSTLKR	0.600	0	VgR_1799_bp	0.821112	0.67	NA	0.622237	0.87	59.58	<= SB
1365	BoLA-DRB3_02002	GKQFTLLEDIHKPYH	3	FTLLEDIHK	1.000	0	VgR_1799_bp	0.820920	0.67	NA	0.566920	2.58	108.40	<= SB
730	BoLA-DRB3_02002	NPRVLPLSTLLLPVS	3	VLPLSTLLL	1.000	0	VgR_1799_bp	0.815368	0.70	NA	0.629512	0.75	55.07	<= SB
1729	BoLA-DRB3_02002	DEDYHAMNTTPGFIN	3	YHAMNTTPG	1.000	0	VgR_1799_bp	0.814965	0.70	NA	0.391416	25.99	723.97	<= SB
215	BoLA-DRB3_02002	NAGFRLLADHISCAD	3	FRLLADHIS	1.000	0	VgR_1799_bp	0.804097	0.74	NA	0.560816	2.90	115.80	<= SB
333	BoLA-DRB3_02002	LDGSDFKTLFSAEKT	5	FKTLFSAEK	1.000	0	VgR_1799_bp	0.760812	0.90	NA	0.614423	1.01	64.84	<= SB
290	BoLA-DRB3_02002	RSNRYFEIHPAEAQA	4	YFEIHPAEA	0.930	0	VgR_1799_bp	0.717390	1.12	NA	0.594975	1.51	80.02	<= SB
1353	BoLA-DRB3_02002	DEGVINVMTLGNGKQ	4	INVMTLGNG	0.940	0	VgR_1799_bp	0.701038	1.20	NA	0.529174	4.81	163.08	<= SB
1302	BoLA-DRB3_02002	GEPFLLYMLPNQIRS	4	LLYMLPNQI	0.790	0	VgR_1799_bp	0.644307	1.49	NA	0.682695	0.21	30.97	<= SB
760	BoLA-DRB3_02002	VRGTIESLDLQKVFT	4	IESLDLQKV	1.000	0	VgR_1799_bp	0.561056	1.98	NA	0.484484	9.22	264.48	<= SB
775	BoLA-DRB3_02002	GTPFIVQQVKAHISS	3	FIVQQVKAH	0.780	0	VgR_1799_bp	0.540009	2.13	NA	0.609294	1.12	68.54	<= WB
578	BoLA-DRB3_02002	DFSFVIVAEEDLVYK	3	FVIVAEEDL	0.890	0	VgR_1799_bp	0.501062	2.42	NA	0.468051	11.39	315.95	<= WB
1344	BoLA-DRB3_02002	DKSIFWTEMDEGVIN	5	WTEMDEGVI	0.880	0	VgR_1799_bp	0.472121	2.64	NA	0.466164	11.64	322.46	<= WB
454	BoLA-DRB3_02002	KLSSLEYLELSTLKR	4	LEYLELSTL	0.710	0	VgR_1799_bp	0.428849	3.01	NA	0.628225	0.77	55.84	<= WB

1454 BoLA-DRB3_02002 GTARVVLLTDKILWP 4 VVLLTDKIL 0.970
0 VgR_1799_bp 0.427805 3.02 NA 0.417907 19.86
543.55 <= WB
1654 BoLA-DRB3_02002 DTSVVSMLSQKSTSS 3 VVSMLSQKS 0.840
0 VgR_1799_bp 0.395101 3.36 NA 0.570548 2.41
104.23 <= WB
1373 BoLA-DRB3_02002 DIHKPYHIAVDWVAG 5 YHIAVDWVA 0.980
0 VgR_1799_bp 0.363110 3.72 NA 0.391803 25.89
720.94 <= WB
629 BoLA-DRB3_02002 SDNRHSVLSAINVKS 4 HSVLSAINV 1.000
0 VgR_1799_bp 0.352996 3.84 NA 0.522043 5.38
176.16 <= WB
1051 BoLA-DRB3_02002 EDDFHCANGQCVDKR 3 FHCANGQCV 0.990
0 VgR_1799_bp 0.331340 4.11 NA 0.357643 34.87
1043.32 <= WB
673 BoLA-DRB3_02002 ADKYTLEACHANGSG 3 YTLEACHAN 0.670
0 VgR_1799_bp 0.317675 4.27 NA 0.425945 18.31
498.27 <= WB
1785 BoLA-DRB3_02002 DMAAKQDKVFFFRKH 6 DKVFFFRKH 0.280
0 VgR_1799_bp 0.313288 4.33 NA 0.314615 47.63
1661.89 <= WB
423 BoLA-DRB3_02002 TNIQQLVSTDLGWPN 2 IQQLVSTDL 0.990
0 VgR_1799_bp 0.270970 4.94 NA 0.425413 18.40
501.15 <= WB
474 BoLA-DRB3_02002 EEVFHPFALAVFEDT 3 FHPFALAVF 0.980
0 VgR_1799_bp 0.270774 4.94 NA 0.553461 3.29
125.39 <= WB
271 BoLA-DRB3_02002 DPEPLLVFSTTKEIR 4 LLVFSTTKE 0.540
0 VgR_1799_bp 0.248507 5.34 NA 0.508255 6.65
204.50 <= WB
1393 BoLA-DRB3_02002 DGWVHIQACEPTFKH 2 WVHIQACEP 0.760
0 VgR_1799_bp 0.231204 5.67 NA 0.476485 10.24
288.39 <= WB
575 BoLA-DRB3_02002 VTKDFSFVIVAEEDL 4 FSFVIVAEE 0.730
0 VgR_1799_bp 0.230319 5.69 NA 0.523439 5.26
173.52 <= WB
606 BoLA-DRB3_02002 LPVHNLGIISALTFD 2 VHNLGIISA 0.550
0 VgR_1799_bp 0.179492 6.91 NA 0.507745 6.70
205.63 <= WB
280 BoLA-DRB3_02002 TTKEIRGLWLRSNRY 4 IRGLWLRSN 1.000
0 VgR_1799_bp 0.170872 7.16 NA 0.661182 0.37
39.09 <= WB
751 BoLA-DRB3_02002 ARTLVWADAVRGTIE 3 LVWADAVRG 0.990
0 VgR_1799_bp 0.164420 7.36 NA 0.549077 3.54
131.48 <= WB
1428 BoLA-DRB3_02002 DGLMFWAVWHEVVQK 4 FWAVWHEVV 0.730
0 VgR_1799_bp 0.163950 7.38 NA 0.488274 8.78
253.85 <= WB
1548 BoLA-DRB3_02002 GTAKATVLKVLHAVH 4 ATVLKVLHA 0.990
0 VgR_1799_bp 0.141960 8.11 NA 0.599711 1.38
76.02 <= WB
391 BoLA-DRB3_02002 SPRAIIVNPPQKVVY 4 IIVNPPQKV 0.870
0 VgR_1799_bp 0.111436 9.39 NA 0.471004 10.98
306.01 <= WB
587 BoLA-DRB3_02002 EDLVYKIDLNKVGAP 4 YKIDLNKVG 0.600
0 VgR_1799_bp 0.110908 9.42 NA 0.424446 18.57
506.42 <= WB

339   BoLA-DRB3_02002   KTLFSAEKTLLEDLS   3   FSAEKTLLE   1.000
0   VgR_1799_bp   0.110431   9.44   NA   0.432872   17.01
462.29   <= WB

Number of strong binders: 12 Number of weak binders: 24

BoLA-DRB3_02601 : Distance to training data 0.000 (using nearest neighbor BoLA-DRB3_02601)

Allele: BoLA-DRB3_02601

Pos   MHC   Peptide   Of   Core Core_Rel
Inverted   Identity   Score_EL %Rank_EL Exp_Bind   Score_BA
%Rank_BA Affinity(nM) BindLevel
----
336   BoLA-DRB3_02601   LNEYYYTVSFTPNIS   5   YTVSFTPNI   0.900
0   Bm86_650_bp   0.363858   2.26   NA   0.614810   0.47
64.56   <= WB
2   BoLA-DRB3_02601   RGIALFVAAVSLIVE   5   FVAAVSLIV   0.970
0   Bm86_650_bp   0.355142   2.35   NA   0.404158   27.88
630.73   <= WB
341   BoLA-DRB3_02601   YTVSFTPNISFDSDH   2   VSFTPNISF   0.940
0   Bm86_650_bp   0.266837   3.32   NA   0.661973   0.08
38.76   <= WB
501   BoLA-DRB3_02601   ANKGQICVYENGKAN   5   ICVYENGKA   1.000
0   Bm86_650_bp   0.250519   3.55   NA   0.394305   30.87
701.69   <= WB
613   BoLA-DRB3_02601   TTKAKDKDPDPEKSS   3   AKDKDPDPE   0.250
0   Bm86_650_bp   0.241909   3.69   NA   0.079962   97.35
21048.97   <= WB
636   BoLA-DRB3_02601   LLLLLAATSVTAASL   4   LAATSVTAA   0.380
0   Bm86_650_bp   0.168068   5.20   NA   0.406979   27.05
611.77   <= WB
419   BoLA-DRB3_02601   YPKLLIKKNSATEIE   5   IKKNSATEI   0.690
0   Bm86_650_bp   0.120243   6.73   NA   0.587377   1.06
86.88   <= WB

Number of strong binders: 0 Number of weak binders: 7

Pos   MHC   Peptide   Of   Core Core_Rel
Inverted   Identity   Score_EL %Rank_EL Exp_Bind   Score_BA
%Rank_BA Affinity(nM) BindLevel
----
100   BoLA-DRB3_02601   GAALVFATYKDAIEH   5   FATYKDAIE   0.990
0   AQP1_299_bp   0.809919   0.30   NA   0.631958   0.28
53.63   <= SB

```
  74   BoLA-DRB3_02601      AQASVRKFPIAKVPL    4   VRKFPIAKV     1.000
0     AQP1_299_bp      0.684693     0.62         NA      0.666779     0.07
36.80   <= SB
 285   BoLA-DRB3_02601      DKMVLELEPTQHQRL    6   LEPTQHQRL     0.450
0     AQP1_299_bp      0.679873     0.63         NA      0.376190    36.81
853.62   <= SB
 140   BoLA-DRB3_02601      VSTLTCFIDQVIATG    3   LTCFIDQVI     1.000
0     AQP1_299_bp      0.612344     0.85         NA      0.461159    13.72
340.41   <= SB
 224   BoLA-DRB3_02601      LRGWNYVWVPLLGPH    3   WNYVWVPLL     0.870
0     AQP1_299_bp      0.538168     1.14         NA      0.627662     0.32
56.18   <= SB
 276   BoLA-DRB3_02601      VETLYKVDGDKMVLE    4   YKVDGDKMV     0.960
0     AQP1_299_bp      0.407664     1.88         NA      0.404084    27.90
631.24   <= SB
   3   BoLA-DRB3_02601      IENLLIRQLINEFLG    5   IRQLINEFL     0.930
0     AQP1_299_bp      0.384321     2.08         NA      0.562517     1.98
113.69   <= WB
 128   BoLA-DRB3_02601      TAGIFATYPRPHVST    4   FATYPRPHV     0.990
0     AQP1_299_bp      0.363690     2.27         NA      0.473734    11.30
297.10   <= WB
  86   BoLA-DRB3_02601      VPLYFAAQYLGGFVG    4   FAAQYLGGF     0.830
0     AQP1_299_bp      0.267335     3.32         NA      0.513585     5.61
193.04   <= WB
 284   BoLA-DRB3_02601      GDKMVLELEPTQHQR    3   MVLELEPTQ     0.530
0     AQP1_299_bp      0.210089     4.24         NA      0.377393    36.42
842.58   <= WB
  26   BoLA-DRB3_02601      DSIMAIIIAGDNESL    5   IIIAGDNES     0.770
0     AQP1_299_bp      0.192564     4.59         NA      0.435198    19.47
450.80   <= WB
 226   BoLA-DRB3_02601      GWNYVWVPLLGPHIG    5   WVPLLGPHI     0.790
0     AQP1_299_bp      0.188758     4.68         NA      0.587248     1.06
87.00   <= WB
 244   BoLA-DRB3_02601      GVWLYKVAIGDHWPE    4   YKVAIGDHW     0.960
0     AQP1_299_bp      0.186372     4.74         NA      0.541188     3.22
143.20   <= WB
  59   BoLA-DRB3_02601      ISGGVSSHLNPAVTL    4   VSSHLNPAV     0.980
0     AQP1_299_bp      0.159150     5.44         NA      0.495785     7.78
234.04   <= WB
 116   BoLA-DRB3_02601      DQGIRQVTGEKATAG    3   IRQVTGEKA     1.000
0     AQP1_299_bp      0.154186     5.59         NA      0.397374    29.95
678.77   <= WB
----------------------------------------------------------------------------
------------------------------------------------------------
Number of strong binders: 6 Number of weak binders: 9
----------------------------------------------------------------------------
------------------------------------------------------------
----------------------------------------------------------------------------
------------------------------------------------------------
 Pos                MHC              Peptide   Of        Core  Core_Rel
Inverted        Identity      Score_EL %Rank_EL  Exp_Bind      Score_BA
%Rank_BA  Affinity(nM)  BindLevel
----------------------------------------------------------------------------
------------------------------------------------------------
 116   BoLA-DRB3_02601      ASGLVYLVYADALSQ    5   YLVYADALS     0.990
0     AQP2_293_bp      0.747843     0.46         NA      0.549063     2.69
131.50   <= SB
```

```
 258   BoLA-DRB3_02601      IGVWIYKLAVDNHWK    5   YKLAVDNHW     1.000
0     AQP2_293_bp      0.677640     0.64         NA      0.586151     1.10
88.04   <= SB
 145   BoLA-DRB3_02601      APVFSCFPAPGVSTL    3   FSCFPAPGV     1.000
0     AQP2_293_bp      0.635814     0.77         NA      0.528738     4.16
163.85   <= SB
 239   BoLA-DRB3_02601      FRSYNWFWVPVVGPH    3   YNWFWVPVV     0.960
0     AQP2_293_bp      0.511976     1.26         NA      0.581742     1.23
92.34   <= SB
 241   BoLA-DRB3_02601      SYNWFWVPVVGPHLG    5   WVPVVGPHL     0.740
0     AQP2_293_bp      0.242360     3.68         NA      0.550429     2.60
129.57   <= WB
  43   BoLA-DRB3_02601      VLASLAVFQLGSVGL    4   LAVFQLGSV     0.780
0     AQP2_293_bp      0.192043     4.60         NA      0.315755    57.12
1641.52   <= WB
 279   BoLA-DRB3_02601      EDEKRPLLSNAKICA    3   KRPLLSNAK     0.470
0     AQP2_293_bp      0.186196     4.74         NA      0.306391    60.22
1816.55   <= WB
 135   BoLA-DRB3_02601      LAIVYGTNATAPVFS    4   YGTNATAPV     0.800
0     AQP2_293_bp      0.165771     5.26         NA      0.556033     2.30
121.95   <= WB
 133   BoLA-DRB3_02601      VNLAIVYGTNATAPV    4   IVYGTNATA     0.550
0     AQP2_293_bp      0.108043     7.28         NA      0.504277     6.71
213.49   <= WB
  80   BoLA-DRB3_02601      GAHLNPAVTVALATI    3   LNPAVTVAL     1.000
0     AQP2_293_bp      0.103477     7.49         NA      0.481998     9.90
271.69   <= WB
----------------------------------------------------------------------------
--------------------------------------------------------------
Number of strong binders: 4 Number of weak binders: 6
----------------------------------------------------------------------------
--------------------------------------------------------------
----------------------------------------------------------------------------
--------------------------------------------------------------
 Pos              MHC              Peptide   Of        Core  Core_Rel
Inverted        Identity      Score_EL %Rank_EL  Exp_Bind      Score_BA
%Rank_BA  Affinity(nM)  BindLevel
----------------------------------------------------------------------------
--------------------------------------------------------------
 578   BoLA-DRB3_02601      DFSFVIVAEEDLVYK    3   FVIVAEEDL     0.850
0     VgR_1799_bp      0.938081     0.09         NA      0.581292     1.25
92.79   <= SB
 478   BoLA-DRB3_02601      HPFALAVFEDTVYWS    4   LAVFEDTVY     1.000
0     VgR_1799_bp      0.810768     0.30         NA      0.564350     1.90
111.46   <= SB
 474   BoLA-DRB3_02601      EEVFHPFALAVFEDT    3   FHPFALAVF     1.000
0     VgR_1799_bp      0.807983     0.31         NA      0.608228     0.57
69.33   <= SB
1344   BoLA-DRB3_02601      DKSIFWTEMDEGVIN    4   FWTEMDEGV     0.760
0     VgR_1799_bp      0.738760     0.48         NA      0.517706     5.18
184.62   <= SB
1365   BoLA-DRB3_02601      GKQFTLLEDIHKPYH    3   FTLLEDIHK     1.000
0     VgR_1799_bp      0.580169     0.98         NA      0.478532    10.48
282.07   <= SB
1785   BoLA-DRB3_02601      DMAAKQDKVFFFRKH    1   MAAKQDKVF     0.470
0     VgR_1799_bp      0.578664     0.99         NA      0.375106    37.16
863.69   <= SB
```

1428 BoLA-DRB3_02601 DGLMFWAVWHEVVQK 5 WAVWHEVVQ 0.640
0 VgR_1799_bp 0.575206 1.00 NA 0.552722 2.47
126.40 <= SB
487 BoLA-DRB3_02601 DTVYWSDWASYSLDS 4 WSDWASYSL 0.980
0 VgR_1799_bp 0.571112 1.02 NA 0.487488 9.01
256.02 <= SB
271 BoLA-DRB3_02601 DPEPLLVFSTTKEIR 4 LLVFSTTKE 0.740
0 VgR_1799_bp 0.550831 1.09 NA 0.585831 1.11
88.34 <= SB
1510 BoLA-DRB3_02601 SPFSIALFEDWLYWS 4 IALFEDWLY 0.990
0 VgR_1799_bp 0.540770 1.13 NA 0.444104 17.39
409.39 <= SB
1606 BoLA-DRB3_02601 SDERYHINSSDILGQ 4 YHINSSDIL 1.000
0 VgR_1799_bp 0.537622 1.15 NA 0.455099 14.98
363.47 <= SB
1373 BoLA-DRB3_02601 DIHKPYHIAVDWVAG 5 YHIAVDWVA 0.970
0 VgR_1799_bp 0.509836 1.27 NA 0.422944 22.60
514.72 <= SB
718 BoLA-DRB3_02601 KPTITSYTMDGQNPR 3 ITSYTMDGQ 0.940
0 VgR_1799_bp 0.449569 1.61 NA 0.421246 23.06
524.26 <= SB
399 BoLA-DRB3_02601 PPQKVVYWTDWGSRP 4 VVYWTDWGS 1.000
0 VgR_1799_bp 0.436997 1.68 NA 0.412264 25.52
577.77 <= SB
580 BoLA-DRB3_02601 SFVIVAEEDLVYKID 3 IVAEEDLVY 0.860
0 VgR_1799_bp 0.433249 1.71 NA 0.534594 3.71
153.79 <= SB
834 BoLA-DRB3_02601 RRVIVAAQVPPFAPG 3 IVAAQVPPF 1.000
0 VgR_1799_bp 0.417543 1.81 NA 0.602011 0.71
74.15 <= SB
1413 BoLA-DRB3_02601 DTAYSHLNTFALSAN 3 YSHLNTFAL 1.000
0 VgR_1799_bp 0.391978 2.00 NA 0.534040 3.75
154.71 <= WB
391 BoLA-DRB3_02601 SPRAIIVNPPQKVVY 4 IIVNPPQKV 0.990
0 VgR_1799_bp 0.387862 2.04 NA 0.594614 0.86
80.33 <= WB
638 BoLA-DRB3_02601 AINVKSFEQWTVHDH 3 VKSFEQWTV 0.890
0 VgR_1799_bp 0.378836 2.13 NA 0.484248 9.54
265.16 <= WB
1743 BoLA-DRB3_02601 NPAFNTRKTELLSED 3 FNTRKTELL 1.000
0 VgR_1799_bp 0.336185 2.52 NA 0.445756 17.03
402.14 <= WB
663 BoLA-DRB3_02601 VTHQLLYWVDADKYT 4 LLYWVDADK 0.450
0 VgR_1799_bp 0.334517 2.54 NA 0.533558 3.79
155.52 <= WB
730 BoLA-DRB3_02601 NPRVLPLSTLLLPVS 3 VLPLSTLLL 0.990
0 VgR_1799_bp 0.319170 2.70 NA 0.586003 1.10
88.18 <= WB
1478 BoLA-DRB3_02601 KRIYWSDANKNVIES 3 YWSDANKNV 0.770
0 VgR_1799_bp 0.310694 2.79 NA 0.524457 4.51
171.62 <= WB
292 BoLA-DRB3_02601 NRYFEIHPAEAQAVG 3 FEIHPAEAQ 0.760
0 VgR_1799_bp 0.308150 2.82 NA 0.517302 5.22
185.43 <= WB
491 BoLA-DRB3_02601 WSDWASYSLDSSNKR 3 WASYSLDSS 0.940
0 VgR_1799_bp 0.281288 3.13 NA 0.495366 7.84
235.10 <= WB

775 BoLA-DRB3_02601 GTPFIVQQVKAHISS 4 IVQQVKAHI 0.620
0 VgR_1799_bp 0.268301 3.30 NA 0.505204 6.60
211.36 <= WB
658 BoLA-DRB3_02601 GIDFDVTHQLLYWVD 3 FDVTHQLLY 0.980
0 VgR_1799_bp 0.262000 3.39 NA 0.463562 13.24
331.67 <= WB
1517 BoLA-DRB3_02601 FEDWLYWSDWGSDSL 3 WLYWSDWGS 0.820
0 VgR_1799_bp 0.260237 3.41 NA 0.333562 51.16
1353.85 <= WB
1302 BoLA-DRB3_02601 GEPFLLYMLPNQIRS 3 FLLYMLPNQ 0.530
0 VgR_1799_bp 0.256183 3.46 NA 0.638267 0.23
50.09 <= WB
1286 BoLA-DRB3_02601 DGYALGADRRYCKVQ 2 YALGADRRY 1.000
0 VgR_1799_bp 0.252955 3.51 NA 0.374162 37.45
872.56 <= WB
641 BoLA-DRB3_02601 VKSFEQWTVHDHIGS 3 FEQWTVHDH 0.960
0 VgR_1799_bp 0.249250 3.57 NA 0.403827 27.97
632.99 <= WB
1337 BoLA-DRB3_02601 GMDYRVTDKSIFWTE 3 YRVTDKSIF 0.990
0 VgR_1799_bp 0.245816 3.62 NA 0.461187 13.71
340.30 <= WB
215 BoLA-DRB3_02601 NAGFRLLADHISCAD 3 FRLLADHIS 0.990
0 VgR_1799_bp 0.242725 3.67 NA 0.504132 6.73
213.83 <= WB
1518 BoLA-DRB3_02601 EDWLYWSDWGSDSLM 5 WSDWGSDSL 0.540
0 VgR_1799_bp 0.240624 3.71 NA 0.426794 21.57
493.72 <= WB
1729 BoLA-DRB3_02601 DEDYHAMNTTPGFIN 3 YHAMNTTPG 0.970
0 VgR_1799_bp 0.239725 3.72 NA 0.378971 35.92
828.32 <= WB
1245 BoLA-DRB3_02601 HPGYRLNTDRKSCDD 3 YRLNTDRKS 1.000
0 VgR_1799_bp 0.226678 3.93 NA 0.305164 60.61
1840.83 <= WB
1454 BoLA-DRB3_02601 GTARVVLLTDKILWP 4 VVLLTDKIL 0.960
0 VgR_1799_bp 0.215918 4.12 NA 0.441974 17.87
418.94 <= WB
1417 BoLA-DRB3_02601 SHLNTFALSANDGLM 5 FALSANDGL 0.990
0 VgR_1799_bp 0.196163 4.51 NA 0.592254 0.92
82.41 <= WB
1385 BoLA-DRB3_02601 VAGNIYFTDGWVHIQ 3 NIYFTDGWV 0.810
0 VgR_1799_bp 0.195861 4.52 NA 0.559819 2.11
117.06 <= WB
1427 BoLA-DRB3_02601 NDGLMFWAVWHEVVQ 5 FWAVWHEVV 0.520
0 VgR_1799_bp 0.182556 4.83 NA 0.498247 7.46
227.89 <= WB
821 BoLA-DRB3_02601 HVSLRSTRNGTFSRR 3 LRSTRNGTF 1.000
0 VgR_1799_bp 0.175593 4.99 NA 0.480502 10.15
276.12 <= WB
294 BoLA-DRB3_02601 YFEIHPAEAQAVGVE 3 IHPAEAQAV 0.800
0 VgR_1799_bp 0.174161 5.03 NA 0.472257 11.57
301.89 <= WB
1051 BoLA-DRB3_02601 EDDFHCANGQCVDKR 3 FHCANGQCV 1.000
0 VgR_1799_bp 0.149662 5.72 NA 0.333285 51.25
1357.92 <= WB
1756 BoLA-DRB3_02601 EDGELKRWASSDSLQ 4 LKRWASSDS 0.880
0 VgR_1799_bp 0.144181 5.88 NA 0.355299 43.75
1070.12 <= WB

665 BoLA-DRB3_02601 HQLLYWVDADKYTLE 3 LYWVDADKY 0.420
0 VgR_1799_bp 0.142737 5.92 NA 0.479867 10.25
278.03 <= WB
686 BoLA-DRB3_02601 SGHVIIRDDLHRPVG 2 HVIIRDDLH 0.610
0 VgR_1799_bp 0.142231 5.94 NA 0.404139 27.88
630.86 <= WB
467 BoLA-DRB3_02601 KRDVVMHEEVFHPFA 3 VVMHEEVFH 0.850
0 VgR_1799_bp 0.141799 5.95 NA 0.432244 20.21
465.45 <= WB
311 BoLA-DRB3_02601 SDQHRVFWTDVSTRR 3 HRVFWTDVS 0.780
0 VgR_1799_bp 0.139449 6.02 NA 0.438485 18.66
435.05 <= WB
351 BoLA-DRB3_02601 DLSLDWVANNLYITD 3 LDWVANNLY 0.930
0 VgR_1799_bp 0.123524 6.58 NA 0.498988 7.37
226.07 <= WB
697 BoLA-DRB3_02601 RPVGVALYPFAGVLF 6 LYPFAGVLF 0.910
0 VgR_1799_bp 0.121316 6.68 NA 0.534600 3.71
153.78 <= WB
1402 BoLA-DRB3_02601 EPTFKHCTDVIDTAY 3 FKHCTDVID 1.000
0 VgR_1799_bp 0.119576 6.76 NA 0.364813 40.52
965.44 <= WB
337 BoLA-DRB3_02601 DFKTLFSAEKTLLED 5 FSAEKTLLE 0.620
0 VgR_1799_bp 0.101744 7.58 NA 0.444699 17.26
406.76 <= WB
1310 BoLA-DRB3_02601 LPNQIRSFSMHGHAQ 4 IRSFSMHGH 1.000
0 VgR_1799_bp 0.099730 7.68 NA 0.514318 5.52
191.52 <= WB
1684 BoLA-DRB3_02601 LLVLGYVLYRRNRDK 5 YVLYRRNRD 0.930
0 VgR_1799_bp 0.097188 7.81 NA 0.482114 9.89
271.35 <= WB
587 BoLA-DRB3_02601 EDLVYKIDLNKVGAP 4 YKIDLNKVG 0.680
0 VgR_1799_bp 0.092362 8.08 NA 0.457399 14.50
354.54 <= WB
306 BoLA-DRB3_02601 GVEFDSDQHRVFWTD 3 FDSDQHRVF 0.950
0 VgR_1799_bp 0.082959 8.72 NA 0.341718 48.39
1239.50 <= WB
422 BoLA-DRB3_02601 GTNIQQLVSTDLGWP 3 IQQLVSTDL 0.930
0 VgR_1799_bp 0.081610 8.82 NA 0.448390 16.43
390.84 <= WB
1722 BoLA-DRB3_02601 DEHPIAADEDYHAMN 4 IAADEDYHA 0.980
0 VgR_1799_bp 0.067657 10.00 NA 0.290287 65.36
2162.32 <= WB

Number of strong binders: 16 Number of weak binders: 42

Link to output xls file NetMHCIIpan_out.xls
